# Design and synthesis of 7-chloroquinoline-derivative-bearing urea, carbamate, or thiourea moieties and their antimalarial activity against *Plasmodium berghei* via the inhibition of β-hematin formation

**DOI:** 10.3389/fchem.2026.1827662

**Published:** 2026-05-29

**Authors:** Hegira Ramírez, Ali S. Mijoba, Sandra Espinosa, Arthur R. Barazarte, María E. Acosta, Jaime E. Charris, Esteban Fernandez-Moreira

**Affiliations:** 1 Dirección de Investigación, Universidad ECOTEC, Guayaquil, Ecuador; 2 Laboratorio de Síntesis Orgánica, Facultad de Farmacia, Universidad Central de Venezuela, Caracas, Venezuela; 3 Laboratory of Pathophysiology, Center for Experimental Medicine, Venezuelan Institute of Scientific Research (IVIC), Caracas, Venezuela; 4 Departamento de Química, Universidad Técnica Particular de Loja, Loja, Ecuador; 5 Departamento de Biología y Química, Universidad Pedagógica Experimental Libertador, Caracas, Venezuela; 6 Unidad de Bioquímica, Facultad de Farmacia, Universidad Central de Venezuela, Caracas, Venezuela; 7 Escuela de Medicina, Universidad Espíritu Santo, Samborondón, Ecuador; 8 Department of Cell Biology, Complutense University School of Medicine, Madrid, Spain

**Keywords:** carbamate, malaria, quinoline, thiourea, urea, β-hematin

## Abstract

We describe a short synthetic route to a new series of 7-chloroquinoline hybrids derived from albendazole-, thioacetazone-, and primaquine-based drugs. The design strategy incorporated a ureido group as a bioisostere of known inhibitors of β-hematin formation (βHF). Compounds **13**–**34** were characterized by infrared (IR), nuclear magnetic resonance (NMR), and elemental analysis. Preliminary *in vitro* screening identified compounds **13**, **14**, **16**–**18**, and **27**–**29** as inhibitors of βHF. Among these, compound **16** was the most active, exhibiting 90.68% ± 0.043% inhibition of βHF compared with 92.55% ± 0.019% for chloroquine (CQ). *In vivo* evaluation in *Plasmodium berghei* ANKA, a CQ-susceptible murine malaria strain, revealed that compound **16** reduced parasitemia to 1.2% ± 0.12% and extended survival to 27.33 ± 0.15 days. This compares favorably with chloroquine (parasitemia: 0.88% ± 0.09%; survival: 29.6 ± 0.13 days). Furthermore, at a tested concentration of 10 mM, compound **16** showed 17.23 ± 0.13 hemolysis, compared to 29.65 ± 0.10% for CQ, indicating no marked lytic action on erythrocytes. This study evaluated the hepatological consequences of malaria infection in mice infected with *P*. *berghei*. Based on its potent *in vitro* and *in vivo* activity, favorable hemolytic profile, and hepatoprotective effects, compound **16** emerges as a promising new candidate for antimalarial development.

## Introduction

Malaria is a life-threatening infectious disease transmitted through the bite of female *Anopheles* mosquitoes, which serve as vectors for *Plasmodium* spp., a genus of unicellular protozoan parasites (Phylum: Apicomplexa). According to the World Health Organization (WHO), the global number of cases in 2023 was 263 million, with approximately 597,000 deaths, many of them children under the age of five (World Malaria Report, 2024). Malaria is caused by six species of *Plasmodium*: *P. falciparum*, *P. vivax*, *P. ovale curtisi*, *P. ovale wallikeri*, *P. malariae*, and occasionally *P. knowlesi*. Among these, *P. falciparum* and *P. vivax* are the most dangerous species worldwide, are often fatal to humans, and are now present in areas containing more than 40% of the world’s population (Plewes et al., 2019).

Despite the effectiveness of artemisinin-based combination therapies, the emergence of resistant parasite strains poses a significant challenge to current treatment strategies (Blasco et al., 2017). Determining the mechanism of action of antimalarial drugs remains a significant challenge in malaria research, despite recent advances in molecular and proteomic technologies (Aguiar et al., 2019; Carolino and Winzeler, 2020; Lu et al., 2021). The heme metabolism remains an attractive target because *Plasmodium* parasites rely on hemoglobin degradation, leading to the release of toxic free heme that must be detoxified into hemozoin. Interference with this process has been a successful strategy for several antimalarial drugs, including chloroquine and artemisinin derivatives (Kapishnikov et al., 2021; Quadros et al., 2022; Sigala and Goldberg, 2014).

Since the discovery of quinine and the potent antimalarial activity of its quinoline nucleus, significant efforts have been made to obtain new natural or synthetic structures containing this nucleus to provide novel treatments for this problem (Poje et al., 2022; Vijayan et al., 2021). As a privileged fragment, quinoline is a rigid, planar molecule and is a pharmacophore present in the core of numerous physiologically active agents that display interesting therapeutic properties (Matada et al., 2021). Structurally, quinoline can be readily modified with a broad range of substituents to provide the molecular diversity necessary to achieve a library of compounds, among which different members can show different biological effects (Kaur and Kumar, 2021; Li et al., 2022; Pepe et al., 2020; Pósa et al., 2022; Zhang et al., 2022).

Urea, carbamate, and thiourea represent privileged functional groups that are well positioned in medicinal and synthetic chemistry, particularly in medicinal chemistry due to their ability to form stable hydrogen bonds with elements that recognize biological targets, such as proteins, enzymes, and receptors. These structural motifs constitute a common framework for a variety of drugs and bioactive compounds endowed with a broad range of therapeutic and pharmacological properties, including antiviral, anticonvulsant, anti-inflammatory, antimicrobial, antimalarial, and antitumor effects (Garuti et al., 2016; Ghosh and Brindisi, 2024; Hashem et al., 2020; Keche et al., 2012; Matošević and Bosak, 2020; Shimshoni et al., 2007).

**FIGURE 1:**
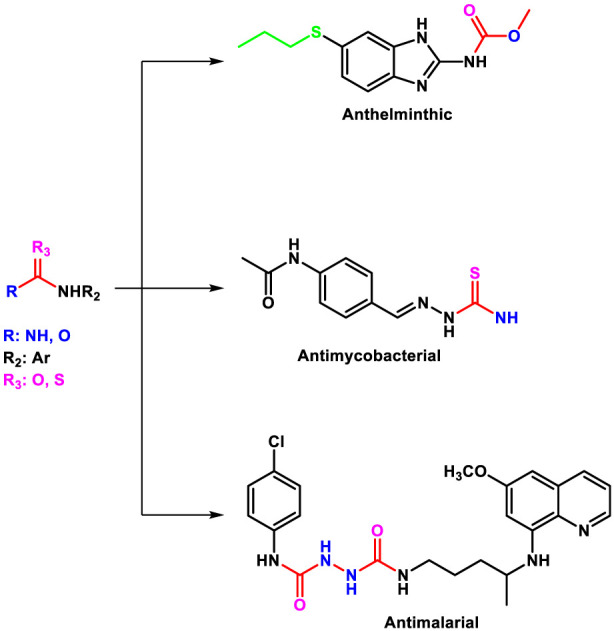
Compounds used as leads for the design of **13**–**34** through tripartite molecular hybridization.

One strategy for the discovery of new drugs is combining two or more pharmacophoric moieties in a single molecule to obtain a synergistic effect or to obtain new agents with a novel mode of action. The quinoline, urea, carbamate, and thiourea moieties have demonstrated potent chemotherapeutic activity individually in certain molecules where they are present. For example, the drug albendazole, which contains carbamate and mercaptan groups, exhibits anthelmintic activity (Matošević and Bosak, 2020). Similarly, the drug thioacetazone, which includes the thiourea functional group, shows antimycobacterial activity (Choi and Jee, 2015). Additionally, the hybrid drug primaquine, which features a double urea function and a quinoline nucleus in its structure, demonstrates antimalarial activity (Nudelman, 2025) (Figure 1).

**FIGURE 2:**
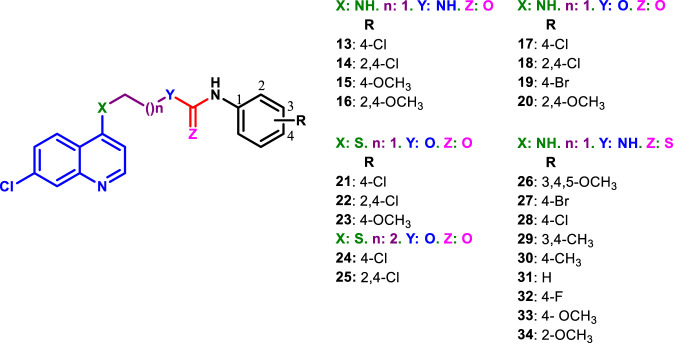
Chemical structures of the compounds **13**–**34**.

Based on these precedents, this study presents the synthesis of hybrid molecules assembled through three pharmacophores consisting of a 7-chloroquinoline core connected via linkers—ethylenediamine, 2-aminoethan-1-ol, 2-mercaptoethan-1-ol, and 3-mercaptopropan-1-ol—to the functional groups of the bioisosteres urea, carbamate, and thiourea, with structural modifications that incorporate electron-withdrawing or -donating groups to confer promising antimalarial properties. This approach obtained compounds **13**–**34**, shown in this study and represented by the general structure in Figure 2. The study also reports the compounds’ potential effect as inhibitors of βHF, their *in vivo* antimalarial activity, their effect on red blood cells (RBCs), molecular docking studies, and their impact on liver markers.

## Materials and methods

### Chemistry

FT-IR spectroscopy was performed on a Perkin-Elmer™ Spectrum two using a Diamond/ZnSe attenuated total reflectance (ATR) sampling accessory (Waltham, MA, United States). Readings were taken at room temperature, 64 scans/min per analysis, at a resolution of 0.5 cm^-1^ over a range of 4,000–450 cm^-1^. The ^1^H and ^13^C NMR spectra were acquired using a Nanalysis™ 100 MHz PRO Benchtop spectrometer (Calgary, AB, Canada) or a Bruker™ Magnet System 500′54 Ascend ULH spectrometer (Billerica, MA, United States) (at 100 and 500 MHz for ^1^H and 25.8 and 125 MHz for ^13^C) using CDCl_3_ or DMSO-*d*_6_ as the solvents; these are reported in ppm downfield from the residual CHCl_3_ δ 7.25 for ^1^H NMR and 77.0 for ^13^C NMR or DMSO at δ 2.54 ppm for ^1^H NMR and 44.5 ppm for ^13^C NMR, respectively. Elemental analyses were performed using a Perkin Elmer™ 2400 CHN elemental analyzer, with the results within ±0.4% of the predicted values. Melting points were determined on a Fisher-Johns™ fusiometer (Thermo Fisher Scientific, Waltham, MA, United States) and were uncorrected. Thin-layer chromatography (TLC) was carried out on Merck™ silica gel F254 0.255-mm plates (Darmstadt, Germany), and spots were visualized by UV fluorescence at 254 nm. Chemical reagents were obtained from Aldrich Chemical Co™ (St. Louis, MO, United States). All solvents were distilled and dried in the usual manner.

#### General procedure for the synthesis of compounds **13**–**34**

To a solution of *N* ^*1*^-(7-chloroquinolin-4-yl) were substituted derivatives **6**–**9** (0.1 mmol) and 4,4-dimethylaminopyridine (DMAP) (0.1 mmol) in dry *N,N*-dimethylformamide (DMF) (8 mL). The mixture was stirred at 50 °C for 20 min under a nitrogen atmosphere. Either (0.2 mmol) of phenylisocyanate **11a**–**i** or phenylisothiocyanate **12a**–**e** were added, and the mixture was stirred at 50 °C for 2 h. After completion of the reaction, ice was added, and the resulting solid was filtered, washed with water and acetone, and purified by recrystallization.

#### 1-(4-chlorophenyl)-3-(2-((7-chloroquinolin-4-yl)amino)ethyl)urea **13**

White solid; yield: 87% (EtOH); m. p. 226 °C, (lit. 228–230 °C) (Nava-Zuazo et al., 2010). IR cm^−1^: 3363, 3301, 1624, 1613, and 1587. ^1^H NMR (DMSO-*d*_6_, 500 MHz): δ 3.51–3.5 (4H, m, HN(CH_2_)_2_NH), 6.39 (H, t, NH, *J* = 5 Hz), 6.57 (1H, d, *J* = 5 Hz, H3), 7.24 (2H, d, *J* = 9 Hz, H3′,5′), 7.42–7.47 (3H, m, H6, H2′,6′), 7.78 (1H, d, *J* = 2 Hz, H8), 8.21 (1H, d, *J* = 9 Hz, H5), 8.38 (1H, d, *J* = 5 Hz, H2), and 8.77 (1H, s, NH). ^13^C NMR (DMSO-*d*_6_, 125.7 MHz): δ 38.2, 43.4, 99.2, 119.7, 117.9, 120.3, 124.4, 124.6, 125.1, 128.9, 129.1, 139.8, 149.5, 150.6, 152.4, and 156.0. Anal. Calcd for C_18_H_16_Cl_2_N_4_O: C, 57.61; H, 4.30; and N, 14.93. Found: C, 57.59; H, 4.32; and N, 15.13.

#### 1-(2-((7-chloroquinolin-4-yl)amino)ethyl)-3-(2,4-dichlorophenyl)urea **14**

White solid; yield: 85% (EtOH); m. p. 248–249 °C. IR cm^−1^: 3381, 3292, 1633, 1617, and 1591. ^1^H NMR (DMSO-*d*_6_, 500 MHz): δ 3.37–3.41 (4H, m, HN(CH_2_)_2_NH), 6.57 (1H, d, *J* = 5 Hz, H3), 7.24 (H, t, NH, *J* = 5 Hz), 7.31 (H, d, *J* = 9 Hz, H6′), 7.42–7.46 (2H, m, H6, NH), 7.52 (1H, s, H3′), 7.75 (1H, s, H8), 8.15–8.23 (2H, m, H5, H6′), and 8.39 (1H, d, *J* = 5 Hz, H2). ^13^C NMR (DMSO-*d*_6_, 125.7 MHz): δ 38.2, 43.2, 99.2, 117.9, 122.4, 122.6, 124.5, 124.6, 125.9, 127.9, 128.0, 128.9, 133.9, 136.3, 149.5, 150.6, 152.4, and 155.6. Anal. Calcd for C_18_H_15_Cl_3_N_4_O: C, 52.77; H, 3.69; and N, 13.68. Found: C, 52.78; H, 3.73; and N, 13.51.

#### 1-(2-((7-chloroquinolin-4-yl)amino)ethyl)-3-(4-methoxyphenyl)urea **15**

White solid; yield: 86% (EtOH); m. p. 198–199 °C. IR cm^−1^: 3372, 3298, 1641, 1624, and 1585. ^1^H NMR (DMSO-*d*_6_, 500 MHz): δ 3.34 (2H, t, *J* = 5 Hz, HN(CH_2_)_2_NH),3.37 (2H, m, HN(CH_2_)_2_NH), 3.67 (3H, s, OCH_3_), 6.26 (1H, t, *J* = 5 Hz, NH), 6.56 (1H, d, *J* = 5 Hz, H3), 6.80 (2H, d, *J* = 9 Hz, H3′,5′), 7.29 (2H, d, *J* = 9 Hz, 2′,6′), 7.43–7.46 (2H, m, H6, NH), 7.78 (1H, s, H8), 8.20 (1H, d, *J* = 9 Hz, H5), 8.38 (1H, d, *J* = 5 Hz, H2), and 8.41 (1H, s, NH). ^13^C NMR (DMSO-*d*_6_, 125.7 MHz): δ 38.3, 43.7, 55.6, 99.2, 114.4, 117.7, 120.3, 120.4, 124.4, 124.6, 127.9, 133.4, 133.8, 149.5, 150.7, 154.6, and 156.5. Anal. Calcd for C_19_H_19_ClN_4_O_2_: C, 61.54; H, 5.16; and N, 15.11. Found: C, 61.57; H, 5.18; and N, 14.96.

#### 1-(2-((7-chloroquinolin-4-yl)amino)ethyl)-3-(2,4-dimethoxyphenyl)urea **16**

White solid; yield: 67% (EtOH); m. p. 216–217 °C. IR cm^−1^: 3366, 3332, 1645, 1612, and 1568. ^1^H NMR (DMSO-*d*_6_, 500 MHz): δ 3.34 (2H, t, *J* = 5 Hz, HN(CH_2_)_2_NH), 3.37 (2H, m, HN(CH_2_)_2_NH), 3.69 (3H, s, OCH_3_), 3.76 (3H, s, OCH_3_), 6.43 (1H, d, *J* = 8 Hz, H5′), 6.54 (1H, m, H3, H3′), 6.88 (1H, t, *J* = 5 Hz, NH), 7.43–7.46 (2H, m, H6, NH), 7.74 (1H, s, NH), 7.78 (1H, s, H8), 7.83 (1H, d, *J* = 9 Hz, H6′), 8.20 (1H, d, *J* = 9 Hz, H5), and 8.38 (1H, d, *J* = 5 Hz, H2). ^13^C NMR (DMSO-*d*_6_, 125.7 MHz): δ 38.2, 43.7, 55.7, 56.1, 99.2, 104.5, 117.8, 120.5, 122.8, 124.4, 124.6, 127.9, 133.4, 133.9, 149.5, 149.7, 150.6, 152.4, 155.2, and 156.6. Anal. Calcd for C_20_H_21_ClN_4_O_3_: C, 59.93; H, 5.28; and N, 13.98. Found: C, 60.05; H, 5.31; and N, 14.27.

#### 2-((7-chloroquinolin-4-yl)amino)ethyl (4-chlorophenyl)carbamate **17**

White solid; yield: 63% (EtOH); m. p. 212–213 °C. IR cm^−1^: 3357, 3308, 1637, 1614, and 1582. ^1^H NMR (DMSO-*d*_6_, 500 MHz): δ 3.34 (2H, q, *J* = 5 Hz, HN(CH_2_)_2_NH), 3.65 (2H, t, *J* = 5 Hz, HN(CH_2_)_2_NH), 6.47 (1H, d, *J* = 5 Hz, H3), 7.25 (1H, t, *J* = 5 Hz, NH), 7.29 (2H, d, *J* = 8.5 Hz, H3′,H5′), 7.41 (1H, dd, *J* = 9 Hz, 2 Hz, H6), 7.46 (2H, d, *J* = 9 Hz, H2′, 6′), 7.77 (1H, s, H8), 8.23 (1H, d, *J* = 9 Hz, H5), 8.36 (1H, d, *J* = 5 Hz, H2), and 8.83 (s, 1H, NH). ^13^C NMR (DMSO-*d*_6_, 125.7 MHz): δ 45.6, 59.2, 99.2, 117.9, 120.3, 124.5, 126.0, 127.9, 129.1, 133.9, 138.9, 149.5, 150.8, and 152.3. Anal. Calcd for C_18_H_15_Cl_2_N_3_O_2_: C, 57.46; H, 4.02; and N, 11.17. Found: C, 57.46; H, 3.98; and N, 11.41.

#### 2-((7-chloroquinolin-4-yl)amino)ethyl (2,4-dichlorophenyl)carbamate **18**

White solid; yield: 69% (EtOH); m. p. 202–203 °C. IR cm^−1^: 3303, 3070, 1646, 1614, and 1581. ^1^H NMR (DMSO-*d*_6_, 500 MHz): δ 3.34 (2H, q, *J* = 5 Hz, HN(CH_2_)_2_NH), 3.65 (2H, t, *J* = 5 Hz, HN(CH_2_)_2_NH), 6.46 (1H, d, *J* = 5 Hz, H3), 7.42 (1H, t, *J* = 5 Hz, NH), 7.35 (1H, d, *J* = 9 Hz, H5′), 7.40 (1H, dd, *J* = 9 Hz, 2 Hz, H6), 7.58 (1H, s, H3′), 7.78 (1H, s, H8), 8.06 (1H, d, *J* = 9 Hz, H6′), 8.22 (1H, d, *J* = 9 Hz, H5), 8.35 (1H, d, *J* = 5 Hz, H2), and 9.12 (1H, s, NH). ^13^C NMR (DMSO-*d*_6_, 125.7 MHz): δ 45.6, 59.2, 99.2, 117.9, 123.7, 123.9, 124.5, 127.3, 127.8, 128.0, 129.1, 129.4, 133.9, 135.3, 149.5, 150.8, and 152.3. Anal. Calcd for C_18_H_14_Cl_3_N_3_O_2_: C, 52.64; H, 3.44; and N, 10.23. Found: C, 52.67; H, 3.45; and N, 10.47.

#### 2-((7-chloroquinolin-4-yl)amino)ethyl (4-bromophenyl)carbamate **19**

White solid; yield: 91% (EtOH); m. p. 209–211 °C. IR cm^−1^: 3297, 3073, 1633, 1585, and 1557. ^1^H NMR (DMSO-*d*_6_, 500 MHz): δ 3.34 (2H, q, *J* = 5 Hz, HN(CH_2_)_2_NH), 3.64 (2H, t, *J* = 5 Hz, HN(CH_2_)_2_NH), 6.48 (1H, d, *J* = 5 Hz, H3), 7.24 (1H, t, *J* = 5 Hz, NH), 7.42 (5H, brs, H6, H2′,3′,5′,6′), 7.77 (1H, s, H8), 8.23 (1H, d, *J* = 9 Hz, H5), 8.36 (1H, d, *J* = 5 Hz, H2), and 8.83 (1H, s, NH). ^13^C NMR (DMSO-*d*_6_, 125.7 MHz): δ 45.6, 59.2, 99.2, 113.9, 117.9, 120.7, 124.5, 127.9, 131.9, 133.9, 139.4, 149.5, 150.8, 152.3, and 152.8. Anal. Calcd for C_18_H_15_BrClN_3_O_2_: C, 51.39; H, 3.59; and N, 9.99. Found: C, 51.43; H, 3.66; and N, 10.23.

#### 2-((7-chloroquinolin-4-yl)amino)ethyl (2,4-dimethoxyphenyl)carbamate **20**

White solid; yield: 61% (EtOH); m. p. 206–208 °C. IR cm^−1^: 3303, 2965, 1644, 1614, and 1581. ^1^H NMR (DMSO-*d*_6_, 500 MHz): δ 3.34 (2H, t, *J* = 5 Hz, HN(CH_2_)_2_NH), 3.65 (2H, m, HN(CH_2_)_2_NH), 3.71 (3H, s, OCH_3_), 3.82 (3H, s, OCH_3_), 6.43 (1H, d, *J* = 9 Hz, H5′), 6.47 (1H, d, *J* = 5, H3), 6.57 (1H, s, 1H, H3′), 7.24 (1H, t, *J* = 5 Hz, NH), 7.42 (1H, d, *J* = 9 Hz, H6′), 7.78 (1H, s, H8), 7.86 (1H, d, *J* = 9 Hz, H6), 8.24 (1H, d, *J* = 9 Hz, H5), 8.36 (1H, d, *J* = 5 Hz, H2), and 8.45 (s, 1H, NH). ^13^C NMR (DMSO-*d*_6_, 125.7 MHz): δ 45.6, 55.7, 56.2, 59.2, 99.2, 104.5, 117.9, 120.9, 122.6, 124.5, 124.6, 127.9, 133.9, 149.6, 150.0, 150.7, 155.4, and 156.3. Anal. Calcd for C_20_H_20_ClN_3_O_4_: C, 59.78; H, 5.02; and N, 10.46. Found: C, 59.73; H, 4.98; and N, 10.67.

#### 2-((7-chloroquinolin-4-yl)thio)ethyl (4-chlorophenyl)carbamate **21**

White solid; yield: 46% (EtOH); m. p. 173–174 °C. IR cm^−1^: 3229, 3190, 1725, 1602, and 1543. ^1^H NMR (DMSO-*d*_6_, 500 MHz): δ 3.56 (2H, t, *J* = 6 Hz, S(CH_2_)_2_O), 4.39 (2H, t, *J* = 6 Hz, S(CH_2_)_2_O), 7.30 (2H, d, *J* = 9 Hz, H3′,5′), 7.45 (2H, d, *J* = 9 Hz, H2′,6′), 7.57 (1H, d, *J* = 5 Hz, H3), 7.63 (1H, d, *J* = 9 Hz, H6), 8.03 (1H, s, H8), 8.07 (1H, d, *J* = 9 Hz, H5), 8.72 (1H, d, *J* = 5 Hz, H2), and 8.83 (s, 1H, NH). ^13^C NMR (DMSO-*d*_6_, 125.7 MHz): δ 29.8, 62.2, 117.3, 120.3, 124.8, 125.7, 126.7, 127.7, 128.8, 129.1, 134.9, 138.4, 146.6, 147.9, 151.3, and 153.6. Anal. Calcd for C_18_H_14_Cl_2_N_2_O_2_S: C, 54.97; H, 3.59; and N, 7.12. Found: C, 55.05; H, 3.63; and N, 7.32.

#### 2-((7-chloroquinolin-4-yl)thio)ethyl (2,4-dichlorophenyl)carbamate **22**

White solid; yield: 82% (EtOH); m. p. 134–136 °C. IR cm^−1^: 3282, 3072, 1694, 1644, and 1529. ^1^H NMR (DMSO-*d*_6_, 500 MHz): δ 3.54 (2H, t, *J* = 6 Hz, S(CH_2_)_2_O), 4.39 (2H, t, *J* = 6 Hz, S(CH_2_)_2_O), 7.36 (1H, d, *J* = 9 Hz, H6), 7.53–7.59 (3H, m, H3′,3, 5), 7.63 (1H, d, *J* = 9 Hz, H6′), 8.03 (1H, s, H8), 8.07 (1H, dd, *J* = 9 Hz, 4 Hz, H5′), 8.72 (1H, d, *J* = 5 Hz, H2), and 9.24 (s, 1H, NH). ^13^C NMR (DMSO-*d*_6_, 125.7 MHz): δ 29.8, 62.7, 117.3, 123.7, 124.8, 125.7, 127.6, 128.0, 128.1, 128.8, 129.4, 134.5, 134.9, 135.3, 146.6, 147.9, 151.3, and 154.2. Anal. Calcd for C_18_H_13_Cl_3_N_2_O_2_S: C, 50.55; H, 3.06; and N, 6.55. Found: C, 50.61; H, 2.98; and N, 6.73.

#### 2-((7-chloroquinolin-4-yl)thio)ethyl (4-methoxyphenyl)carbamate **23**

White solid; yield: 95% (EtOH); m. p. 163–164 °C. IR cm^−1^: 3270, 3168, 1694, 1636, 1599, and 1559. ^1^H NMR (DMSO-*d*_6_, 500 MHz): δ 3.53 (2H, q, J = 6 Hz, S(CH_2_)_2_NH), 3.68 (3 h, s, OCH_3_), 4.37 (2H, t, *J* = 6 Hz, HN(CH_2_)_2_O), 6.83 (2H, d, *J* = 9 Hz, H3′,5′), 7.32 (2H, d, *J* = 9 Hz, 2′, 6′), 7.56 (1H, d, *J* = 5 Hz, H3), 7.62 (1H, d, *J* = 9 Hz, H6), 8.02 (1H, s, H8), 8.06 (1H, d, *J* = 9 Hz, H5), 8.72 (1H, d, *J* = 5, H2), and 9.54 (1H, s, NH). ^13^C NMR (DMSO-*d*_6_, 125.7 MHz): δ 29.9, 55.6, 61.9, 114.4, 117.3, 120.4, 124.8, 125.7, 127.7, 128.8, 133.4, 134.9, 139.4, 146.7, 147.9, 151.2, and 154.8. Anal. Calcd for C_19_H_17_ClN_2_O_3_S: C, 58.69; H, 4.41; and N, 7.20. Found: C, 58.73; H, 4.41; and N, 7.42.

#### 3-((7-chloroquinolin-4-yl)thio)propyl (4-chlorophenyl)carbamate **24**

White solid; yield: 61% (EtOH); m. p. 180–181 °C. IR cm^−1^: 3290, 3076, 1699, 1645, and 1558. ^1^H NMR (DMSO-*d*_6_, 500 MHz): δ 2.07 (2H, q, *J* = 7 Hz, S(CH_2_)_3_O), 3.30 (2H, t, *J* = 6 Hz, S(CH_2_)_3_O), 4.23 (2H, t, *J* = 6 Hz, HN(CH_2_)_2_O), 7.31 (2H, d, *J* = 8 Hz, H3′,5′), 7.45–7.47 (3H, m, H 2′, 6′, 3), 7.63 (1H, d, *J* = 9 Hz, H6), 8.02 (1H, s, H8), 8.07 (1H, d, *J* = 9 Hz, H5), 8.71 (1H, d, *J* = 5, H2), and 9.81 (1H, s, NH). ^13^C NMR (DMSO-*d*_6_, 125.7 MHz): δ 27.1, 27.8, 63.4, 117.2, 120.3, 124.8, 125.8, 127.6, 128.7, 129.1, 134.9, 138.5, 146.7, 147.3, 147.9, 151.2, and 153.9. Anal. Calcd for C_19_H_16_Cl_2_N_2_O_2_S: C, 56.03; H, 3.96; and N, 6.88. Found: C, 56.01; H, 4.03; and N, 7.15.

#### 3-((7-chloroquinolin-4-yl)thio)propyl (2,4-dichlorophenyl)carbamate **25**

White solid; yield: 82% (EtOH); m. p. 139–140 °C. IR cm^−1^: 3296, 3085, 1683, 1637, and 1561. ^1^H NMR (DMSO-*d*_6_, 500 MHz): δ 2.06 (2H, q, *J* = 7 Hz, S(CH_2_)_3_O), 3.35 (2H, t, *J* = 6 Hz, S(CH_2_)_3_O), 4.22 (2H, t, *J* = 6 Hz, HN(CH_2_)_2_O), 7.38 (1H, d, *J* = 9 Hz, H6), 7.45(1H, d, J = 5 Hz, H3), 7.60–7.64 (2H, m, Ar), 8.02 (2H, m, H8, 3′), 8.07 (1H, m, H5, 6′), 8.71(1H, d, *J* = 5, H2), and 9.11 (1H, brs, NH). ^13^C NMR (DMSO-*d*_6_, 125.7 MHz): δ 27.0, 27.9, 63.8, 117.1, 123.7, 124.8, 125.8, 127.6, 128.1, 128.7, 129.1, 129.4, 134.7, 134.9, 135.3, 147.6, 147.9, 151.2, and 154.3. Anal. Calcd for C_19_H_15_Cl_3_N_2_O_2_S: C, 51.66; H, 3.42; and N, 6.34. Found: C, 51.60; H, 3.43; and N, 6.15.

#### 1-(2-((7-chloroquinolin-4-yl)amino)ethyl)-3-(3,4,5-trimethoxyphenyl)thiourea **26**

White solid; yield: 67% (EtOH); m. p. 249–250 °C. IR cm^−1^: 3370, 3279, 2936, 1606, 1579, 1448, and 1125. ^1^H NMR (DMSO-*d*_6_, 100 MHz): δ 3.55 (2H, t, *J* = 5 Hz, HN(CH_2_)_2_NH), 3.67 (3H, s, OCH_3_), 3.71 (6 h, s, OCH_3_), 3.84 (2H, t, *J* = 6 Hz, HN(CH_2_)_2_NH), 6.65 (2H, s, H2′,6′), 6.65 (1H, d, *J* = 6 Hz, H3), 6.47 (1H, dd, *J* = 9 Hz, 2 Hz, H6), 7.82 (1H, d, *J* = 2 Hz, H8), 8.22 (1H, d, *J* = 9 Hz, H5), 8.44 (1H, d, *J* = 6 Hz, H2), and 9.57 (s, 1H, NH). ^13^C NMR (DMSO-*d*_6_, 25.8 MHz): δ 38.4, 43.0, 56.4, 60.6, 99.3, 102.2, 117.8, 124.6, 124.6, 127.9, 134.0, 134.5, 135.6, 149.4, 150.9, 152.4, 153.4, and 180.9. Anal. Calcd for C_21_H_23_ClN_4_O_3_S: C, 56.43; H, 5.19; and N, 12.54. Found: C, 56.49; H, 5.23; and N, 12.75.

#### 1-(4-bromophenyl)-3-(2-((7-chloroquinolin-4-yl)amino)ethyl)thiourea **27**

Brown solid; yield: 53% (EtOH); m. p. 264–266 °C. IR cm^−1^: 3292, 3170, 2960, 1610, 1587, 1488, and 1246. ^1^H NMR (DMSO-*d*_6_, 100 MHz): δ 3.81 (2H, t, *J* = 7 Hz, HN(CH_2_)_2_NH), 4.24 (2H, t, *J* = 7 Hz, HN(CH_2_)_2_NH), 7.47 (4H, s, H2′,3′,5′,6′), 7.64 (1H, d, *J* = 4 Hz, H3), 7.68 (1H, dd, *J* = 9 Hz, 2 Hz, H6), 8.07 (1H, d, *J* = 9 Hz, H5), 8.16 (1H, d, *J* = 2 Hz, H8), 8.84 (1H, s, NH), and 9.05 (1H, d, *J* = 4 Hz, H2). ^13^C NMR (DMSO-*d*_6_, 25.8 MHz): δ 38.2, 43.4, 99.2, 119.7, 117.9, 120.3, 124.4, 124.6, 125.1, 128.9, 129.1, 139.8, 149.5, 150.6, 152.4, and 156.0. Anal. Calcd for C_18_H_16_BrClN_4_S: C, 49.61; H, 3.70; and N, 12.86. Found: C, 49.61; H, 3.72; and N, 13.09.

#### 1-(4-chlorophenyl)-3-(2-((7-chloroquinolin-4-yl)amino)ethyl)thiourea **28**

Beige solid; yield: 72% (EtOH); m. p. 274–276 °C. IR cm^−1^: 3293, 3171, 2973, 1622, 1589, 1491, and 1239. ^1^H NMR (DMSO-*d*_6_, 100 MHz): δ 3.85 (2H, t, *J* = 7 Hz, HN(CH_2_)_2_NH), 4.14 (2H, t, *J* = 7 Hz, HN(CH_2_)_2_NH), 7.26–7.68 (6H, m, NH, H3,6, 2′,3′,5′,6′), 8.02 (1H, d, *J* = 9 Hz, H5), 8.11 (1H, d, *J* = 2 Hz, H8), 8.87 (1H, brs, NH), and 8.97 (1H, d, *J* = 4 Hz, H2). ^13^C NMR (DMSO-*d*_6_, 25.8 MHz): δ 38.7, 42.7, 53.0, 120.6, 120.8, 126.3, 126.9, 127.7, 128.1, 138.3, 146.9, 152.9, and 156.7. Anal. Calcd for C_18_H_16_Cl_2_N_4_S: C, 55.25; H, 4.12; and N, 14.32. Found: C, 55.14; H, 4.07; and N, 14.47.

#### 1-(2-((7-chloroquinolin-4-yl)amino)ethyl)-3-(3,4-dimethylphenyl)thiourea **29**

Beige solid; yield: 63% (EtOH); m. p. 266–268 °C. IR cm^−1^: 3308, 3040, 2919, 1640, 1542, 1445, and 1228. ^1^H NMR (DMSO-*d*_6_, 100 MHz): δ 2.16 (3H, s, CH_3_), 2.19 (3H, s, CH_3_), 3.82 (2H, t, *J* = 6 Hz, HN(CH_2_)_2_NH), 4.15 (2H, t, *J* = 6 Hz, HN(CH_2_)_2_NH), 6.97-7.21 (3H, m, H2′,5′,6′), 7.59 (1H, d, *J* = 4 Hz, H3), 7.65 (dd, *J* = 9 Hz, 2 Hz, H6), 8.04 (1H, d, *J* = 9 Hz, H5), 8.13 (1H, d, *J* = 2 Hz, H8), 8.37 (1H, brs, NH), and 8.99 (1H, d, *J* = 4 Hz, H2). ^13^C NMR (DMSO-*d*_6_, 25.8 MHz): δ 18.9, 19.9, 38.9, 42.8, 53.1, 116.3, 120.1, 120.9, 126.9, 127.6, 128.1, 130.1, 135.1, 138.3, 146.9, 149.8, and 152.9. Anal. Calcd for C_20_H_21_ClN_4_S: C, 62.41; H, 5.50; and N, 14.56. Found: C, 62.47; H, 5.52; and N, 14.76.

#### 1-(2-((7-chloroquinolin-4-yl)amino)ethyl)-3-(p-tolyl)thiourea **30**

Brown solid; yield: 73% (EtOH); m. p. 268–270 °C. IR cm^−1^: 3287, 3038, 2876, 1636, 1590, 1494, and 1237. ^1^H NMR (DMSO-*d*_6_, 100 MHz): δ 2.24 (3H, s, CH_3_), 3.82 (2H, t, *J* = 6 Hz, HN(CH_2_)_2_NH), 4.17 (2H, t, *J* = 6 Hz, HN(CH_2_)_2_NH), 7.06 (2H, d, *J* = 8 Hz, H3′,5′), 7.32 (2H, d, *J* = 4 Hz, H3), 7.61–7.69 (2H, m, H6, 5), 8.04 (1H, d, *J* = 8 Hz, H5), 8.12 (1H, d, *J* = 2 Hz, H8), 8.43 (1H, brs, NH), and 8.94 (1H, d, *J* = 4 Hz, H2). ^13^C NMR (DMSO-*d*_6_, 25.8 MHz): δ 20.7, 41.3, 53.1, 118.9, 120.9, 121.6, 124.4, 124.6, 126.9, 127.7, 128.1, 129.7, 130.1, 131.1, 131.4, 145.8, 151.2, and 152.9. Anal. Calcd for C_19_H_19_ClN_4_S: C, 61.53; H, 5.16; and N, 15.11. Found: C, 61.55; H, 5.22; and N, 15.33.

#### 1-(2-((7-chloroquinolin-4-yl)amino)ethyl)-3-phenylthiourea **31**

Brown solid; yield: 76% (EtOH); m. p. 276–278 °C. IR cm^−1^: 3134, 3040, 2973, 1648, 1567, 1495, and 1244. ^1^H NMR (DMSO-*d*_6_, 100 MHz): δ 3.80 (2H, t, *J* = 7 Hz, HN(CH_2_)_2_NH), 4.18 (2H, t, *J* = 7 Hz, HN(CH_2_)_2_NH), 7.02 (2H, t, *J* = 8 Hz, Ar), 7.22-7.69 (5H, m, Ar), 8.04 (1H, d, *J* = 9 Hz, H5), 8.11 (1H, d, *J* = 2 Hz, H8), 8.63 (1H, brs, NH), and 9.00 (1H, d, *J* = 4 Hz, H2). ^13^C NMR (DMSO-*d*_6_, 25.8 MHz): δ 42.8, 53.1, 118.8, 120.9, 122.4, 127.0, 127.6, 128.2, 129.3, 135.2, 137.7, 140.2, 146.6, 149.8, and 152.8. Anal. Calcd for C_18_H_17_ClN_4_S: C, 60.58; H, 4.80; and N, 15.70. Found: C, 60.64; H, 4.86; and N, 15.94.

#### 1-(2-((7-chloroquinolin-4-yl)amino)ethyl)-3-(4-fluorophenyl)thiourea **32**

Brown solid; yield: 53% (EtOH); m. p. 136–138 °C. IR cm^−1^: 3241, 3173, 2958, 1610, 1575, 1505, and 1210. ^1^H NMR (DMSO-*d*_6_, 100 MHz): δ 3.78 (2H, t, *J* = 6 Hz, HN(CH_2_)_2_NH), 4.16 (2H, t, *J* = 6 Hz, HN(CH_2_)_2_NH), 7.10 (2H, t, *J* = 8 Hz, H3′,5′), 7.37-7.70(4H, m, H3,6,2′,6′), 8.07 (1H, d, *J* = 8 Hz, H5), 8.11 (1H, d, *J* = 2 Hz, H8), 8.63 (1H, brs, NH), and 8.99 (1H, d, *J* = 4 Hz, H2). ^13^C NMR (DMSO-*d*_6_, 25.8 MHz): δ 41.2, 53.1, 115.2, 116.1, 118.4, 120.0, 120.7, 121.0, 121.3, 126.6, 127.7, 128.1, 129.7, 143.0, 144.7, 145.9, 149.6, and 152.8. Anal. Calcd for C_18_H_16_ClFN_4_S: C, 57.67; H, 4.30; and N, 14.95. Found: C, 57.69; H, 4.28; and N, 15.17.

#### 1-(2-((7-chloroquinolin-4-yl)amino)ethyl)-3-(4-methoxyphenyl)thiourea **33**

Brown solid; yield: 69% (EtOH); m. p. 218–220 °C. IR cm^−1^: 3275, 3142, 2934, 1614, 1584, 1452, and 1222. ^1^H NMR (DMSO-*d*_6_, 100 MHz): δ 3.71–3.86 (5H, m, OCH_3_ and HN(CH_2_)_2_NH), 4.16 (2H, t, *J* = 6 Hz, HN(CH_2_)_2_NH), 6.86 (2H, d, *J* = 9 Hz, H3′,5′), 7.33(2H, d, *J* = 9 Hz, H2′,6′), 7.54 (1H, d, *J* = 5 Hz, H3), 7.63 (1H, dd, J = 9 Hz, 2 Hz, H6), 8.04 (1H, d, *J* = 9 Hz, H5), 8.11 (1H, d, J = 2 Hz, H8), 8.33 (1H, s, NH), and 8.94 (1H, d, *J* = 5 Hz, H2). ^13^C NMR (DMSO-*d*_6_, 25.8 MHz): δ 40.9, 52.7, 55.7, 114.5, 120.8, 121.0, 124.4, 125.4, 126.8, 127.8, 132.9, 134.7, 152.3, and 155.1. Anal. Calcd for C_19_H_19_ClN_4_OS: C, 58.98; H, 4.95; and N, 14.48. Found: C, 59.06; H, 4.93; and N, 14.76.

#### 1-(2-((7-chloroquinolin-4-yl)amino)ethyl)-3-(2-methoxyphenyl)thiourea **34**

Brown solid; yield: 43% (EtOH); m. p. 206–208 °C. IR cm^−1^: 3144, 3038, 2968, 1608, 1522, 1431, and 1246. ^1^H NMR (DMSO-*d*_6_, 100 MHz): δ 3.73-3.90 (5H, m, OCH_3_ and HN(CH_2_)_2_NH), 4.19 (2H, t, *J* = 6 Hz, HN(CH_2_)_2_NH), 6.87-7.01 (2H, m, H3,6′), 7.60–7.84 (4H, m, H6, 3′,4′,5′), 8.12 (1H, d, *J* = 9 Hz, H5), 8.16 (1H, d, J = 2 Hz, H8), 8.86 (1H, s, NH), and 9.04 (1H, d, *J* = 5 Hz, H2). ^13^C NMR (DMSO-*d*_6_, 25.8 MHz): δ 40.4, 53.2, 56.1, 111.4, 120.8, 119.6, 122.8, 124.7, 126.6, 127.7, 128.0, 128.1, 134.9, 146.8, 149.8, 152.6, and 155.3. Anal. Calcd for C_19_H_19_ClN_4_OS: C, 58.98; H, 4.95; and N, 14.48. Found: C, 58.94; H, 5.03; and N, 14.66.

### Biology

#### Inhibition of β-hematin formation

The assay was performed as per Baelmans et al. (2000) and Dorn et al. (1995). Hemin chloride solution (50 μL, 4 mM) dissolved in dimethyl sulfoxide (DMSO) (5.2 mg mL^−1^) was distributed in 96-well microplates. The compounds were dissolved in DMSO, and different concentrations (5–100 μM) were added to the test wells (50 μL). Water (50 μL) and DMSO (50 μL) were used as controls. Experiments were performed in triplicate. Acetate buffer (100 μL, 0.2 M, pH 4.4) was used to generate βH. The plates were incubated at 37 °C for 48 h and centrifuged (4000 RPM × 15 min, IEC-CENTRA, MP4R). The supernatant was discarded, and the pellet was washed twice with DMSO (200 μL) and dissolved in NaOH (200 μL, 0.2 N). The aggregates were further solubilized with NaOH (0.1 N), and their absorbance values were recorded at 405 nm (BIORAD-550 microplate reader). Results are expressed as the percentage inhibition of βHF.

#### Parasite, experimental host, and strain maintenance

The mouse model protocol followed Dorn et al. (1995) and Kerns and Di (2008). Male BALB/c mice weighing 18–22 g were maintained on a commercial pellet diet and handled according to local and national regulations, and the research protocols were approved by the Institute of Biomedicine Committee on Animal Research. A rodent malaria ANKA strain of *P. berghei* was used to infect the animals. Mice were infected by intraperitoneal (i.p.) injection with 1 × 10^6^ infected erythrocytes diluted in phosphate-buffered saline (PBS; 10 mM, pH 7.4, 0.1 mL). Parasitemia was monitored by microscopic examination of Giemsa-stained smears.

#### 4-day suppressive test

The percentage of parasitemia and the survival times of mice infected with *P. berghei* and treated as per Charris et al. (2019) and Peters and Robinson (1999). BALB/c mice (18–22 g) were infected via the caudal vein with 10^6^ *P. berghei*-infected red blood cells (n = 4). Treatment began 2 h after infection with the active compounds from the *in vitro* test of βHF. The compounds were dissolved in DMSO (0.1 M) and subsequently diluted with a saline-Tween 20 solution (2%). Each compound (dose 20 mg kg^−1^) was administered i. p. for 4 days. On day 4, the parasite load was assessed by examining Giemsa-stained smears. CQ (20 mg kg^−1^) was used as a positive control. The survival time of mice infected with *P. berghei* and treated with saline solution was used as a baseline control. The results are expressed as the percentage of parasitemia, and the survival curve was based on the number of days of mouse survival after treatment compared with infected but untreated mice.

#### In vitro toxicity on mouse red blood cells

To evaluate the *in vitro* toxicological effect, we used a model based on the lysis of RBCs, measuring the hemoglobin released in the supernatant fraction (Crupi et al., 2019; Mehta et al., 1984). Hemoglobin released was measured using spectrophotometer at 550 nm. RBCs in 50% Alsever’s solution were centrifuged at 800 *g* for 10 min and then washed three times with saline solution to obtain 100% RBCs. The synthesized compounds at three concentrations (0.1, 1, and 10 mM) were incubated with a 2% final suspension of RBCs at 37 °C for 45 min. The release of hemoglobin by an equal number of RBCs after hypotonic lysis in 0.05 volumes of water was used as a 100% positive control, while RBCs treated with saline solution served as negative controls. Results were expressed as percentage hemolysis (%) for each concentration of each tested compound.

#### Determination of biochemical parameters

Another objective of the study was to determine the hepatological consequences of malaria infection and the effect caused by each molecule tested in mice infected with *P. berghei*. For this purpose, the concentrations of markers such as alanine aminotransferase (ALT), aspartate aminotransferase (AST), and gamma-glutamyl transferase (GGT) present in the serum were determined. For this determination, the method described by Wroblewski and Ladue (1956) was used, employing a commercial kit from STANBIO Laboratory. In brief, 1 mL of the reaction mixture (67 mM Tris, pH 7.5, 200 mM L-aspartate, 0.5 U MDH, 10 mM α-ketoglutarate, and 0.15 mM NADH (disodium salt)) was incubated for 3 min at 37 °C. Then, 0.1 mL of serum (test sample) was added. The change in absorbance was recorded at 1-min intervals for 3 min using a spectrophotometer (STAT FAX) at 340 nm for ALT and 405 nm for AST, respectively. To determine GGT, the method described by Rogić (2008) and the STANBIO Laboratory Kit was used. In briefly, 200 µL of the reaction mixture (0.032 mM L-γ-glutamyl-3-carboxy-4-nitroaniline, 4 mM Tris pH 8.5, and 4 mM glycylglycine) was incubated for 3 min at 37 °C. Then, 20 µL of serum (test sample) was added. The change in absorbance was recorded at 1-min intervals for 3 min using a plate reader at 405 nm.

### Molecular docking

#### Ligand preparation

Compound structures were constructed using ChemDraw and minimized in Chimera. The structures were then converted to pdbqt format using Open Babel 2.4.1 (Li et al., 2004).

#### Receptor preparation

βH is chemically, spectroscopically, and crystallographically identical to the malaria pigment hemozoin. The receptor structure was modeled from the crystal structure of the βH dimer obtained from the Cambridge Crystallographic Data Centre (CCDC) (Pagola et al., 2000). A 3 × 3 × 3 supercell was constructed using the supercell building function in VESTA (Momma and Izumi, 2006).

For receptor preparation, polar hydrogens and Gasteiger charges were assigned in AutoDock Tools. The search space was configured to encompass the entire crystal surface, with the center at (x, y, z): (13.8, 21.5, 11.9) and dimensions (x, y, z): (42.3, 44.9, 29.1).

#### Virtual screening

Virtual screening was performed on a library consisting of 7-chloroquinoline derivatives using AutoDock Vina software, which is based on a Lamarckian genetic algorithm and a hybrid scoring function (Trott and Olson, 2010).

## Results and discussion

### Chemistry

**SCHEME 1:**
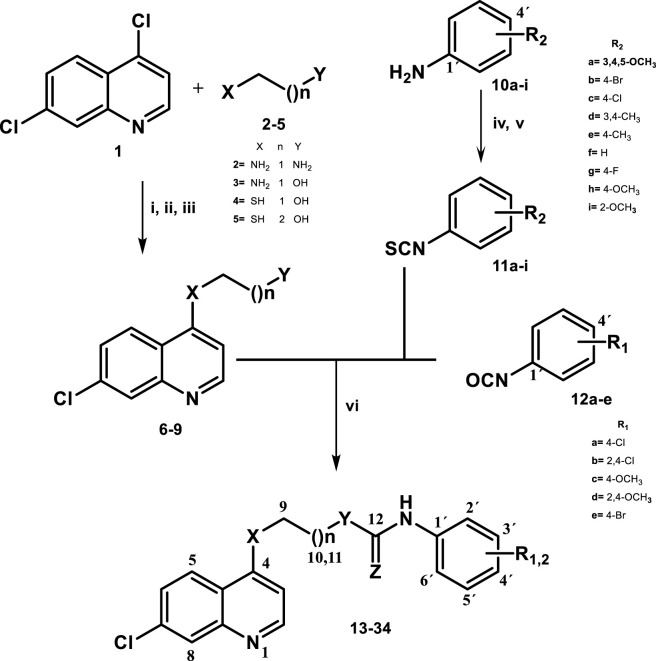
Reagents and conditions for the synthesis of (**6**–**9**, **11a**–**i**, and **13**–**34**): (i) 1,2-ethylenediamine **2** (10 equiv), 80 °C, 12 h; (ii) 2-aminoethanol **3**, Et_3_N, refluxing, 12 h; (iii) 2-mercaptoethan-1-ol **4**, and 3-mercaptopropan-1-ol **5**, DMAP, DMF, 80 °C, 12 h; (iv) CS_2_, NEt_3_, rt; (v) ClCO_2_Me, CHCl_3_, NEt_3_, rt; (vi) DMAP, DMF, 50 °C, 2 h.

Compounds **13**–**34** were prepared starting from 4,7-dichloroquinoline **1**, which was reacted as per Gutiérrez et al. (2023a) and Gutiérrez et al. (2023b) through an addition–elimination mechanism to yield *N,S*-(7-chloroquinolin)4-substituted derivatives **6**–**9**. The target compounds **13**–**34** were synthesized via coupling reactions of intermediates **6–9** with a series of substituted phenyl isothiocyanates **11a**–**i** and phenyl isocyanates **12a**–**e**. The reactions were carried out in DMF at 50 °C for 2 h using DMAP. Phenyl isothiocyanates **11a**–**i** were prepared in the laboratory as per Hodgkins and Preston Reeves (1964) and Segawa et al. (1992), while phenyl isocyanates **12a**–**e** were commercially available. The products were isolated in good-to-excellent yields (60%–89%) after purification by recrystallization (Scheme 1).

The chemical structures of the synthesized compounds were confirmed based on their infrared (IR) and nuclear magnetic resonance (NMR) spectra, and their purity was ascertained by elemental analysis. The FT-IR spectra of all synthetic compounds were measured in the range 4,000–400 cm^−1^. In all spectra, two strong absorption peaks with typical broader shape were found in the range 3,300–3,100 cm^−1^, corresponding to N-H bond stretching with extensive H-bonding environment. Sharp and intense peaks were noted, while broad peaks were observed for others. The aromatic and aliphatic C-H peaks were well resolved, while overlapping of aromatic C-H bonds with N-H was noticed. From 2,993 to 2,839 cm^−1^, sharp peaks were noticed which correspond to saturated C-H bonds. Similarly, in the region of 1,700–1,549 cm^−1^, stretching vibrations for the aromatic C=C and C=S bonds were observed. Additional stretching bands at approximately 1,243–1,200 cm^−1^ were assigned to bending vibrations of the sulfur-containing groups.

In the ^1^H NMR spectra, the signals of the respective protons of each compound were assigned based on their chemical shifts, multiplicities, and coupling constants. The aliphatic signals expected at upfield shifts for compounds **13**–**34** were present between 1.84 and 4.38 ppm, and were assigned to protons 9, 10, and 11. The quinoline moiety protons appeared as a doublet around 6.5 ppm (d, *J* = 5 Hz) assigned to proton H3, a double doublet around 7.3 ppm (dd, *J* = 8 and 2 Hz) corresponding to proton H6, a doublet around 7.5 ppm (d, *J* = 8 Hz) for the proton H5, a doublet around 7.9 ppm (d, *J* = 2 Hz) corresponding to proton H8, and a doublet around 8.5 ppm (d, *J* = 5 Hz) assigned to proton H2. The aromatic region of the ^1^H NMR spectra featured signal patterns of 6.5–8.0 ppm, which were characteristic of the substitution pattern of each aromatic ring. The structures of all target compounds were confirmed using ^13^C NMR, DEPT-135°, COSY, HSQC, and HMBC.

## Biology

### Antimalarial activity

All derivatives were tested *in vitro* for their ability to inhibit β-hematin formation and *in vivo* for their efficacy in a murine model (Table 1). The *in vitro* assay was used to assess the ability of derivatives **13**–**34** to inhibit β-hematin formation. To evaluate the potential antimalarial activity of these compounds, we tested their capacity to inhibit heme crystallization, considering that heme can spontaneously crystallize under the acidic and low-oxygen conditions found in the parasite’s vacuole (Baelmans et al., 2000; Dorn et al., 1995). Results showing more than 80% inhibition of heme crystallization were considered significant; compounds **13**, **14**, **16**–**18**, and **27**–**29** met this criterion (Table 1). These compounds were less active than chloroquine (92.55% ± 0.019%) in inhibiting heme crystallization.

**TABLE 1:** Inhibition of β-hematin formation (IβHF) and effects on *P. berghei*-infected mice (20 mg kg^−1^) of the 7-chloroquinoline derivatives 13–34.

N°	X	Y	Z	n	R	%IβHF(±SD)^a^	%P^b^(±SD)^d^	Sd^c^ (±SD)^d^	(%) Hemolysis
13	NH	NH	O	1	4-Cl	83.03 ± 0.043	2.45 ± 0.06†**	26.01 ± 0.07**	19.45 ± 0.16†
14	NH	NH	O	1	2,4-Cl	79.09 ± 0.029	3.53 ± 0.10†**	24.2 ± 0.13**	22.52 ± 0.11
15	NH	NH	O	1	4-OCH_3_	66.28 ± 0.037	nd	nd	nd
16	NH	NH	O	1	2,4-OCH_3_	90.68 ± 0.043	1.2 ± 0.12*	27.33 ± 0.15*	17.23 ± 0.13†
17	NH	O	O	1	4-Cl	86.26 ± 0.023	2.39 ± 0.15**	24.33 ± 0.10**	21.19 ± 0.11
18	NH	O	O	1	2,4-Cl	81.48 ± 0.018	1.30 ± 0.13*	25.10 ± 0.12**	18.15 ± 0. 24†
19	NH	O	O	1	4-Br	19.50 ± 0.023	nd	nd	nd
20	NH	O	O	1	2,4-OCH_3_	65.66 ± 0.008	nd	nd	nd
21	S	O	O	1	4-Cl	33.40 ± 0.026	nd	nd	nd
22	S	O	O	1	2,4-Cl	30.07 ± 0.013	nd	nd	nd
23	S	O	O	1	4-OCH_3_	24.35 ± 0.028	nd	nd	nd
24	S	O	O	2	4-Cl	67.63 ± 0.068	nd	nd	nd
25	S	O	O	2	2,4-Cl	74.61 ± 0.032	nd	nd	nd
26	NH	NH	S	1	3,4,5-OCH_3_	40.90 ± 0.088	nd	nd	nd
27	NH	NH	S	1	4-Br	86.89 ± 0.020	2.86 ± 0.12**	22.66 ± 0.16**	21.15 ± 0.20
28	NH	NH	S	1	4-Cl	87.80 ± 0.041	2.40 ± 0.02**	23.33 ± 0.12**	16.50 ± 0.22†
29	NH	NH	S	1	3,4-CH_3_	86.36 ± 0.011	3.65 ± 0.25**	14.66 ± 0.15*	37.16 ± 0.19†
30	NH	NH	S	1	4-CH_3_	71.96 ± 0.028	nd	nd	nd
31	NH	NH	S	1	H	59.39 ± 0.014	nd	nd	nd
32	NH	NH	S	1	4-F	50.00 ± 0.027	nd	nd	nd
33	NH	NH	S	1	4-OCH_3_	64.92 ± 0.096	nd	nd	nd
34	NH	NH	S	1	2-OCH_3_	31.13 ± 0.039	nd	nd	nd
CQ	--	--	--	--	--	92.55 ± 0.019	0.88 ± 0.09**	29.6 ± 0.13**	29.65 ± 0.10
CiSS	--	--	--	--	--	--	89.97 ± 0.13	8.6 ± 0.17	nd

^a^ Percentage inhibition of β-hematin formation (%IβHF) (n = 3).

^b^ %P: percentage of parasitemia.

^c^ Sd: survival days.

^d^ SD: standard deviation. CQ: chloroquine. CiSS: control infected and treated with saline solution (n = 4). nd: not determined. †p < 0.001 compared to chloroquine. *p < 0.01 and **p < 0.001, compared to control infected (CiSS). The data were analyzed using one-way ANOVA, followed by Dunnett’s multiple comparison test to determine significance compared to the infected control (CiSS) and Bonferroni’s test for comparing the derivatives against the positive control CQ.

The presence of urea and thiourea moieties appeared to be favorable when the phenyl ring was substituted with chlorine or bromine atoms at positions 4 or 2,4, with the exception of compounds **16** (2,4-OCH_3_) and **29** (3,4-CH_3_), which also showed excellent activity. Compounds **17** and **18**, carbamates with a 4-Cl, 2,4-Cl substitution, were the only active members of this compound family. The remaining compounds displayed moderate to weak activity against β-hematin formation. Another variable to take into account would be the length of the connector; according to the results, two carbon atoms are ideal, along with the presence of a greater number of nitrogen atoms.
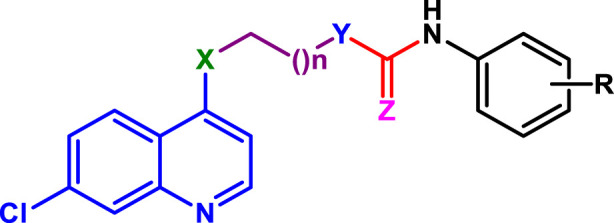


Compounds that showed inhibition of heme crystallization equal to or greater than 80% were evaluated *in vivo* in mice infected with *P. berghei* ANKA, a CQ-susceptible strain of murine malaria. The antimalarial potential of these compounds was determined by the ability of compounds **13**, **14**, **16**–**18**, and **27**–**29** to increase mouse survival and reduce parasitemia *in vivo*, as assessed on the fourth day post-infection compared to the untreated control group. Mice were treated i. p. once daily with compounds **13**, **14**, **16**, **17**–**18**, and **27**–**29** (20 mg kg^−1^) or CQ (20 mg kg^−1^) following previously reported protocols (Charris et al., 2019; Dorn et al., 1995; Kerns and Di, 2008; Peters and Robinson, 1999).

The Institute of Immunology Bioethical Committee approved the study according to universal guidelines of the National Research Council’s Institute for Laboratory Animal Research (ILAR) and the ethical principles for medical research by the World Medical Association Declaration of Helsinki. All animals were kept in ventilated cages with corncob bedding, under standard conditions (22 °C ± 3 °C, 40%–70% relative humidity, 12 h/12 h 6 AM–6 PM light/dark cycle) with food and water *ad libitum*.

According to the results in Table 1, the evaluated derivatives were able to increase the survival of infected mice and reduce parasitemia. Control mice died at 8.6 ± 0.17 days. Compounds **13**, **14**, **17**, and **27**–**29**, as monotherapy, prolonged the average survival time of infected mice to 14.66 ± 0.15 to 26.01 ± 0.07 days but were not able to reduce or delay the evolution of malaria (3.65% ± 0.25% and 2.39% ± 0.15%). Compounds **16** and **18** prolonged the average survival time of infected mice to between 27.33 ± 0.15 and 25.10 ± 0.12 days, respectively, and showed a reduction in the evolution of malaria (1.2% ± 0.12% and 1.30% ± 0.13%, respectively). CQ prolonged mouse survival time to 29.6 ± 0.13 days and decreased the development of malaria to 0.88% ± 0.09%.

The hemolytic response of compounds **13**, **14**, **16**–**18**, and **27**–**29** was further determined (Crupi et al., 2019; Mehta et al., 1984). Hemolysis was less than 40% in mouse red blood cells at a concentration of 10 mM, showing that these compounds do not have a marked lytic action on the RBCs of mice (Table 1).

The results of ALT, AST and GGT activities are presented in Table 2. In normal mice, the ALT activity was 101.06 ± 0.43 IU/L, the AST activity was 51.40 ± 0.22 IU/L, and the GGT level was 80.65 ± 0.46 IU/L. In the infected mice, ALT, AST, and GGT activities were significantly elevated compared to the control mice (*p* < 0.01). The study revealed significant increase in *P. berghei* infection in the enzymatic activity level of these biomarkers in mice compared to the control group (*p* < 0.01). This result agrees with George et al. (2011), who found that *P. berghei* malaria infection significantly increased the activity level of ALT, AST, and GGT. Compounds **13**, **16**, **27**, and **28** demonstrated the most favorable liver enzyme profiles in *P. berghei*-infected mice, suggesting potential hepatoprotective effects. Compounds **14** and **29** showed moderate elevations in liver enzymes. Chloroquine remains a reference standard, with moderate but acceptable liver enzyme elevations. In contrast, **17** and **18** were associated with marked hepatotoxicity. These findings provide a foundation for selecting and optimizing antimalarial therapies with minimal hepatic side effects and highlight the importance of comprehensive liver function monitoring in preclinical drug evaluation.

**TABLE 2:** Results of liver function parameters of *P. berghei*-infected mice, the control group, and those treated with compounds (20 mg kg^−1^).

No	ALT (IU/L) ± S.D	AST(IU/L) ± S.D	GGT (IU/L)± S.D
13	148.56 ± 0.13	69.98 ± 0.26	91.65 ± 0.76
14	162.74 ± 0.22	81.23 ± 0.62	102.65 ± 0.16
16	135.56 ± 0.21	62.50 ± 0.25	89.65 ± 0.35
17	203.45 ± 0.33	113.26 ± 0.24	121.5 ± 0.77
18	200.47 ± 0.56	110.23 ± 0.57	118.54 ± 0.76
27	150.43 ± 0.52	62.54 ± 0.14	90.15 ± 0.25
28	148.75 ± 0.26	61.30 ± 0.18	90.68 ± 0.16
29	152.06 ± 0.73	69.30 ± 0.12	91.23 ± 0.14
CQ	171.02 ± 0.25	71.56 ± 1.22	90.64 ± 0.54
UC	101.06 ± 0.43	51.40 ± 0.22	80.65 ± 0.46
CiSS	205.75 ± 0.88	115.23 ± 0.20	122.15 ± 1.12

CQ, chloroquine; UC, uninfected control; CiSS, control infected and treated with saline solution (n = 4); ALT, alanine aminotransferase; AST, aspartate aminotransferase; GGT, gamma-glutamyl transferase (GGT); IU/L, International Units/liter; S. D, standard deviation.

#### Molecular docking

The detailed mechanism by which β-hematin inhibitors obstruct crystal growth is still unknown. It has been proposed that the mechanism of quinoline inhibition is by binding to hematin (Dorn et al., 1998; Egan, 2006; Egan et al., 1994; Kapishnikov et al., 2021). Two options are possible: a) binding to the fastest emergent faces of the β-hematin crystal (Buller et al., 2002; Egan et al., 2001; Pagola et al., 2000) or b) through generation of a drug–heme complex covering the hemozoin crystal to impede further crystal growth (Sullivan et al., 1996). Both pathways involve the binding of the compound to Fe(III)PPIX. De Villiers et al. (2012) described crystal complexes of quinidine-heme (QD-Fe(III) PPIX) and quinine-heme (QN-Fe(III)PPIX), revealing that three critical interactions are implicated in binding: coordination, hydrogen bonding, and π–π stacking. In quinine-heme and quinidine-heme structures, there is a hydrogen bond between the propionate group of Fe(III)PPIX and the protonated quinuclidine nitrogen. In addition, Kelly et al., (2001), using the ^1^H NMR technique, reported that the 4,5-dihydroxyxantone–heme complex is stabilized through hydrogen bonding between the hydroxyl groups and the propionate side chains of the heme, as well as π–π stacking between both aromatic systems. Although there is no clear information about the requirements for π–π stacking, it was proposed that large planar aromatic molecular surfaces may favor binding with either hematin or hemozoin.

The molecular docking results show that the 7-chloroquinoline derivatives (**13–34**) exhibit binding energies ranging from −8.3 to −11.4 kcal/mol, indicating significant affinity in most cases toward the β-hematin surface. Compound **13** showed the highest binding energy (−11.4 kcal/mol), followed by **17** and **19**. However, a lack of linear correlation is observed for compound **19**, which displays favorable binding energy but a notably low percentage inhibition of βHF (19.50%); this could be attributed to the pharmacokinetics and bioavailability factors. On the other hand, compounds such as **16**, **27**, **28**, and **29** with lower binding energies show exceptional activity, possibly due to improved pharmacokinetic properties (Table 3).

**TABLE 3:** Molecular docking results of 7-chloroquinoline derivatives against β-hematin.

Compound	Binding energy kcal/mol (β-hematin)	Compound	Binding energy kcal/mol (β-hematin)
13	−11.4	25	−10.6
14	−10.8	26	−8.3
15	−10.5	27	−9.8
16	−10.6	28	−10.0
17	−11.1	29	−10.9
18	−10.5	30	−10.7
19	−11.2	31	−9.9
20	−10.4	32	−10.1
21	−10.9	33	−10.1
22	−10.4	34	−9.9
23	−10.4	CQ	−8.9
24	−10.7	​	​

**FIGURE 3:**
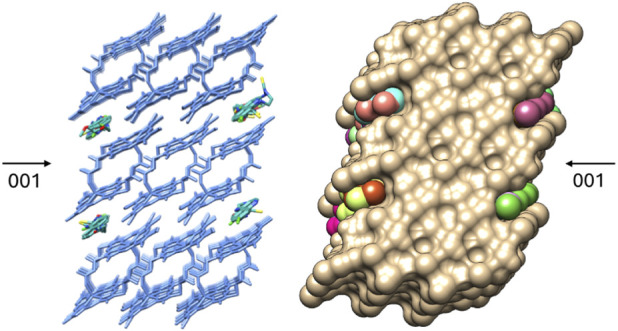
3D and surface view of the structure of the β-hematin crystal with the compounds.

Conformational analysis of the docking poses reveals that the compounds preferentially orient within the grooves of the {001} faces of β-hematin, with the quinoline ring intercalated parallel to the porphyrin plane, favoring co-facial π–π stacking interactions (Figure 3). These interactions are considered the main stabilizing force in hemozoin binding, as reported by Buller et al. (2002) for chloroquine analogs.

**FIGURE 4:**
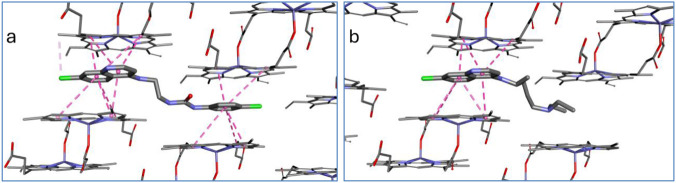
Compounds docked at (001) face of β-hematin. **(a)** Best docking of synthesized compound (**13**). **(b)** Best docking of CQ.

Additionally, the flexible side chain of the derivatives, containing functional groups such as urea, carbamate, or thiourea, along with an aromatic residue, fits into adjacent hydrophobic grooves, establishing co-facial π–π interactions and Van der Waals forces. This results in the formation of complexes with higher binding affinity than CQ (Figure 4).

The presence of halogen substituents (Cl and Br) or methoxy groups (OCH_3_) on the terminal aromatic ring appears to modulate steric and electronic complementarity, influencing both binding affinity and final biological activity.

## Conclusion

Malaria is a devastating disease that poses a significant public health risk in various regions of the world. It is a serious illness that affects both humans and animals and is caused by protozoan parasites of the genus *Plasmodium*. Despite the effectiveness of treatments such as chloroquine and artemisinin-based combination therapies and other antimalarial drugs, the emergence of resistant parasite strains presents a major challenge to current malaria treatment strategies. This study has demonstrated a strong correlation between the inhibition of βHF, parasitemia reduction, low hemolytic activity, hepatoprotective effects, and increased survival rates in infected mice. A possible mechanism for the action of 4-substituted-7-chloroquinoline agents involves their inhibition of β-hematin formation *in vivo*. These compounds may exert their effects through π–π stacking interactions between the porphyrin ring of the hematin structure and the quinoline ring, as has been reported for CQ and other aminoquinolines. This interaction likely facilitates the active accumulation of the heme-compound complex within erythrocytes, leading to parasite death via oxidative stress. Although these derivatives were effective as inhibitors of βHF, they were unable to extend the survival rate of infected mice beyond 27 days. This limited survival may be attributed to the pharmacokinetics and bioavailability of these compounds *in vivo*. Further experiments are necessary to fully elucidate the mechanism of action and optimize the therapeutic potential of these agents.

## Data Availability

The original contributions presented in the study are included in the article/Supplementary Material; further inquiries can be directed to the corresponding authors.

## In memoriam

We dedicate this article in memory of the late Professor Arthur R. Barazarte (Libertador Experimental Pedagogical University, Venezuela). Professor Barazarte completed his doctoral studies in Medicinal Chemistry at the Central University of Venezuela’s Faculty of Pharmacy. His work was directed by a clear mission: to foster collaboration in training the next-generation of researchers to develop new molecules with potential chemotherapeutic activity.
