# Supplementary material for: Design and synthesis of 7-chloroquinoline-derivative-bearing urea, carbamate, or thiourea moieties and their antimalarial activity against Plasmodium berghei via the inhibition of β-hematin formation
Source: Front Chem. 2026 May 29;14:1827662. doi: 10.3389/fchem.2026.1827662 (PMC13261810; doi:10.3389/fchem.2026.1827662)
Supplement: Supplementary file 1 [file DataSheet1.pdf]

Supplementary material

Design, synthesis of 7-chloroquinoline derivatives bearing urea, carbamate, or thiourea moieties and their antimalarial activity against *Plasmodium berghei* via inhibition of β hematin formation

Hegira Ramírez, Ali S. Mijoba, Sandra Espinosa, Arthur R. Barazarte, María E. Acosta, Jaime E. Charris, Esteban Fernandez-Moreira

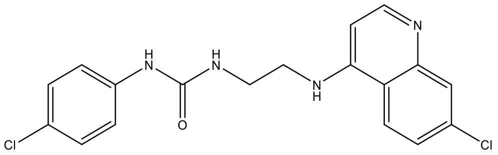

13

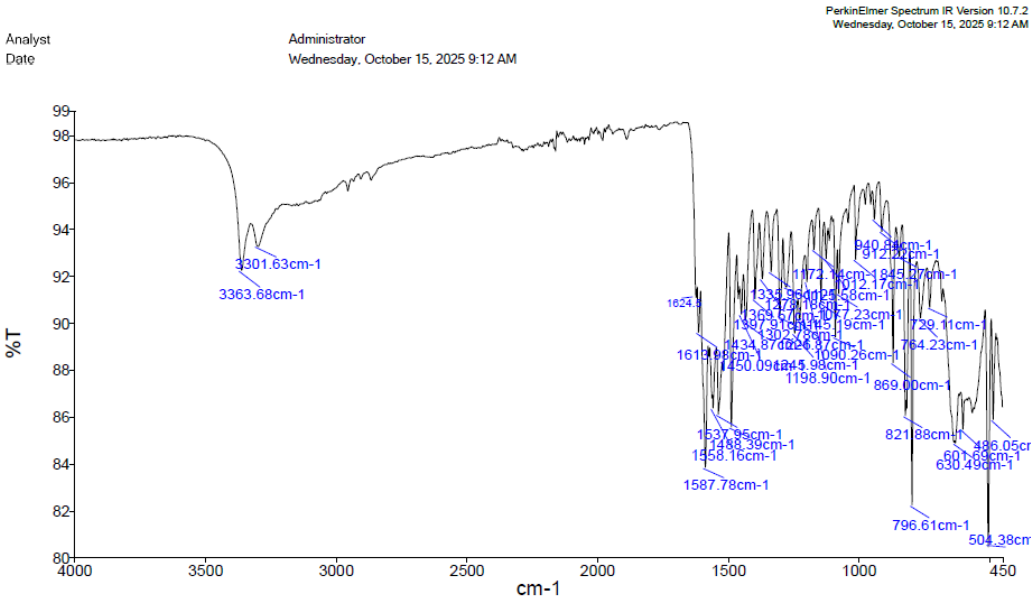

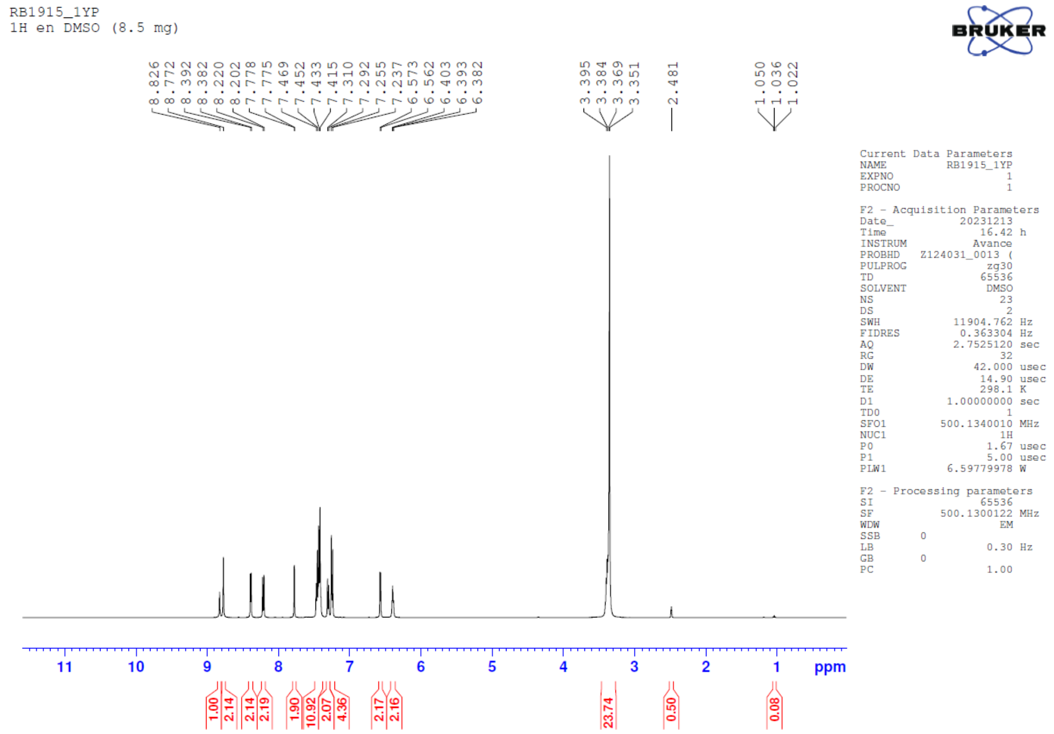
RB1915_1YP
1H en DMSO (8.5 mg)
BRUKER
8.826 8.772 8.392 8.382 8.220 8.202 7.778 7.775 7.469 7.452 7.433 7.415 7.310 7.292 7.255 7.237 6.573 6.562 6.403 6.393 6.382
3.395 3.384 3.369 3.351
2.481
1.050 1.036 1.022
11 10 9 8 7 6 5 4 3 2 1 ppm
1.00 2.14 2.14 2.19 1.90 10.92 2.07 4.36 2.17 2.16 23.74 0.50 0.08
Current Data Parameters
NAME RB1915_1YP
EXPNO 1
PROCNO 1
F2 - Acquisition Parameters
Date_ 20231213
Time 16.42 h
INSTRUM Avance
PROBHD Z124031_0013 (
PULPROG zg30
TD 65536
SOLVENT DMSO
NS 23
DS 2
SWH 11904.762 Hz
FIDRES 0.363304 Hz
AQ 2.7525120 sec
RG 32
DW 42.000 usec
DE 14.90 usec
TE 298.1 K
D1 1.00000000 sec
TD0 1
SFO1 500.1340010 MHz
NUC1 1H
P0 1.67 usec
P1 5.00 usec
PLW1 6.59779978 W
F2 - Processing parameters
SI 65536
SF 500.1300122 MHz
WDW EM
SSB 0
LB 0.30 Hz
GB 0
PC 1.00

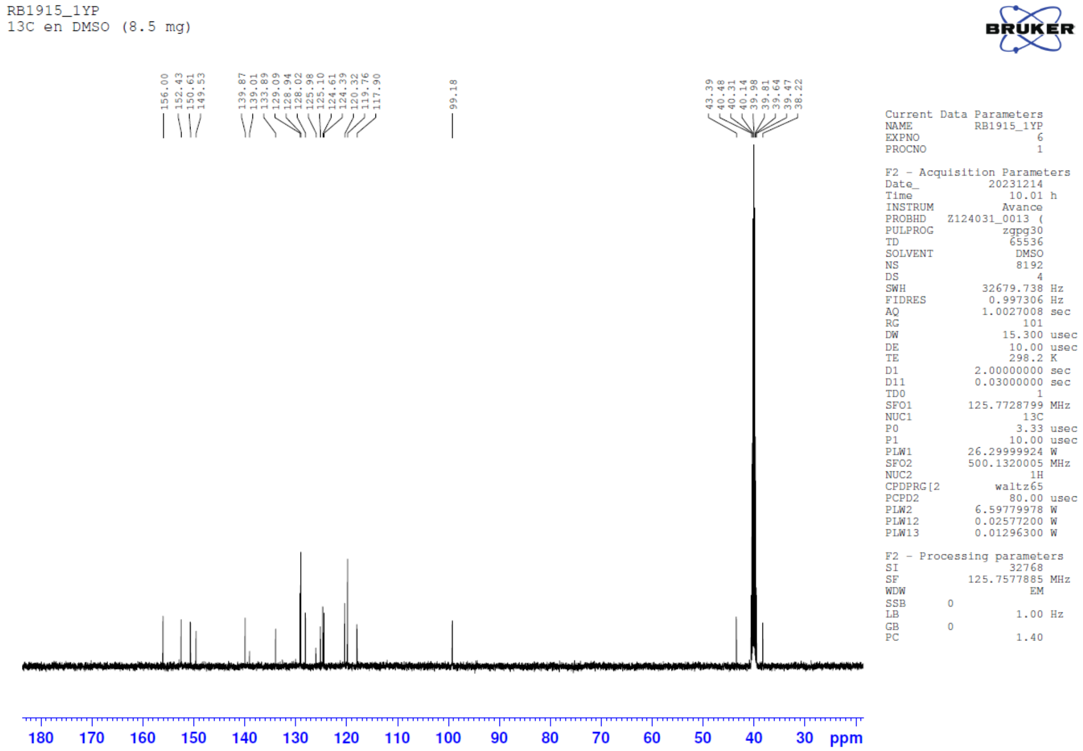
RB1915_1YP
13C en DMSO (8.5 mg)
BRUKER
156.00 152.43 150.61 149.53
139.87 139.01 133.89 129.09 128.94 128.02 125.98 125.10 124.61 124.39 120.32 119.76 117.90
99.18
43.39 40.48 40.31 40.14 39.98 39.81 39.64 39.47 38.22
180 170 160 150 140 130 120 110 100 90 80 70 60 50 40 30 ppm
Current Data Parameters
NAME RB1915_1YP
EXPNO 6
PROCNO 1
F2 - Acquisition Parameters
Date_ 20231214
Time 10.01 h
INSTRUM Avance
PROBHD Z124031_0013 (
PULPROG zgpg30
TD 65536
SOLVENT DMSO
NS 8192
DS 4
SWH 32679.738 Hz
FIDRES 0.997306 Hz
AQ 1.0027008 sec
RG 101
DW 15.300 usec
DE 10.00 usec
TE 298.2 K
D1 2.00000000 sec
D11 0.03000000 sec
TD0 1
SFO1 125.7728799 MHz
NUC1 13C
P0 3.33 usec
P1 10.00 usec
PLW1 26.29999924 W
SFO2 500.1320005 MHz
NUC2 1H
CPDPRG[2 waltz65
PCPD2 80.00 usec
PLW2 6.59779978 W
PLW12 0.02577200 W
PLW13 0.01296300 W
F2 - Processing parameters
SI 32768
SF 125.7577885 MHz
WDW EM
SSB 0
LB 1.00 Hz
GB 0
PC 1.40

RB1915_1YP
DEPT en DMSO (8.5 mg)

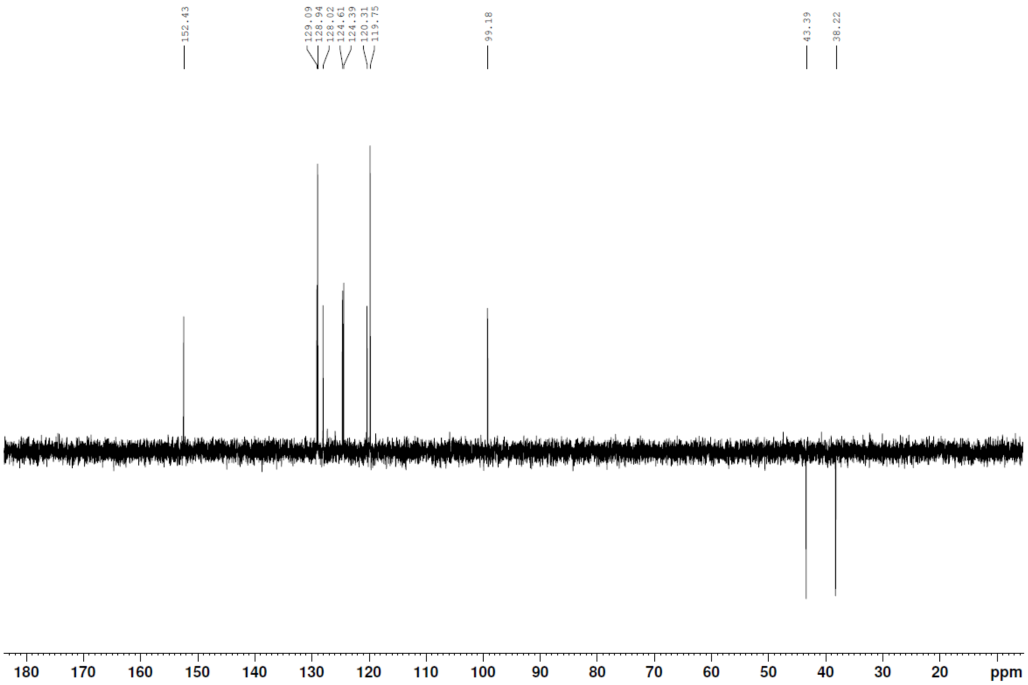

RB1915_1YP
COSY en DMSO (8.5 mg)

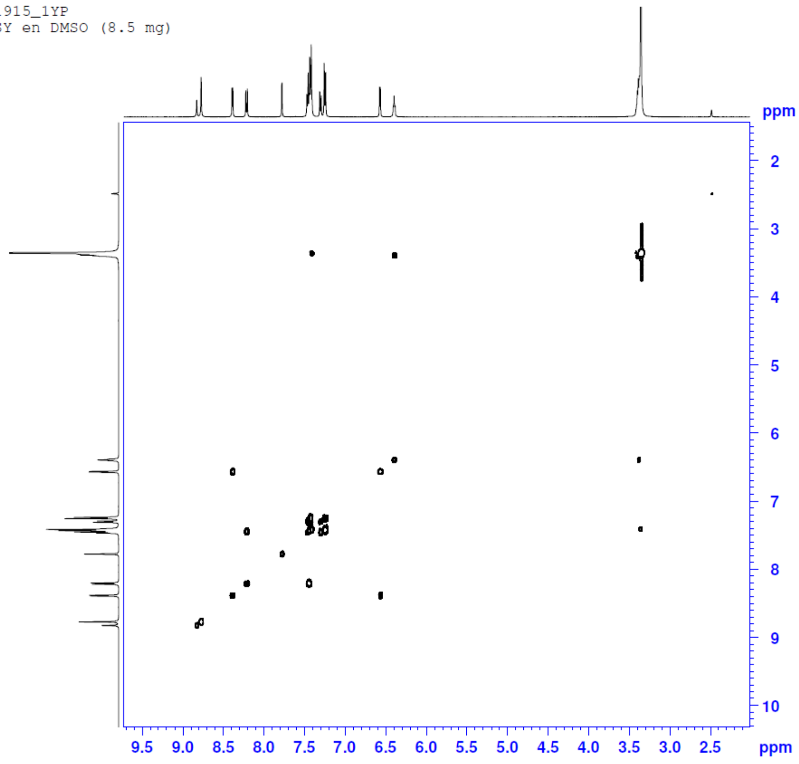

Current Data Parameters	
NAME	RB1915_1YP
EXPNO	3
PROCNO	1

F2 - Acquisition Parameters	
Date_	20231213
Time	18.21 h
INSTRUM	Avance
PROBHD	Z124031_0013 (
PULPROG	cosygpppqf
TD	2048
SOLVENT	DMSO
NS	4
DS	16
SWH	7142.857 Hz
FIDRES	6.975446 Hz
AQ	0.1433600 sec
RG	64
DW	70.000 usec
DE	10.00 usec
TE	298.2 K
D0	0.00000300 sec
D1	2.00000000 sec
D11	0.03000000 sec
D12	0.00002000 sec
D13	0.00000400 sec
D16	0.00020000 sec
IN0	0.00014000 sec
TDav	1
SFO1	500.1330787 MHz
NUC1	1H
P0	5.00 usec
P1	5.00 usec
P17	2500.00 usec
PLW1	6.59779978 W
PLW10	0.18327001 W
GPNAM[1]	SMSQ10.100
GPZ1	10.00 %
P16	1000.00 usec

F1 - Acquisition parameters	
TD	256
SFO1	500.1331 MHz
FIDRES	55.803570 Hz
SW	14.282 ppm
FnMODE	QF

F2 - Processing parameters	
SI	2048
SF	500.1300122 MHz
WDW	QSINE
SSB	0
LB	0 Hz
GB	0
PC	1.40

F1 - Processing parameters	
SI	2048
MC2	QF
SF	500.1300122 MHz
WDW	QSINE
SSB	0
LB	0 Hz
GB	0

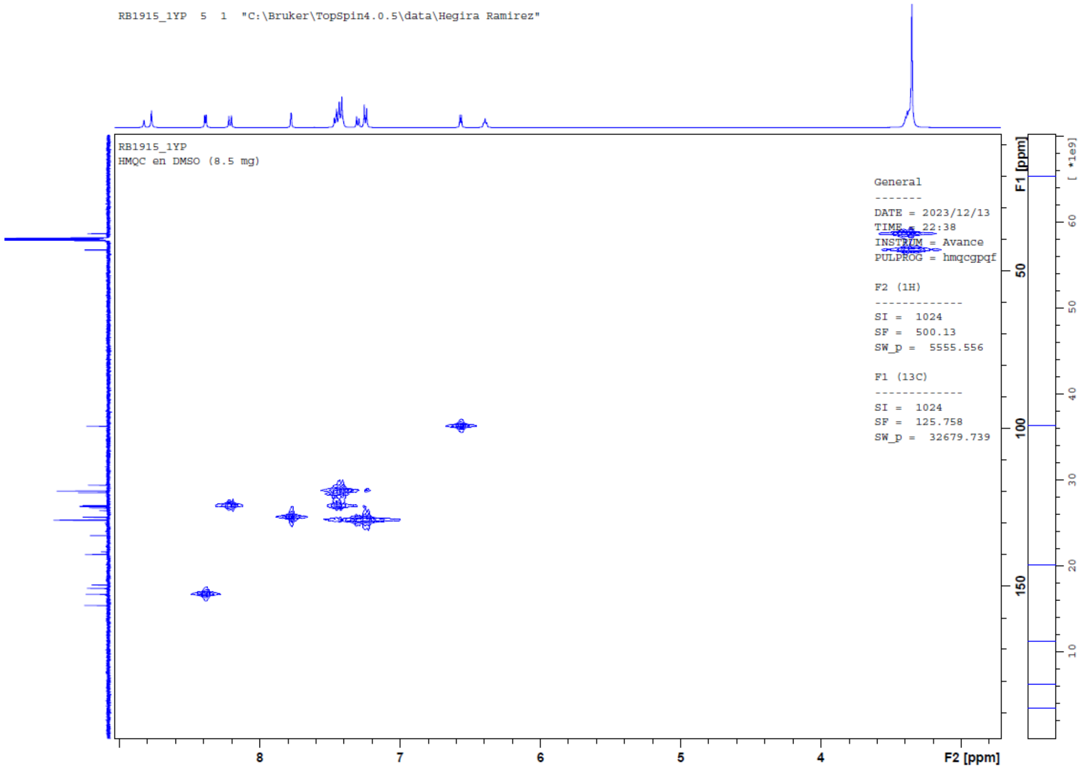

RB1915_1YP 5 1 "C:\Bruker\TopSpin4.0.5\data\Hegira Ramirez"
RB1915_1YP
HMQC en DMSO (8.5 mg)
General

DATE = 2023/12/13
TIME = 22:38
INSTRUM = Avance
PULPROG = hmqcgpqf
F2 (1H)

SI = 1024
SF = 500.13
SW_p = 5555.556
F1 (13C)

SI = 1024
SF = 125.758
SW_p = 32679.739
F1 [ppm]
F2 [ppm]
[*1e9]

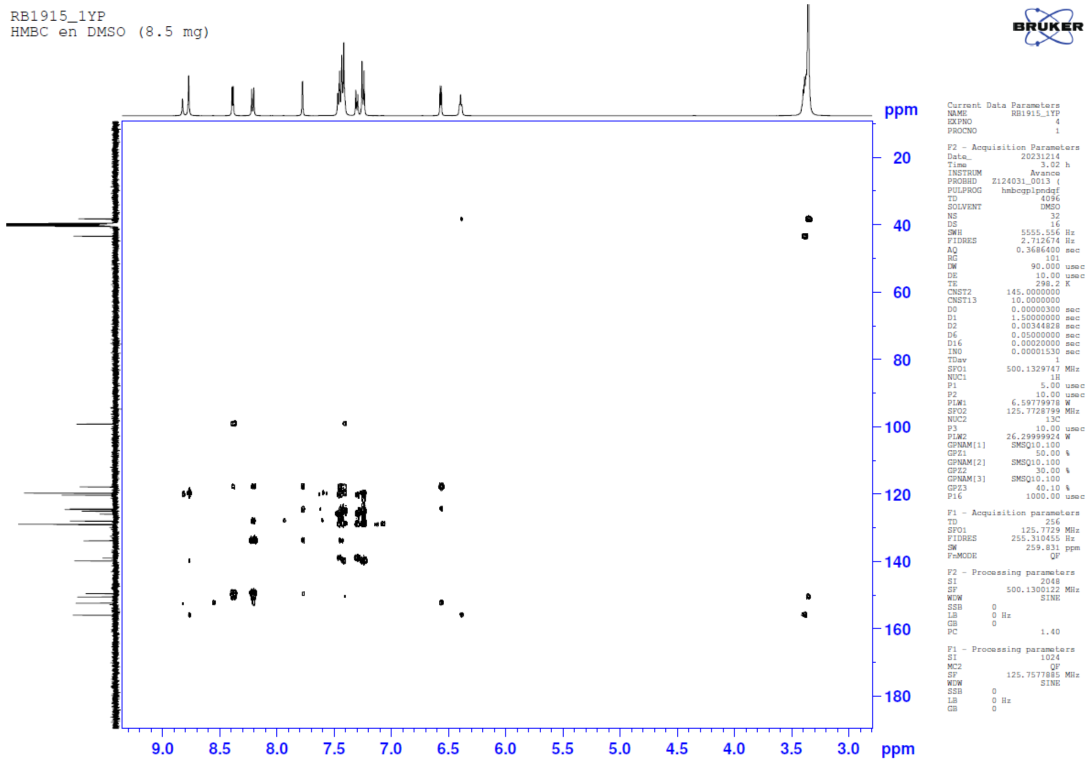

RB1915_1YP
HMBC en DMSO (8.5 mg)
ppm
Current Data Parameters
NAME RB1915_1YP
EXPNO 4
PROCNO 1
F2 - Acquisition Parameters
Date_ 20231214
Time 3.02 h
INSTRUM Avance
PROBHD Z124031_0013 (
PULPROG hmbcgplpndqf
TD 4096
SOLVENT DMSO
NS 32
DS 16
SWH 5555.556 Hz
FIDRES 2.712674 Hz
AQ 0.3686400 sec
RG 101
DW 90.000 usec
DE 10.00 usec
TE 298.2 K
CNST2 145.0000000
CNST13 10.0000000
D0 0.00000300 sec
D1 1.50000000 sec
D2 0.00344828 sec
D6 0.05000000 sec
D16 0.00020000 sec
IN0 0.00001530 sec
TDav 1
SFO1 500.1329747 MHz
NUC1 1H
P1 5.00 usec
P2 10.00 usec
PLW1 6.59779978 W
SFO2 125.7728799 MHz
NUC2 13C
P3 10.00 usec
PLW2 26.29999924 W
GPNAM[1] SMSQ10.100
GPZ1 50.00 %
GPNAM[2] SMSQ10.100
GPZ2 30.00 %
GPNAM[3] SMSQ10.100
GPZ3 40.10 %
P16 1000.00 usec
F1 - Acquisition parameters
TD 256
SFO1 125.7729 MHz
FIDRES 255.310455 Hz
SW 259.831 ppm
FnMODE QF
F2 - Processing parameters
SI 2048
SF 500.1300122 MHz
WDW SINE
SSB 0
LB 0 Hz
GB 0
PC 1.40
F1 - Processing parameters
SI 1024
MC2 QF
SF 125.7577885 MHz
WDW SINE
SSB 0
LB 0 Hz
GB 0

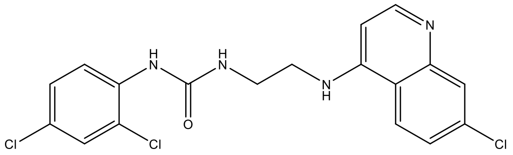

14

PerkinElmer Spectrum IR Version 10.7.2
Wednesday, October 15, 2025 9:15 AM

Analyst Administrator
Date Wednesday, October 15, 2025 9:15 AM

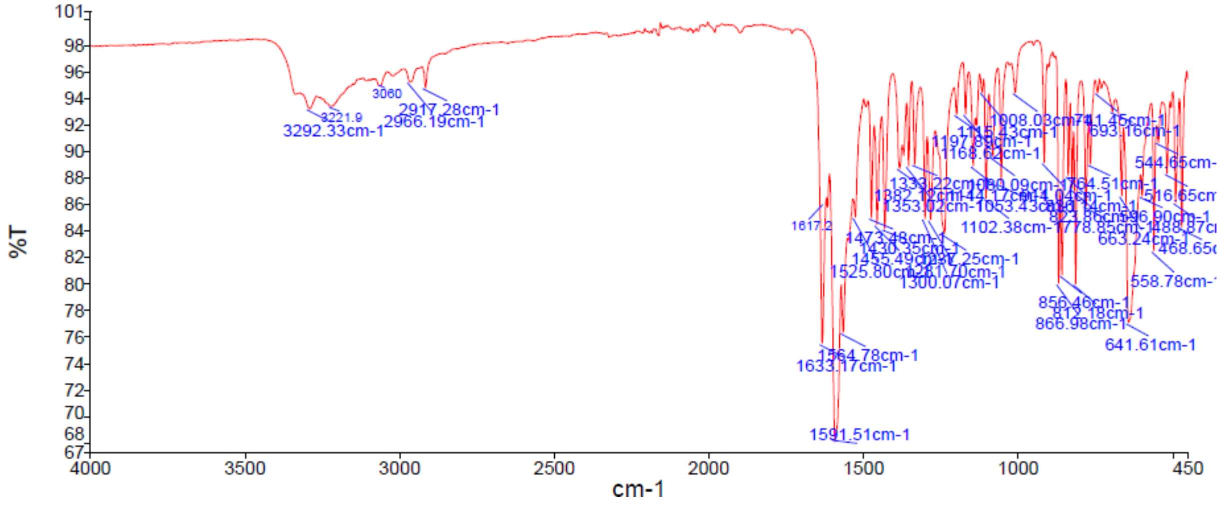

RB1916_2YP
1H en DMSO (8.4 mg)

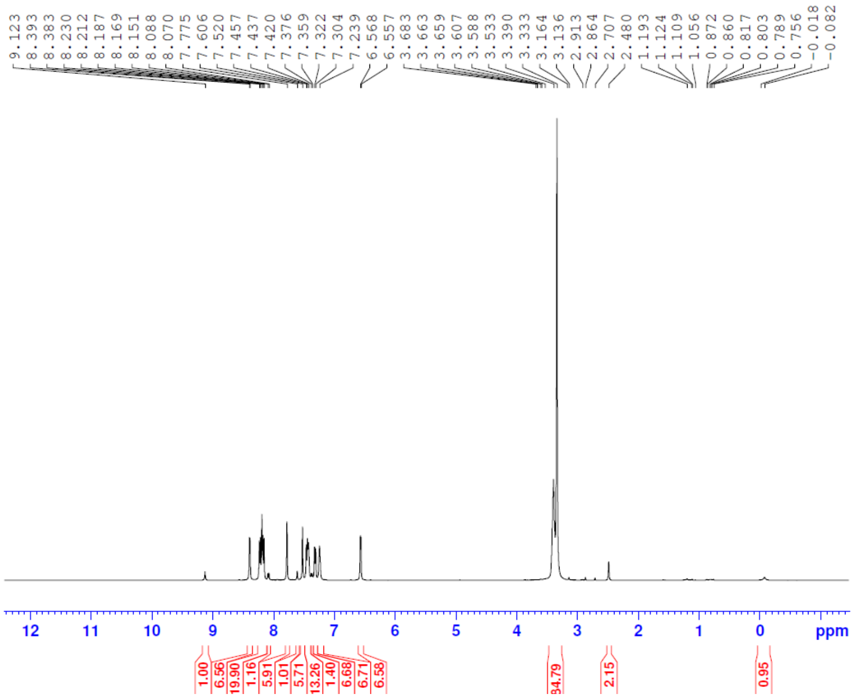

Current Data Parameters
NAME RB1916_2YP
EXPNO 1
PROCNO 1

F2 - Acquisition Parameters
Date_ 20231214
Time 15.37 h
INSTRUM Avance
PROBHD Z124031_0013 (
PULPROG zg30
TD 65536
SOLVENT DMSO
NS 123
DS 2
SWH 11904.762 Hz
FIDRES 0.363304 Hz
AQ 2.7525120 sec
RG 32
DW 42.000 usec
DE 14.90 usec
TE 298.2 K
D1 1.00000000 sec
TD0 1
SFO1 500.1340010 MHz
NUC1 1H
P0 1.67 usec
P1 5.00 usec
PLW1 6.59779978 W

F2 - Processing parameters
SI 65536
SF 500.1300122 MHz
WDW EM
SSB 0
LB 0.30 Hz
GB 0
PC 1.00

RB1916_2YP
13C en DMSO (8.4 mg)

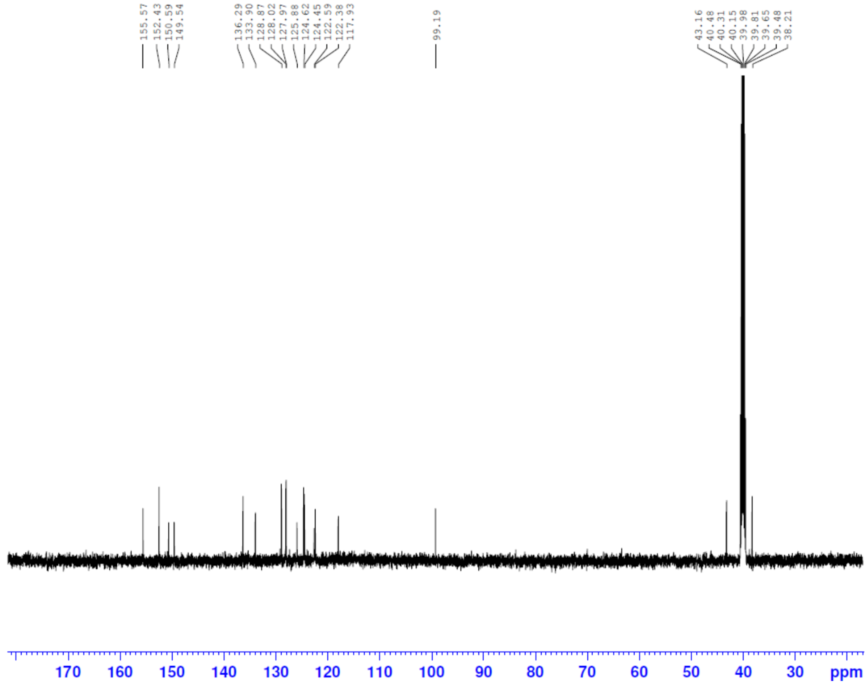

Current Data Parameters
NAME RB1916_2YP
EXPNO 3
PROCNO 1

F2 - Acquisition Parameters
Date_ 20231215
Time 14.03 h
INSTRUM Avance
PROBHD Z124031_0013 (
PULPROG zgpg30
TD 65536
SOLVENT DMSO
NS 8192
DS 4
SWH 32679.738 Hz
FIDRES 0.997306 Hz
AQ 1.0027008 sec
RG 101
DW 15.300 usec
DE 10.00 usec
TE 298.2 K
D1 2.00000000 sec
D11 0.03000000 sec
TD0 1
SFO1 125.7728799 MHz
NUC1 13C
P0 3.33 usec
P1 10.00 usec
PLW1 26.29999924 W
SFO2 500.1320005 MHz
NUC2 1H
CPDPRG[2 waltz65
PCPD2 80.00 usec
PLW2 6.59779978 W
PLW12 0.02577200 W
PLW13 0.01296300 W

F2 - Processing parameters
SI 32768
SF 125.7577885 MHz
WDW EM
SSB 0
LB 1.00 Hz
GB 0
PC 1.40

RB1916_2YP
DEPT en DMSO (8.4 mg)

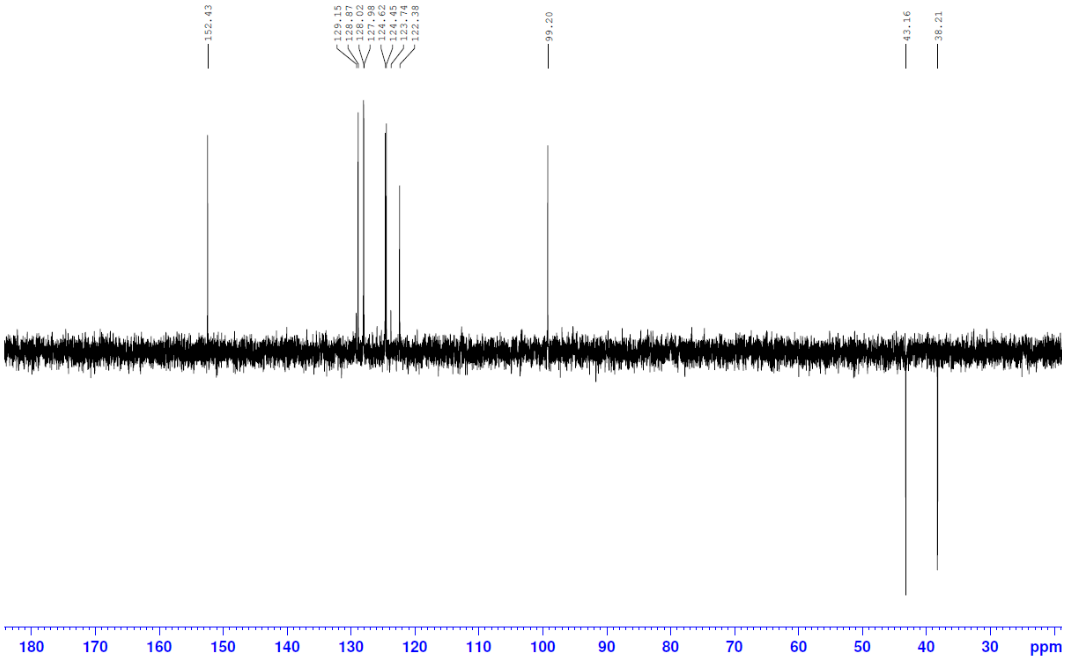

RB1916_2YP
COSY en DMSO (8.4 mg)

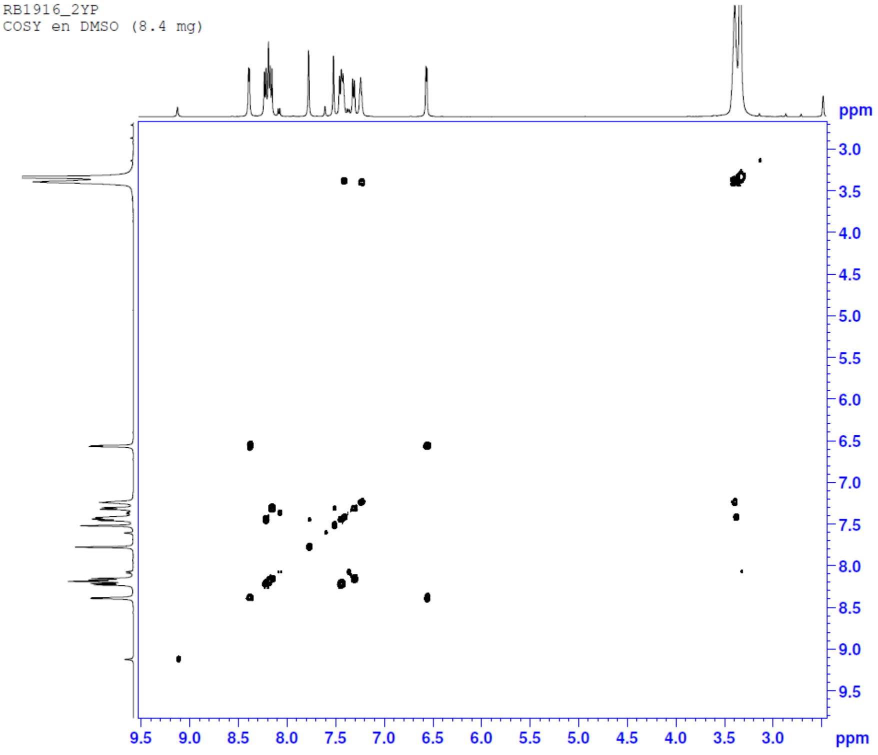


```
Current Data Parameters
NAME          RB1916_2YP
EXPNO                  4
PROCNO                 1

F2 - Acquisition Parameters
Date_           20231214
Time               20.27 h
INSTRUM           Avance
PROBHD   Z124031_0013 (
PULPROG       cosygpppqf
TD                  2048
SOLVENT             DMSO
NS                     8
DS                    16
SWH             6250.000 Hz
FIDRES          6.103516 Hz
AQ             0.1638400 sec
RG                    64
DW                80.000 usec
DE                 10.00 usec
TE                 298.2 K
D0            0.00000300 sec
D1            2.00000000 sec
D11           0.03000000 sec
D12           0.00002000 sec
D13           0.00000400 sec
D16           0.00020000 sec
IN0           0.00016000 sec
TDav                   1
SFO1         500.1328637 MHz
NUC1                  1H
P0                  5.00 usec
P1                  5.00 usec
P17              2500.00 usec
PLW1          6.59779978 W
PLW10         0.18327001 W
GPNAM[1]      SMSQ10.100
GPZ1               10.00 %
P16              1000.00 usec

F1 - Acquisition parameters
TD                   256
SFO1            500.1329 MHz
FIDRES         48.828125 Hz
SW                12.497 ppm
FnMODE                QF

F2 - Processing parameters
SI                  2048
SF           500.1300122 MHz
WDW                QSINE
SSB        0
LB         0 Hz
GB         0
PC                  1.40

F1 - Processing parameters
SI                  2048
MC2                   QF
SF           500.1300122 MHz
WDW                QSINE
SSB        0
LB         0 Hz
GB         0
```

RB1916_2YP
HMQC en DMSO (8.4 mg)

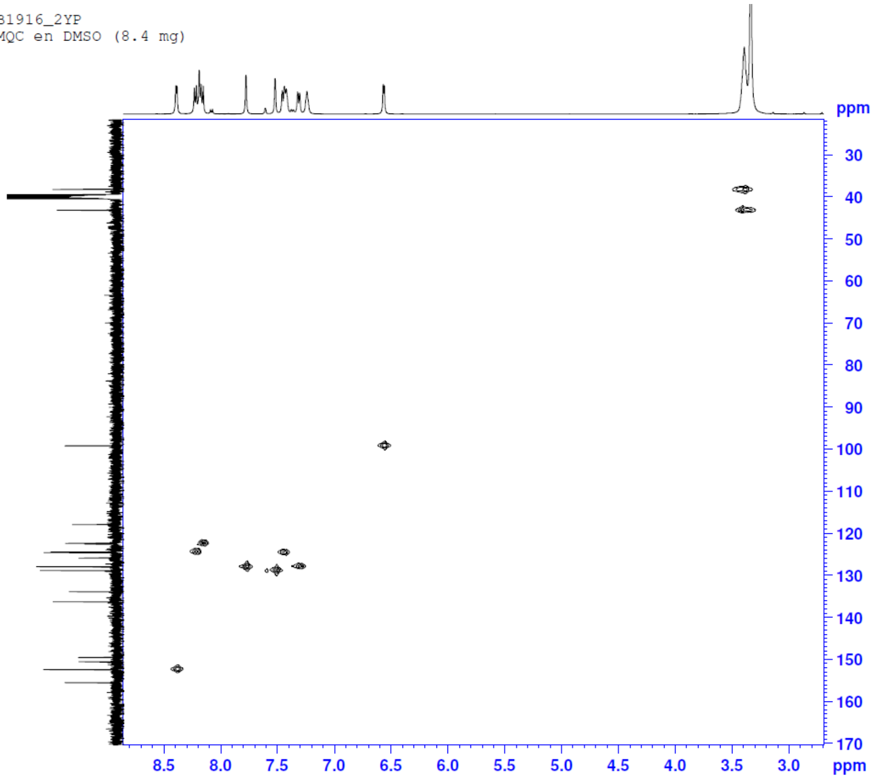


```
Current Data Parameters
NAME          RB1916_2YP
EXPNO                  5
PROCNO                 1

F2 - Acquisition Parameters
Date_           20231215
Time              1.41 h
INSTRUM           Avance
PROBHD    Z124031_0013 (
PULPROG         hmqcgpqf
TD                  4096
SOLVENT             DMSO
NS                    40
DS                    16
SWH           6250.000 Hz
FIDRES        3.051758 Hz
AQ           0.3276800 sec
RG                   101
DW              80.000 usec
DE               10.00 usec
TE               298.2 K
CNST2      145.0000000
D0          0.00000300 sec
D1          1.50000000 sec
D2          0.00344828 sec
D12         0.00002000 sec
D13         0.00000400 sec
D16         0.00020000 sec
IN0         0.00001530 sec
TDav                   1
SFO1       500.1328637 MHz
NUC1                  1H
P1                  5.00 usec
P2                 10.00 usec
PLW1        6.59779978 W
SFO2       125.7728799 MHz
NUC2                 13C
CPDPRG[2            garp
P3                 10.00 usec
PCPD2              70.00 usec
PLW2       26.29999924 W
PLW12       0.53672999 W
GPNAM[1]     SMSQ10.100
GPZ1               50.00 %
GPNAM[2]     SMSQ10.100
GPZ2               30.00 %
GPNAM[3]     SMSQ10.100
GPZ3               40.10 %
P16              1000.00 usec

F1 - Acquisition parameters
TD                   256
SFO1         125.7729 MHz
FIDRES     255.310455 Hz
SW            259.831 ppm
FnMODE                QF

F2 - Processing parameters
SI                  1024
SF         500.1300122 MHz
WDW                QSINE
SSB                    2
LB         0 Hz
GB         0
PC                  1.40

F1 - Processing parameters
SI                  1024
MC2                   QF
SF         125.7577885 MHz
WDW                QSINE
SSB                    2
LB         0 Hz
GB         0
```

RB1916_2YP
HMBC en DMSO (8.4 mg)

Print this file (Ctrl+P)

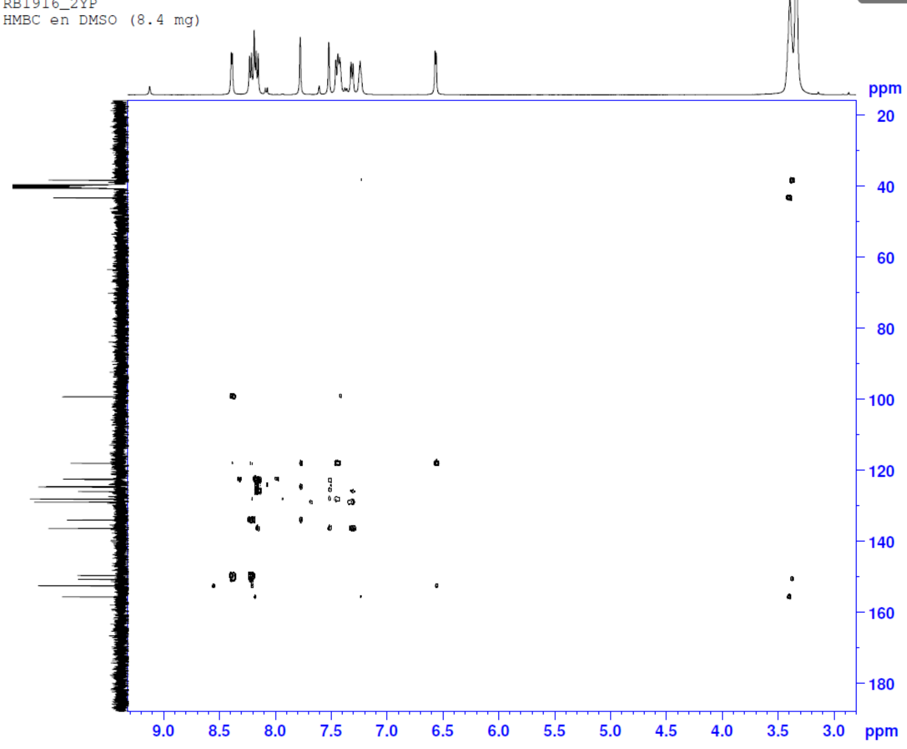


```
Current Data Parameters
NAME          RB1916_2YP
EXPNO                  6
PROCNO                 1

F2 - Acquisition Parameters
Date_           20231215
Time              7.04 h
INSTRUM           Avance
PROBHD    Z124031_0013 (
PULPROG      hmbcgplpndqf
TD                  4096
SOLVENT             DMSO
NS                    40
DS                    16
SWH           6250.000 Hz
FIDRES        3.051758 Hz
AQ           0.3276800 sec
RG                   101
DW              80.000 usec
DE               10.00 usec
TE               298.1 K
CNST2      145.0000000
CNST13      10.0000000
D0          0.00000300 sec
D1          1.50000000 sec
D2          0.00344828 sec
D6          0.05000000 sec
D16         0.00020000 sec
IN0         0.00001530 sec
TDav                   1
SFO1       500.1328637 MHz
NUC1                  1H
P1                  5.00 usec
P2                 10.00 usec
PLW1        6.59779978 W
SFO2       125.7728799 MHz
NUC2                 13C
P3                 10.00 usec
PLW2       26.29999924 W
GPNAM[1]     SMSQ10.100
GPZ1               50.00 %
GPNAM[2]     SMSQ10.100
GPZ2               30.00 %
GPNAM[3]     SMSQ10.100
GPZ3               40.10 %
P16              1000.00 usec

F1 - Acquisition parameters
TD                   256
SFO1         125.7729 MHz
FIDRES     255.310455 Hz
SW            259.831 ppm
FnMODE                QF

F2 - Processing parameters
SI                  2048
SF         500.1300122 MHz
WDW                 SINE
SSB        0
LB         0 Hz
GB         0
PC                  1.40

F1 - Processing parameters
SI                  1024
MC2                   QF
SF         125.7577885 MHz
WDW                 SINE
SSB        0
LB         0 Hz
GB         0
```

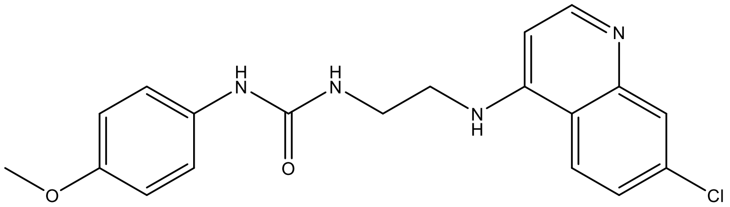
O
H
N
O
N
N
Cl

15

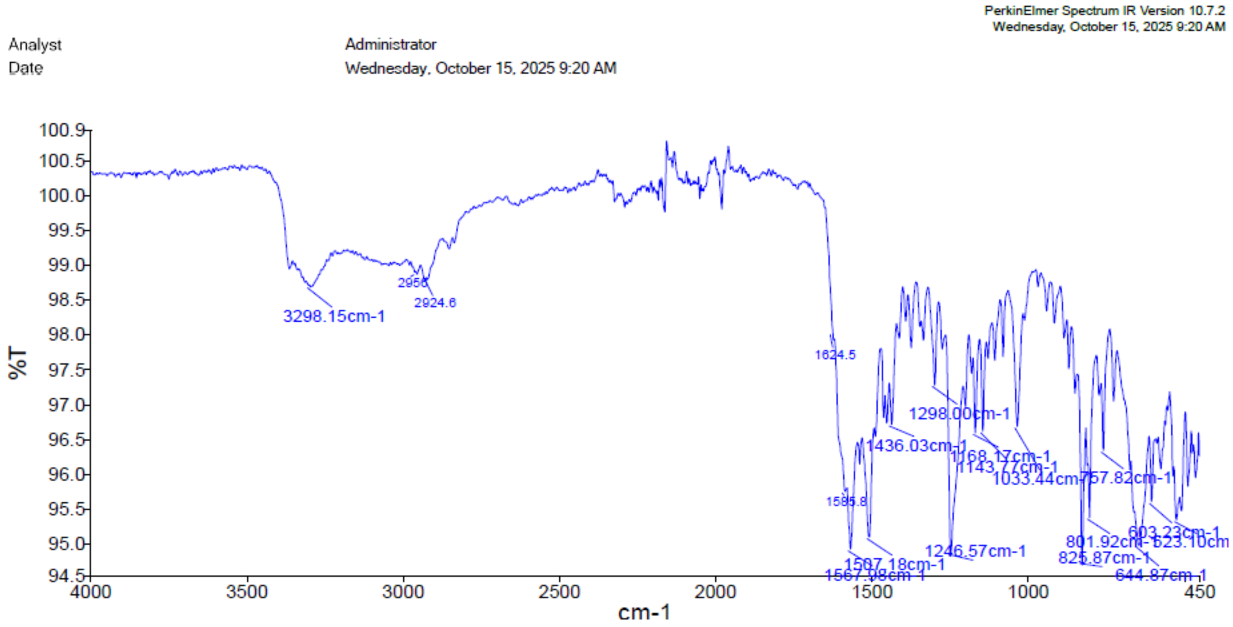
PerkinElmer Spectrum IR Version 10.7.2
Wednesday, October 15, 2025 9:20 AM
Analyst
Administrator
Date
Wednesday, October 15, 2025 9:20 AM
%T
cm-1
3298.15cm-1
2924.6
1624.5
1436.03cm-1
1298.00cm-1
1246.57cm-1
1033.44cm-1
757.82cm-1
801.92cm-1
825.87cm-1
644.87cm-1

RB1919_5YP
1H en DMSO (8.1 mg)

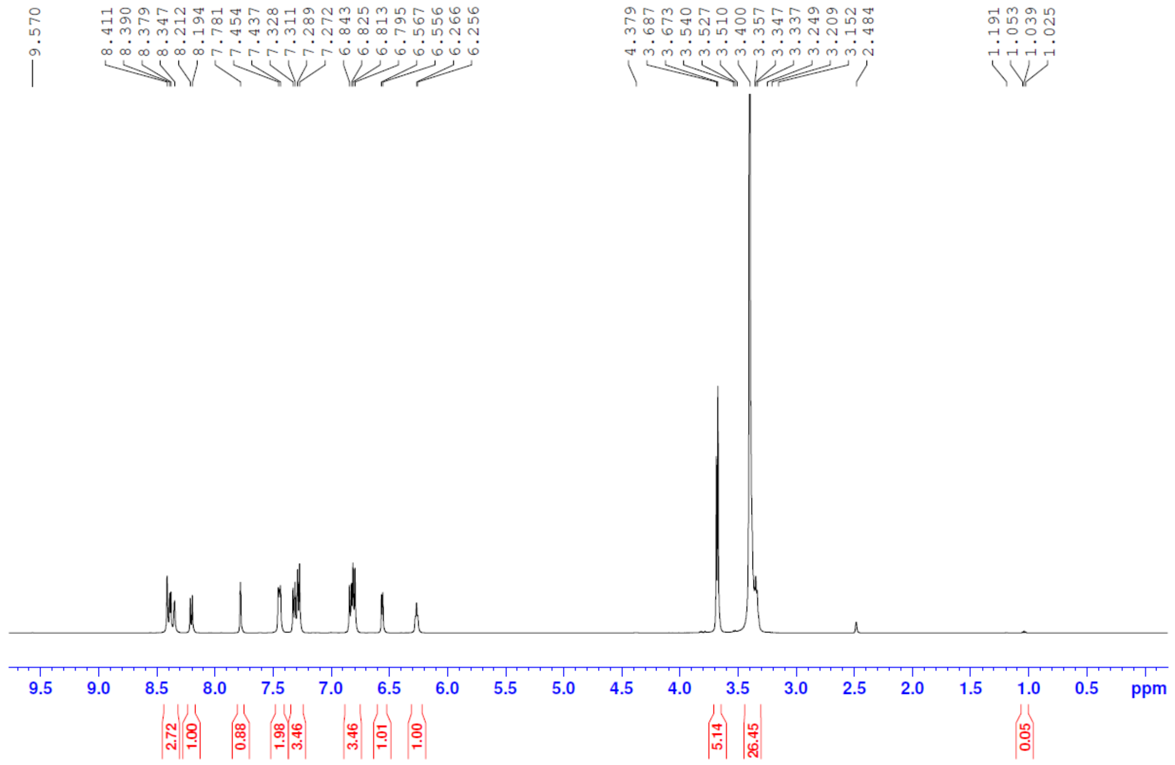

RB1919_5YP
13C en DMSO (8.1 mg)

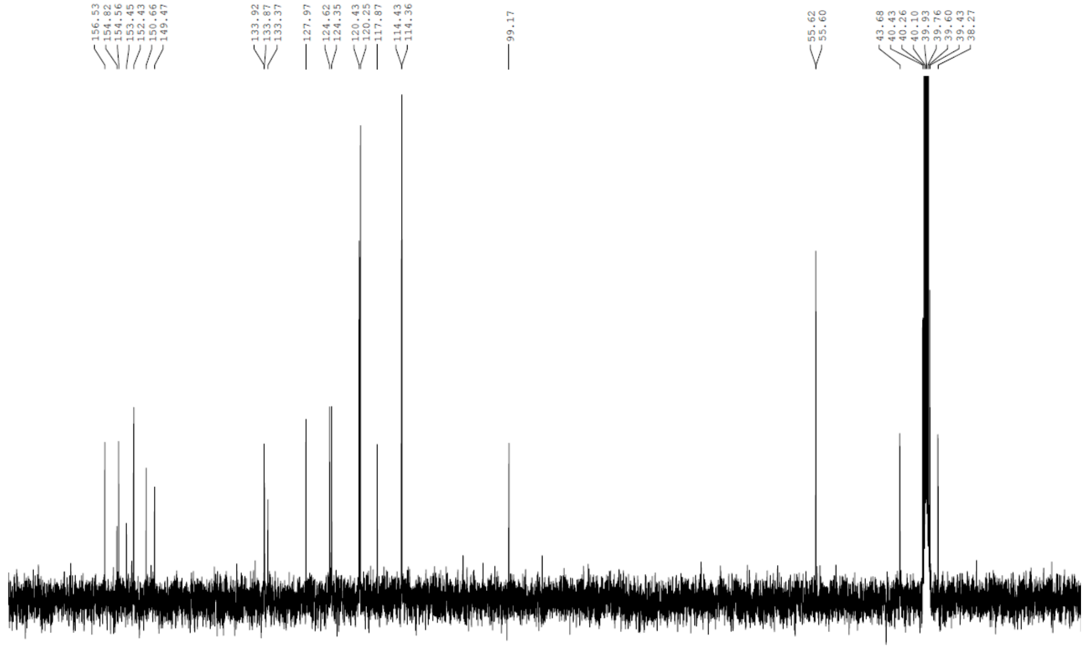

RB1919_5YP
DEPT en DMSO (8.1 mg)

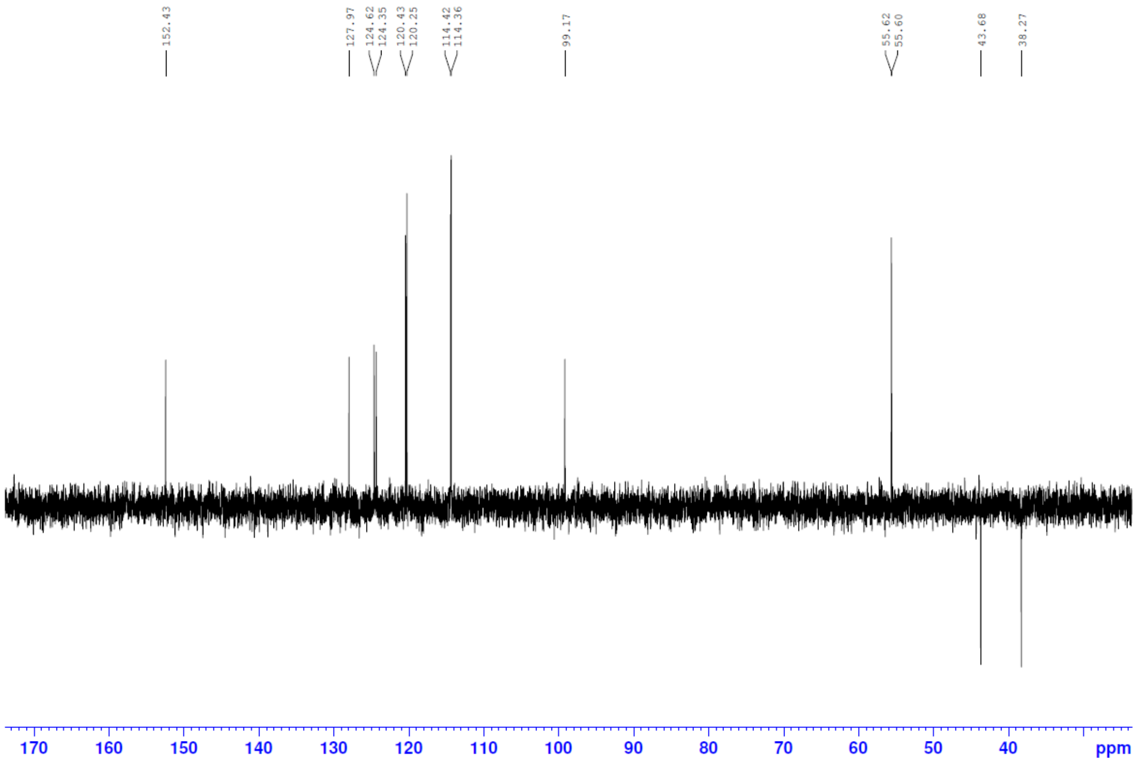

RB1919_5YP
COSY en DMSO (8.1 mg)

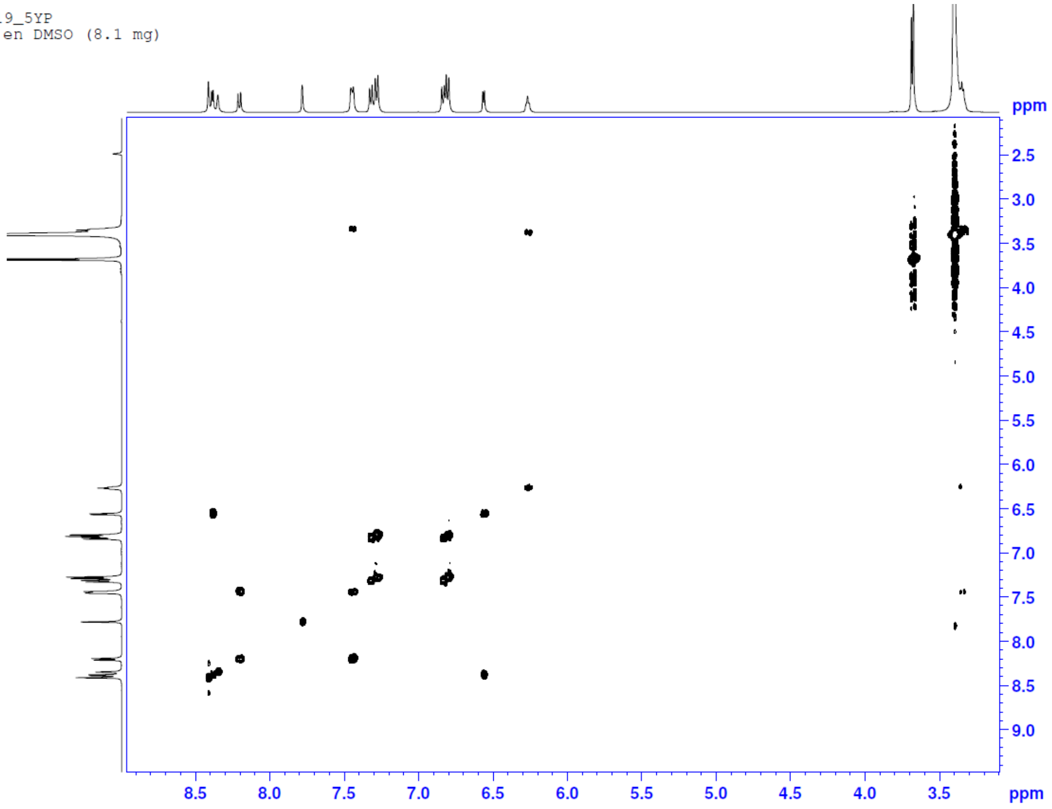

RB1919_5YP
HMQC en DMSO (8.1 mg)

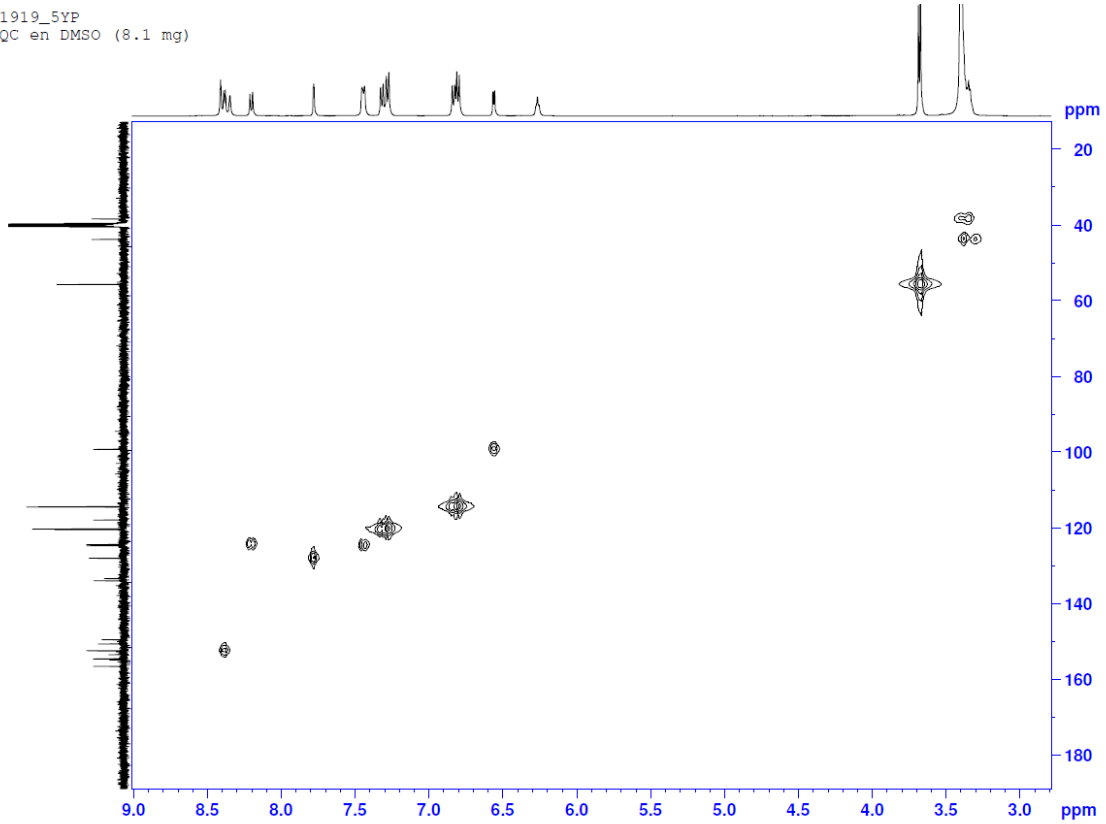

RB1919_5YP
HMBC en DMSO (8.1 mg)

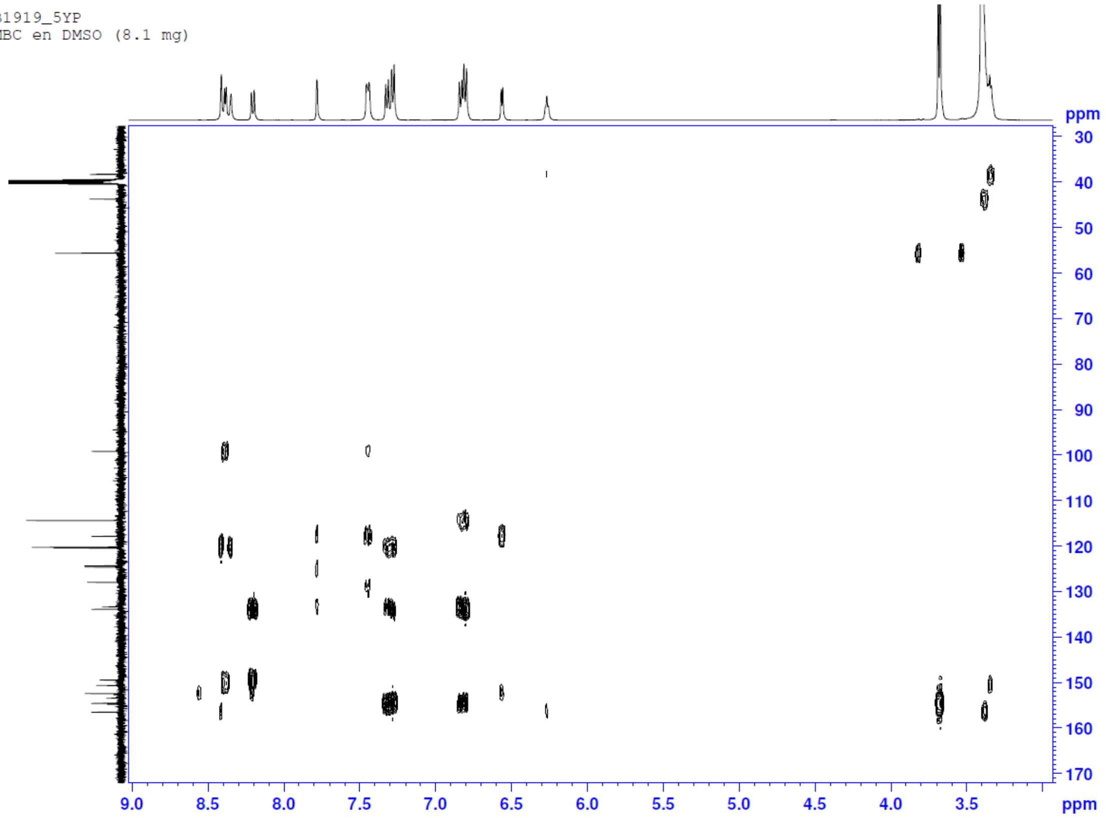

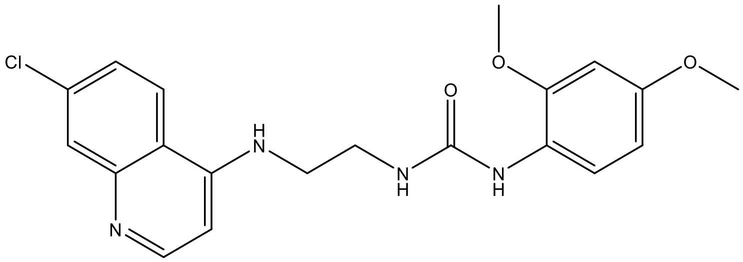
Cl
H
N
N
O
N
H
N
H
O
O

16

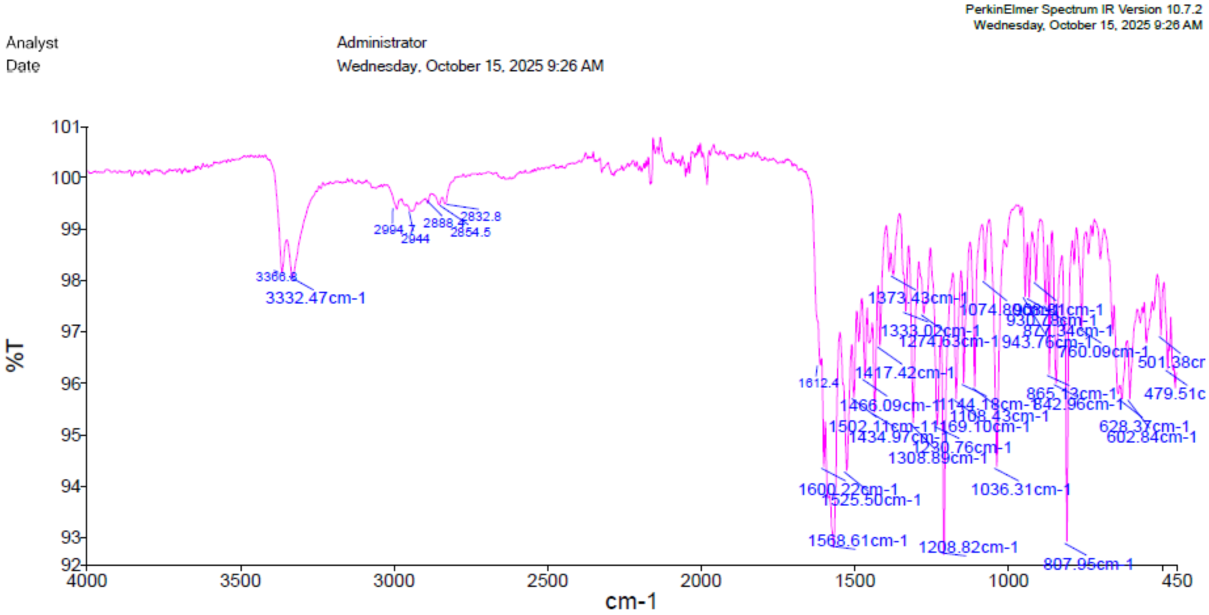
PerkinElmer Spectrum IR Version 10.7.2
Wednesday, October 15, 2025 9:26 AM
Analyst
Administrator
Date
Wednesday, October 15, 2025 9:26 AM
%T
cm-1
3332.47cm-1
1568.61cm-1
1208.82cm-1
807.95cm-1
1036.31cm-1
1600.22cm-1
1525.50cm-1
1417.42cm-1
1373.43cm-1
760.09cm-1
602.84cm-1
628.37cm-1

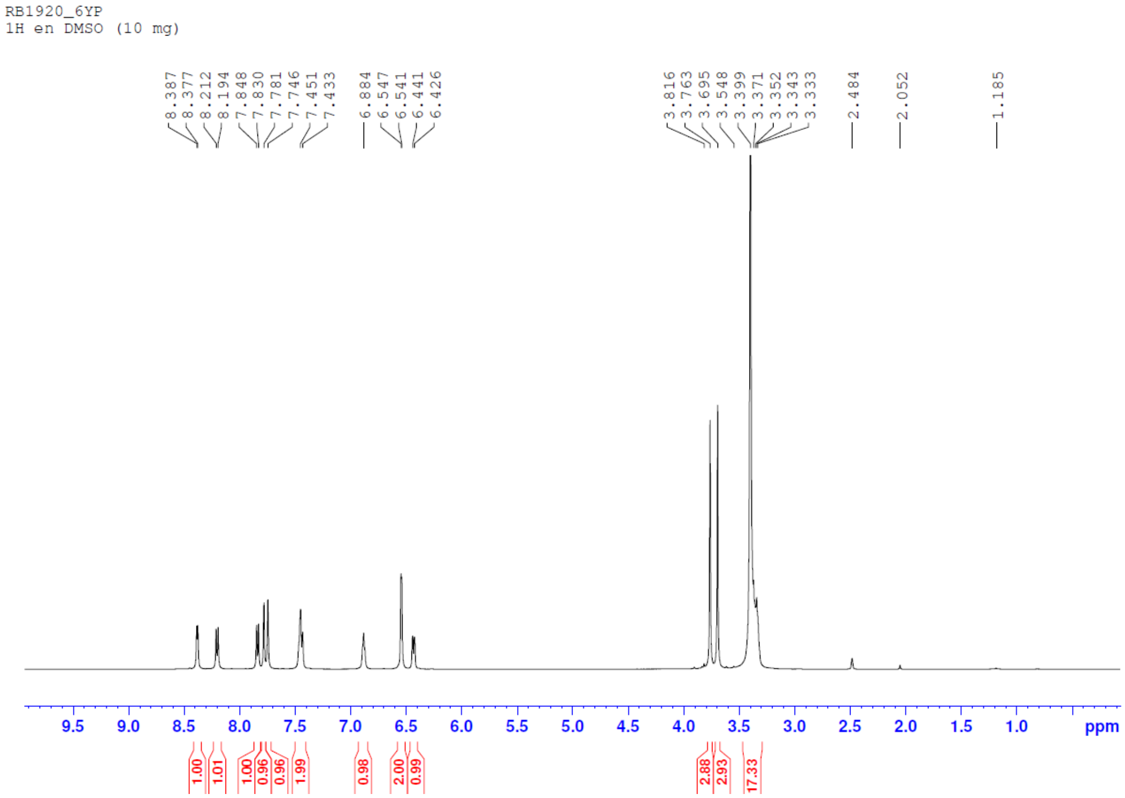
RB1920_6YP
1H en DMSO (10 mg)
8.387
8.377
8.212
8.194
7.848
7.830
7.781
7.746
7.451
7.433
6.884
6.547
6.541
6.441
6.426
3.816
3.763
3.695
3.548
3.399
3.371
3.352
3.343
3.333
2.484
2.052
1.185
9.5 9.0 8.5 8.0 7.5 7.0 6.5 6.0 5.5 5.0 4.5 4.0 3.5 3.0 2.5 2.0 1.5 1.0 ppm
1.00
1.01
1.00
0.96
0.96
1.99
0.98
2.00
0.99
2.88
2.93
17.33

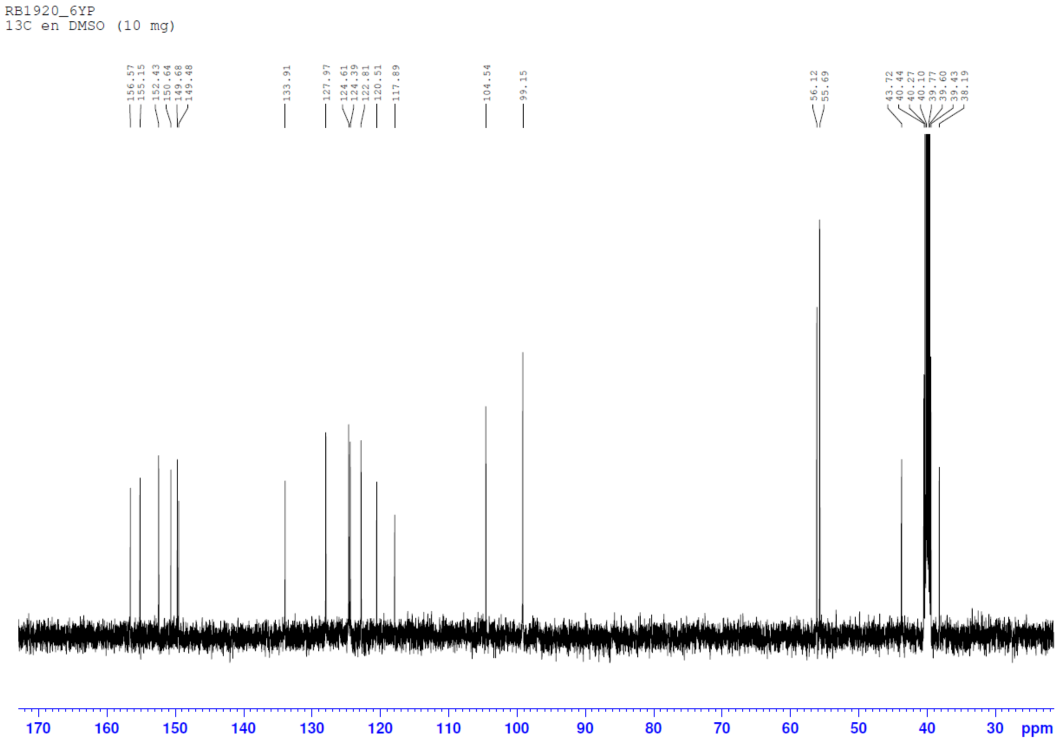
RB1920_6YP
13C en DMSO (10 mg)
156.57
155.15
152.43
150.64
149.68
149.48
133.91
127.97
124.61
124.39
122.81
120.51
117.89
104.54
99.15
56.12
55.69
43.72
40.44
40.27
40.10
39.77
39.60
39.43
38.19
170 160 150 140 130 120 110 100 90 80 70 60 50 40 30 ppm

RB1920_6YP
DEPT en DMSO (10 mg)

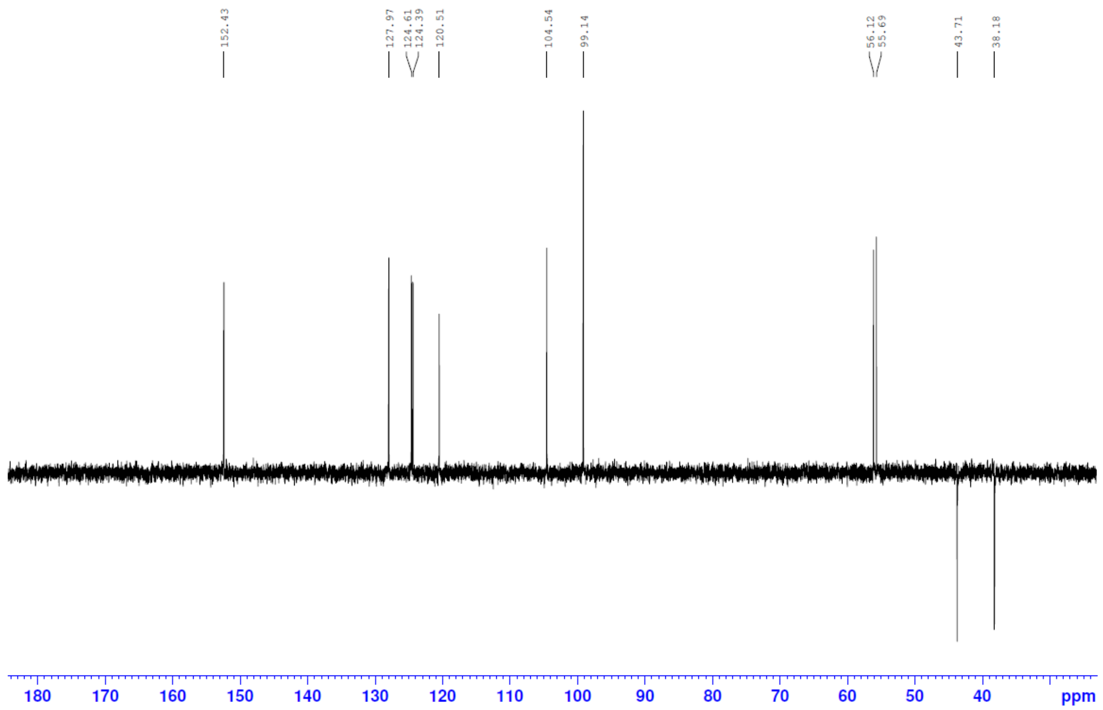

RB1920_6YP
COSY en DMSO (10 mg)

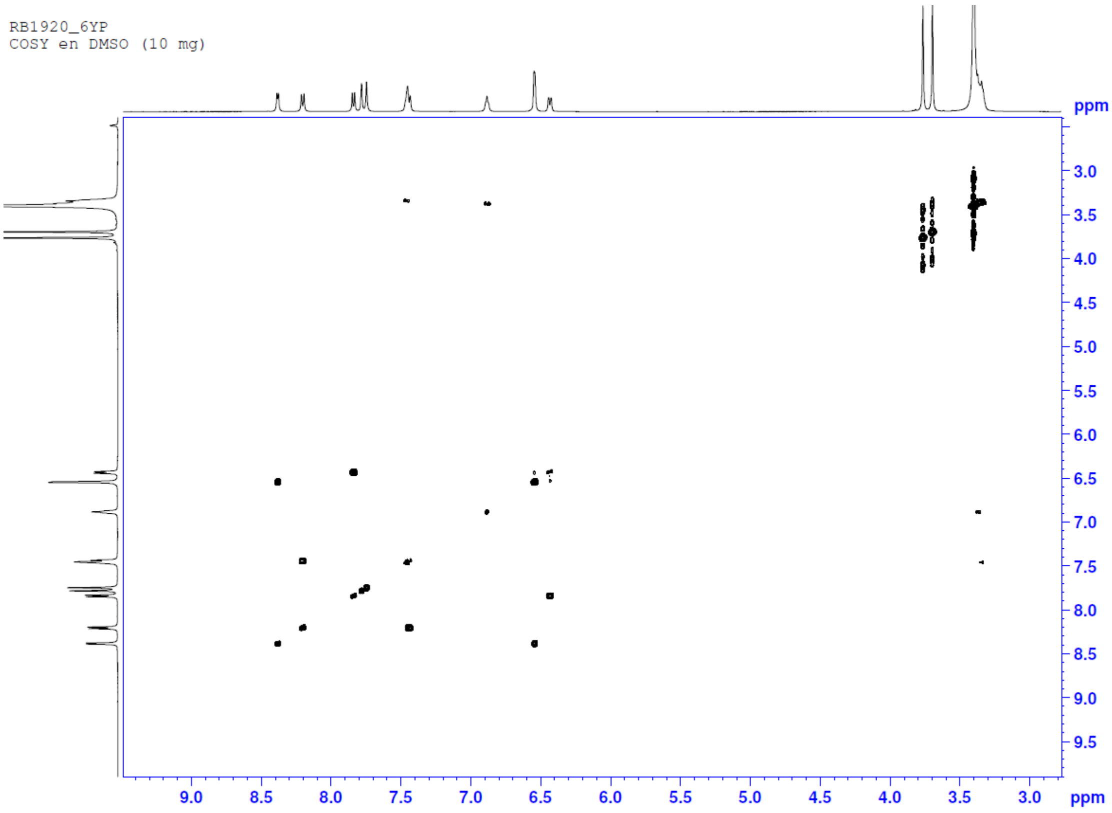

RB1920_6YP
HMQC en DMSO (10 mg)

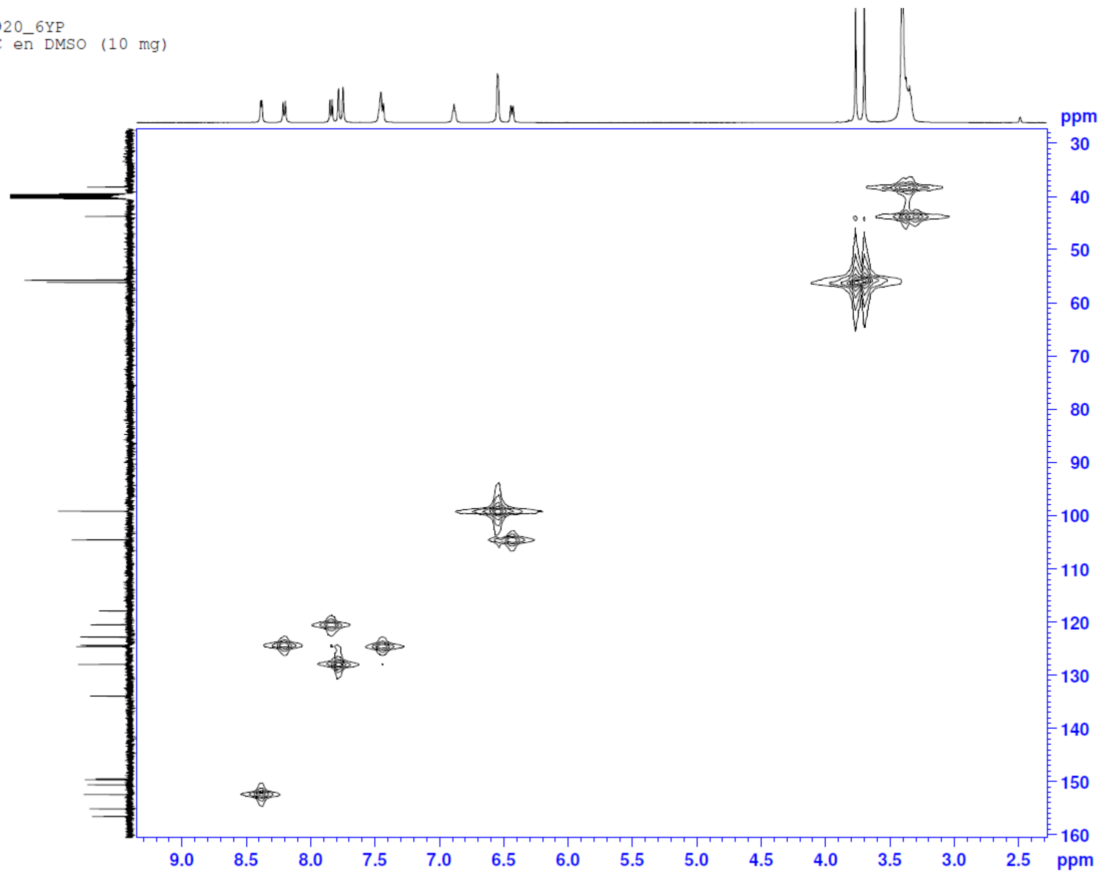

RB1920_6YP
HMBC en DMSO (10 mg)

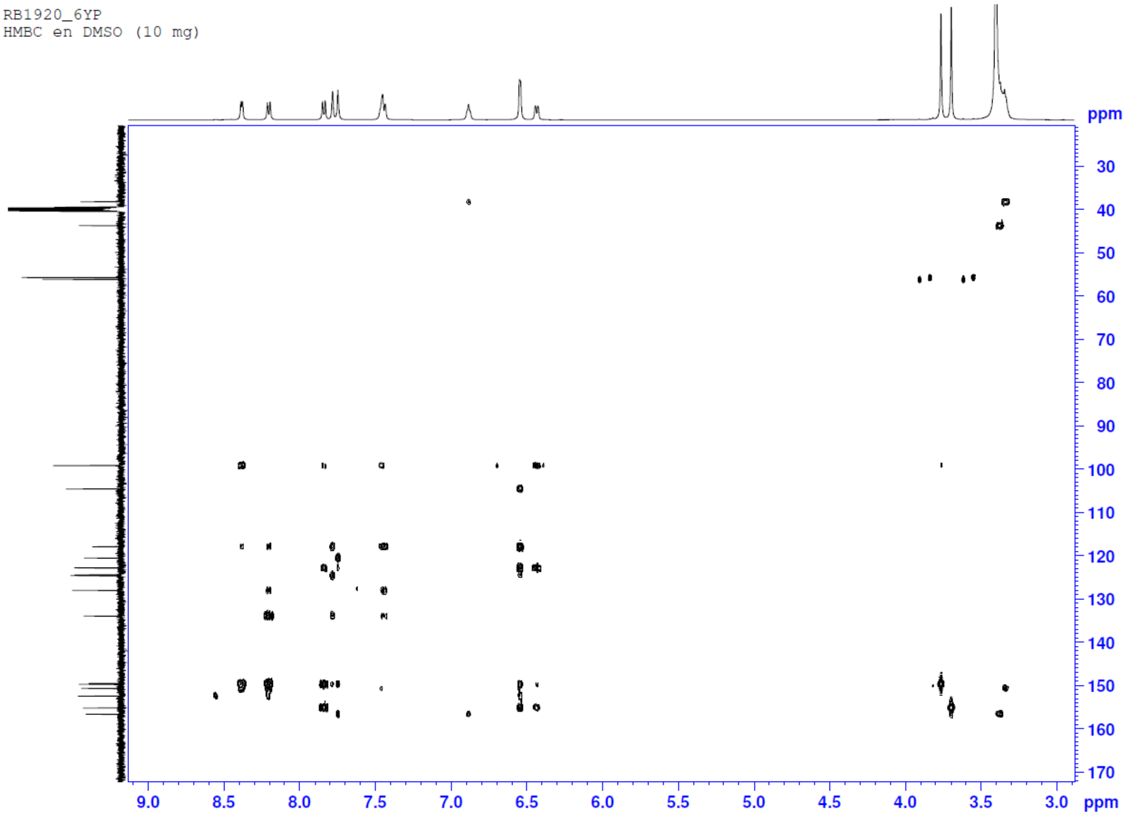

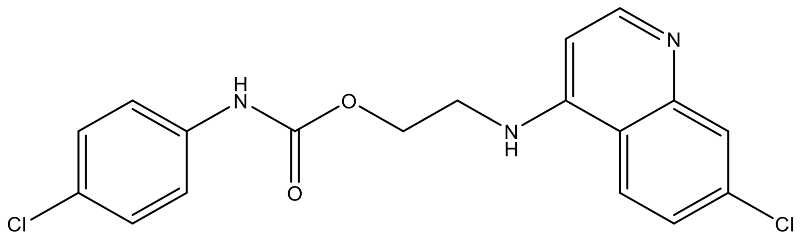

H
N
O
O
N
H
N
Cl
Cl

17

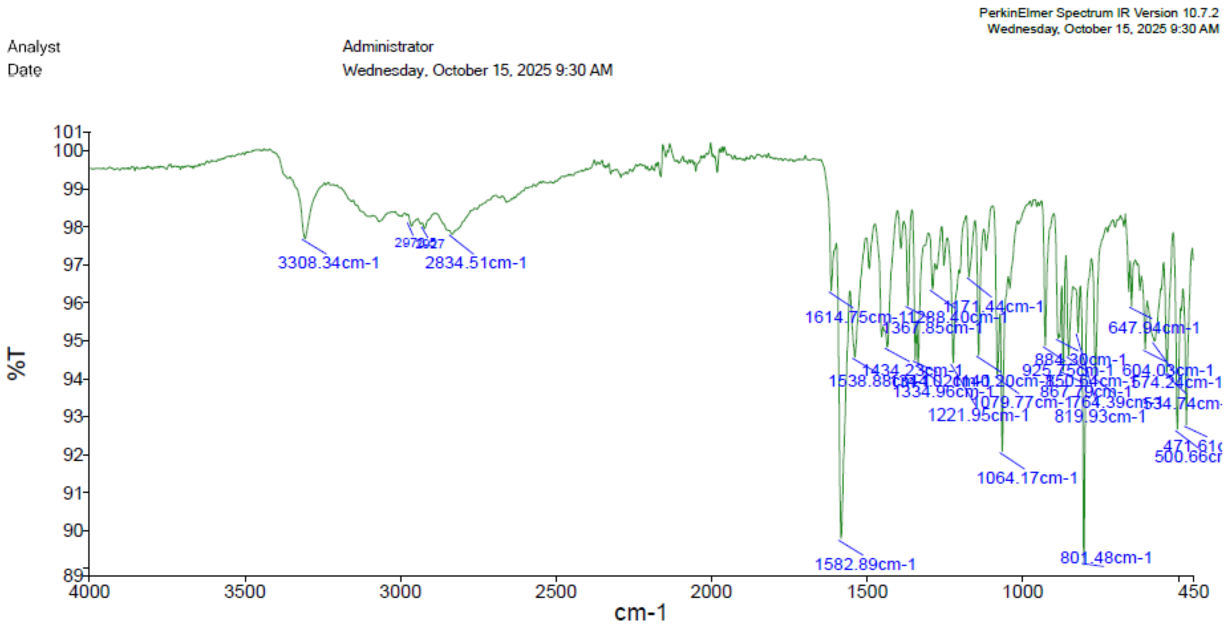

PerkinElmer Spectrum IR Version 10.7.2
Wednesday, October 15, 2025 9:30 AM
Analyst
Administrator
Date
Wednesday, October 15, 2025 9:30 AM
%T
cm-1
3308.34cm-1
2834.51cm-1
1614.75cm-1
1367.85cm-1
1171.44cm-1
647.94cm-1
884.30cm-1
1434.23cm-1
1334.96cm-1
1079.77cm-1
1221.95cm-1
819.93cm-1
1064.17cm-1
1582.89cm-1
801.48cm-1

RB1931_7YP
1H en DMSO (9.5 mg)

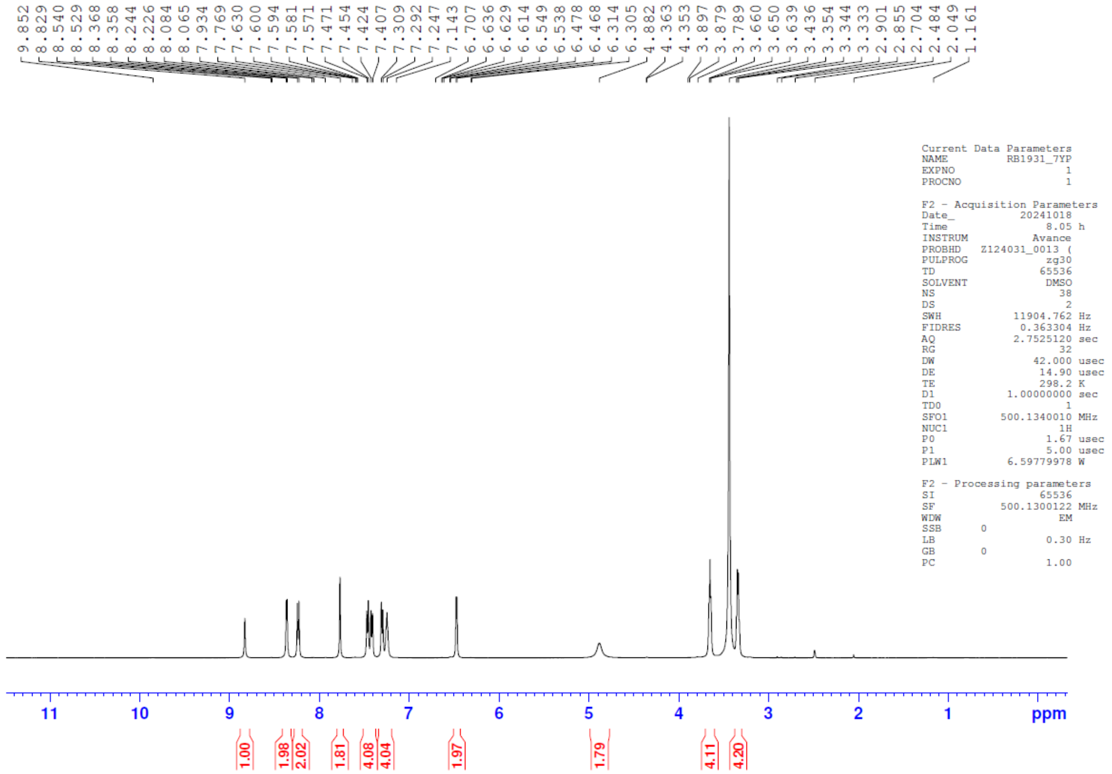

RB1931_7YP
13C en DMSO (9.5 mg)

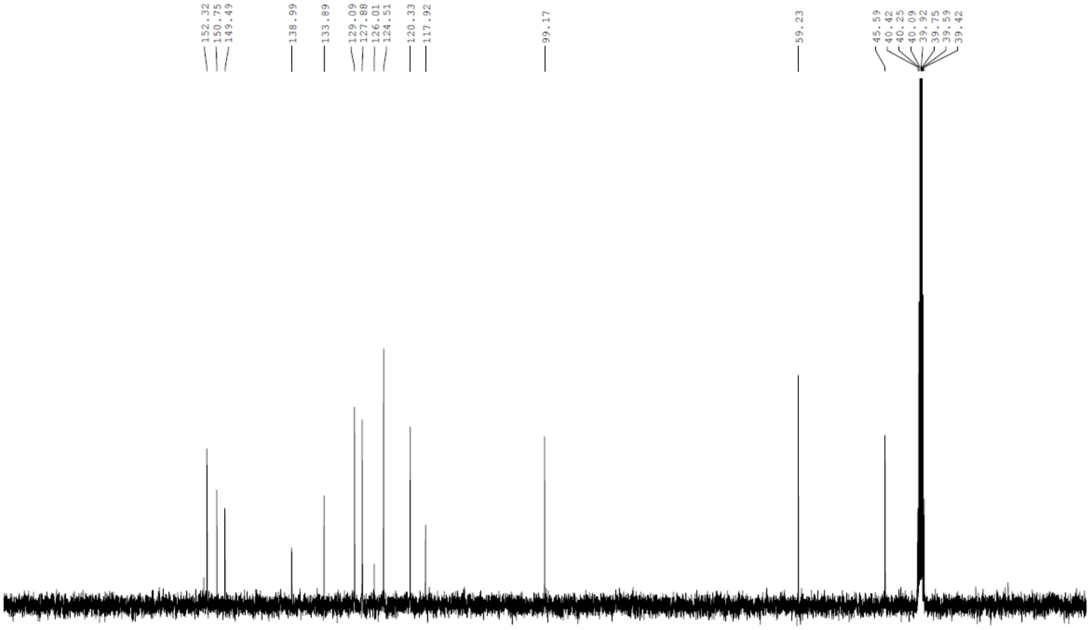

RB1931_7YP
DEPT en DMSO (9.5 mg)

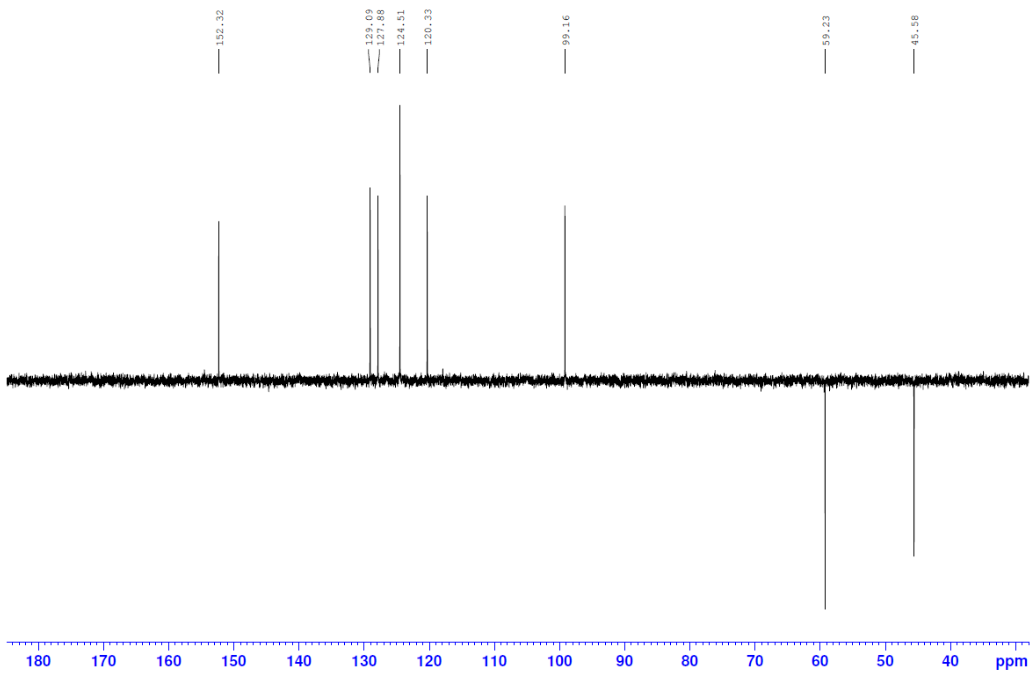

RB1931_7YP
COSY en DMSO (9.5 mg)

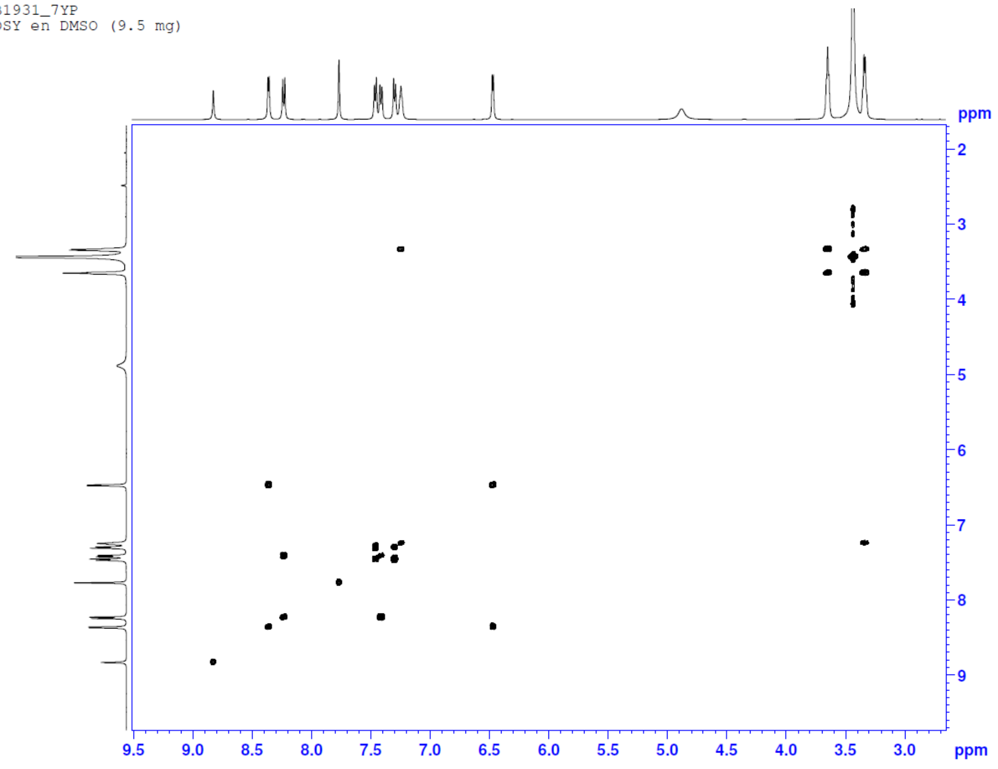

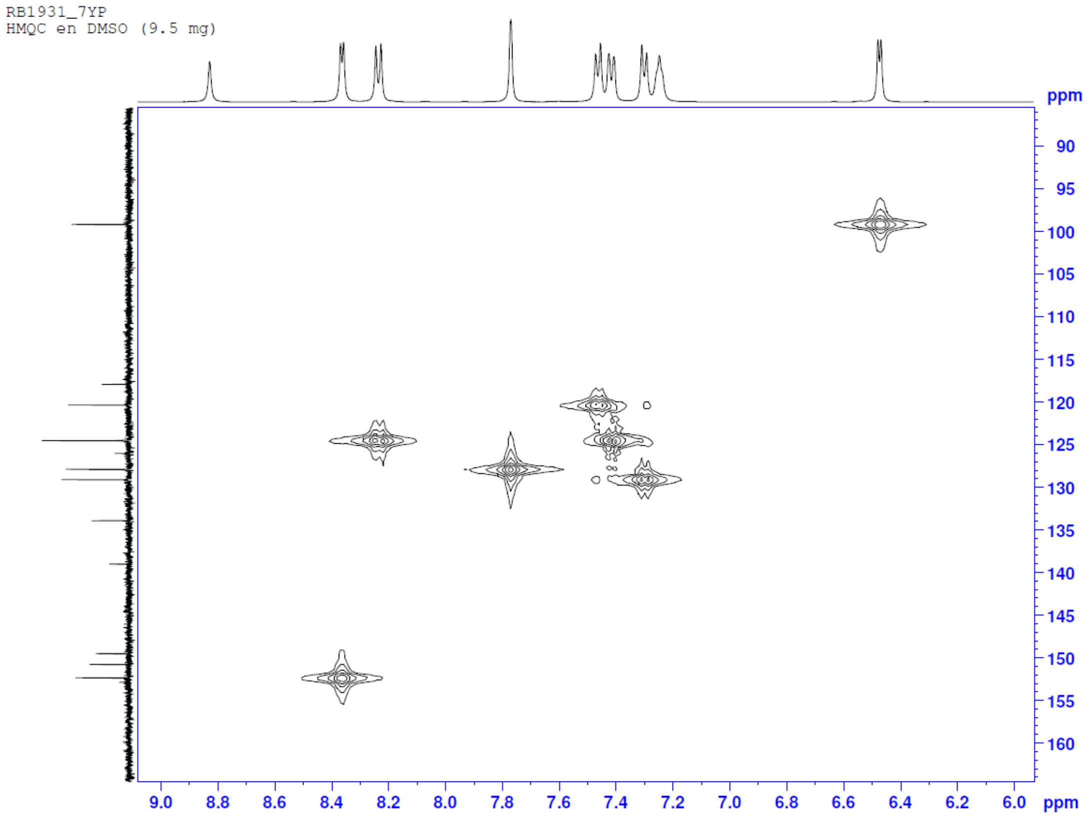
RB1931_7YP
HMQC en DMSO (9.5 mg)
ppm
90
95
100
105
110
115
120
125
130
135
140
145
150
155
160
9.0
8.8
8.6
8.4
8.2
8.0
7.8
7.6
7.4
7.2
7.0
6.8
6.6
6.4
6.2
6.0
ppm

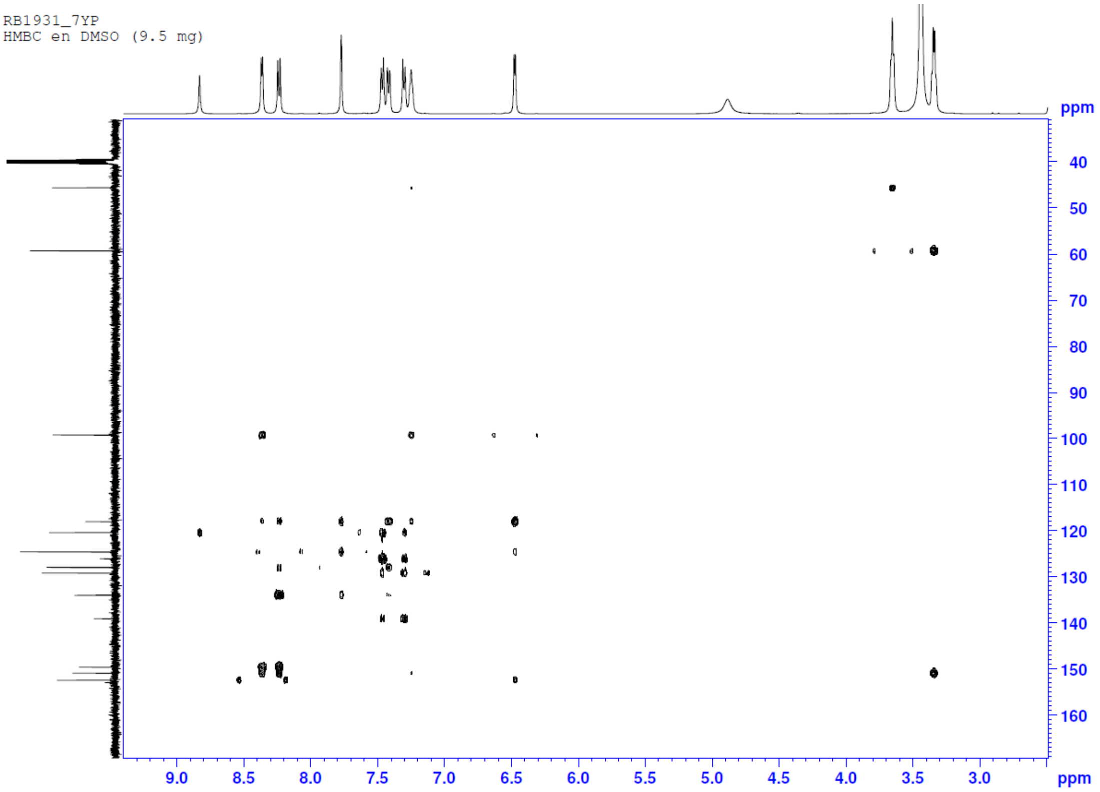
RB1931_7YP
HMBC en DMSO (9.5 mg)
ppm
40
50
60
70
80
90
100
110
120
130
140
150
160
9.0
8.5
8.0
7.5
7.0
6.5
6.0
5.5
5.0
4.5
4.0
3.5
3.0
ppm

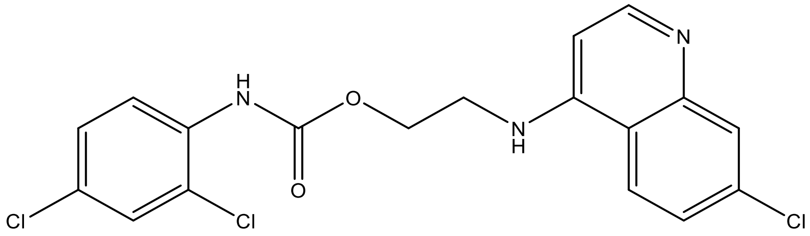

18

PerkinElmer Spectrum IR Version 10.7.2
Wednesday, October 15, 2025 9:33 AM

Analyst	Administrator
Date	Wednesday, October 15, 2025 9:33 AM

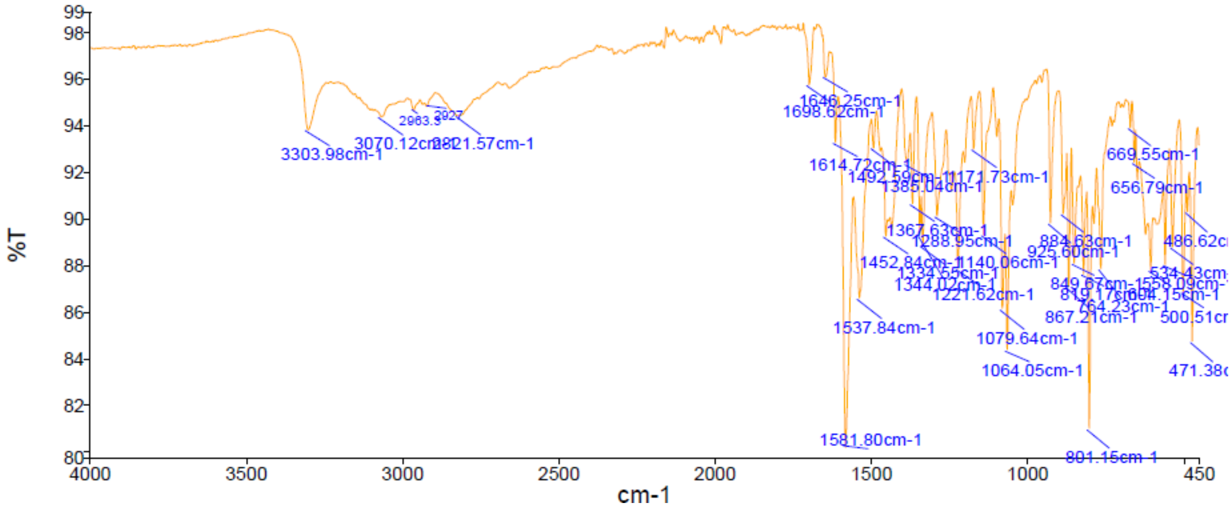

RB1932_8YP
1H en DMSO (9.7 mg)

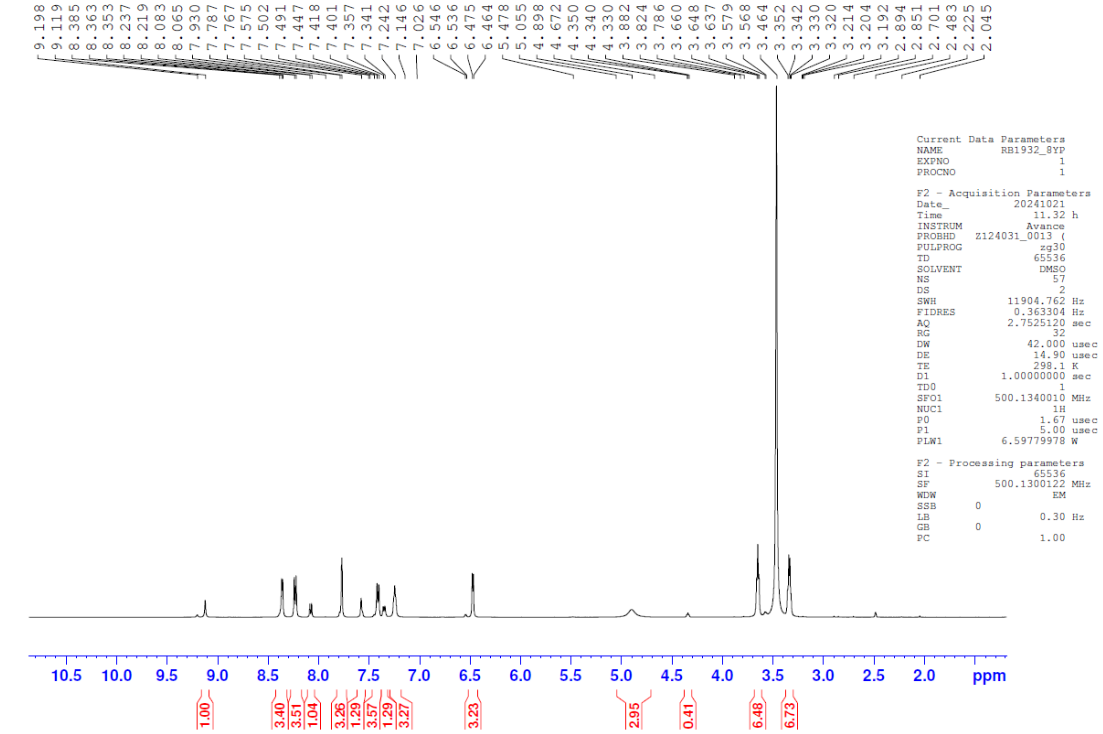

RB1932_8YP
13C en DMSO (9.7 mg)

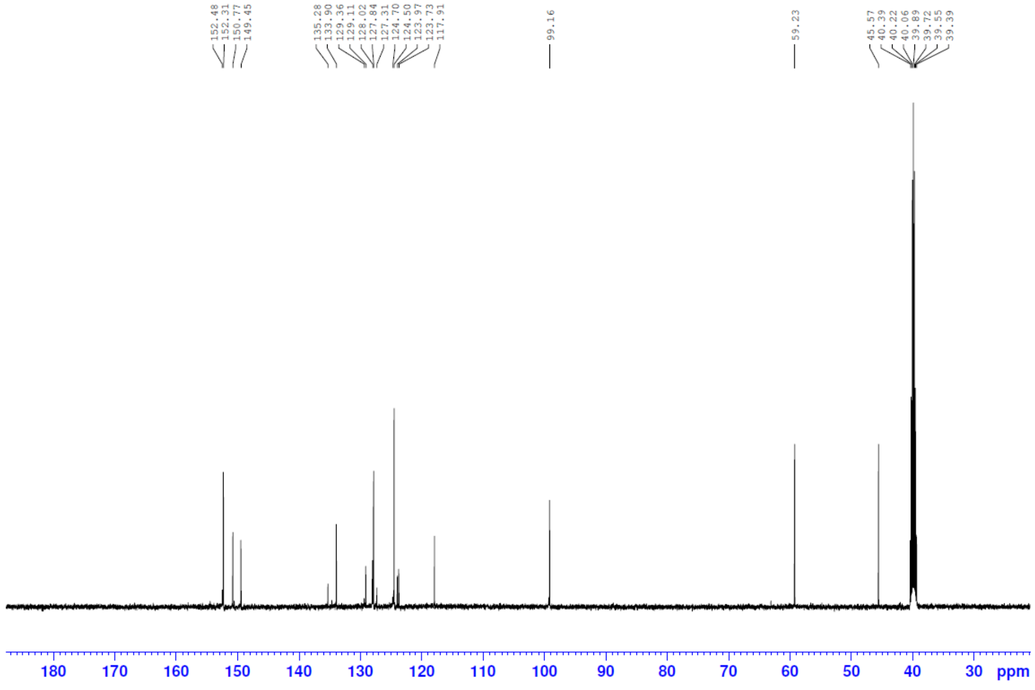

RB1932_8YP
DEPT en DMSO (9.7 mg)

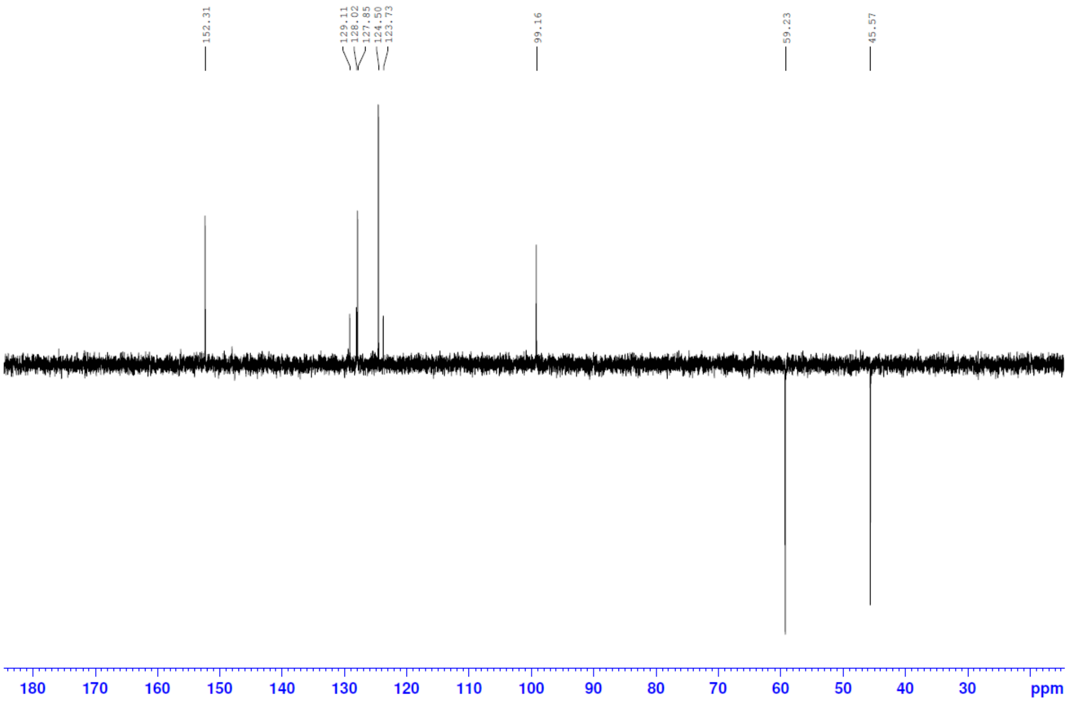

RB1932_8YP
COSY en DMSO (9.7 mg)

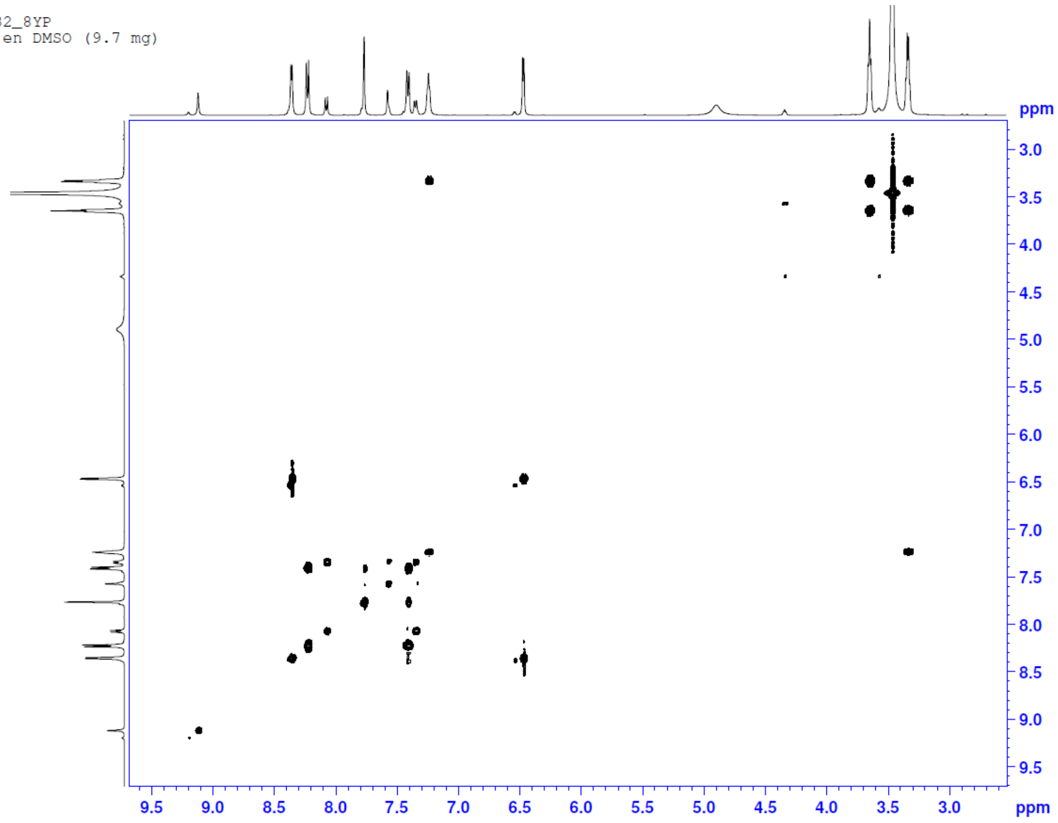

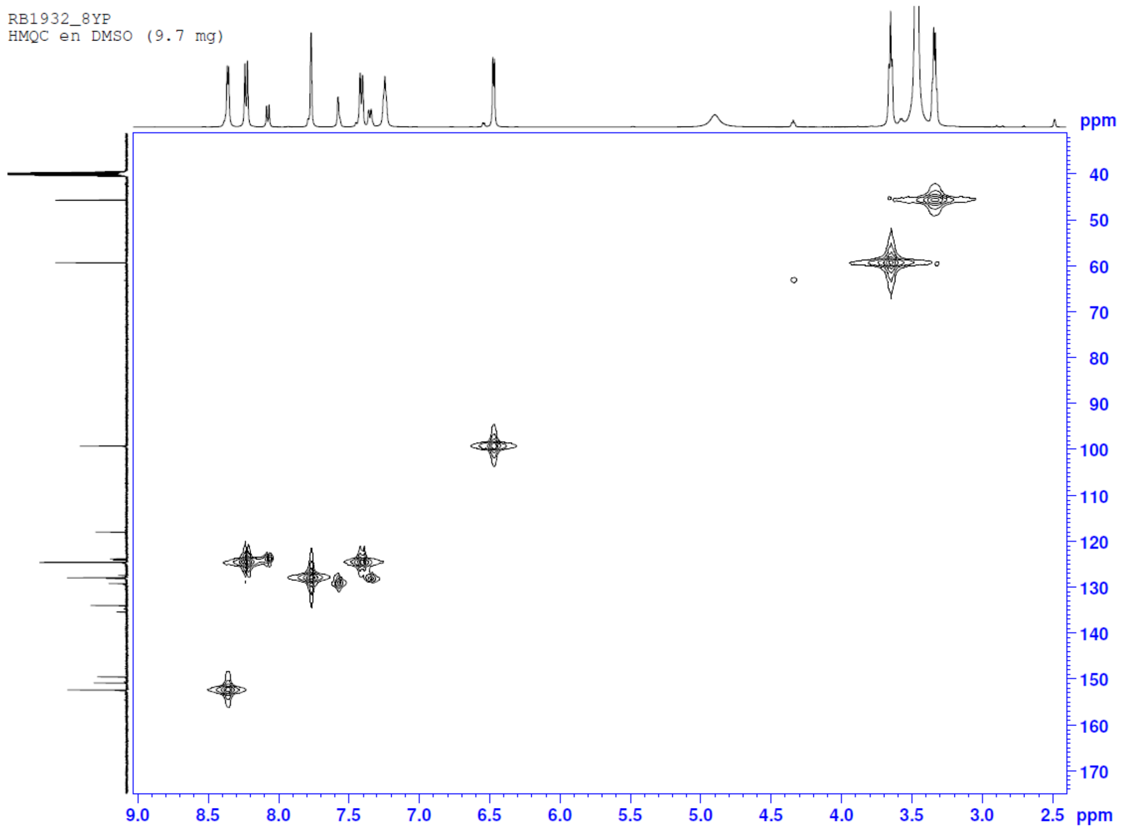
RB1932_8YP
HMQC en DMSO (9.7 mg)
ppm
40
50
60
70
80
90
100
110
120
130
140
150
160
170
9.0
8.5
8.0
7.5
7.0
6.5
6.0
5.5
5.0
4.5
4.0
3.5
3.0
2.5
ppm

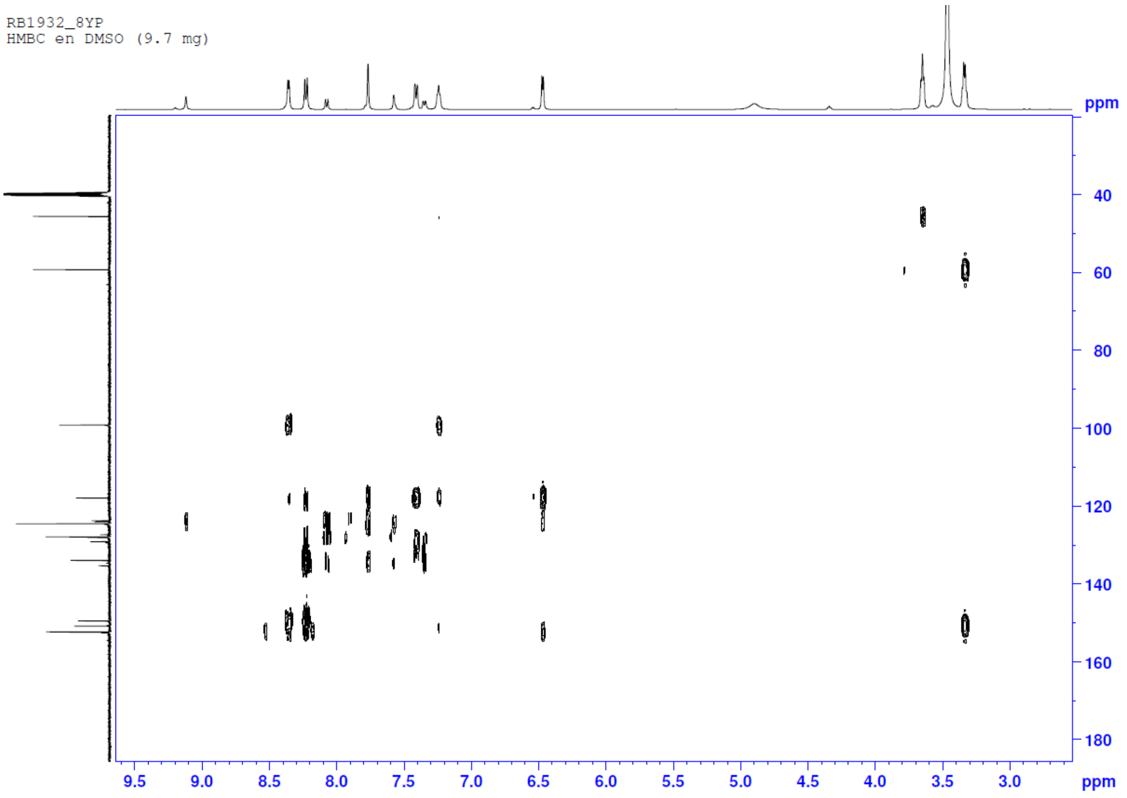
RB1932_8YP
HMBC en DMSO (9.7 mg)
ppm
40
60
80
100
120
140
160
180
9.5
9.0
8.5
8.0
7.5
7.0
6.5
6.0
5.5
5.0
4.5
4.0
3.5
3.0
ppm

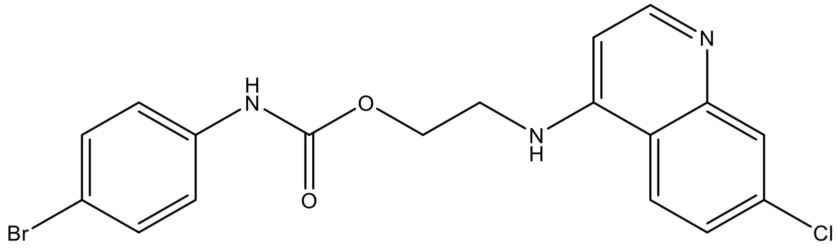
Br
H
N
O
O
N
H
N
Cl

19

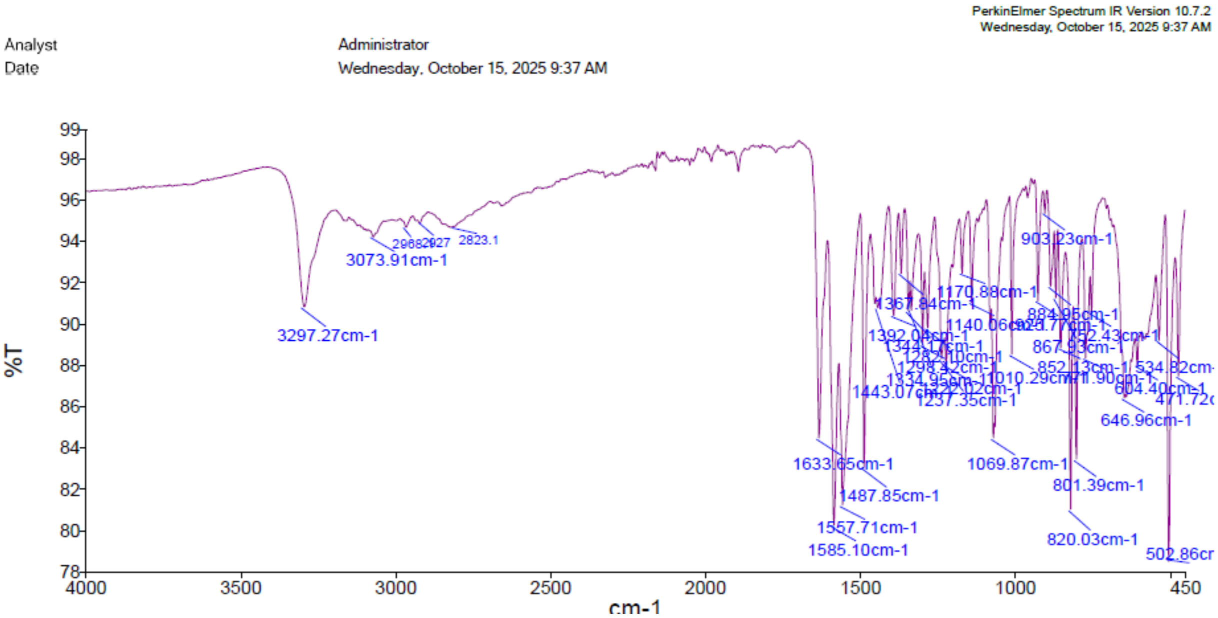
PerkinElmer Spectrum IR Version 10.7.2
Wednesday, October 15, 2025 9:37 AM
Analyst
Administrator
Date
Wednesday, October 15, 2025 9:37 AM
%T
cm-1
3297.27cm-1
3073.91cm-1
2823.1
1633.65cm-1
1487.85cm-1
1557.71cm-1
1585.10cm-1
1069.87cm-1
801.39cm-1
820.03cm-1
903.23cm-1
646.96cm-1

RB1934_10YP
1H en DMSO (7,4 mg)

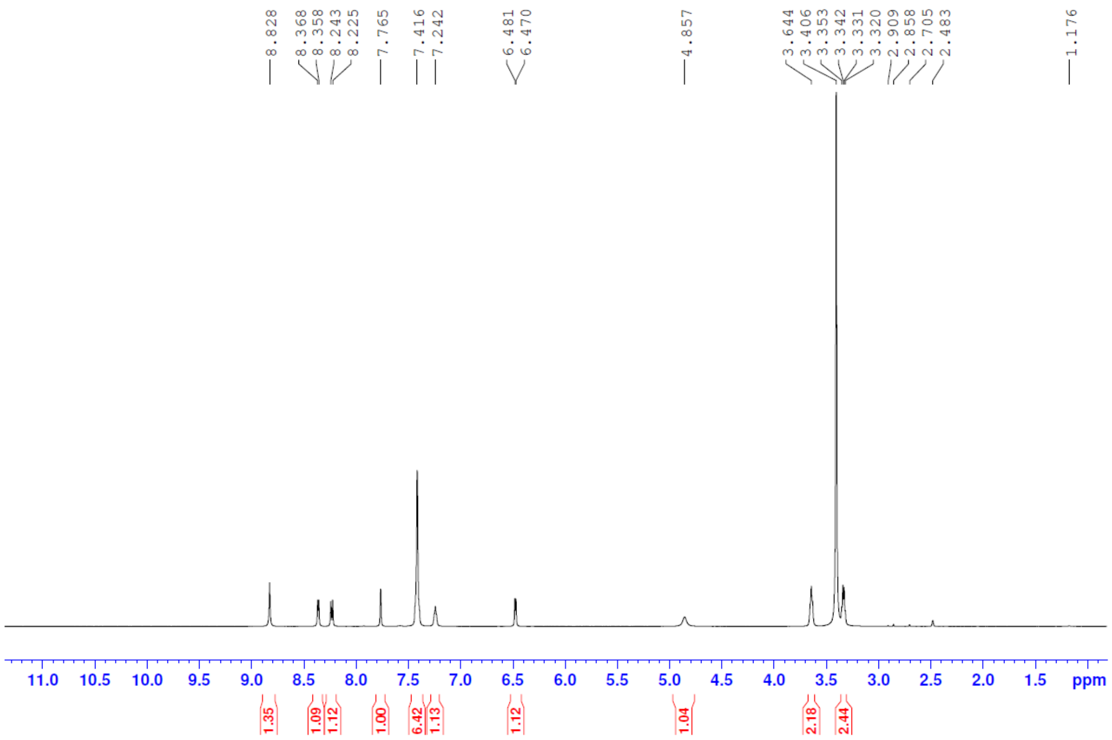

RB1934_10YP
13C en COSY (7,4 mg)

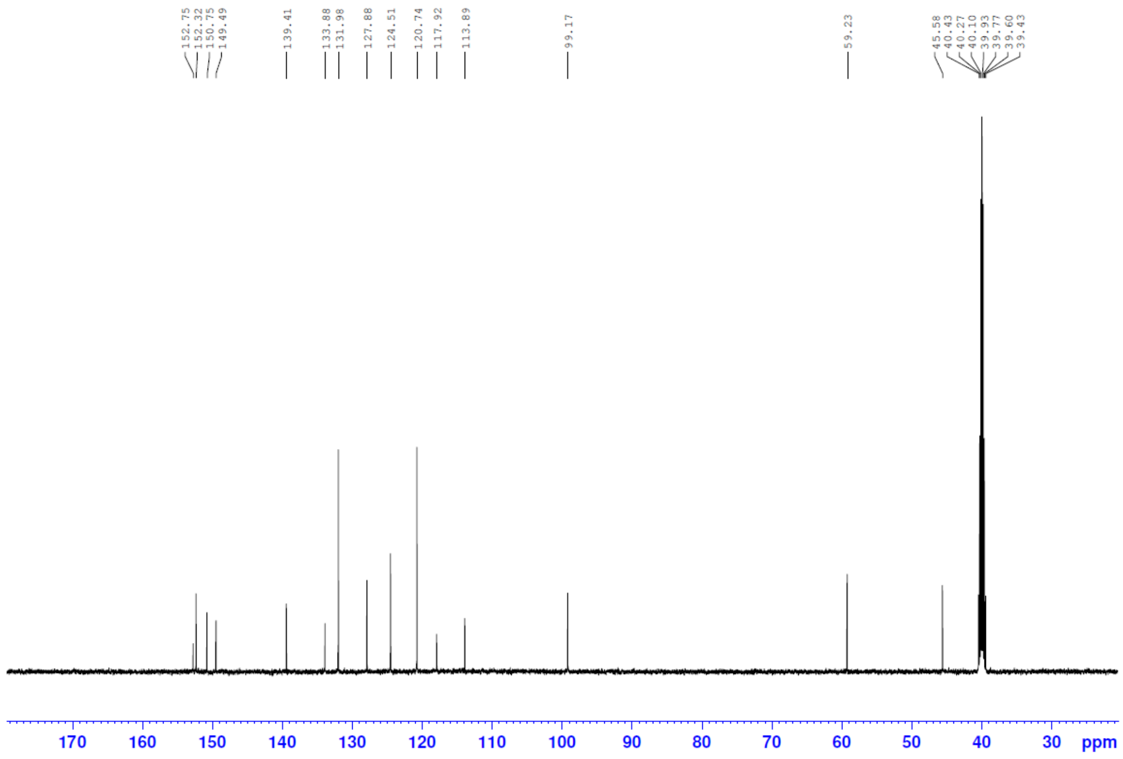

RB1934_10YP
DEPT en COSY (7,4 mg)

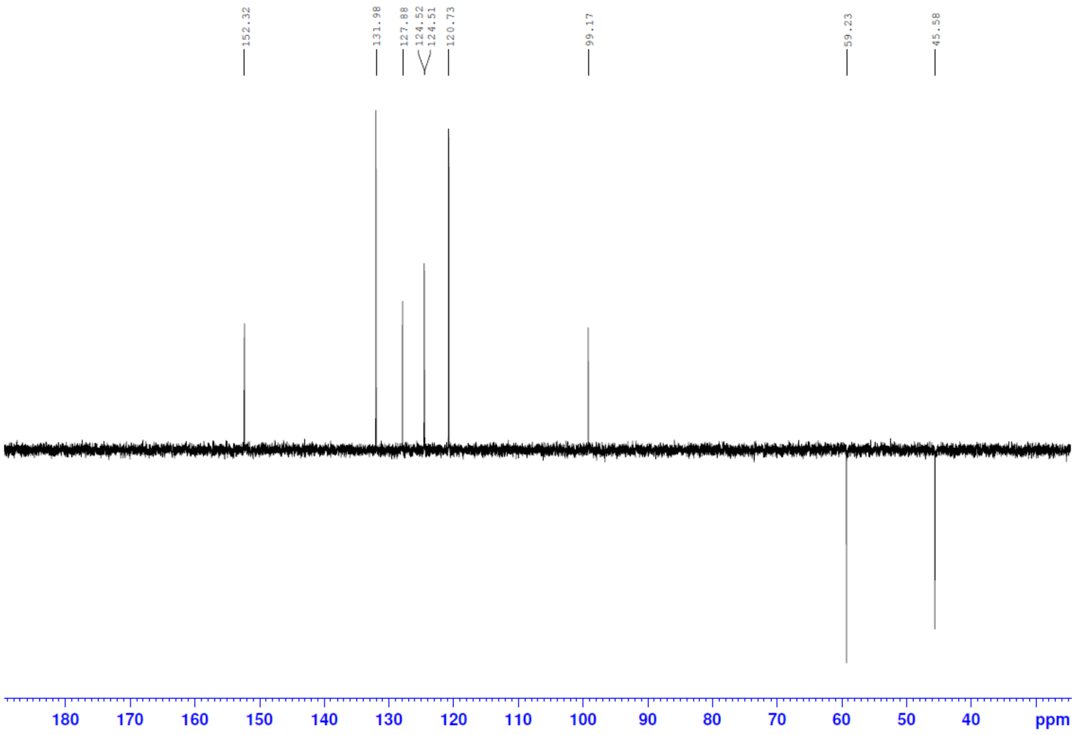

RB1934_10YP
1H en COSY (7,4 mg)

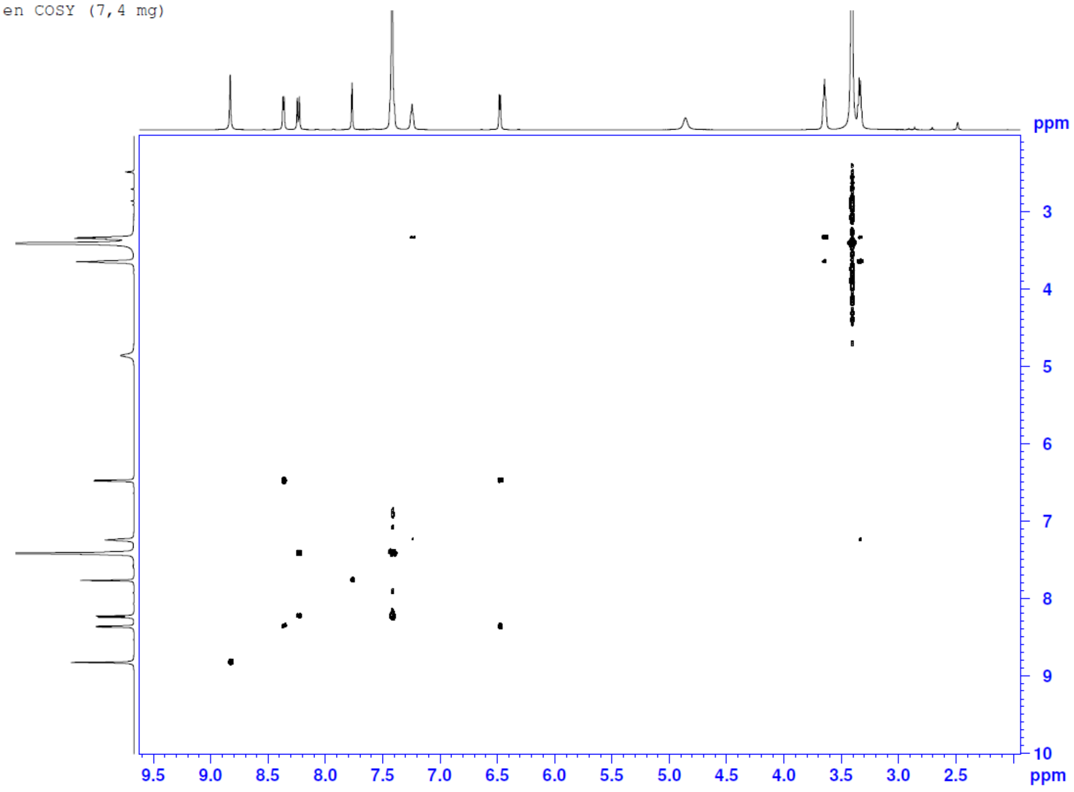

RB1934_10YP
HMQC en COSY (7,4 mg)

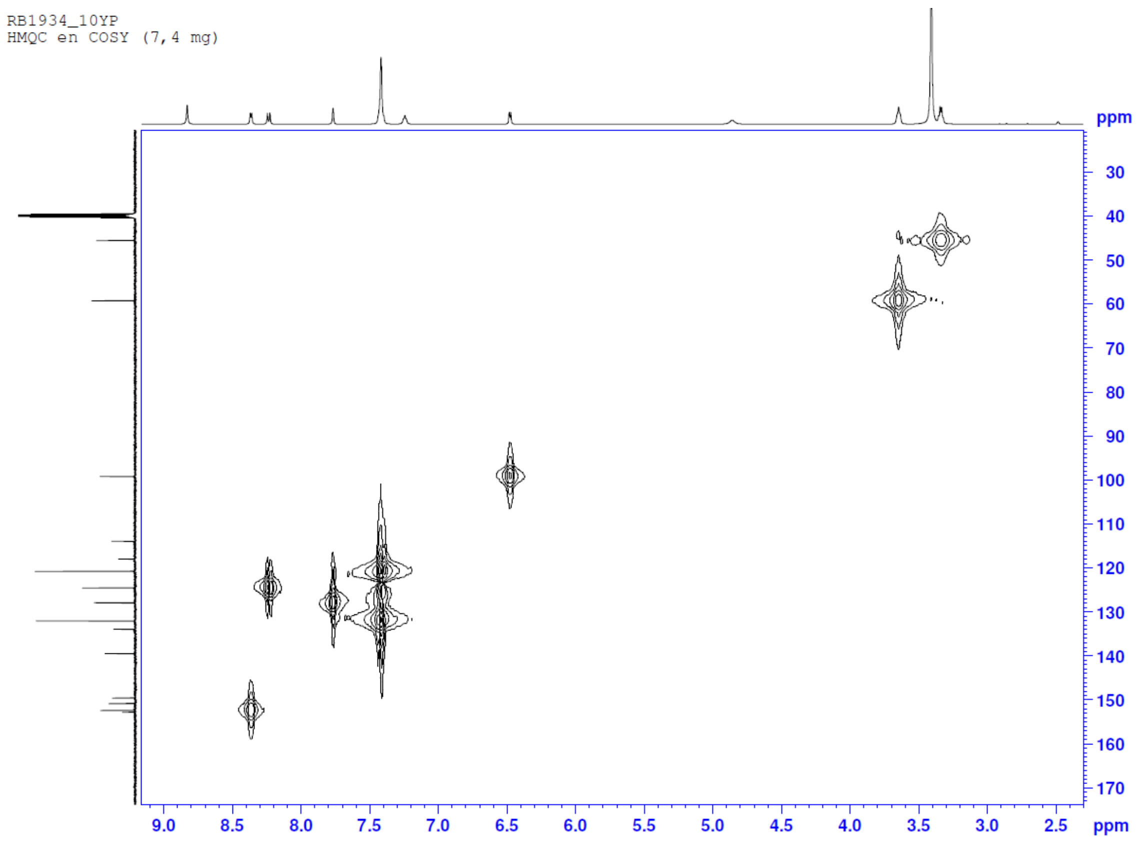

RB1934_10YP
HMBC en COSY (7,4 mg)

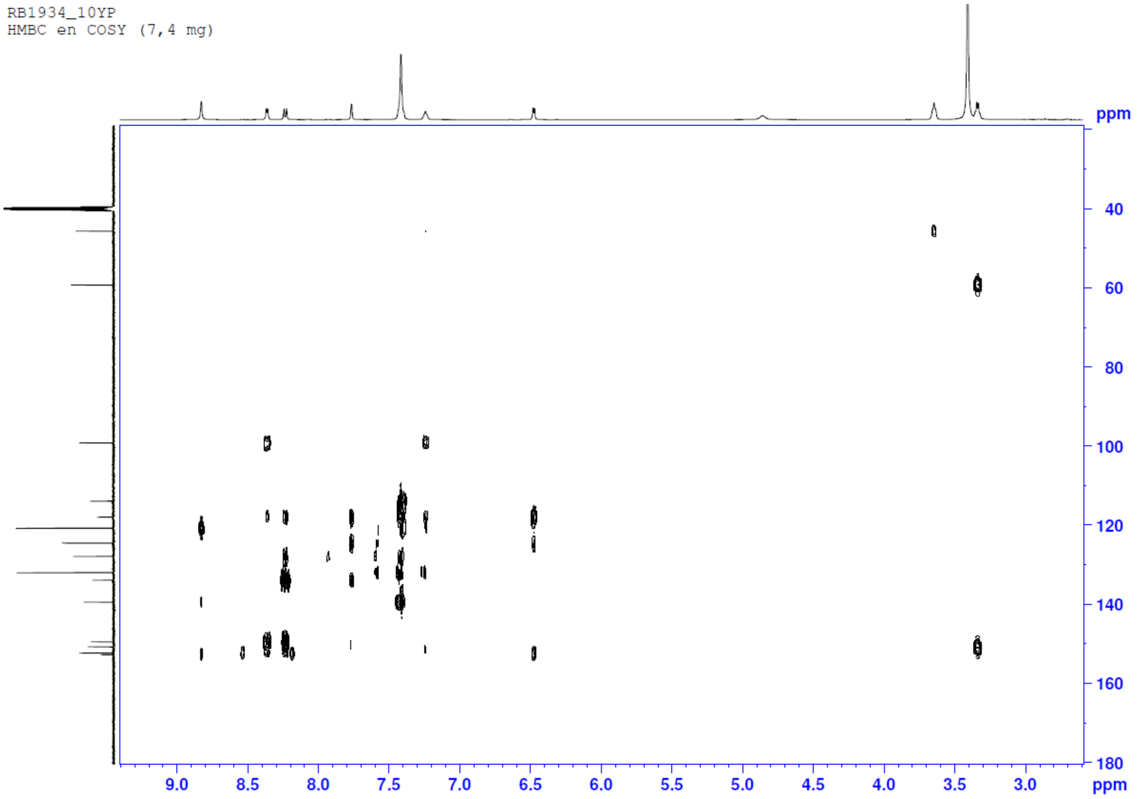

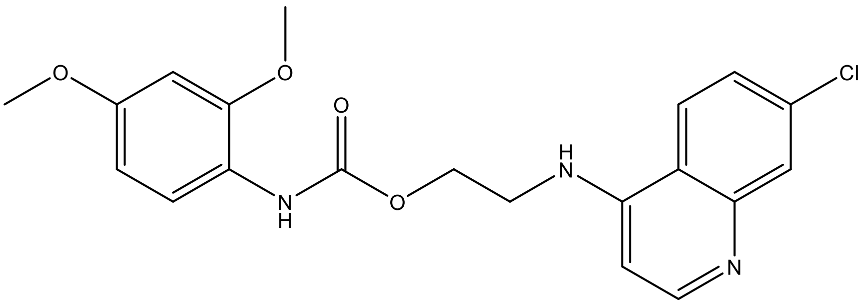
O
O
O
O
N
H
H
N
Cl
N

20

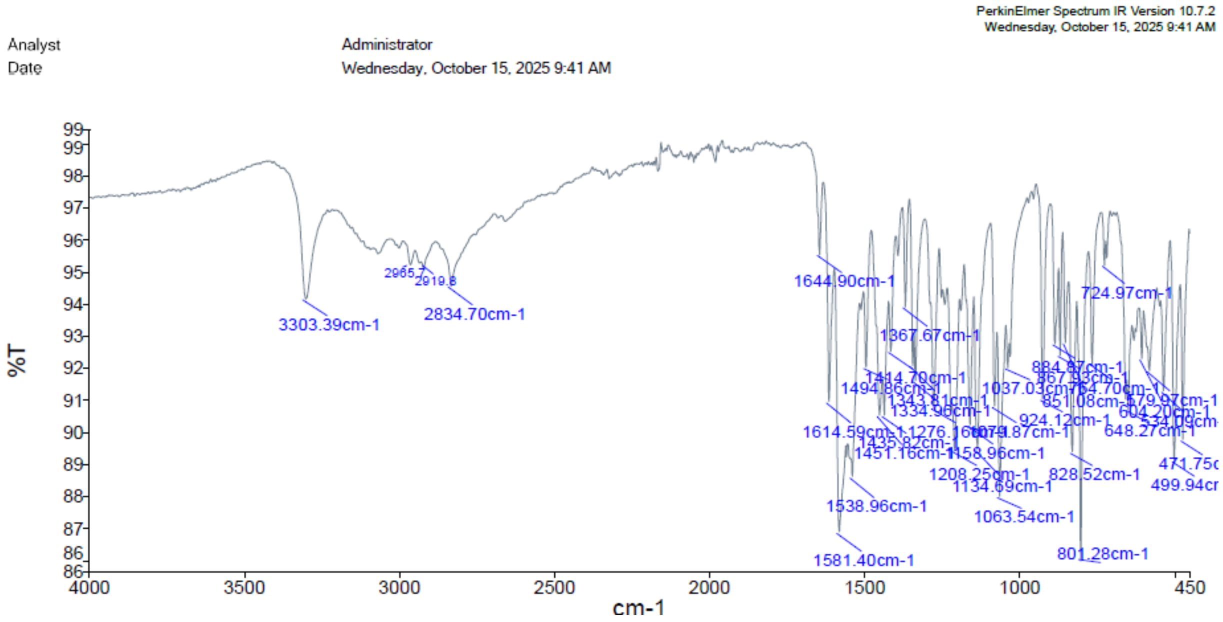
PerkinElmer Spectrum IR Version 10.7.2
Wednesday, October 15, 2025 9:41 AM
Analyst
Administrator
Date
Wednesday, October 15, 2025 9:41 AM
%T
cm-1
3303.39cm-1
2834.70cm-1
1644.90cm-1
1581.40cm-1
1538.96cm-1
724.97cm-1
801.28cm-1
828.52cm-1
1063.54cm-1
1367.67cm-1

RB1936_12YP
1H en DMSO (9.7 mg)

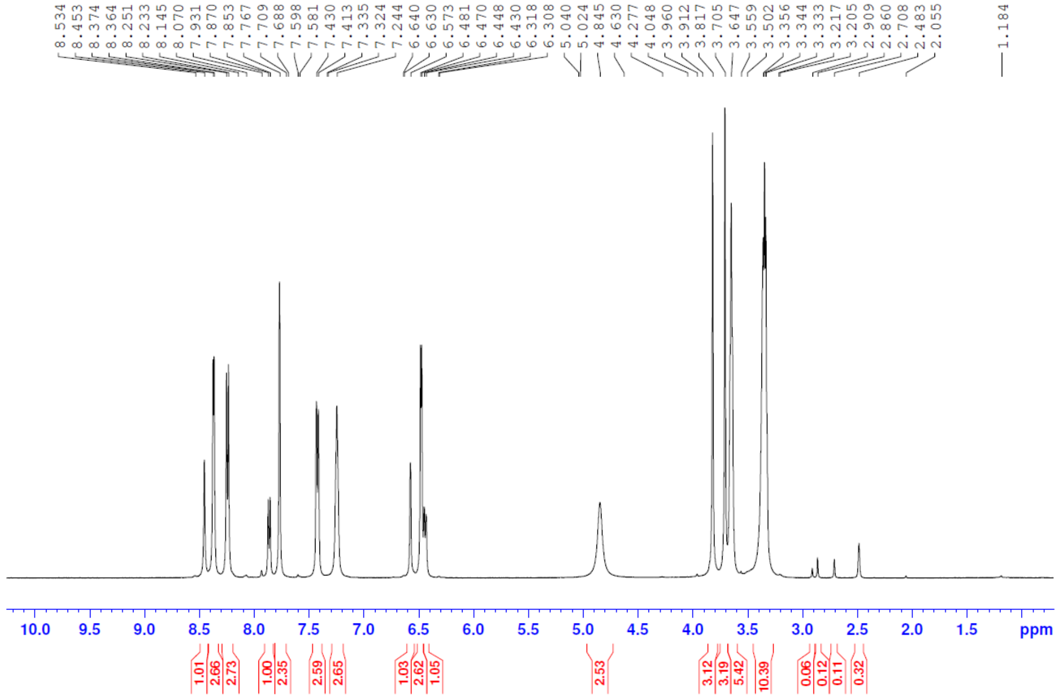

RB1936_12YP
13C en DMSO (9.7 mg)

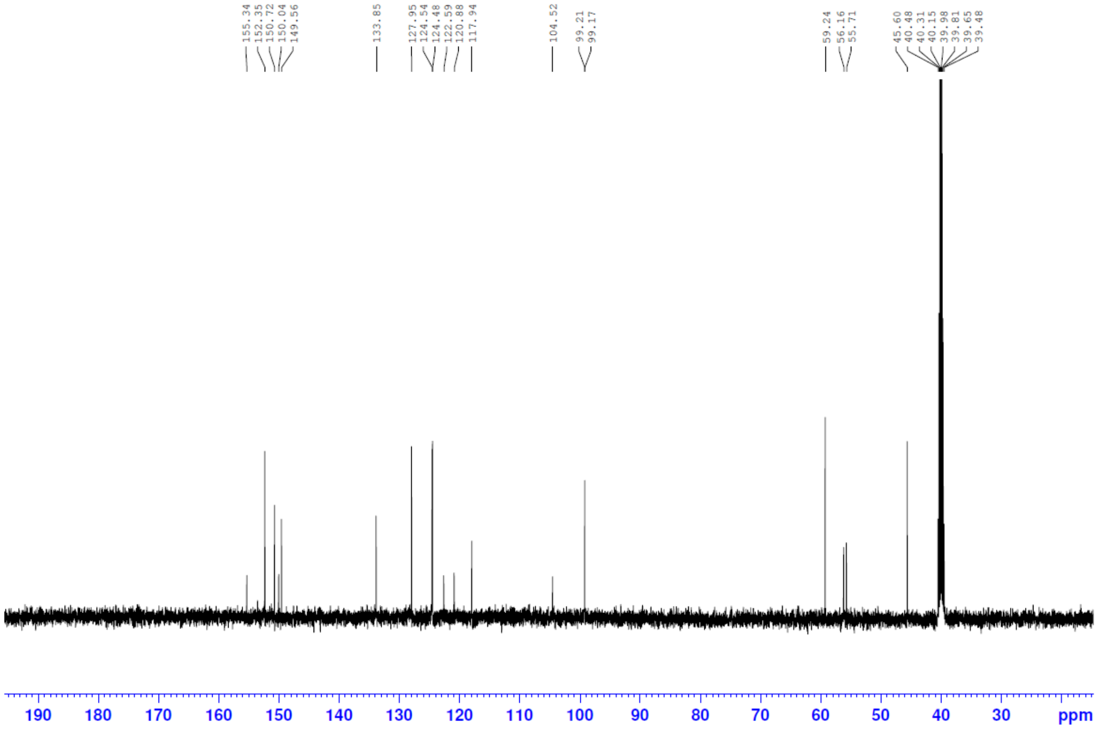

RB1936_12YP
DEPT en DMSO (9.7 mg)

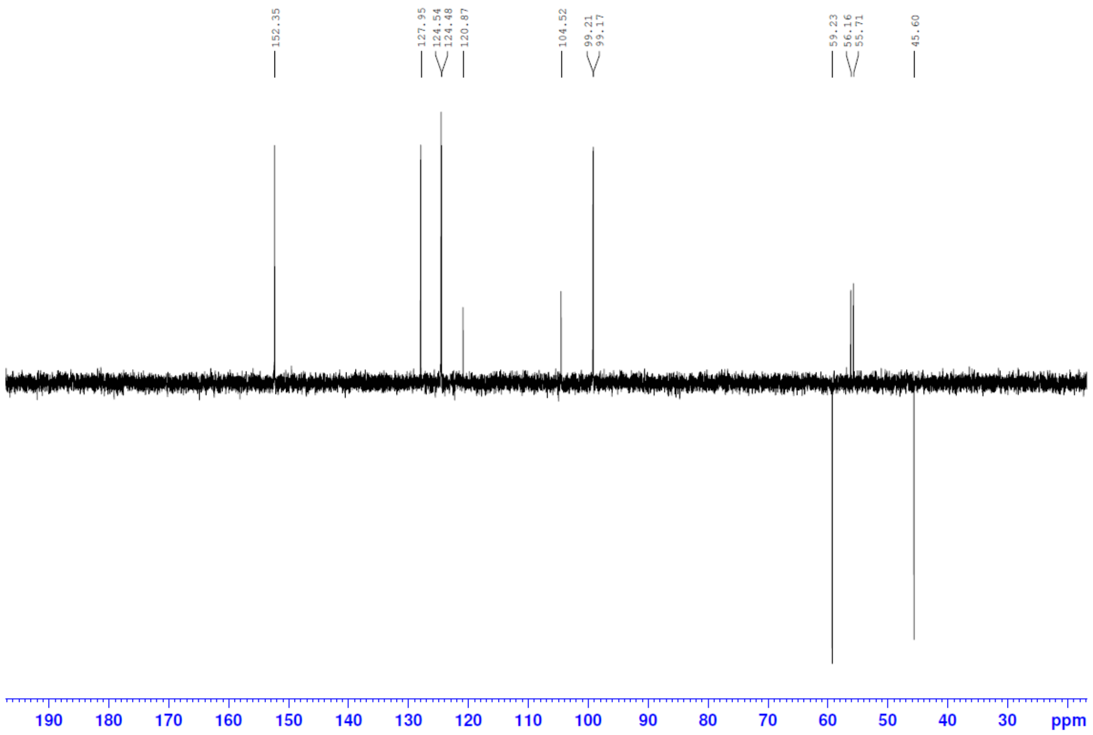

RB1936_12YP
COSY en DMSO (9.7 mg)

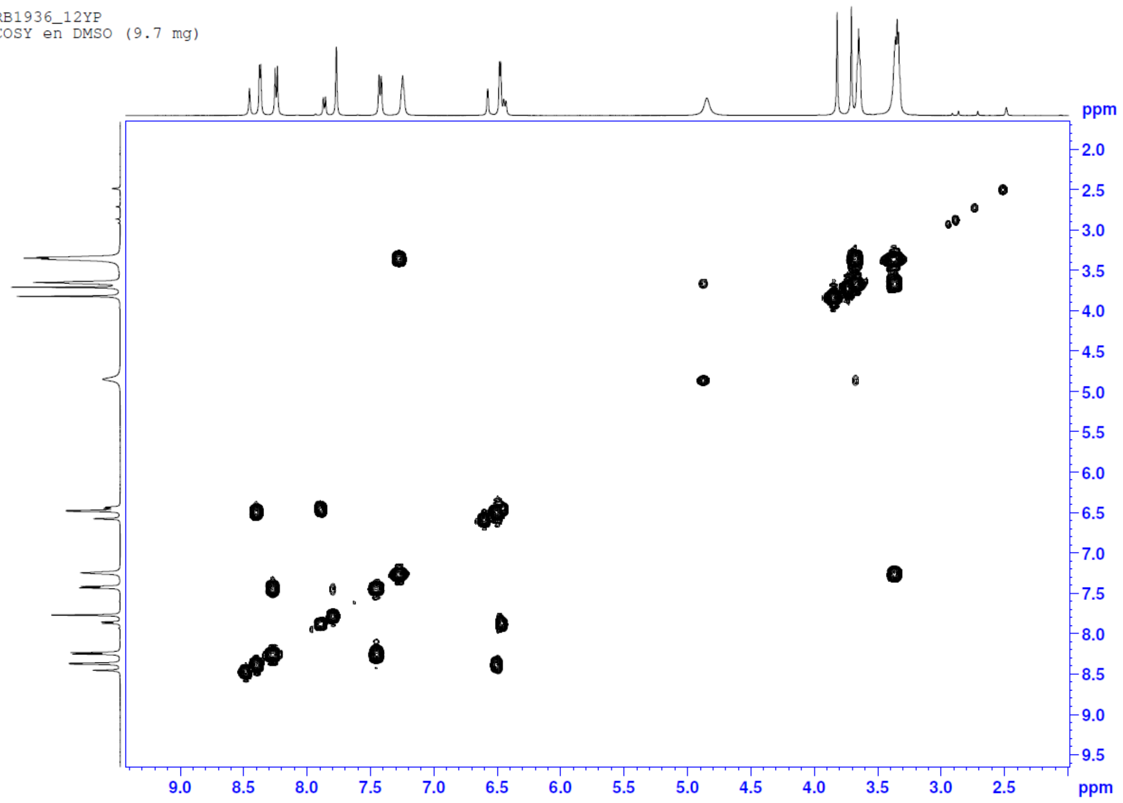

RB1936_12YP
HMQC en DMSO (9.7 mg)

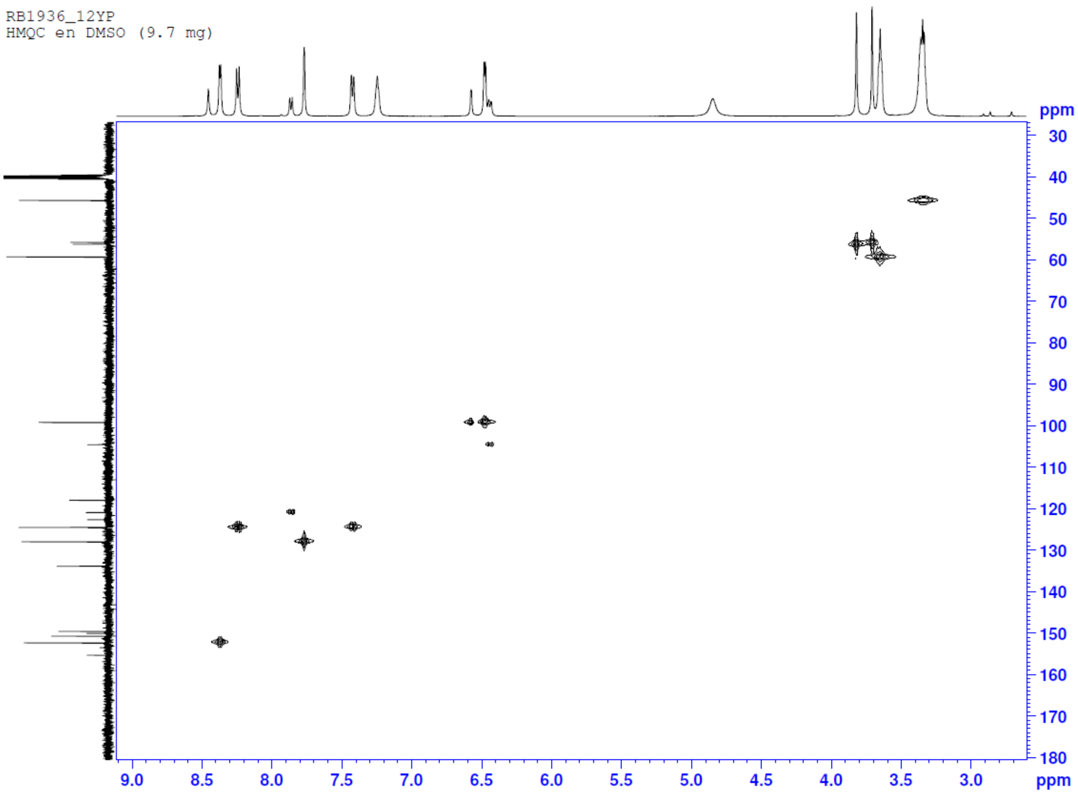

RB1936_12YP
HMBC en DMSO (9.7 mg)

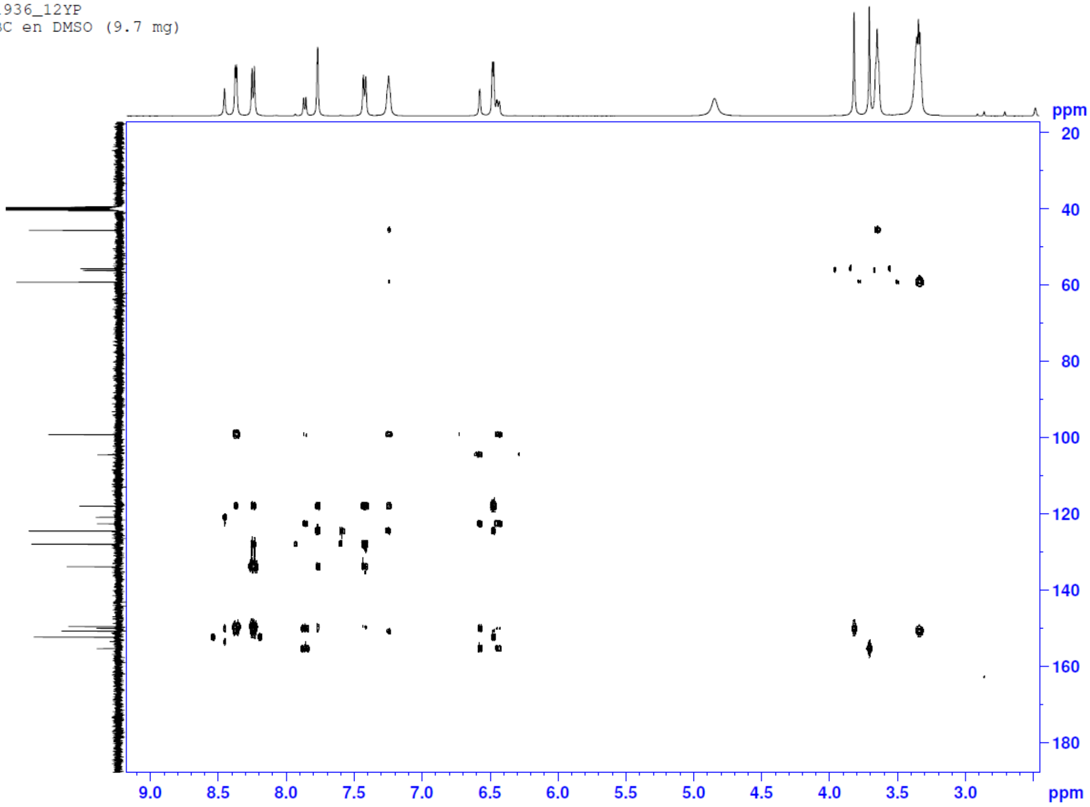

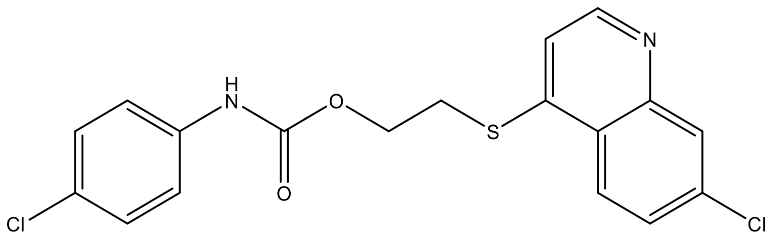

H
N
O
O
S
N
Cl
Cl

21

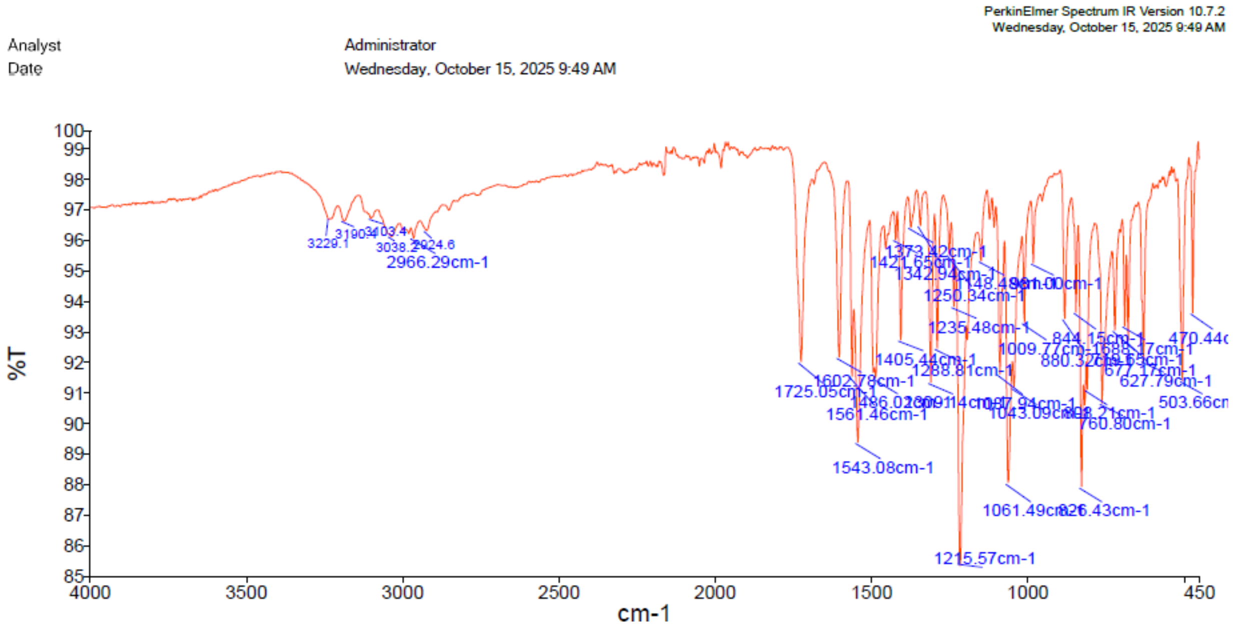

PerkinElmer Spectrum IR Version 10.7.2
Wednesday, October 15, 2025 9:49 AM
Analyst
Administrator
Date
Wednesday, October 15, 2025 9:49 AM
%T
cm-1
2966.29cm-1
1725.05cm-1
1602.78cm-1
1561.46cm-1
1543.08cm-1
1405.44cm-1
1288.81cm-1
1250.34cm-1
1235.48cm-1
1215.57cm-1
1009.77cm-1
880.32cm-1
844.15cm-1
826.43cm-1
760.80cm-1
677.17cm-1
627.79cm-1
503.66cm

RB1937_13YP
1H en DMSO (8.9 mg)

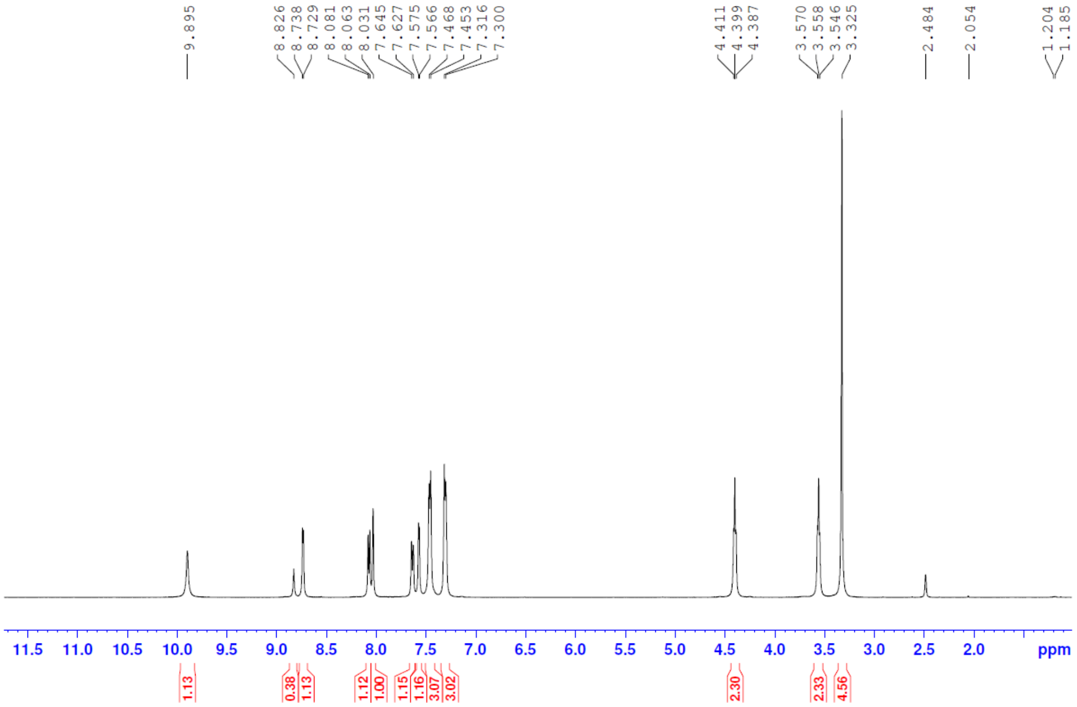

RB1937_13YP
13C en DMSO (8.9 mg)

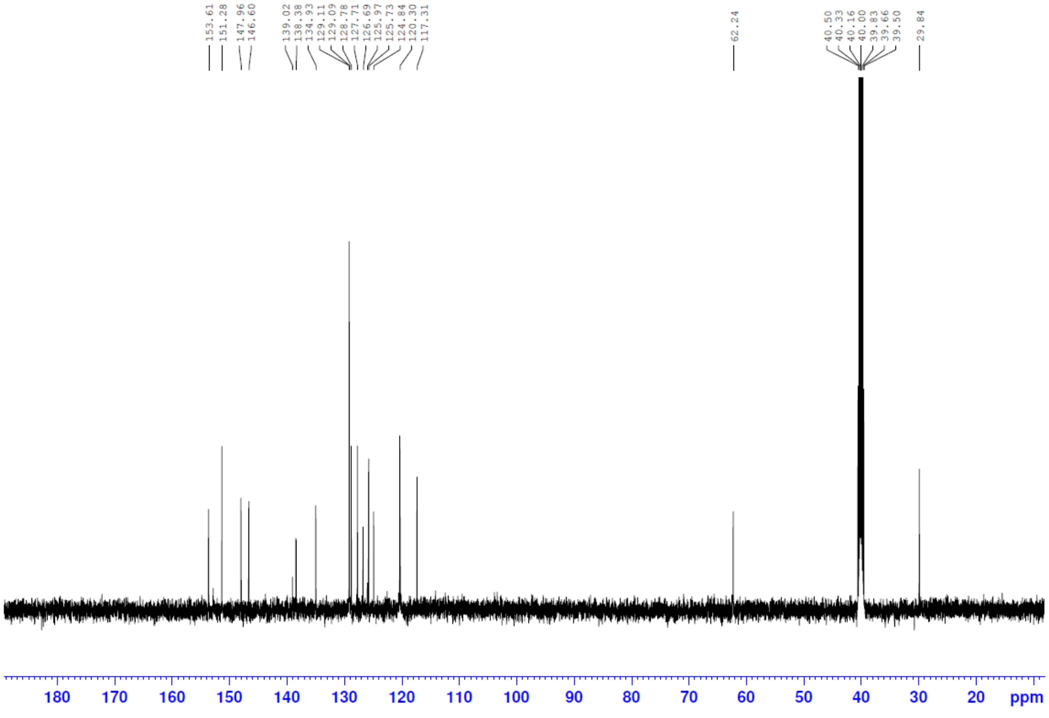

RB1937_13YP
DEPT en DMSO (8.9 mg)

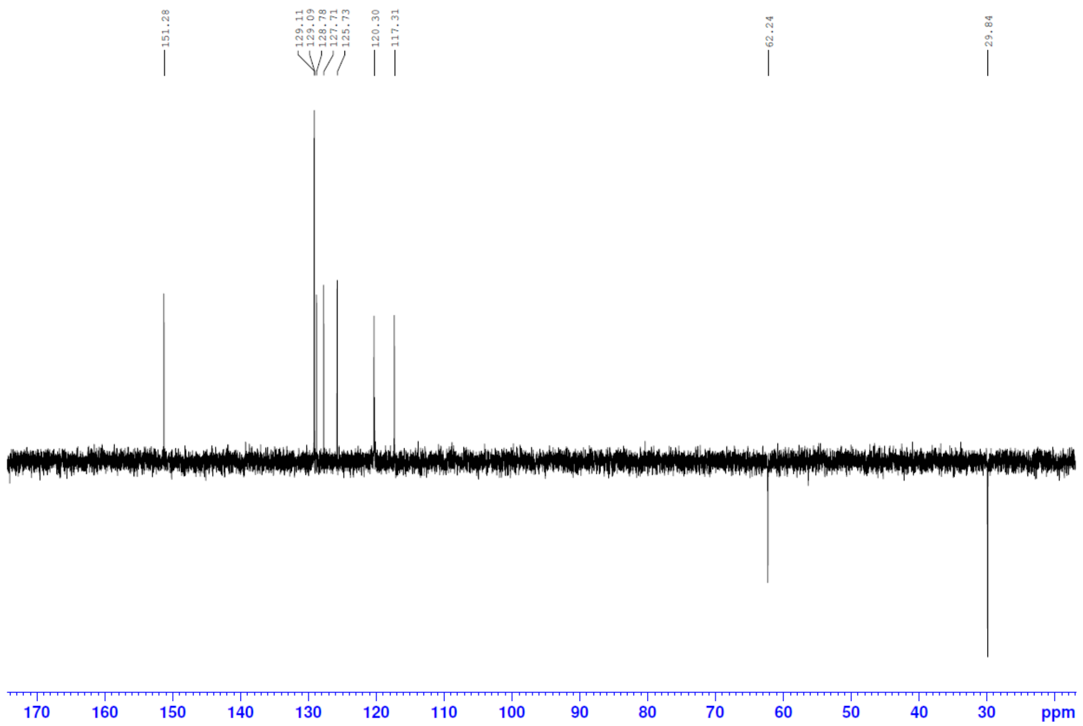

RB1937_13YP
COSY en DMSO (8.9 mg)

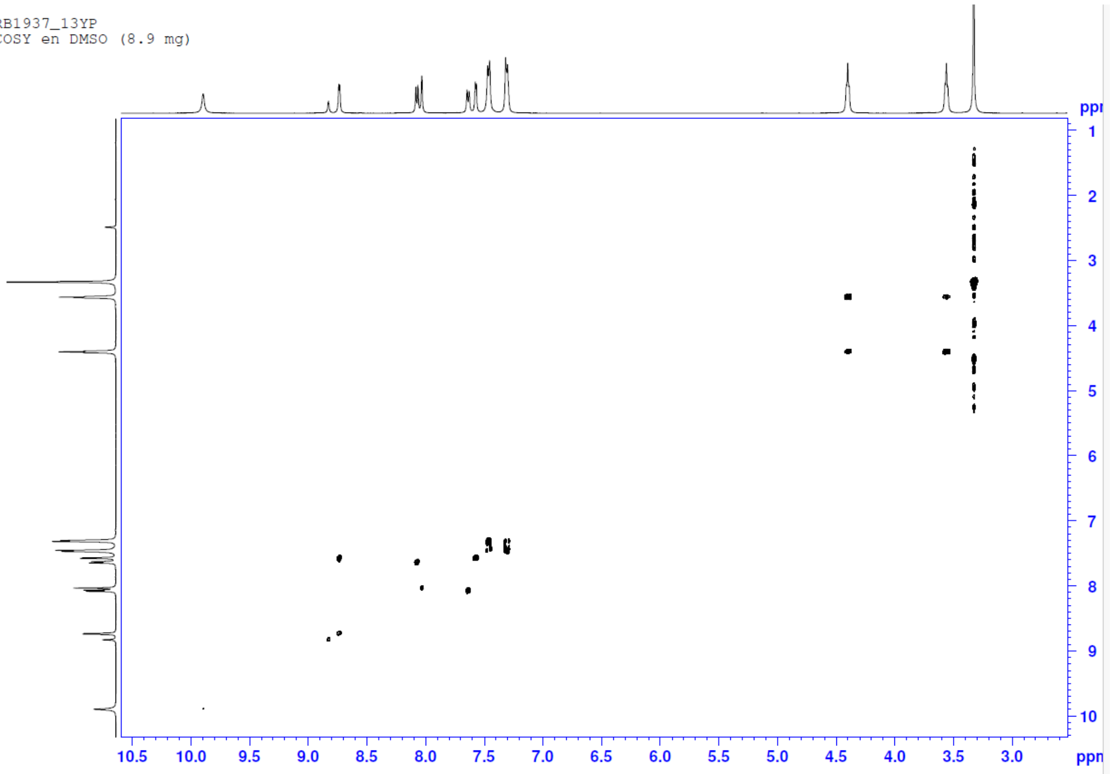

RB1937_13YP
HMQC en DMSO (8.9 mg)

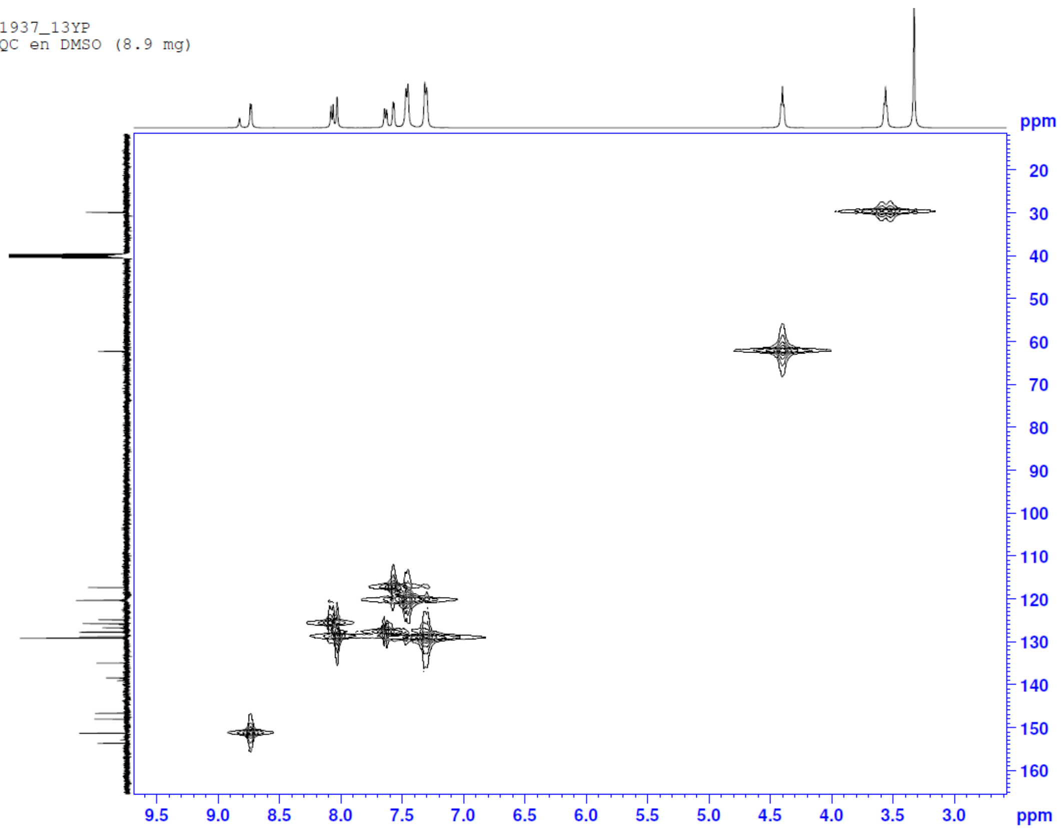

RB1937_13YP
HMBC en DMSO (8.9 mg)

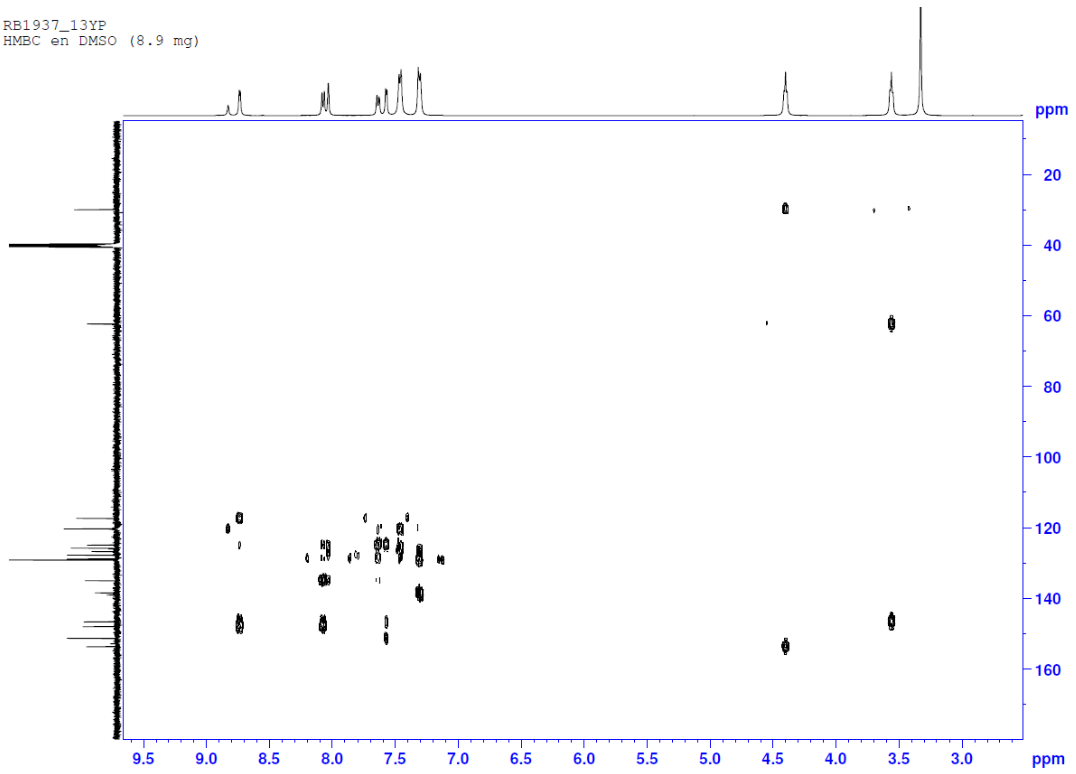

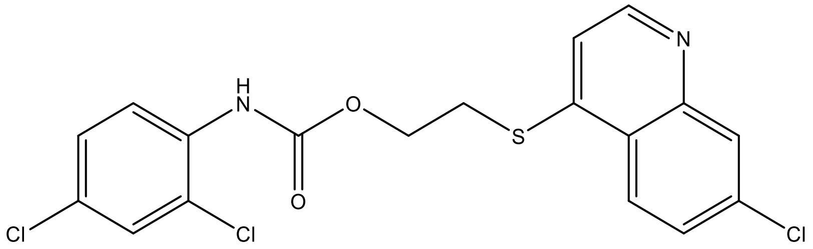

22

PerkinElmer Spectrum IR Version 10.7.2
Wednesday, October 15, 2025 9:52 AM

Analyst Administrator
Date Wednesday, October 15, 2025 9:52 AM

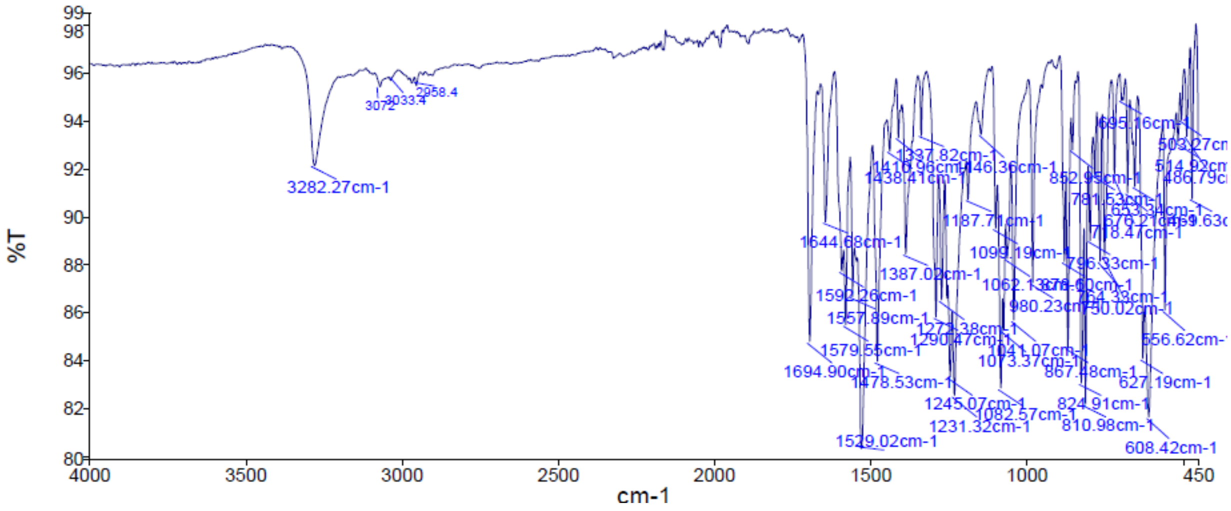

RB1938_14YP
1H en DMSO (9.1 mg)

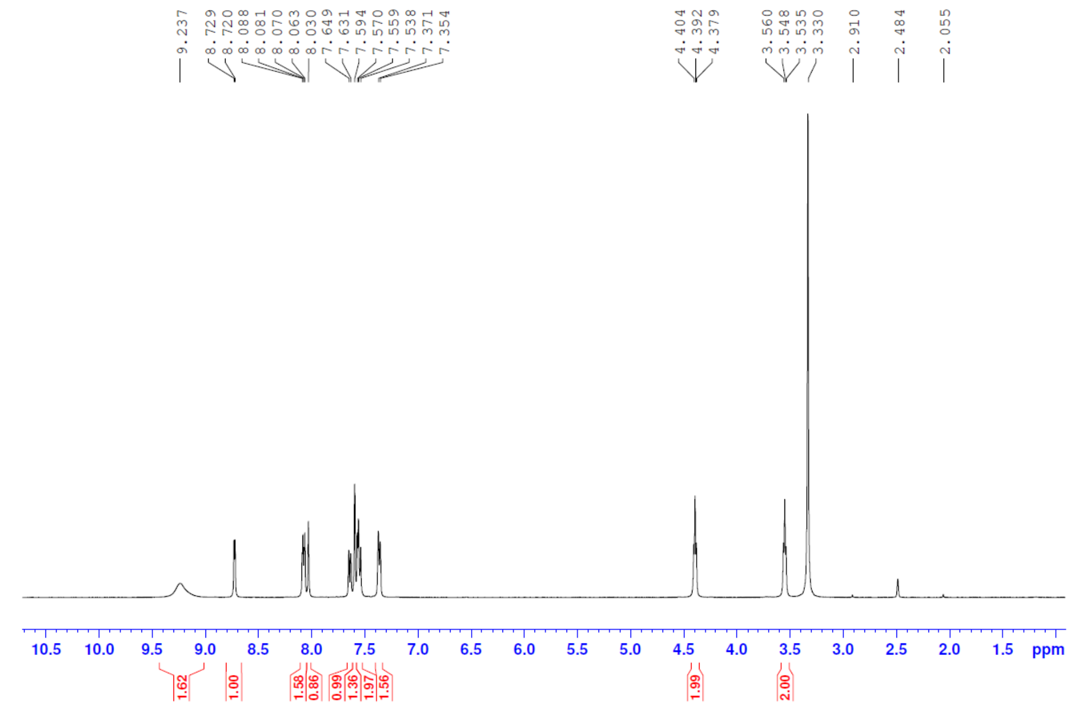

RB1938_14YP
13C en DMSO (9.1 mg)

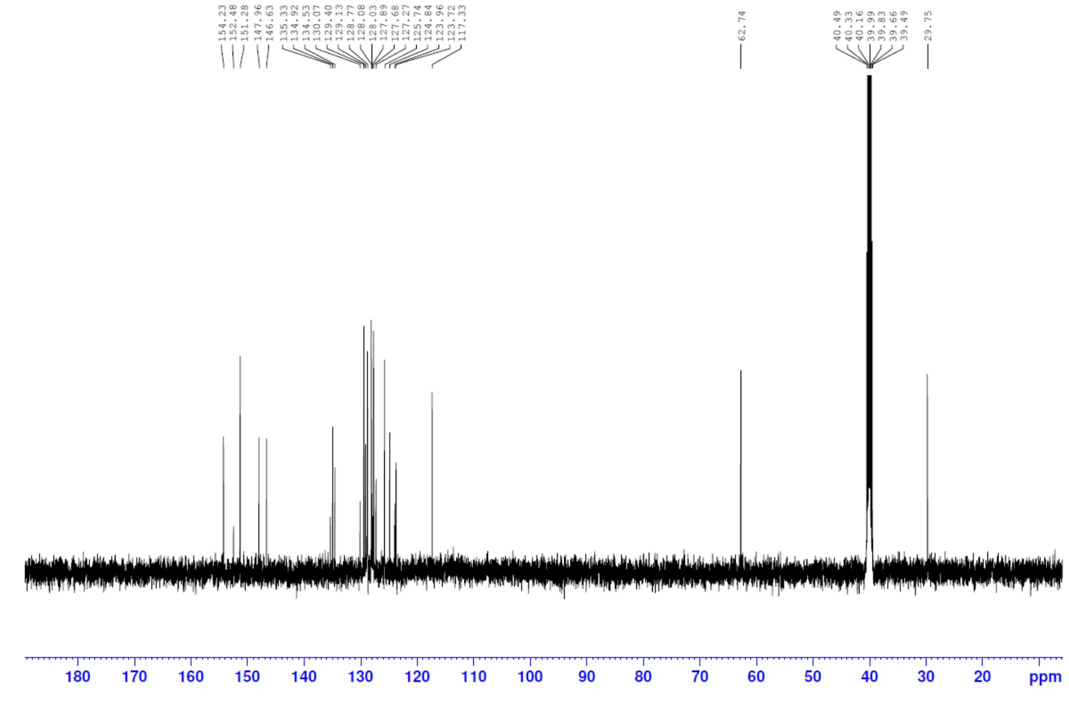

RB1938_14YP
DEPT en DMSO (9.1 mg)

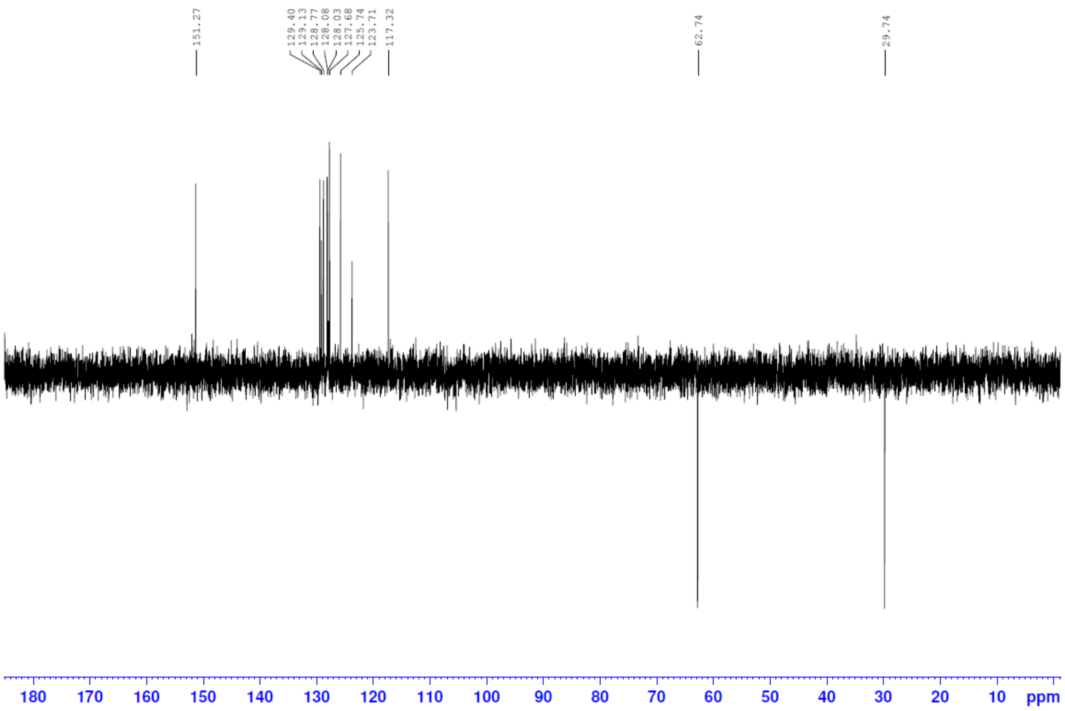

RB1938_14YP
COSY en DMSO (9.1 mg)

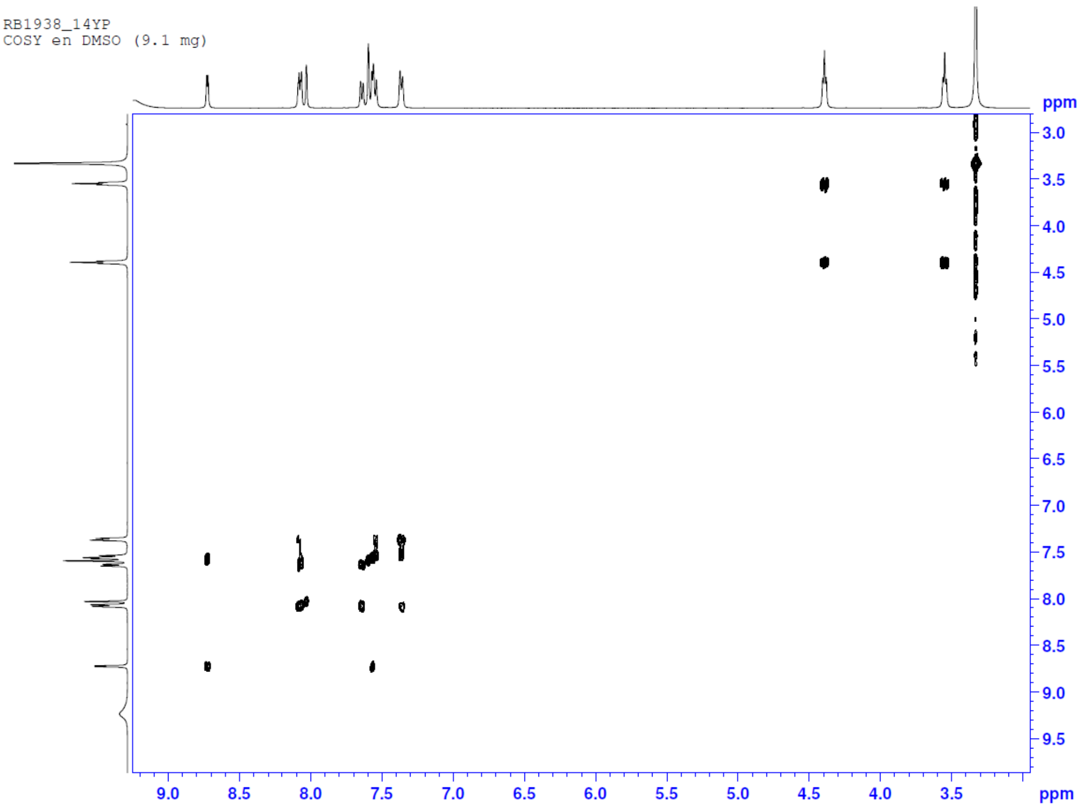

RB1938_14YP
HMQC en DMSO (9.1 mg)

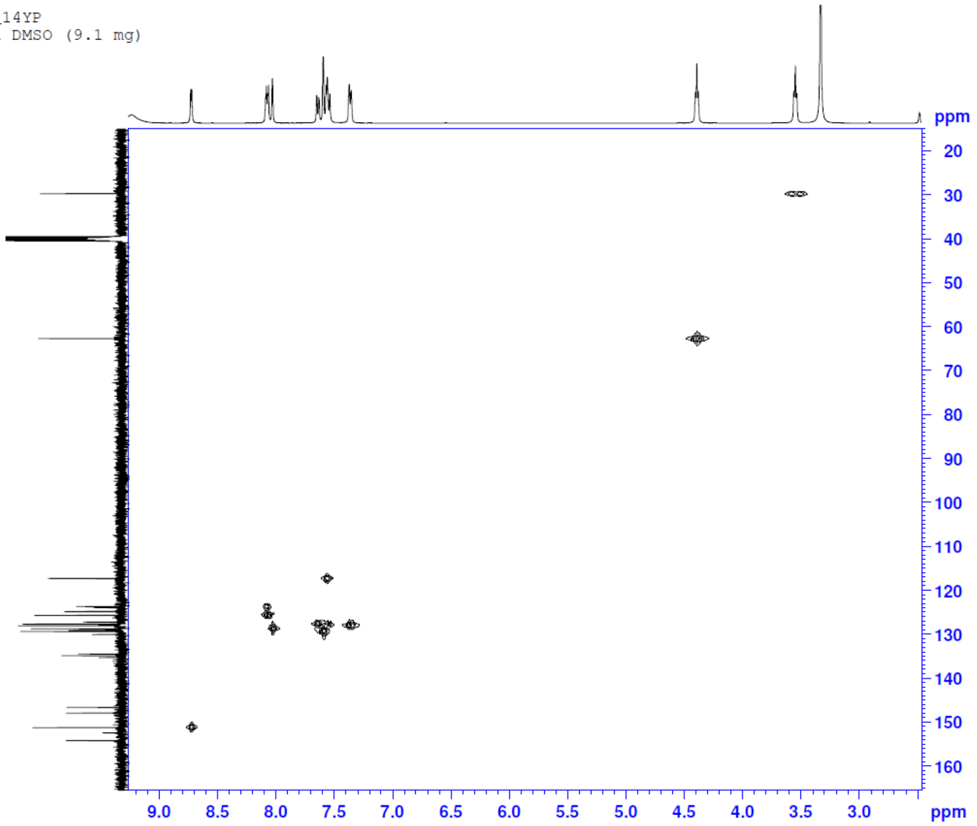

RB1938_14YP
HMBC en DMSO (9.1 mg)

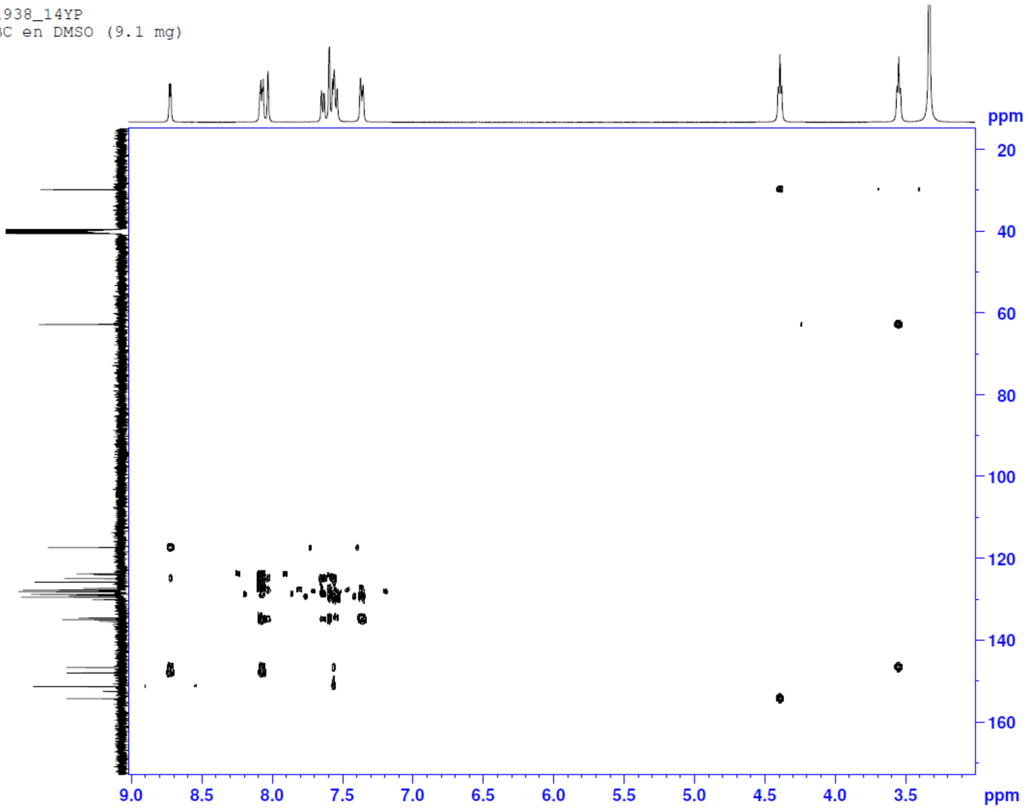

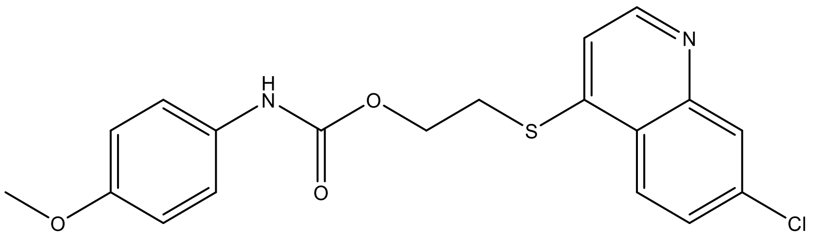
H
N
O
O
S
N
O
Cl

23

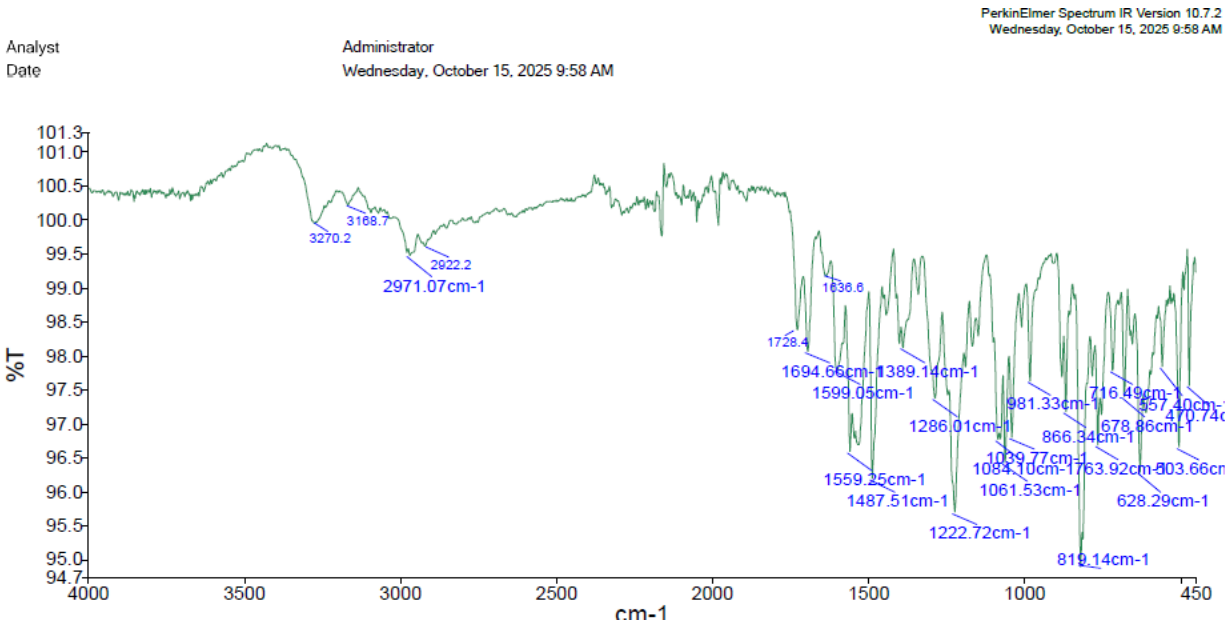
PerkinElmer Spectrum IR Version 10.7.2
Wednesday, October 15, 2025 9:58 AM
Analyst
Administrator
Date
Wednesday, October 15, 2025 9:58 AM
%T
cm-1
3270.2
3168.7
2922.2
2971.07cm-1
1728.4
1636.6
1694.66cm-1
1389.14cm-1
1599.05cm-1
1286.01cm-1
1559.25cm-1
1487.51cm-1
1222.72cm-1
981.33cm-1
866.34cm-1
1039.77cm-1
1084.10cm-1
1061.53cm-1
819.14cm-1
716.49cm-1
678.86cm-1
628.29cm-1

RB1943_19YP
1H en DMSO (5,8 mg)

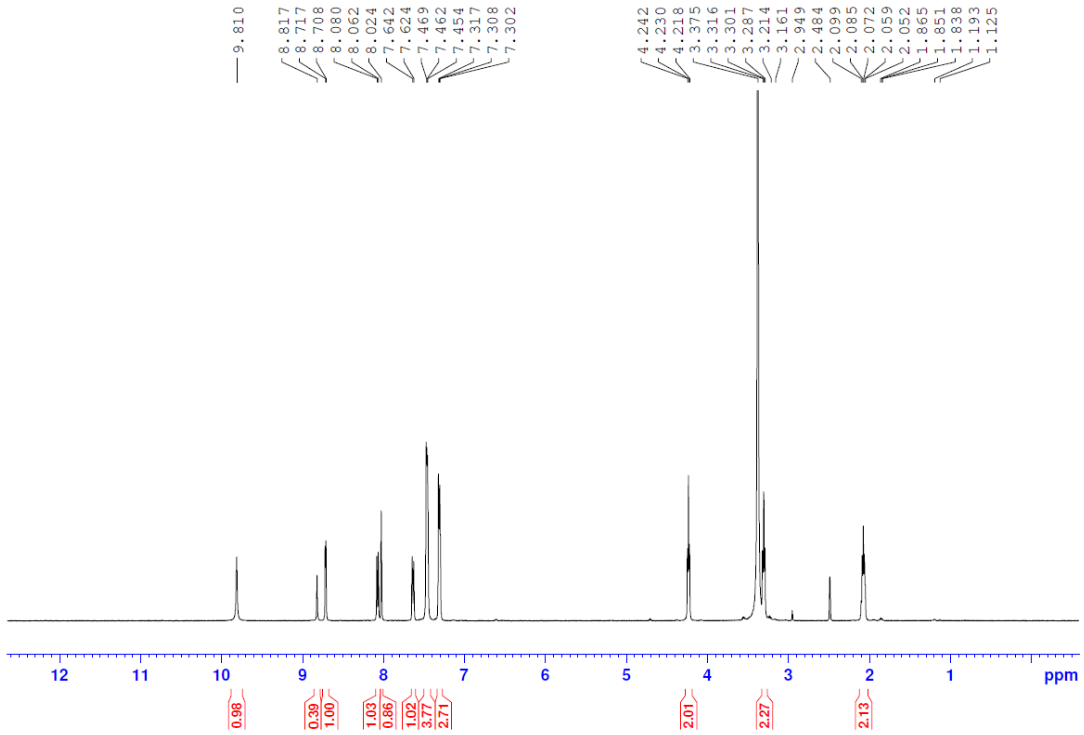

RB1943_19YP
13C en DMSO (5,8 mg)

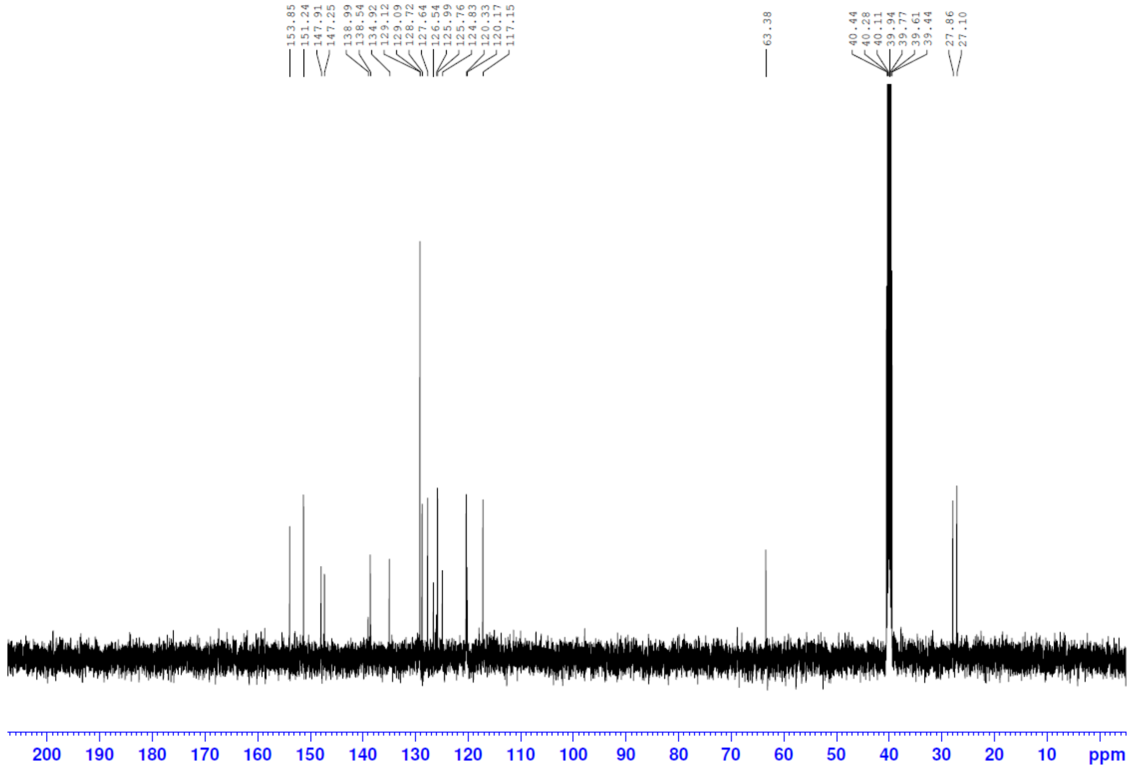

RB1943_19YP
DEPT en DMSO (5,8 mg)

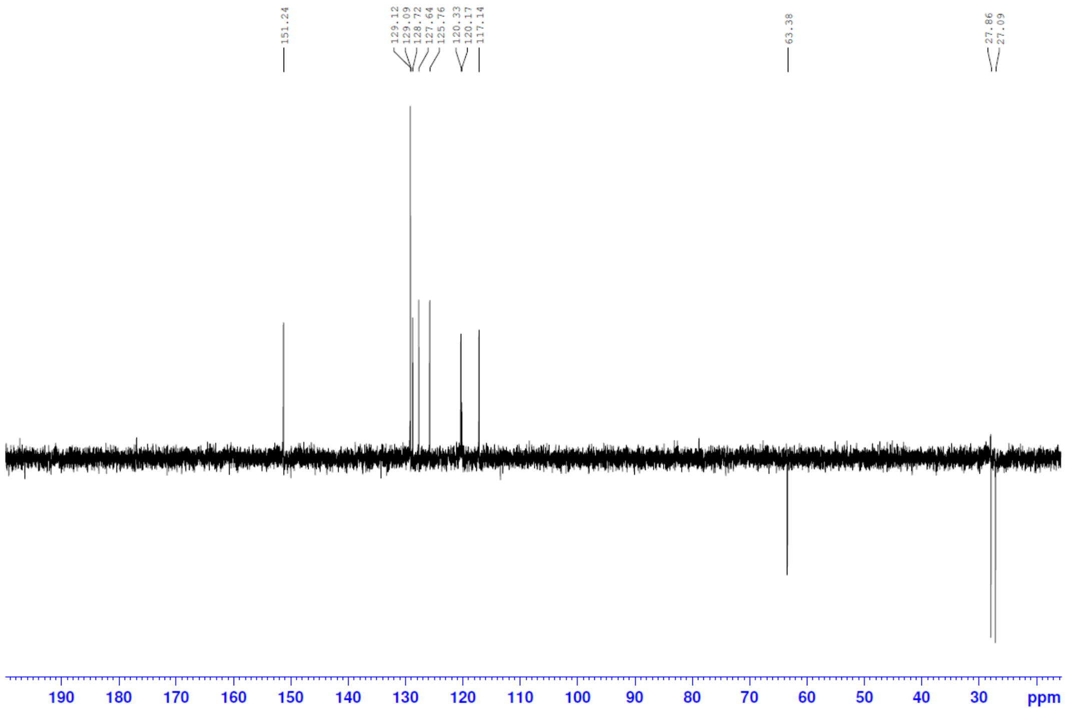

RB1943_19YP
COSY en DMSO (5,8 mg)

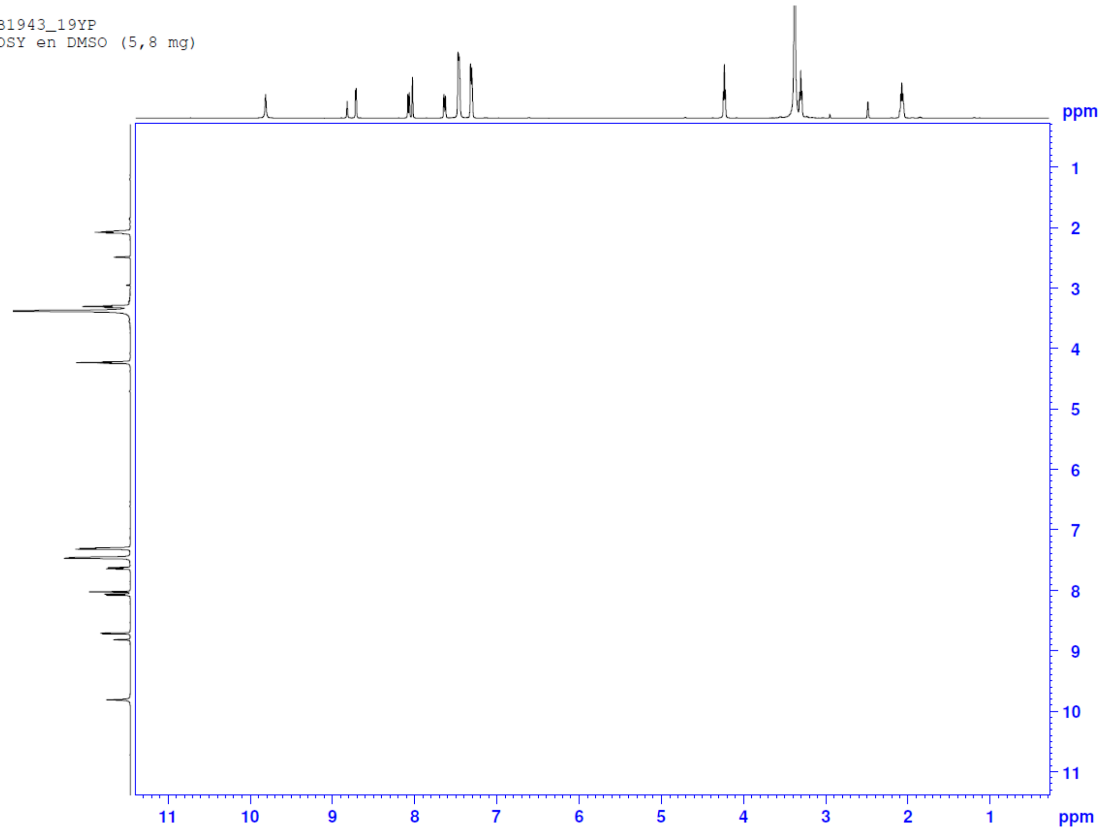

RB1943_19YP
HMQC en DMSO (5,8 mg)

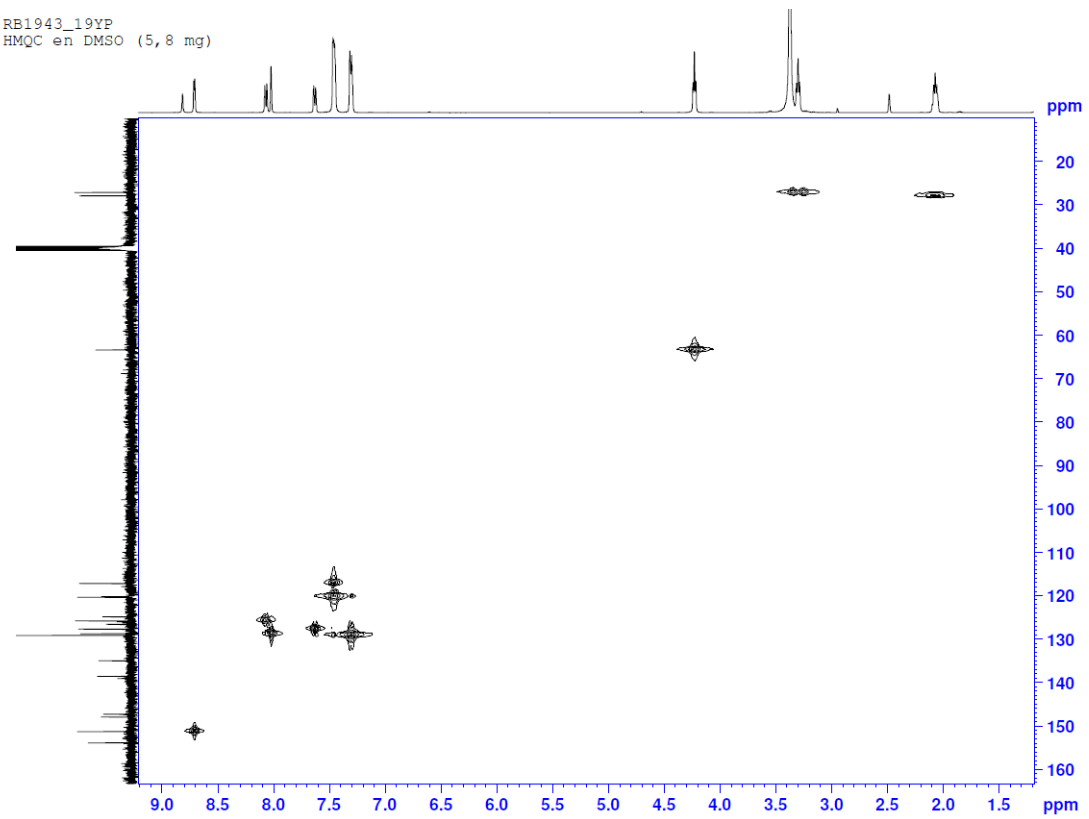

RB1943_19YP
HMBC en DMSO (5,8 mg)

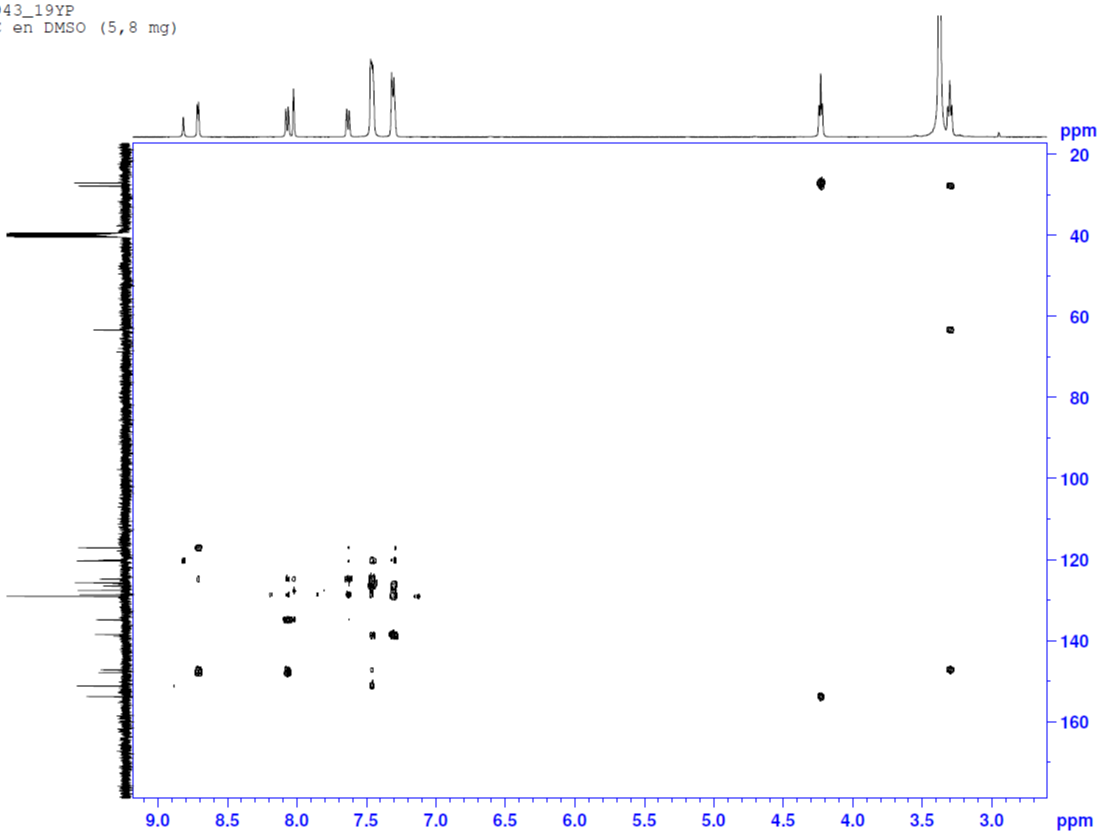

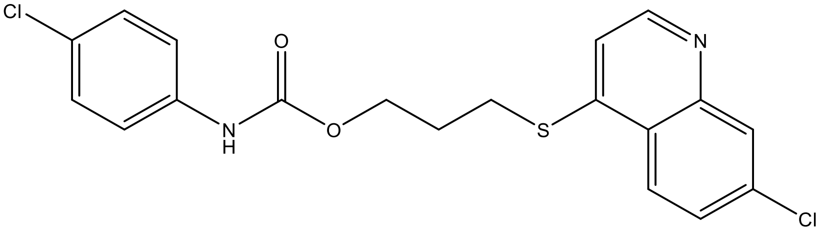
Cl
O
N
H
O
S
N
Cl

24

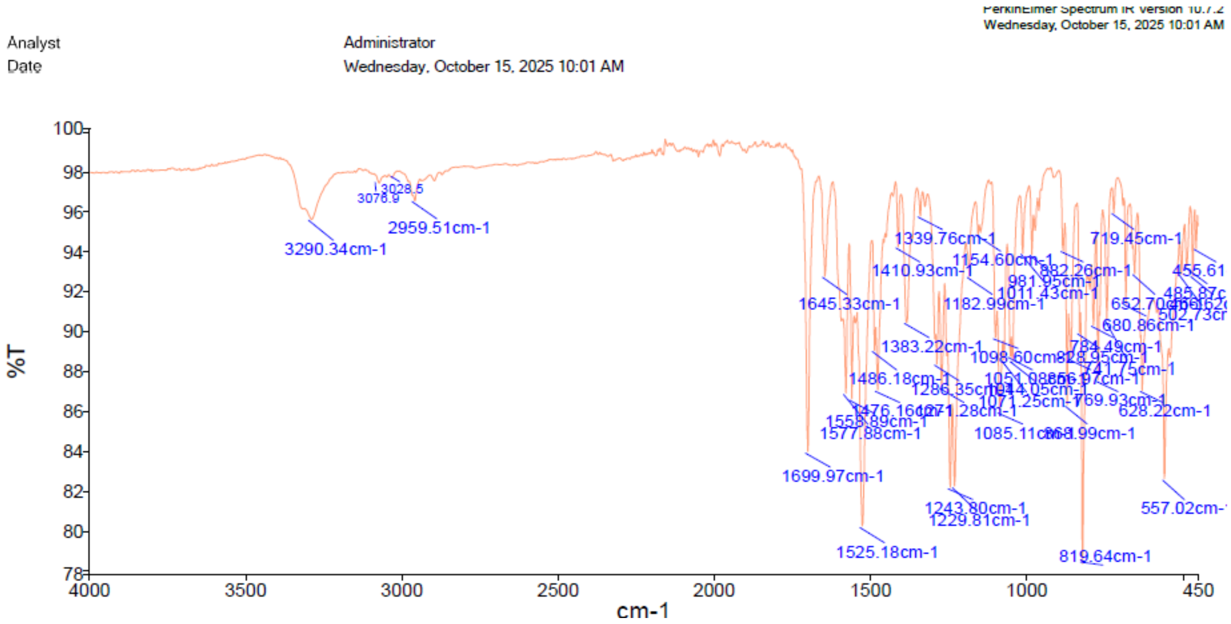
PerkinElmer Spectrum IR Version 10.7.2
Wednesday, October 15, 2025 10:01 AM
Analyst
Administrator
Date
Wednesday, October 15, 2025 10:01 AM
%T
cm-1
3290.34cm-1
2959.51cm-1
1699.97cm-1
1645.33cm-1
1525.18cm-1
1577.88cm-1
1486.18cm-1
1410.93cm-1
1383.22cm-1
1339.76cm-1
1243.80cm-1
1229.81cm-1
1182.99cm-1
1154.60cm-1
1085.11cm-1
1011.43cm-1
981.95cm-1
882.26cm-1
819.64cm-1
719.45cm-1
680.86cm-1
652.70cm-1
628.22cm-1
557.02cm-1

RB1944_20YP
1H en DMSO (9.9 mg)

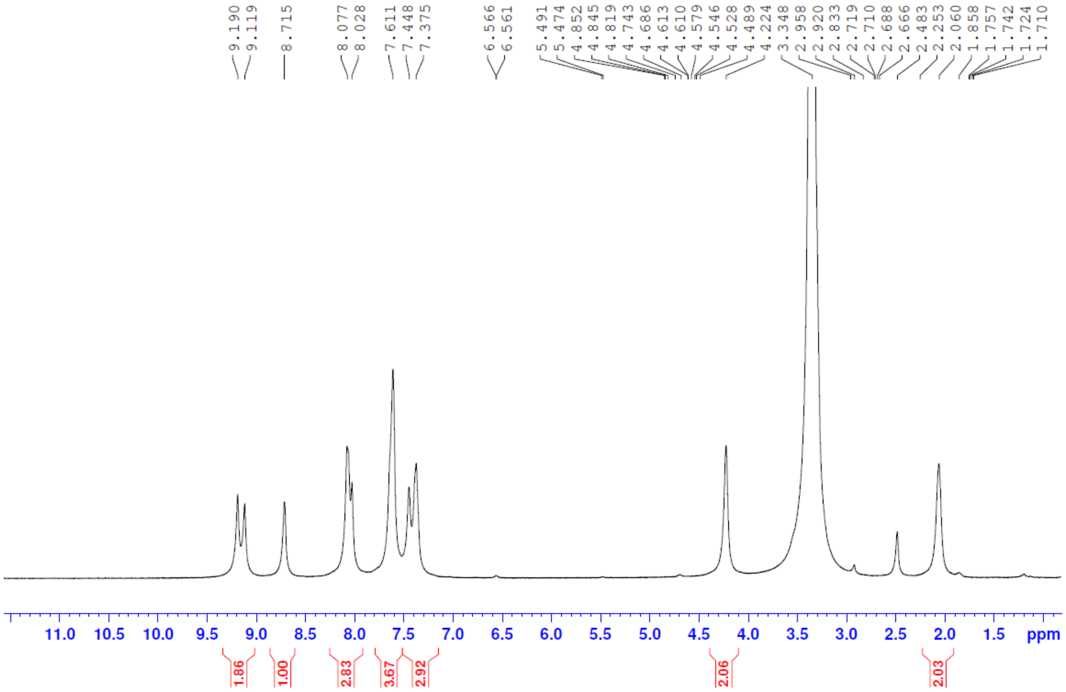

RB1944_20YP
13C en DMSO (9.9 mg)

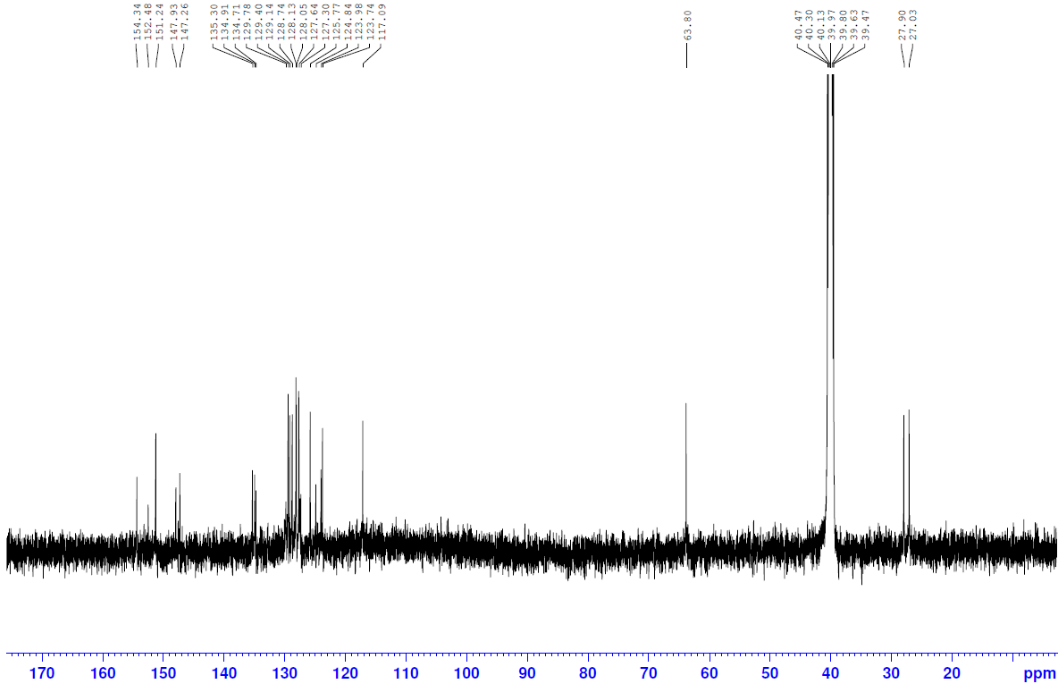

RB1944_20YP
DEPT en DMSO (9.9 mg)

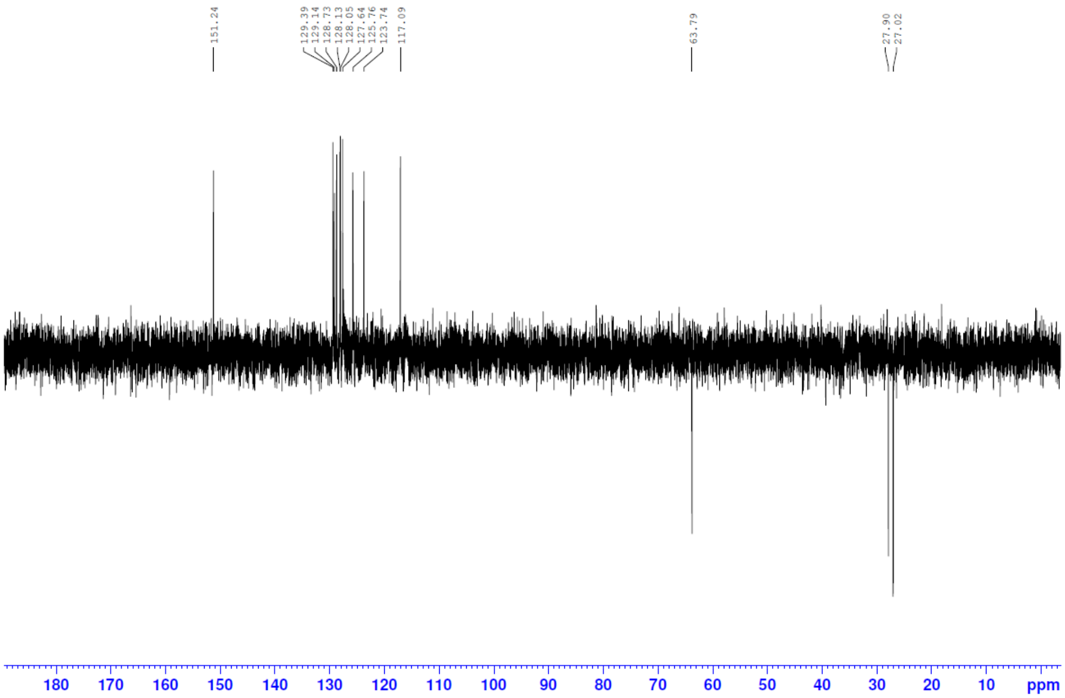

RB1944_20YP
COSY en DMSO (9.9 mg)

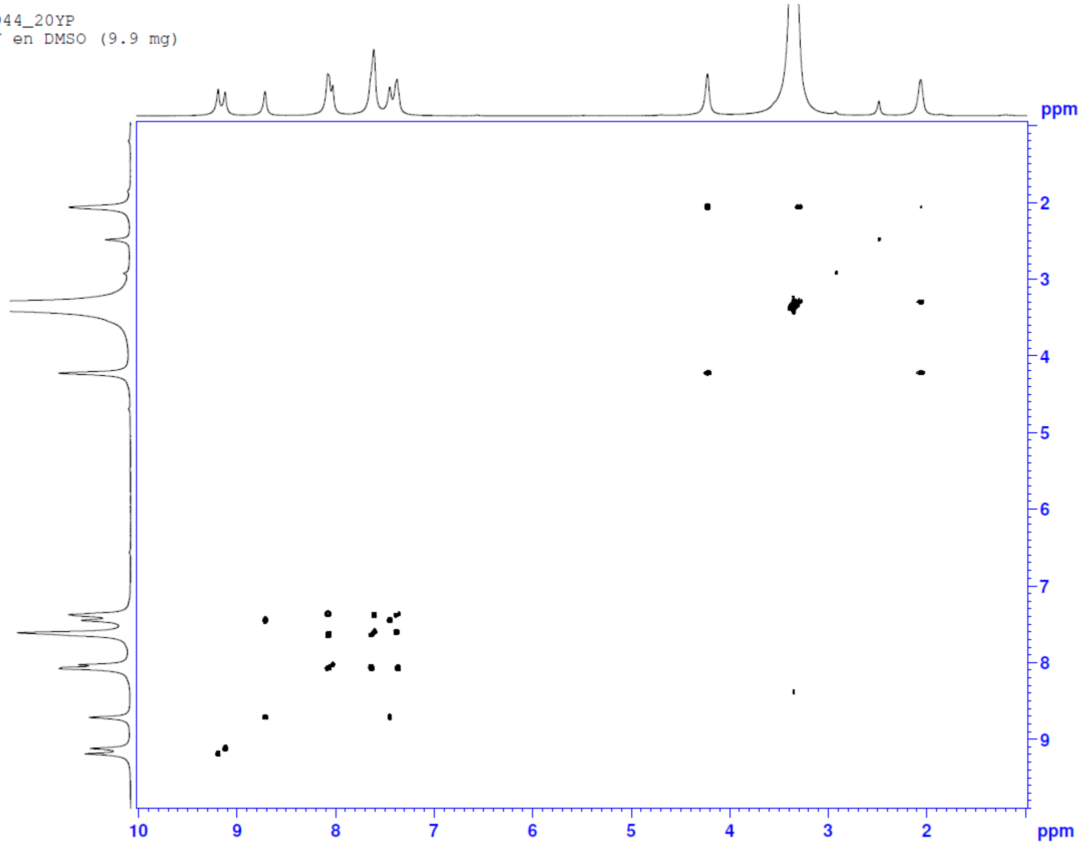

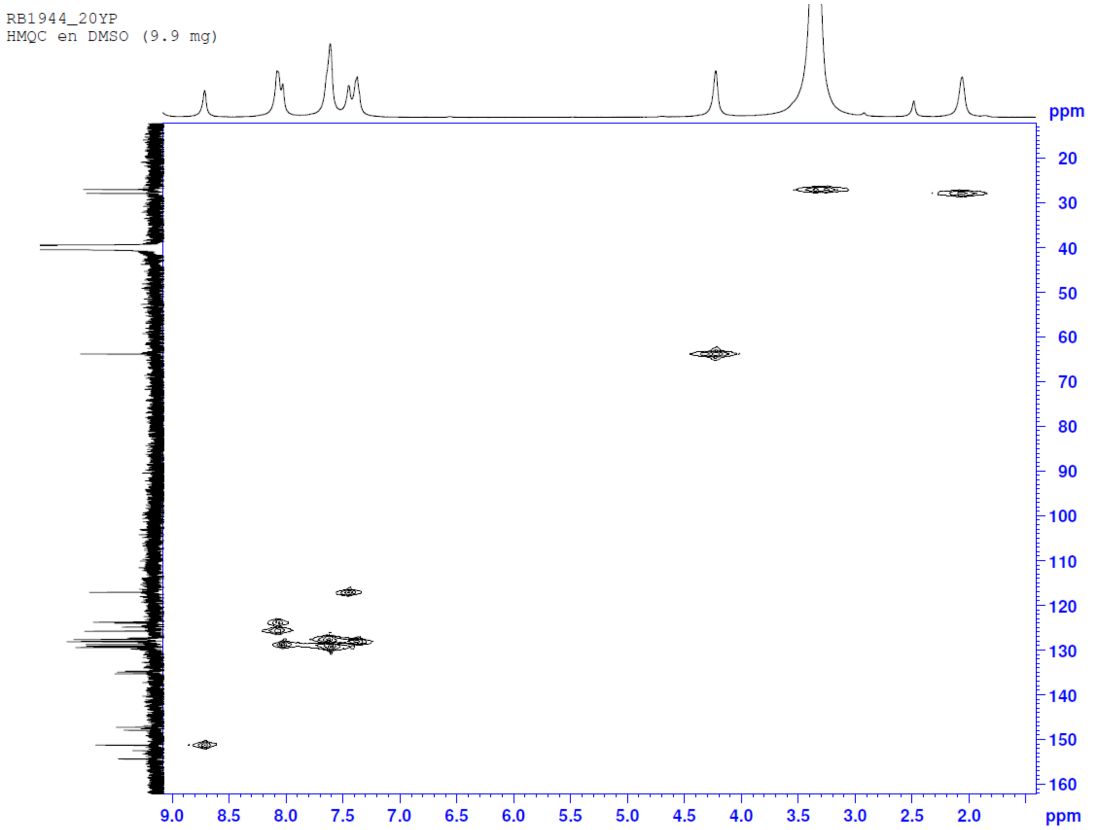
RB1944_20YP
HMQC en DMSO (9.9 mg)
ppm
20
30
40
50
60
70
80
90
100
110
120
130
140
150
160
9.0
8.5
8.0
7.5
7.0
6.5
6.0
5.5
5.0
4.5
4.0
3.5
3.0
2.5
2.0
ppm

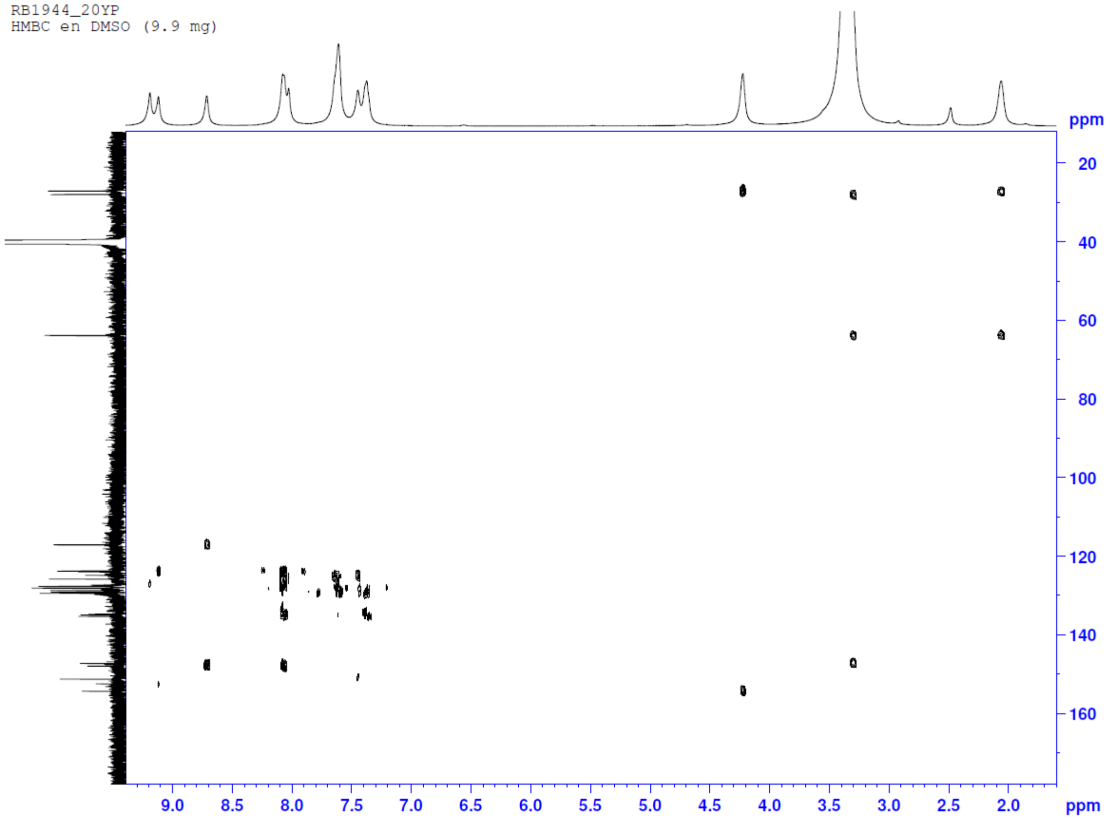
RB1944_20YP
HMBC en DMSO (9.9 mg)
ppm
20
40
60
80
100
120
140
160
9.0
8.5
8.0
7.5
7.0
6.5
6.0
5.5
5.0
4.5
4.0
3.5
3.0
2.5
2.0
ppm

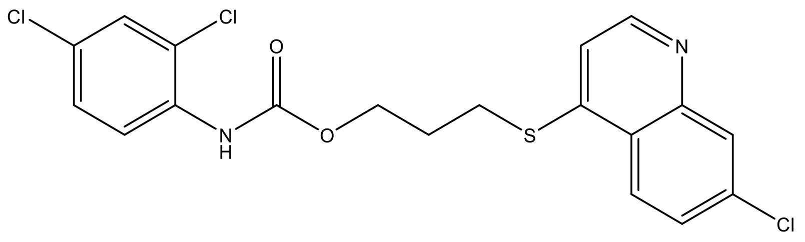
Cl
Cl
O
N
H
O
S
N
Cl

25

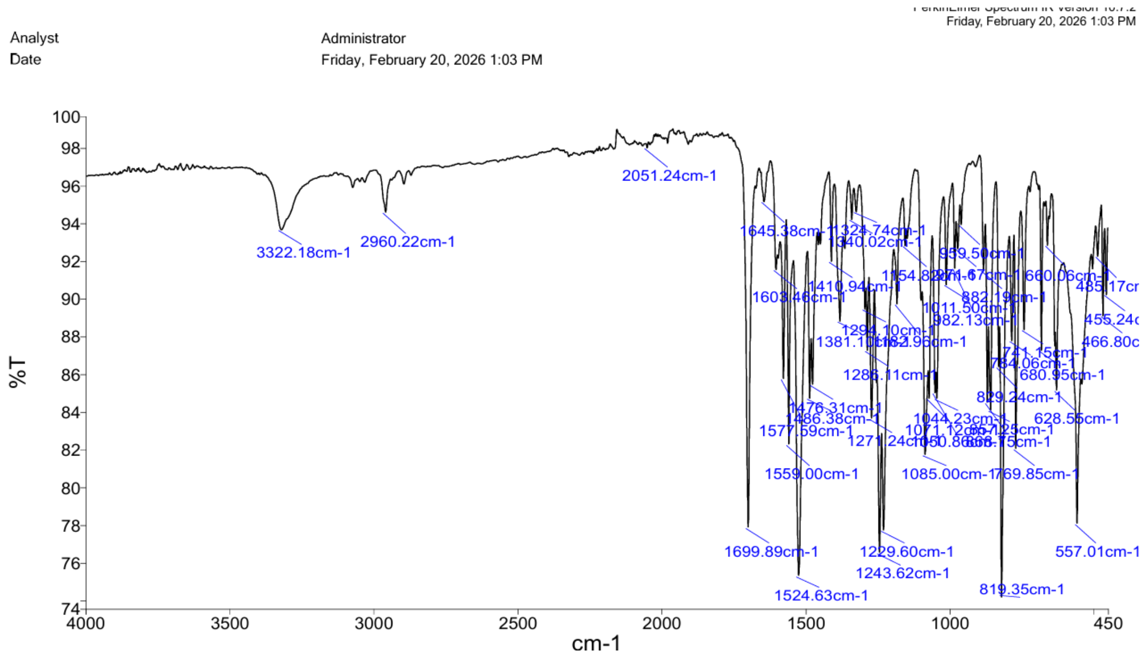
Friday, February 20, 2026 1:03 PM
Analyst
Date
Administrator
Friday, February 20, 2026 1:03 PM
%T
cm-1
3322.18cm-1
2960.22cm-1
2051.24cm-1
1645.38cm-1
1603.46cm-1
1577.59cm-1
1559.00cm-1
1699.89cm-1
1524.63cm-1
1476.31cm-1
1486.38cm-1
1410.94cm-1
1340.02cm-1
1294.10cm-1
1286.11cm-1
1229.60cm-1
1243.62cm-1
1085.00cm-1
1044.23cm-1
1011.50cm-1
959.50cm-1
982.13cm-1
882.19cm-1
829.24cm-1
819.35cm-1
741.15cm-1
680.95cm-1
660.06cm-1
628.55cm-1
557.01cm-1
466.80c
455.24c

RB1945_21YP
1H en DMSO

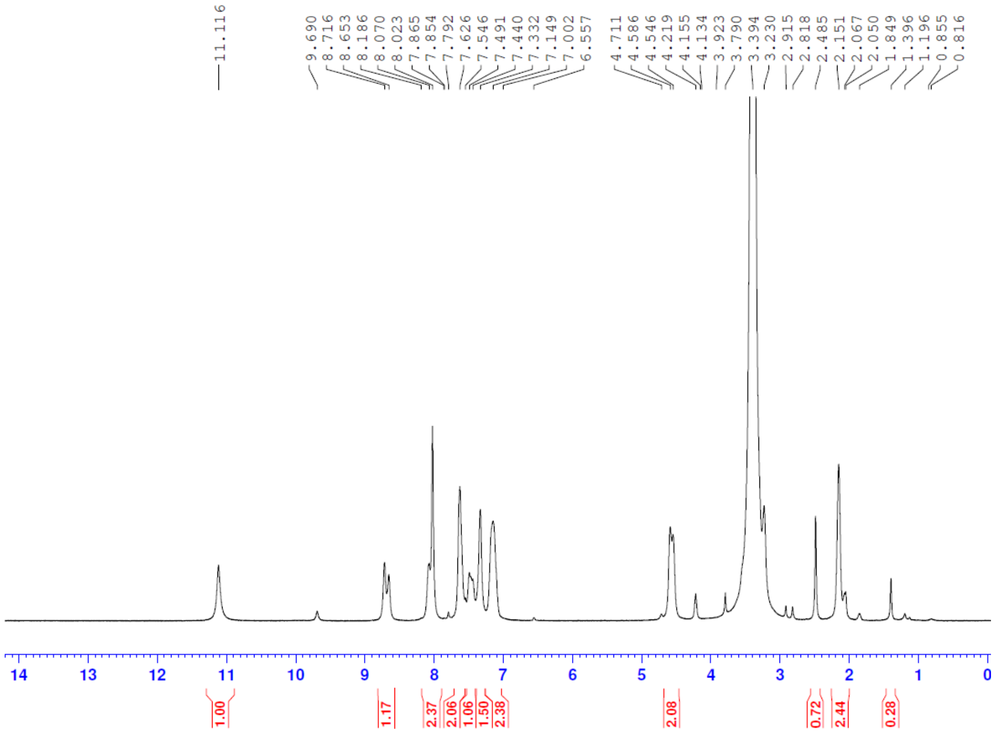

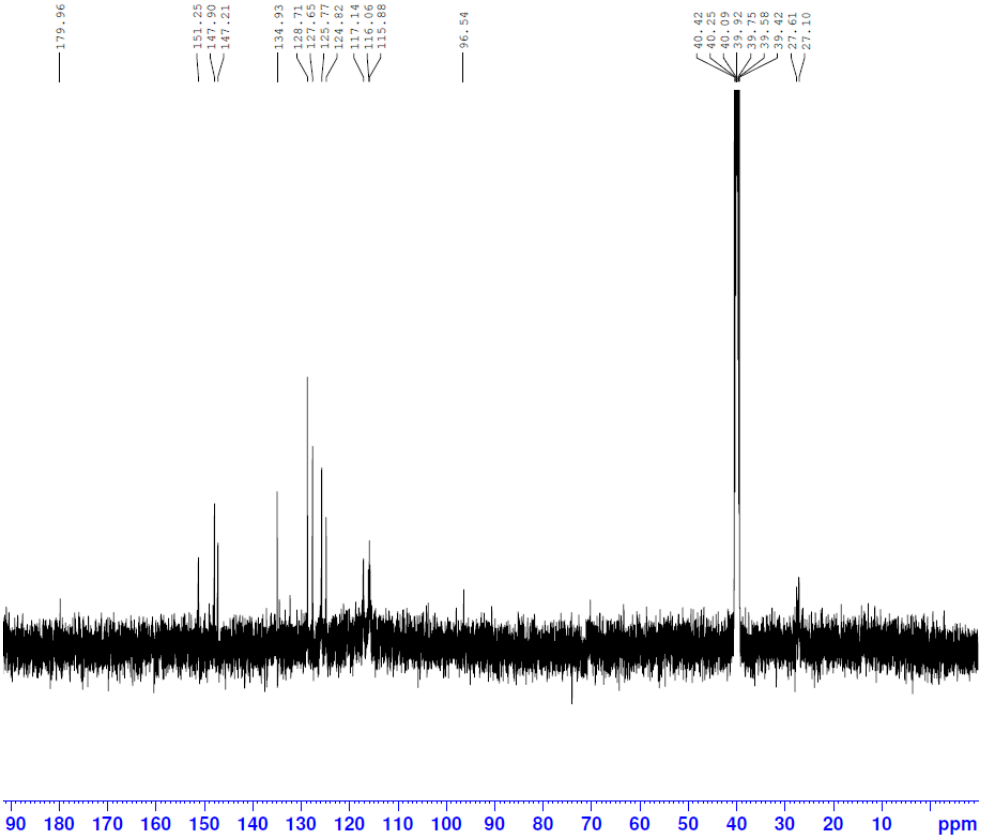

RB1945_21YP
DEPT en DMSO

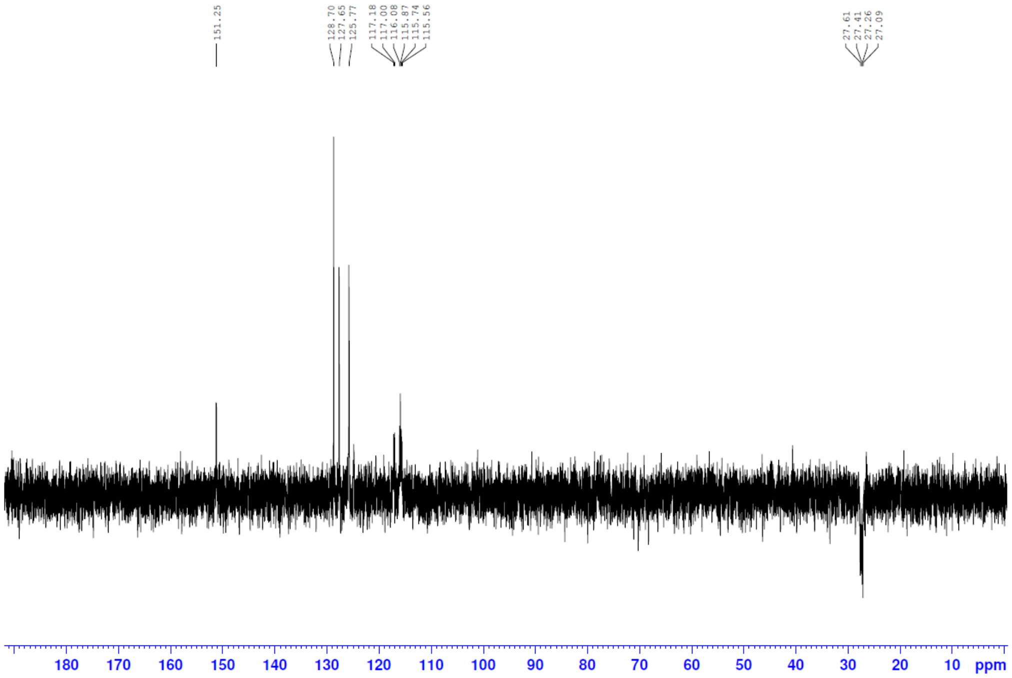

RB1945_21YP
COSY en DMSO

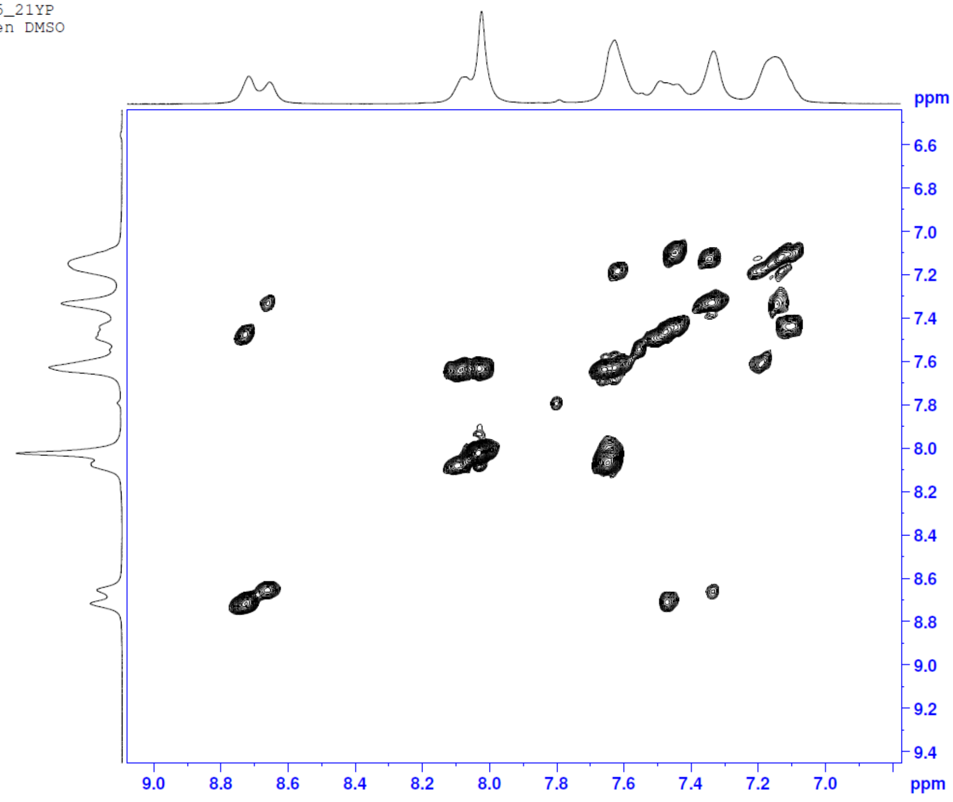

RB1945_21YP
HMQC en DMSO

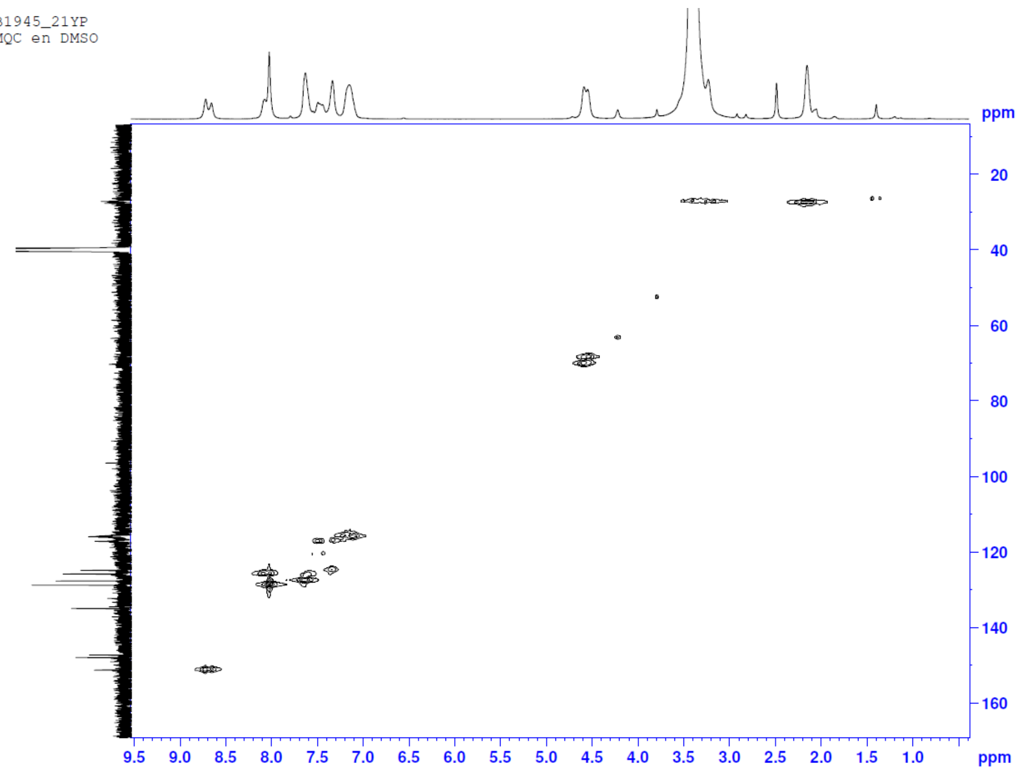

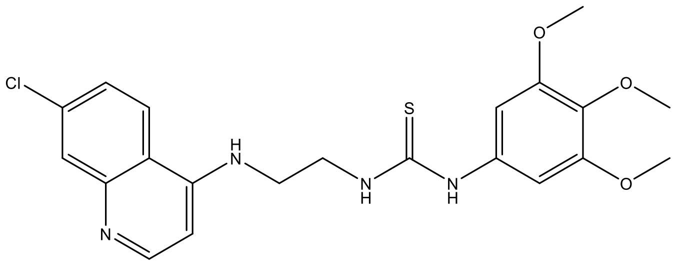

26

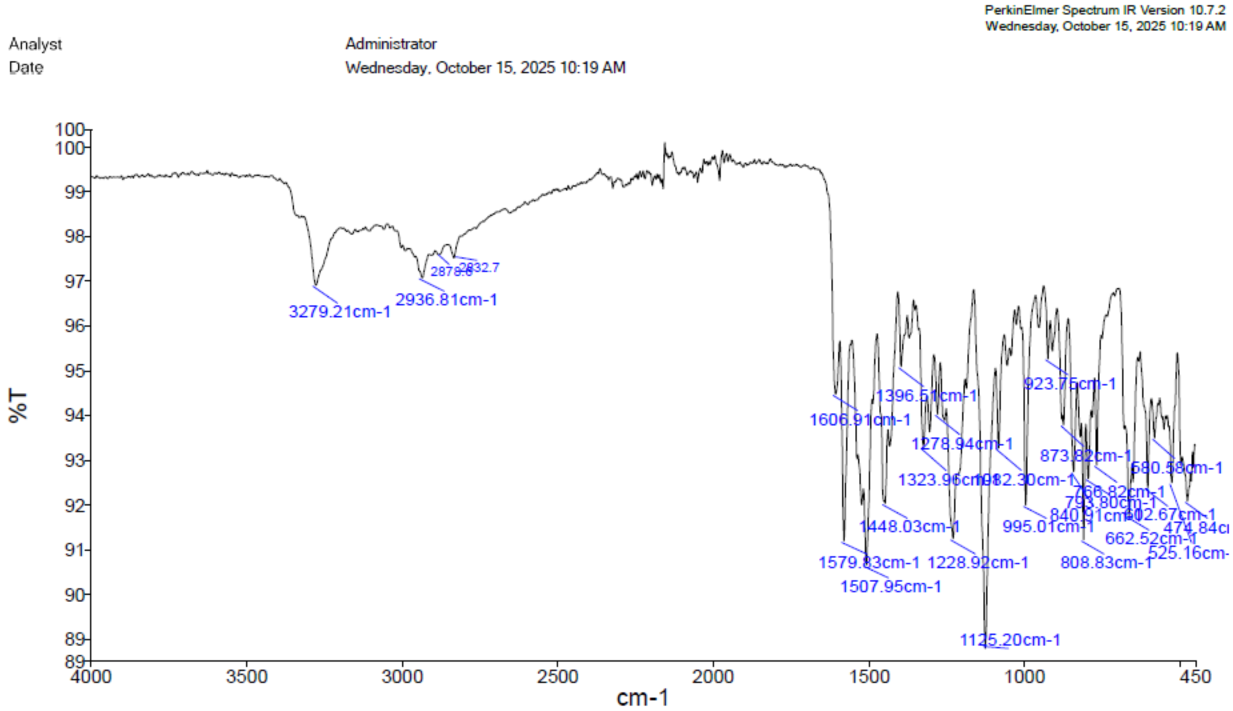
PerkinElmer Spectrum IR Version 10.7.2
Wednesday, October 15, 2025 10:19 AM
Analyst
Administrator
Date
Wednesday, October 15, 2025 10:19 AM
%T
cm-1
3279.21cm-1
2936.81cm-1
1606.91cm-1
1579.83cm-1
1507.95cm-1
1448.03cm-1
1396.51cm-1
1323.96cm-1
1278.94cm-1
1228.92cm-1
1125.20cm-1
995.01cm-1
923.75cm-1
873.82cm-1
808.83cm-1
680.58cm-1
662.52cm-1
525.16cm-

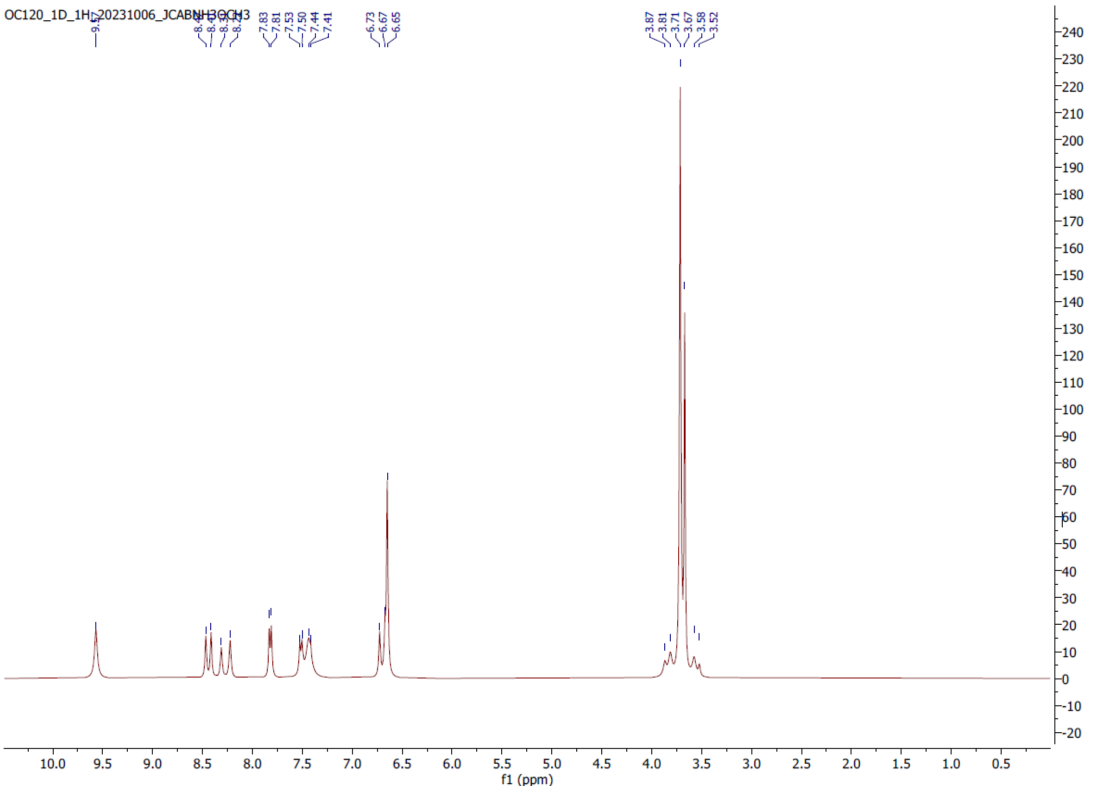
OC120_1D_1H_20231006_JCABNH3OCH3
f1 (ppm)

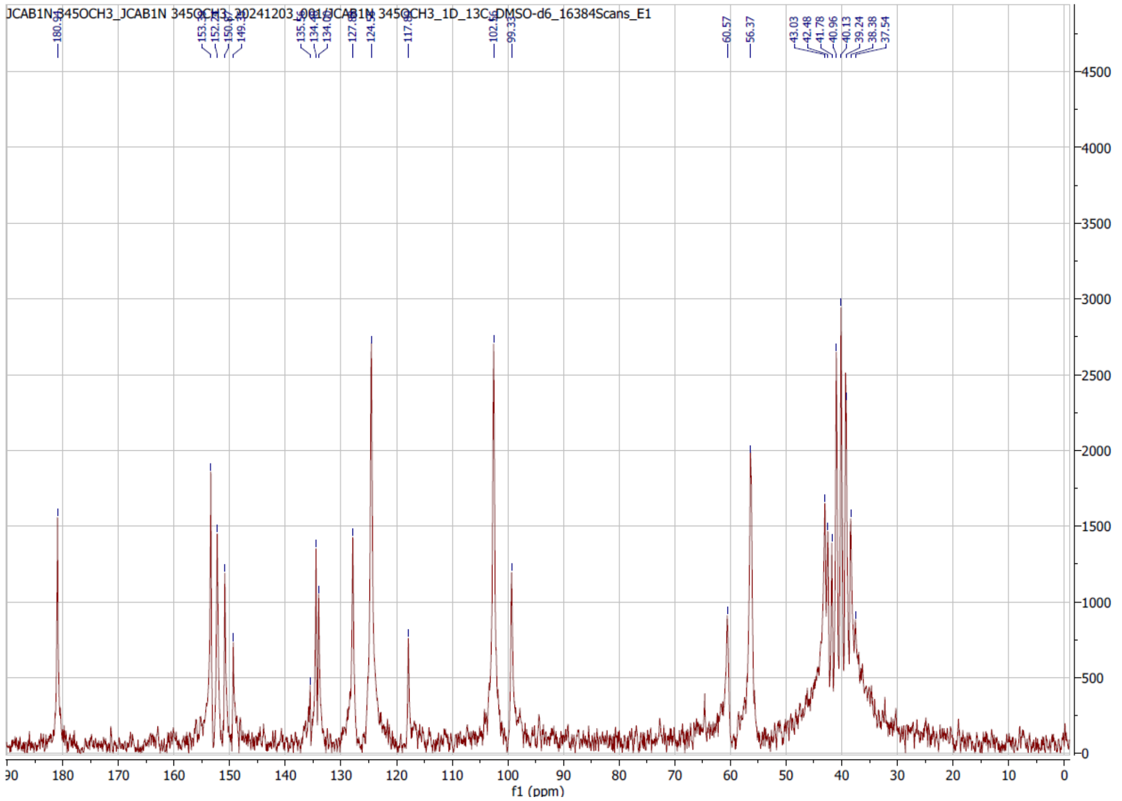
f1 (ppm)

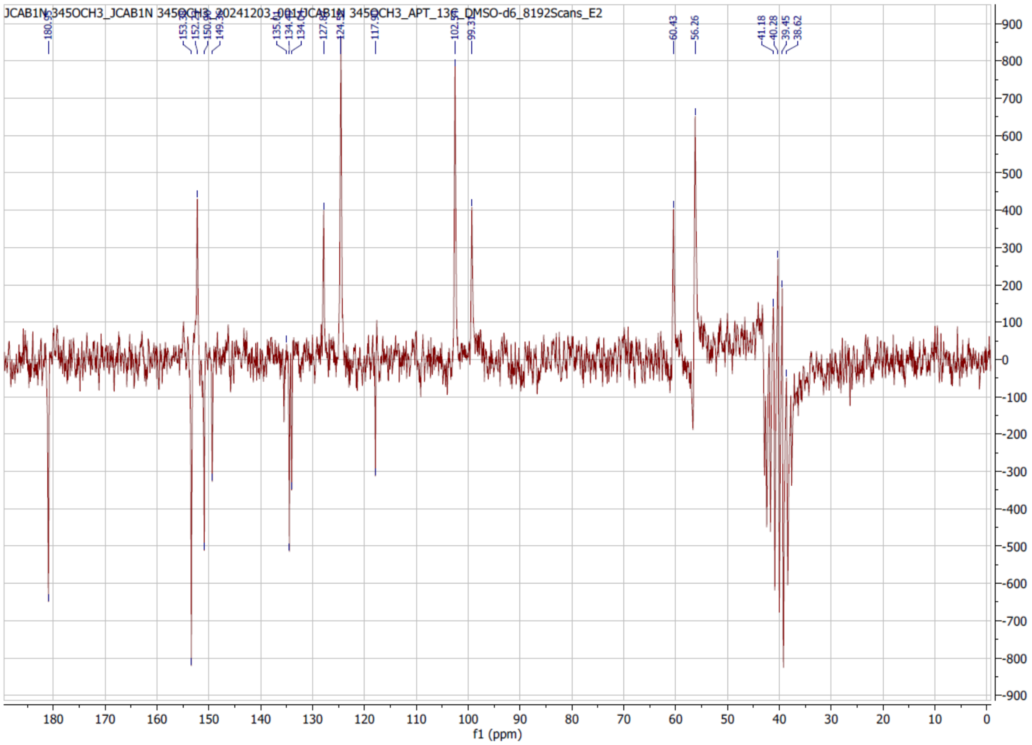
JCAB1N 345OCH3_JCAB1N 345OCH3_20241203_001/JCAB1N 345OCH3_APT_13C_DMSO-d6_8192Scans_E2
60.43
56.26
41.18
40.28
39.45
38.62
f1 (ppm)

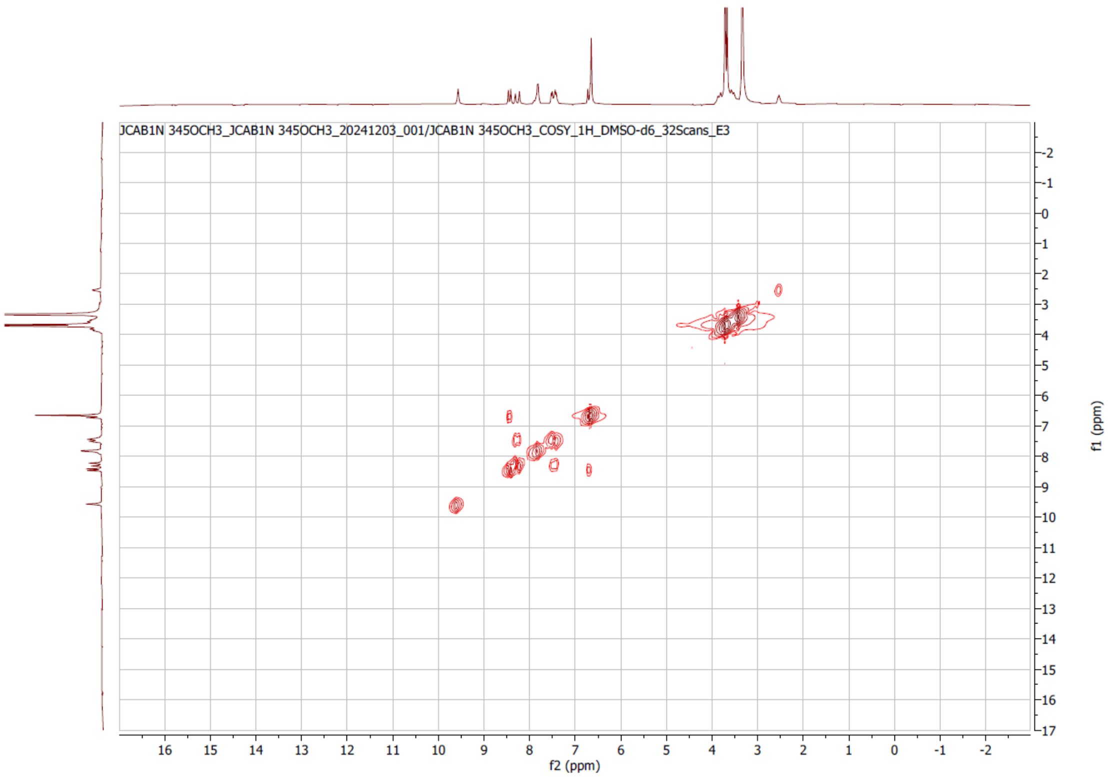
JCAB1N 345OCH3_JCAB1N 345OCH3_20241203_001/JCAB1N 345OCH3_COSY_1H_DMSO-d6_32Scans_E3
f2 (ppm)
f1 (ppm)

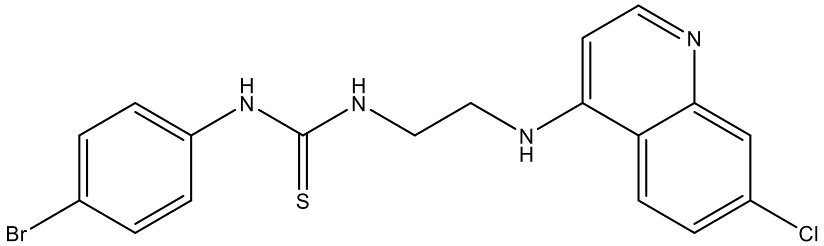

Br
S
H
N
N
H
N
H
Cl

27

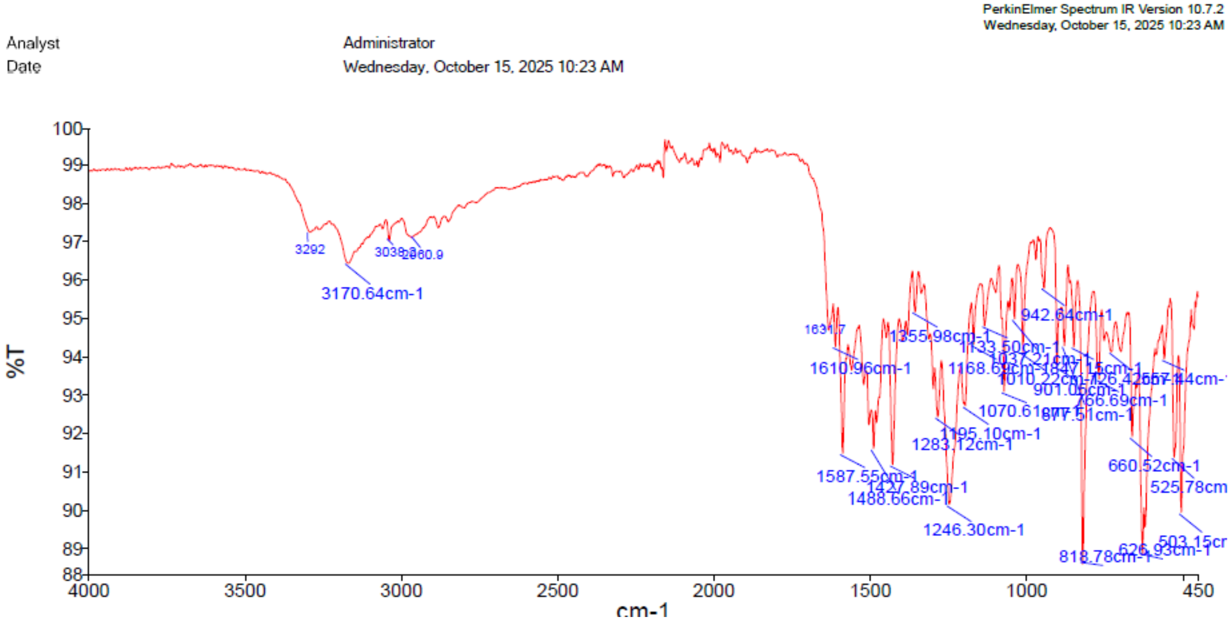

PerkinElmer Spectrum IR Version 10.7.2
Wednesday, October 15, 2025 10:23 AM
Analyst
Administrator
Date
Wednesday, October 15, 2025 10:23 AM
%T
cm-1
3292
3170.64cm-1
942.64cm-1
1355.98cm-1
1610.96cm-1
1070.61cm-1
1195.10cm-1
1283.12cm-1
660.52cm-1
1587.55cm-1
1427.89cm-1
1488.66cm-1
525.78cm
1246.30cm-1
818.78cm-1

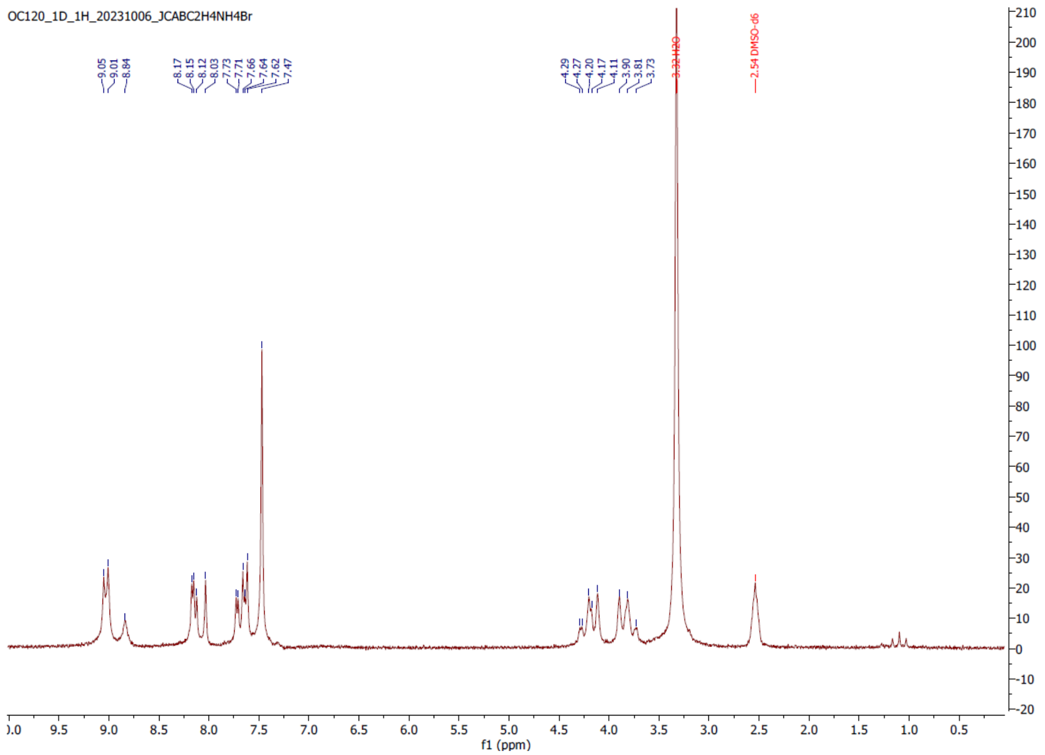
OC120_1D_1H_20231006_JCABC2H4NH4Br
9.05
9.01
8.84
8.17
8.15
8.12
8.03
7.73
7.71
7.66
7.64
7.62
7.47
4.29
4.27
4.20
4.17
4.11
3.90
3.81
3.73
3.32 H2O
2.54 DMSO-d6
f1 (ppm)

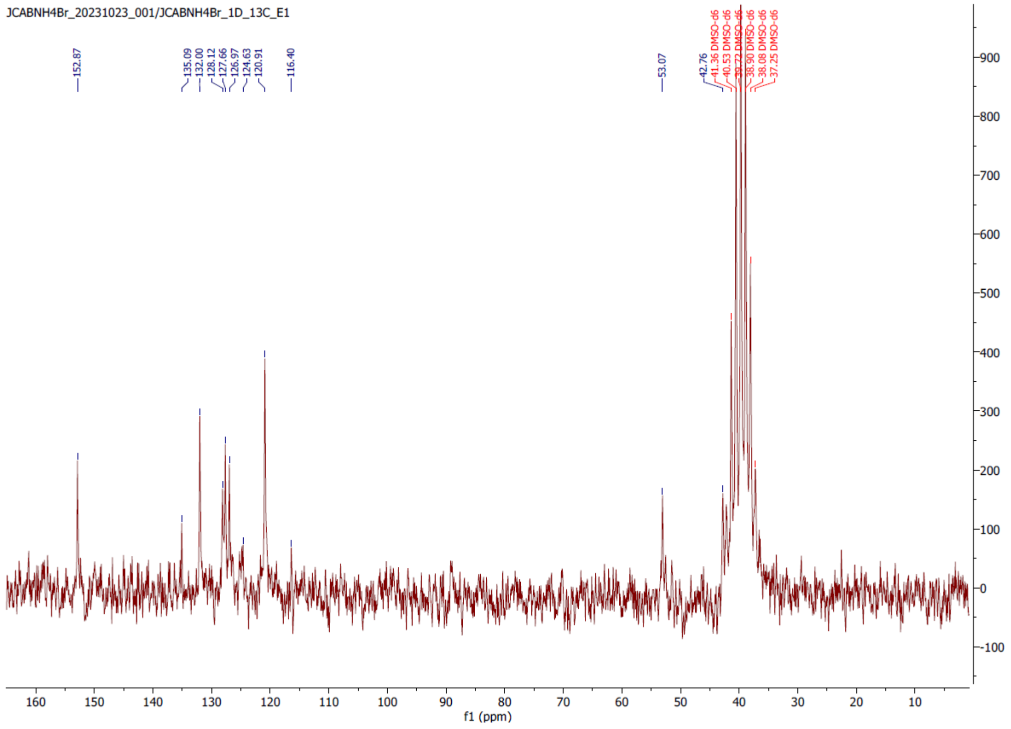
JCABNH4Br_20231023_001/JCABNH4Br_1D_13C_E1
152.87
135.09
132.00
128.12
127.66
126.97
124.63
120.91
116.40
53.07
42.76
41.36 DMSO-d6
40.53 DMSO-d6
39.72 DMSO-d6
38.90 DMSO-d6
38.08 DMSO-d6
37.25 DMSO-d6
f1 (ppm)

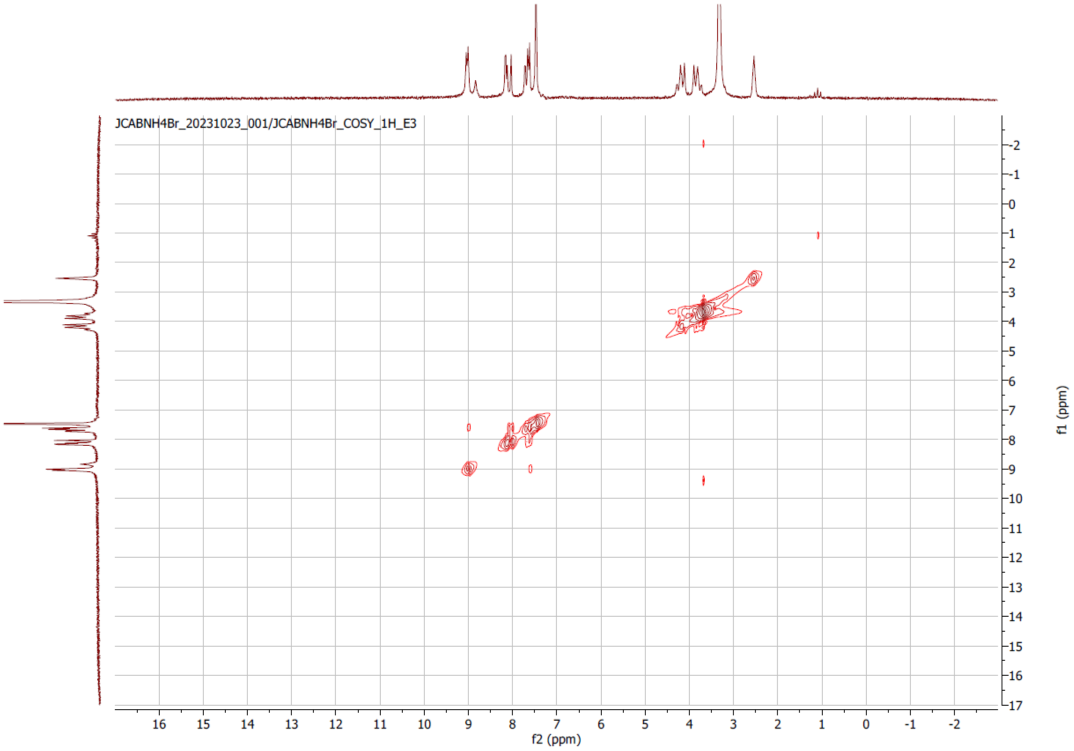
JCABNH4Br_20231023_001/JCABNH4Br_COSY_1H_E3
f2 (ppm)
f1 (ppm)

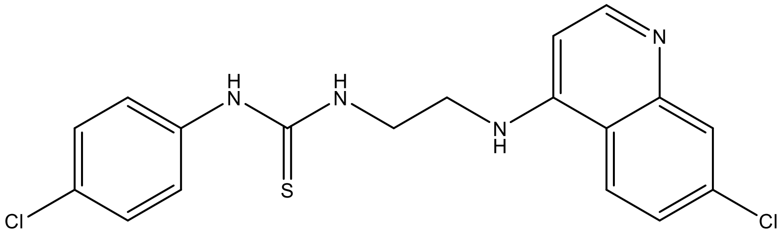

H
N
H
N
N
H
N
S
Cl
Cl

28

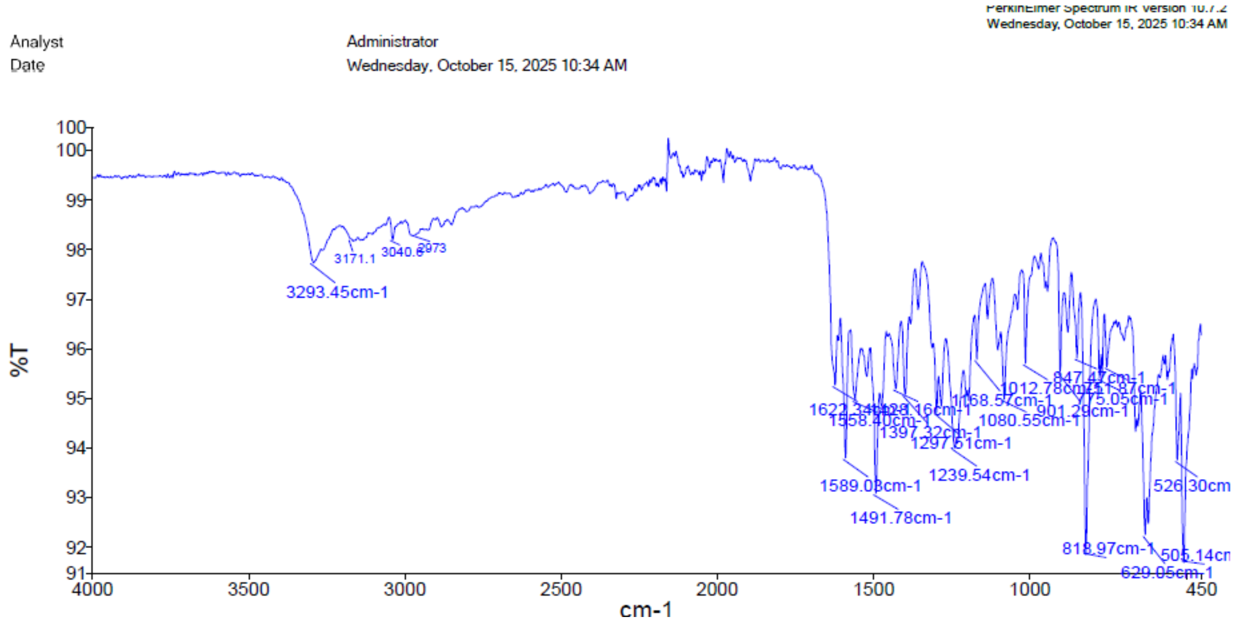

Wednesday, October 15, 2025 10:34 AM
Analyst
Administrator
Date
Wednesday, October 15, 2025 10:34 AM
%T
cm-1
3293.45cm-1
3171.1
1589.08cm-1
1491.78cm-1
1558.40cm-1
1397.32cm-1
1297.51cm-1
1239.54cm-1
1080.55cm-1
1012.78cm-1
901.29cm-1
847.47cm-1
775.05cm-1
818.97cm-1
629.05cm-1
526.30cm

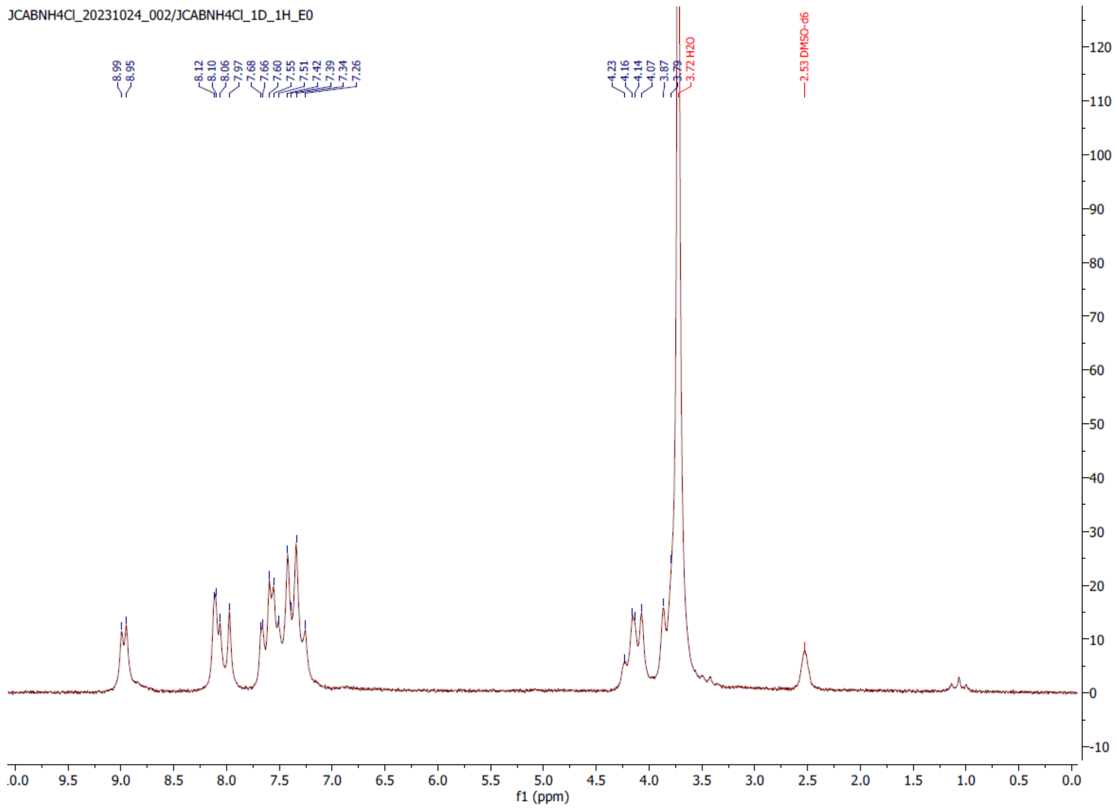
JCABNH4Cl_20231024_002/JCABNH4Cl_1D_1H_E0
8.99
8.95
8.12
8.10
8.06
7.97
7.68
7.66
7.60
7.55
7.51
7.42
7.39
7.34
7.26
4.23
4.16
4.14
4.07
3.87
3.79
3.72 H2O
2.53 DMSO-d6
f1 (ppm)

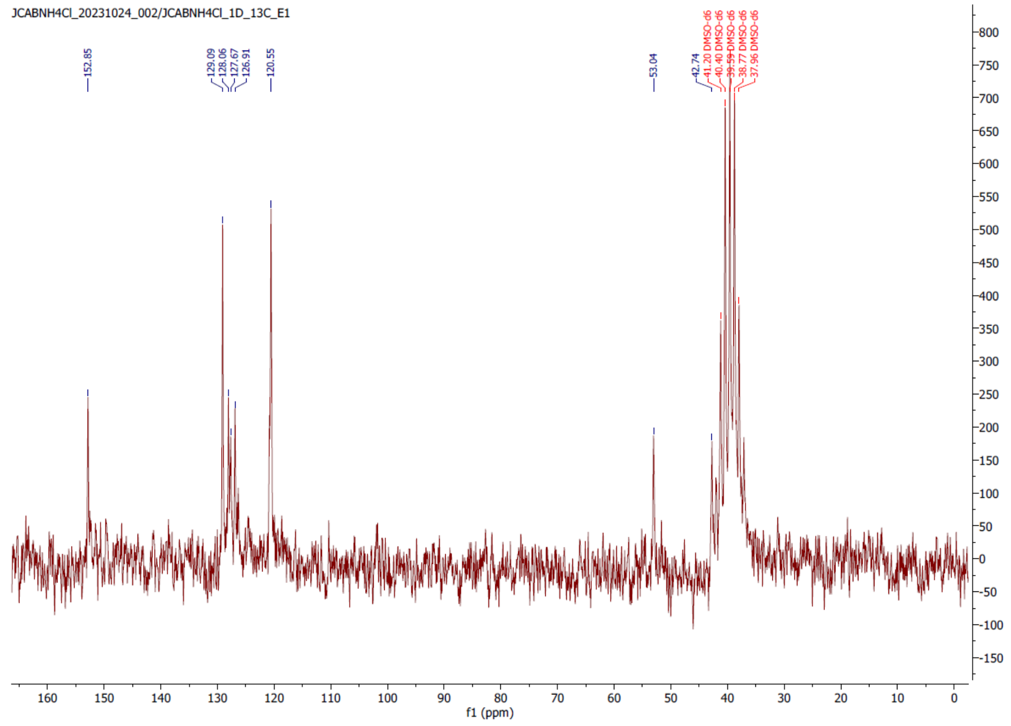
JCABNH4Cl_20231024_002/JCABNH4Cl_1D_13C_E1
152.85
129.09
128.06
127.67
126.91
120.55
53.04
42.74
41.20 DMSO-d6
40.40 DMSO-d6
39.59 DMSO-d6
38.77 DMSO-d6
37.96 DMSO-d6
f1 (ppm)

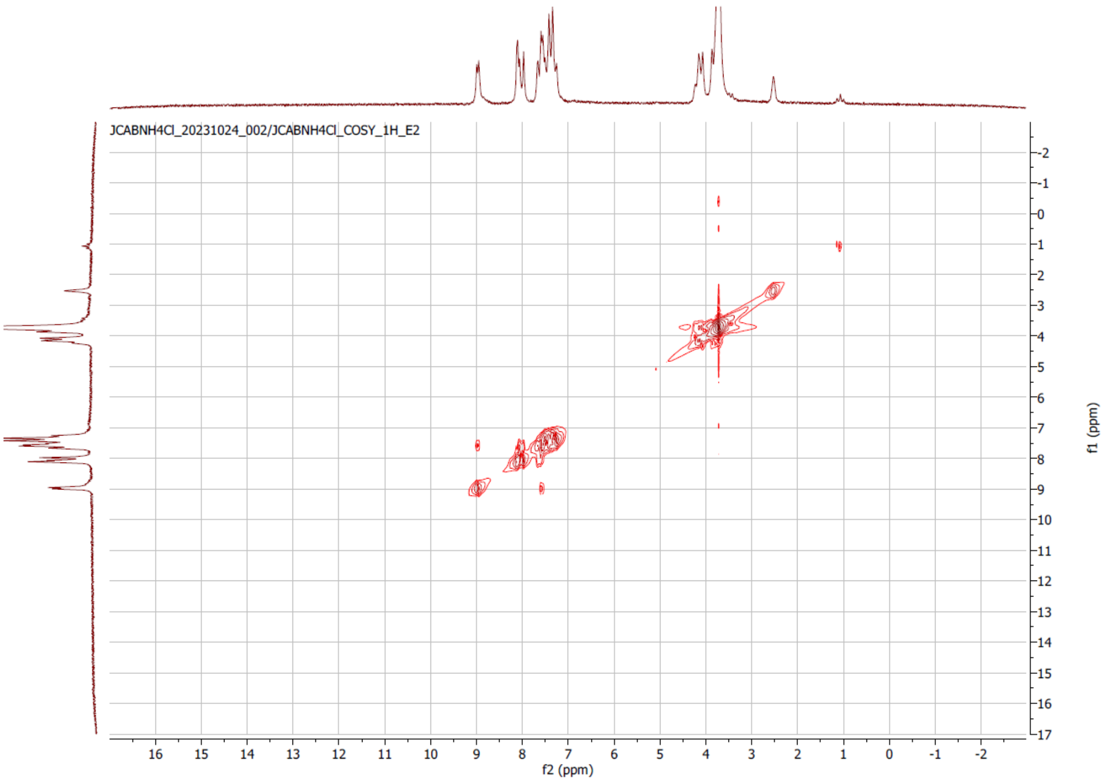
JCABNH4Cl_20231024_002/JCABNH4Cl_COSY_1H_E2
f2 (ppm)
f1 (ppm)

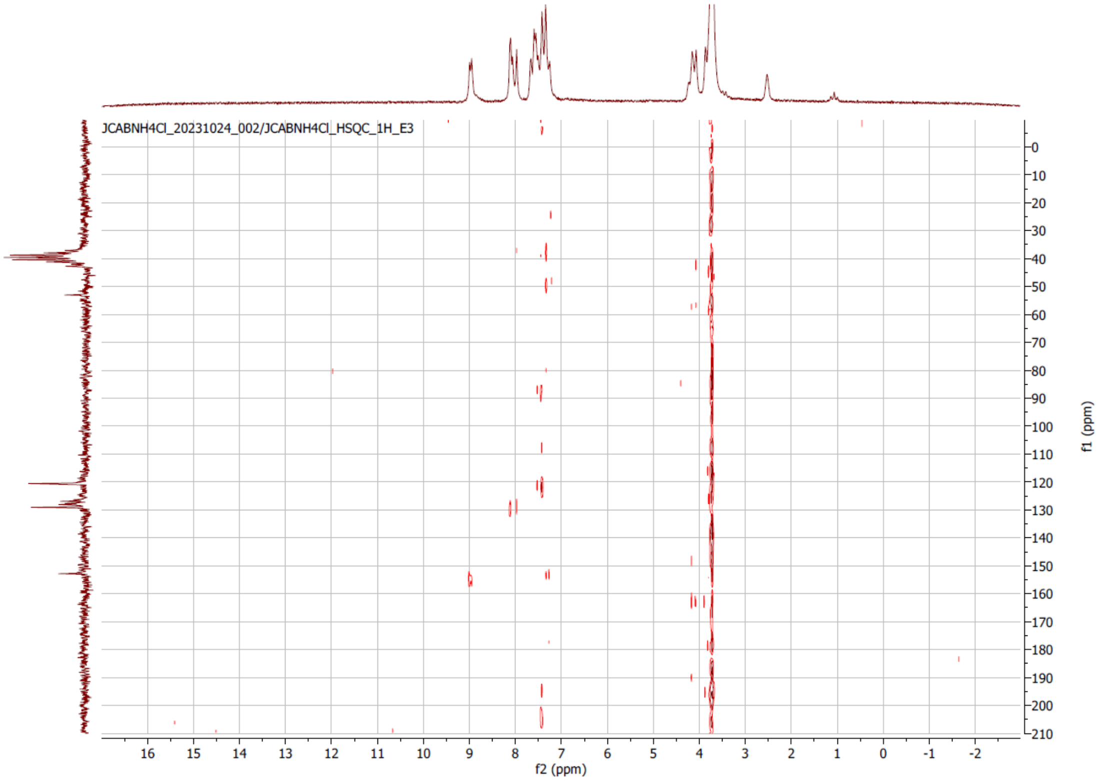
JCABNH4Cl_20231024_002/JCABNH4Cl_HSQC_1H_E3
f2 (ppm)
f1 (ppm)

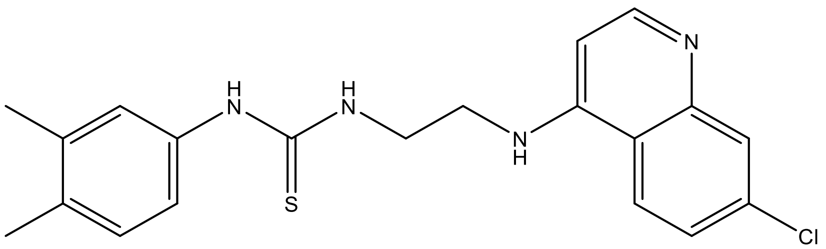
H
N
H
N
S
N
H
N
Cl

29

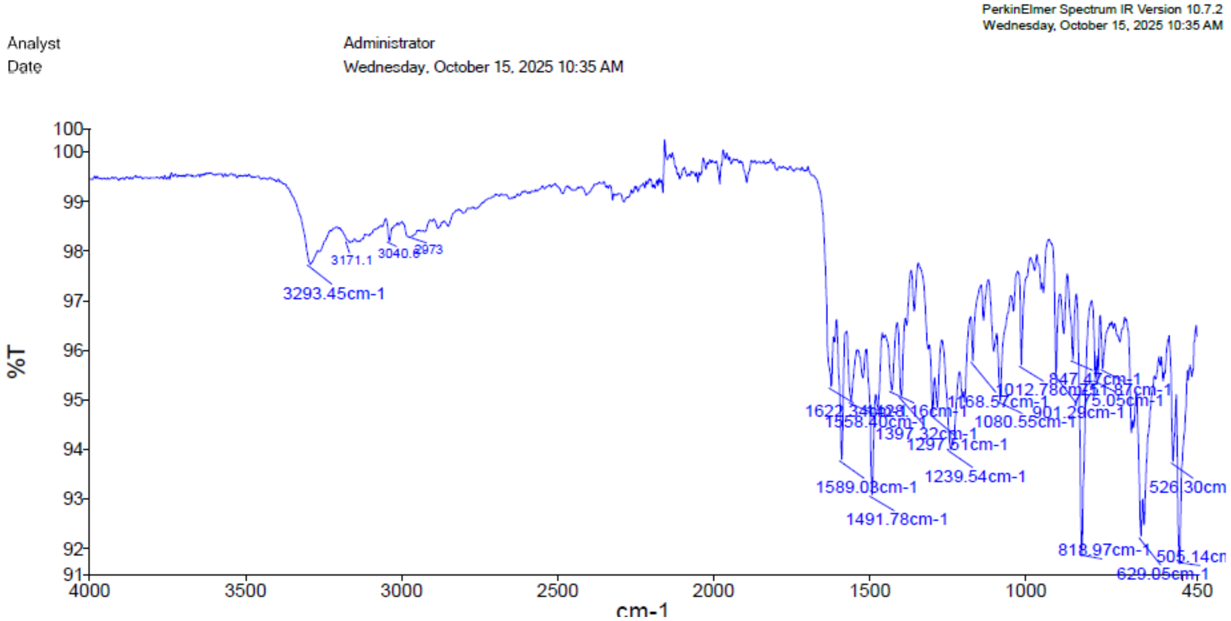
PerkinElmer Spectrum IR Version 10.7.2
Wednesday, October 15, 2025 10:35 AM
Analyst
Administrator
Date
Wednesday, October 15, 2025 10:35 AM
%T
cm-1
3293.45cm-1
3171.1
3040.6
2973
1589.03cm-1
1491.78cm-1
1239.54cm-1
1080.55cm-1
901.29cm-1
818.97cm-1
526.30cm
505.14cm
629.05cm-1

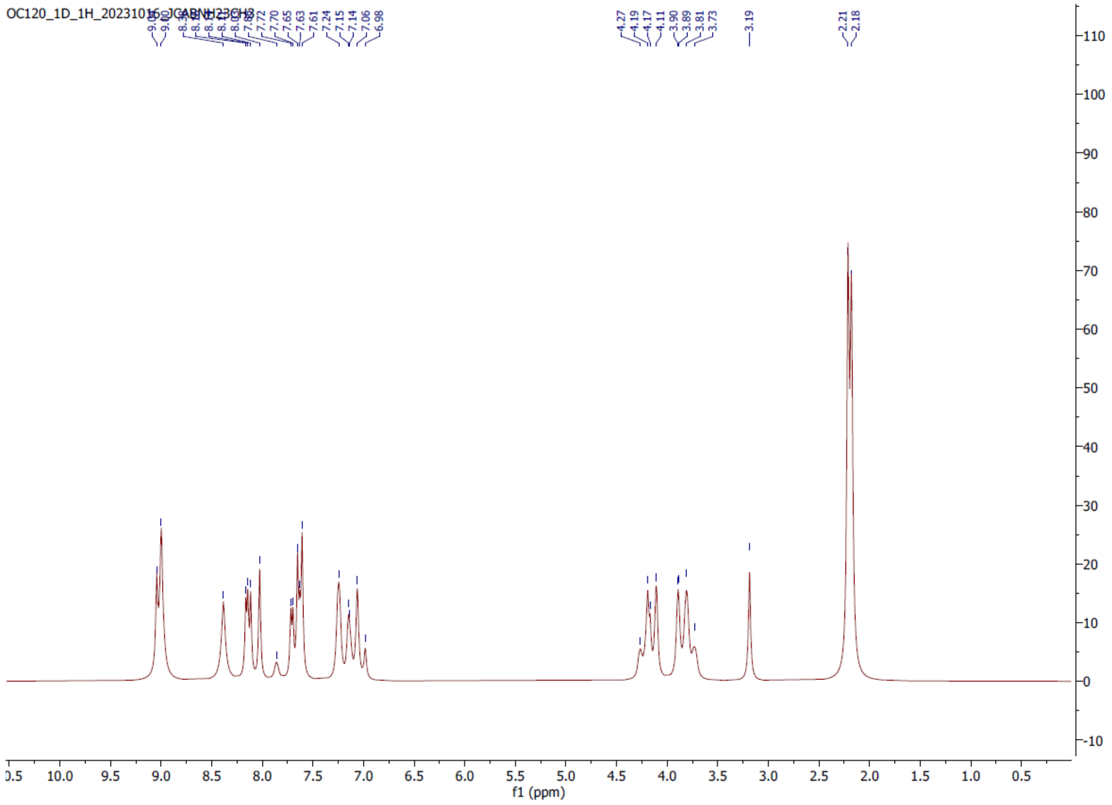
OC120_1D_1H_20231016_JCABNH23CH3
4.27
4.19
4.17
4.11
3.90
3.89
3.81
3.73
3.19
2.21
2.18
f1 (ppm)

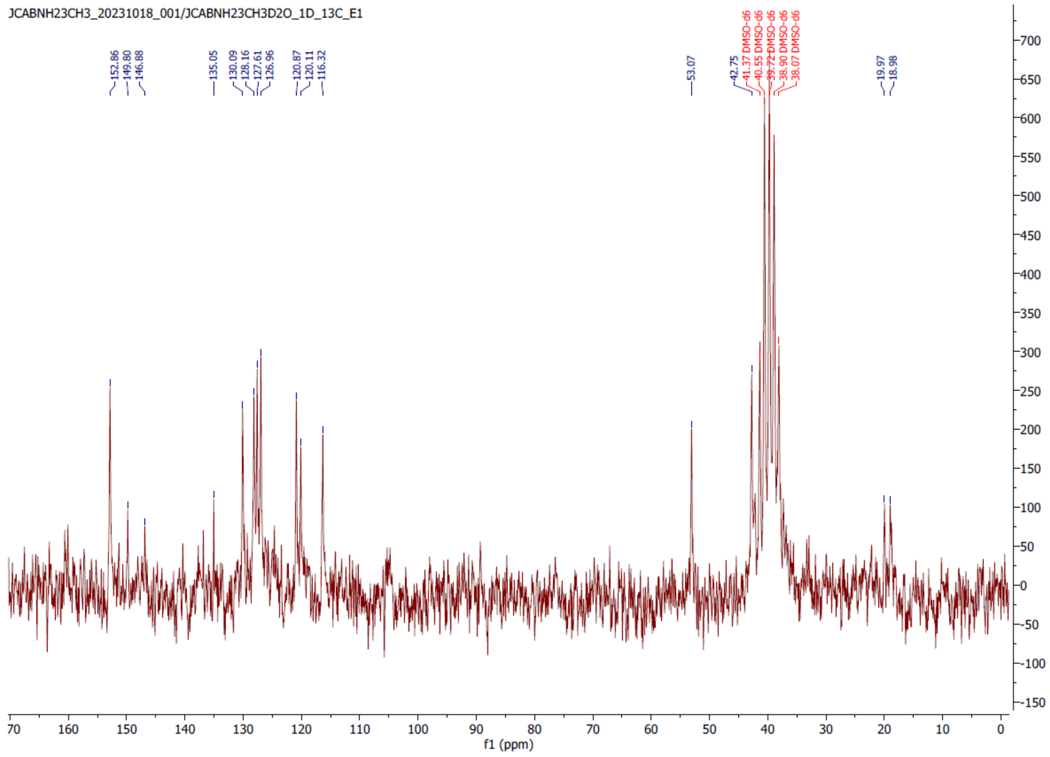
JCABNH23CH3_20231018_001/JCABNH23CH3D2O_1D_13C_E1
152.86
149.80
146.88
135.05
130.09
128.16
127.61
126.96
120.87
120.11
116.32
53.07
42.75
41.37 DMSO-d6
40.55 DMSO-d6
39.72 DMSO-d6
38.90 DMSO-d6
38.07 DMSO-d6
19.97
18.98
f1 (ppm)

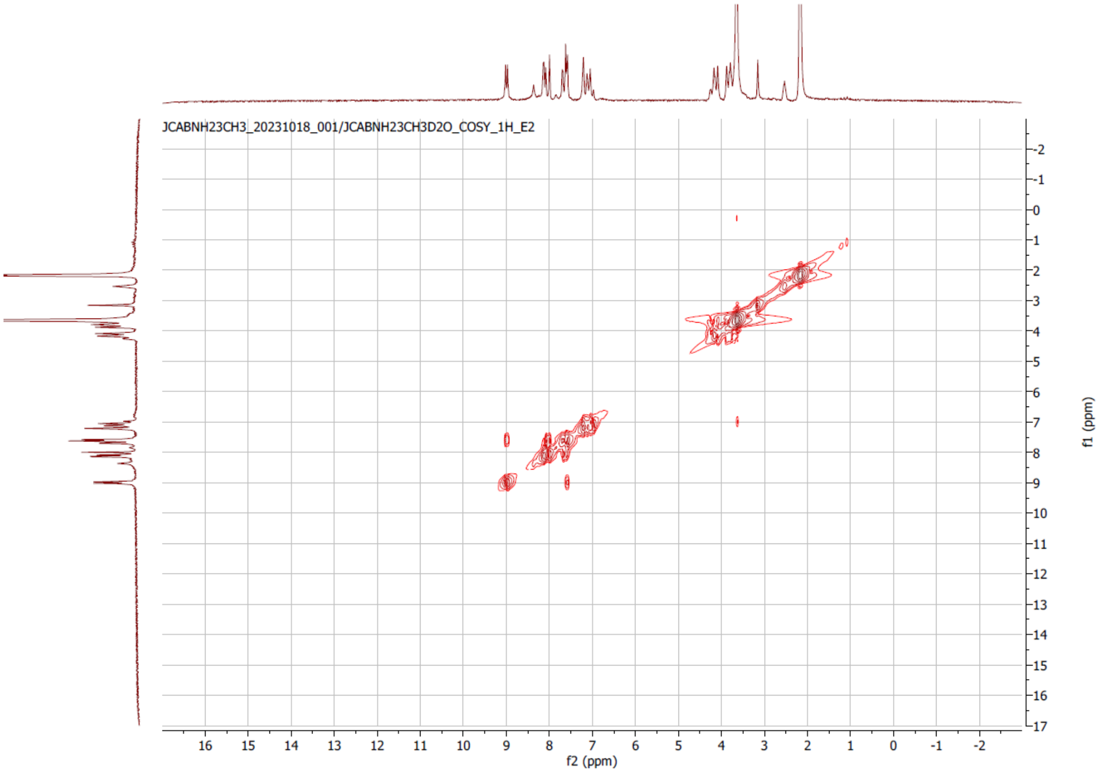
JCABNH23CH3_20231018_001/JCABNH23CH3D2O_COSY_1H_E2
f2 (ppm)
f1 (ppm)

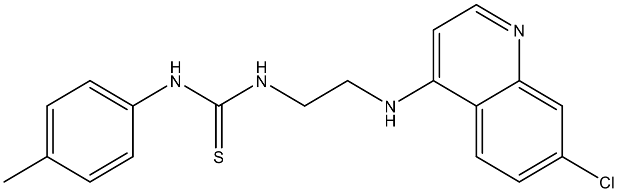

N
H
S
Cl

30

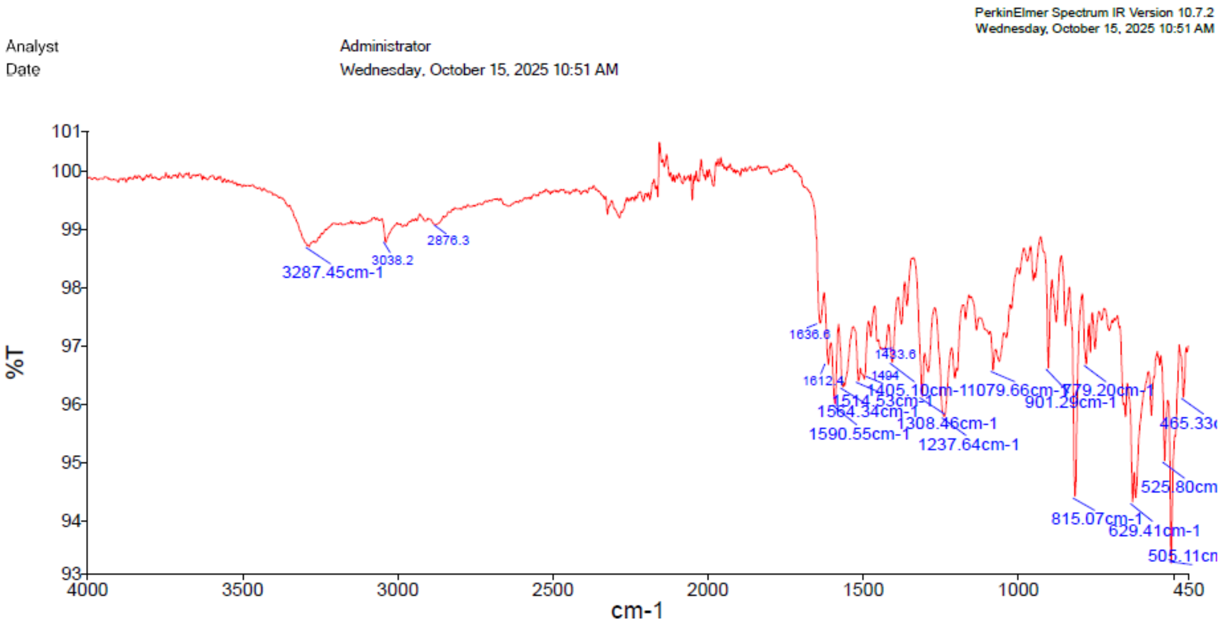

PerkinElmer Spectrum IR Version 10.7.2
Wednesday, October 15, 2025 10:51 AM
Analyst
Administrator
Date
Wednesday, October 15, 2025 10:51 AM
%T
cm-1
3287.45cm-1
3038.2
2876.3
1636.6
1612.4
1590.55cm-1
1564.34cm-1
1514.53cm-1
1433.6
1494
1405.10cm-1
1308.46cm-1
1237.64cm-1
1079.66cm-1
901.29cm-1
779.20cm-1
815.07cm-1
629.41cm-1
525.80cm
505.11cm
465.33

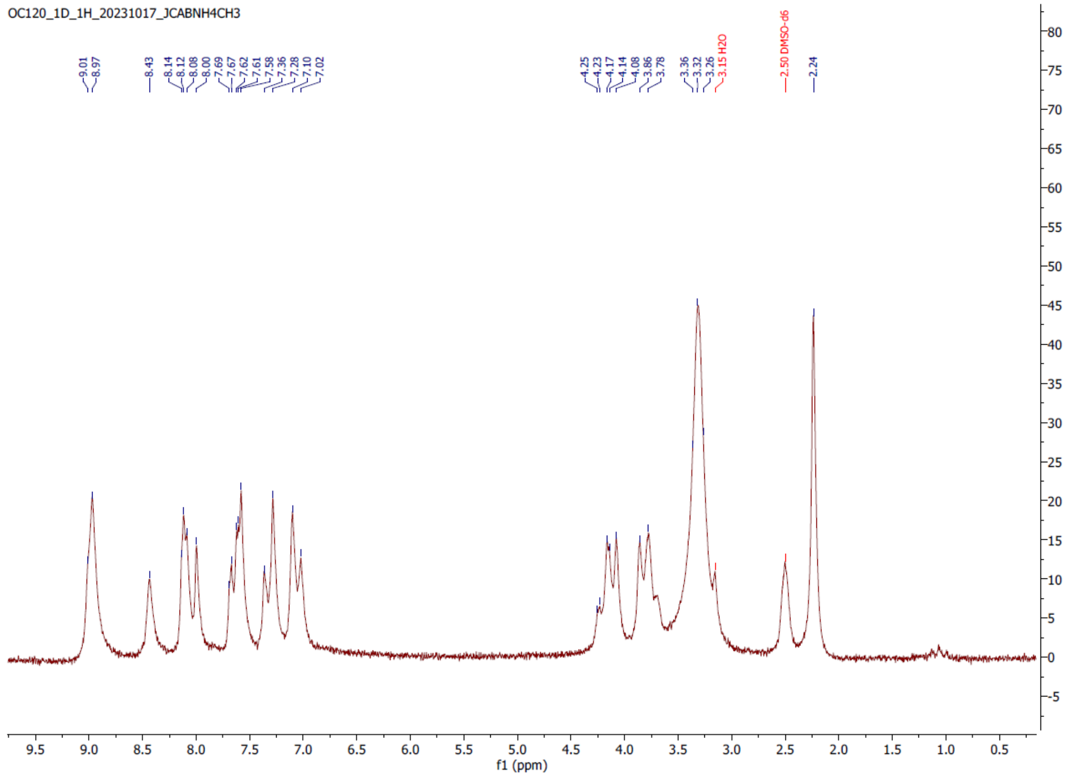
OC120_1D_1H_20231017_JCABNH4CH3
9.01 8.97 8.43 8.14 8.12 8.08 8.00 7.69 7.67 7.62 7.61 7.58 7.36 7.28 7.10 7.02
4.25 4.23 4.17 4.14 4.08 3.86 3.78 3.36 3.32 3.26
3.15 H2O
2.50 DMSO-d6
2.24
f1 (ppm)

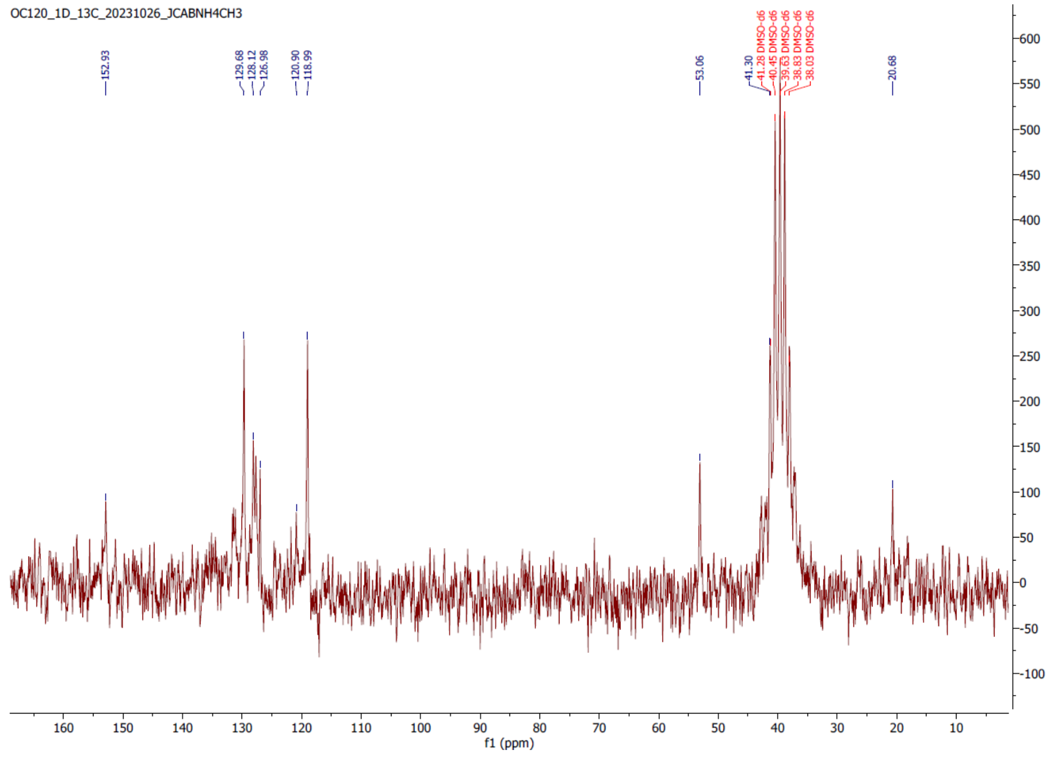
OC120_1D_13C_20231026_JCABNH4CH3
152.93
129.68 128.12 126.98 120.90 118.99
53.06
41.30
41.28 DMSO-d6
40.45 DMSO-d6
39.63 DMSO-d6
38.83 DMSO-d6
38.03 DMSO-d6
20.68
f1 (ppm)

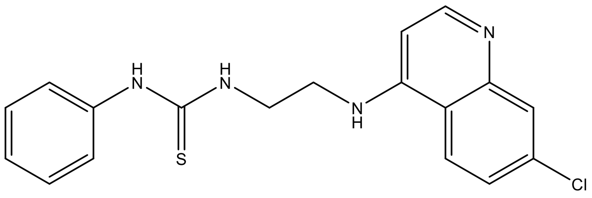

31

PerkinElmer Spectrum IR Version 10.7.2
Wednesday, October 15, 2025 10:54 AM

Analyst Administrator
Date Wednesday, October 15, 2025 10:54 AM

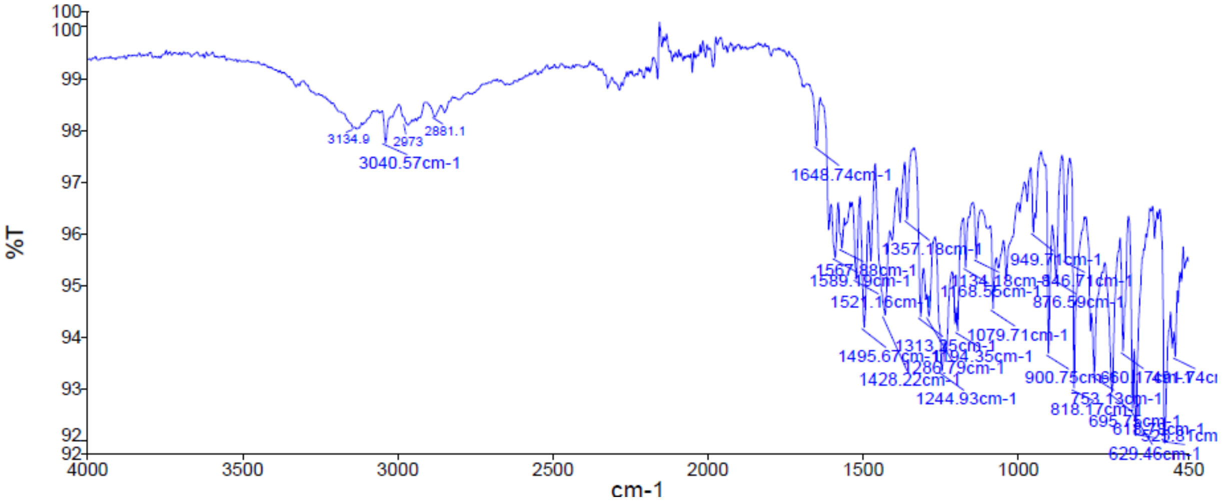

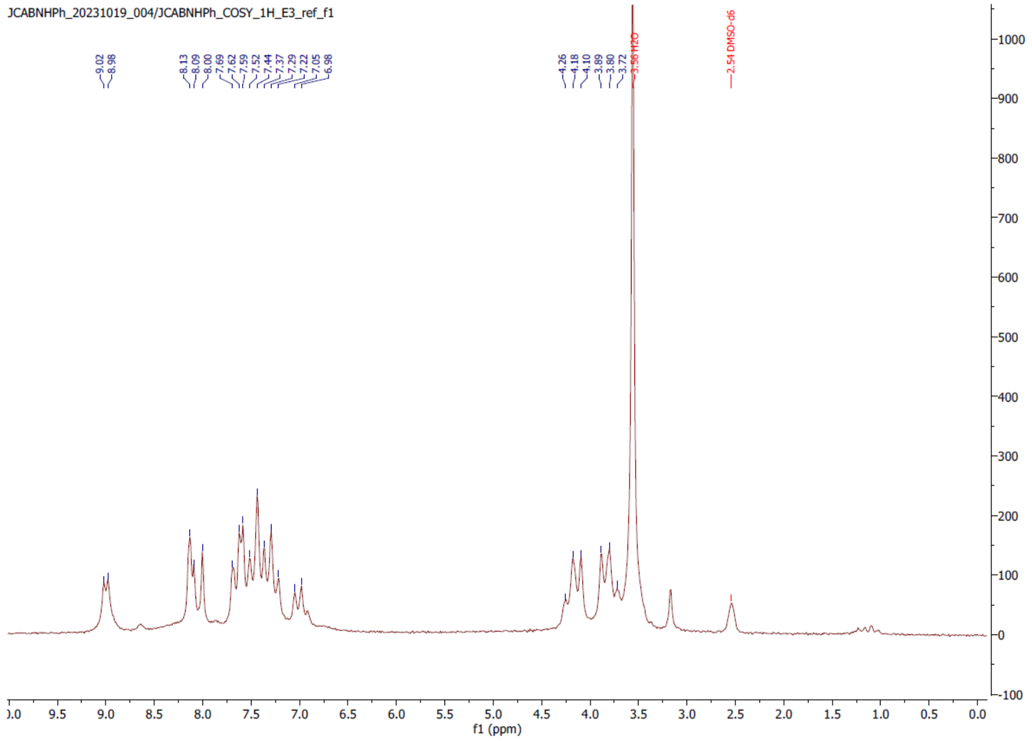
JCABNHPh_20231019_004/JCABNHPh_COSY_1H_E3_ref_f1
9.02
8.98
8.13
8.09
8.00
7.69
7.62
7.59
7.52
7.44
7.37
7.29
7.22
7.05
6.98
4.26
4.18
4.10
3.89
3.80
3.72
3.56 H2O
2.54 DMSO-d6
f1 (ppm)

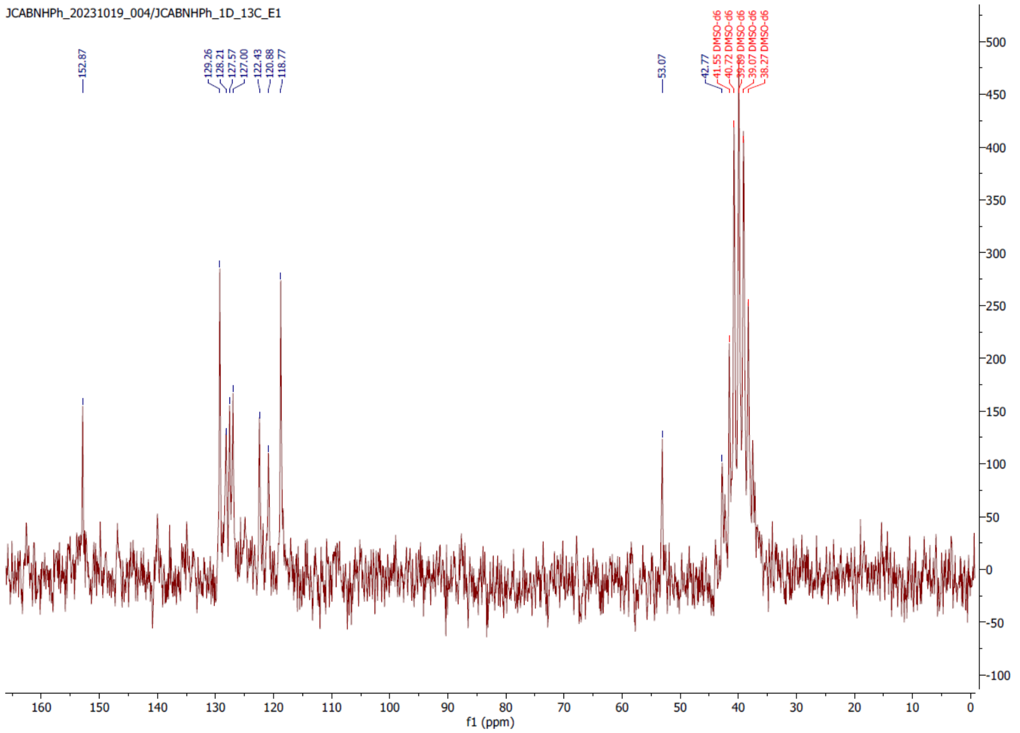
JCABNHPh_20231019_004/JCABNHPh_1D_13C_E1
152.87
129.26
128.21
127.57
127.00
122.43
120.88
118.77
53.07
42.77
41.55 DMSO-d6
40.72 DMSO-d6
39.89 DMSO-d6
39.07 DMSO-d6
38.27 DMSO-d6
f1 (ppm)

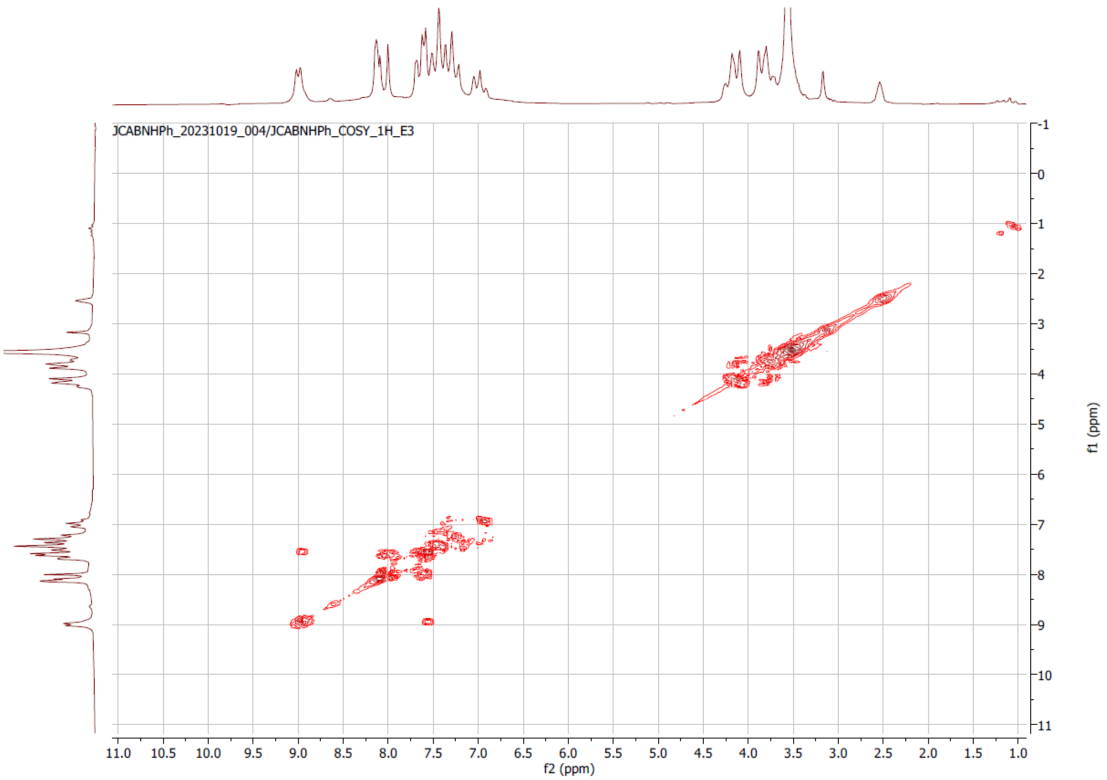
JCABNHPh_20231019_004/JCABNHPh_COSY_1H_E3
f2 (ppm)
f1 (ppm)

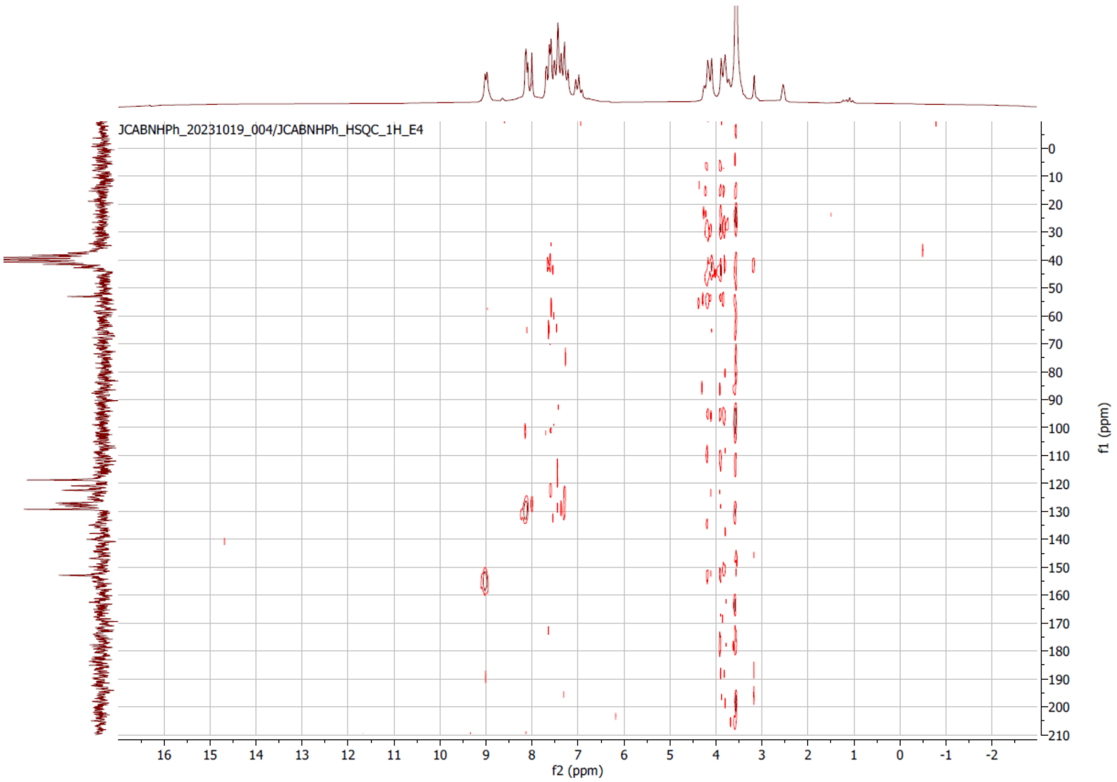
JCABNHPh_20231019_004/JCABNHPh_HSQC_1H_E4
f2 (ppm)
f1 (ppm)

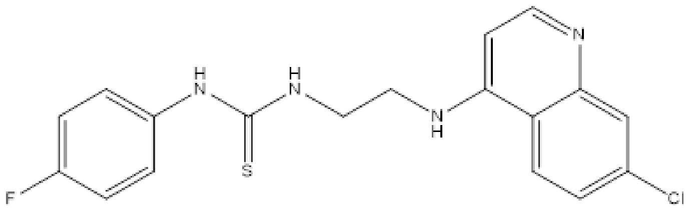

F
H
N
S
H
N
N
H
N
Cl

32

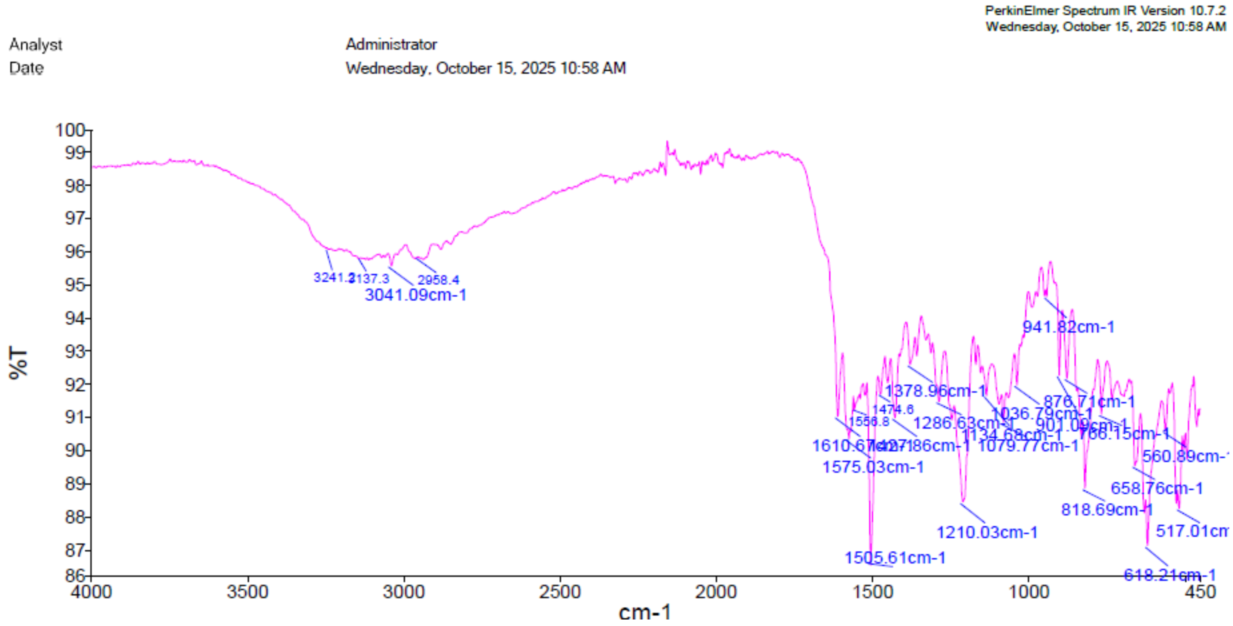

PerkinElmer Spectrum IR Version 10.7.2
Wednesday, October 15, 2025 10:58 AM
Analyst
Administrator
Date
Wednesday, October 15, 2025 10:58 AM
%T
cm-1
3041.09cm-1
941.82cm-1
1378.96cm-1
1210.03cm-1
1505.61cm-1
1575.03cm-1
818.69cm-1
658.76cm-1
517.01cm
618.21cm-1

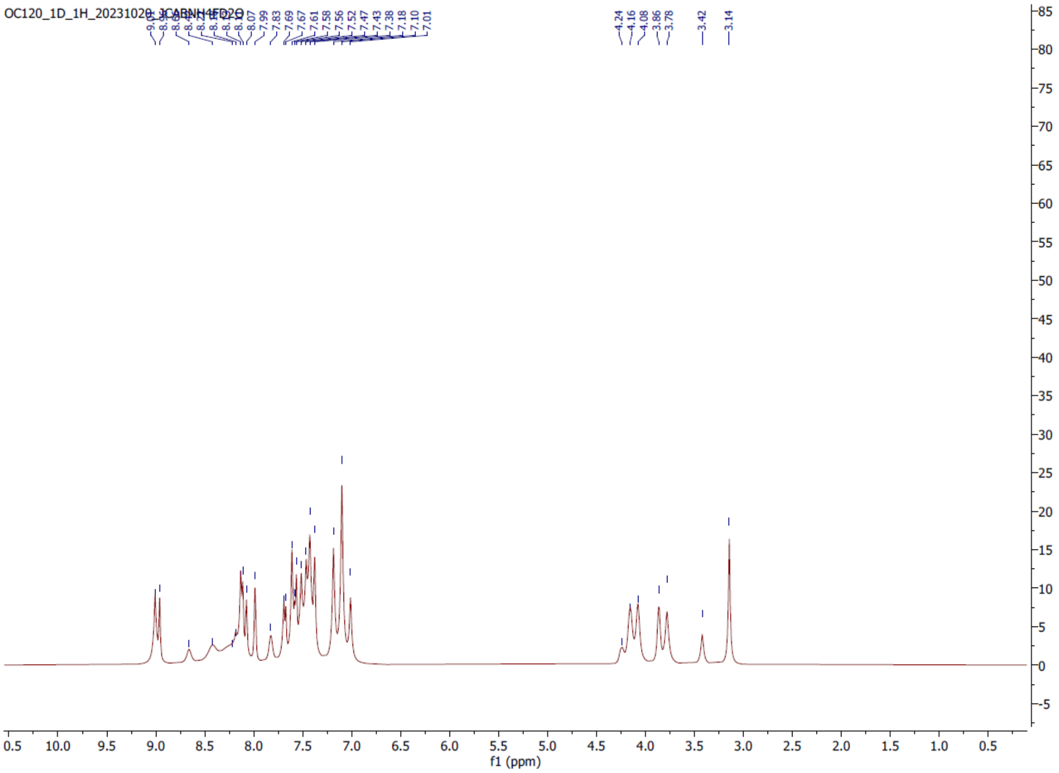
OC120_1D_1H_20231020_JCABNH4FD2O
4.24
4.16
4.08
3.86
3.78
3.42
3.14
f1 (ppm)

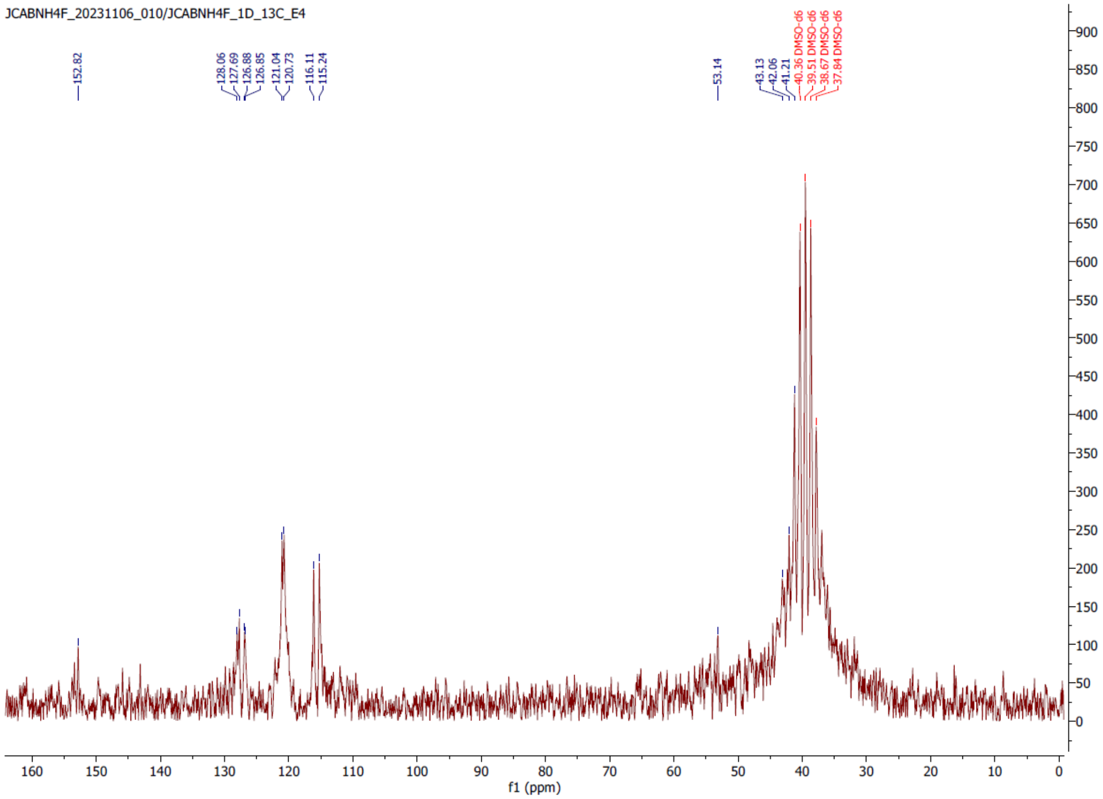
JCABNH4F_20231106_010/JCABNH4F_1D_13C_E4
152.82
128.06
127.69
126.88
126.85
121.04
120.73
116.11
115.24
53.14
43.13
42.06
41.21
40.36 DMSO-d6
39.51 DMSO-d6
38.67 DMSO-d6
37.84 DMSO-d6
f1 (ppm)

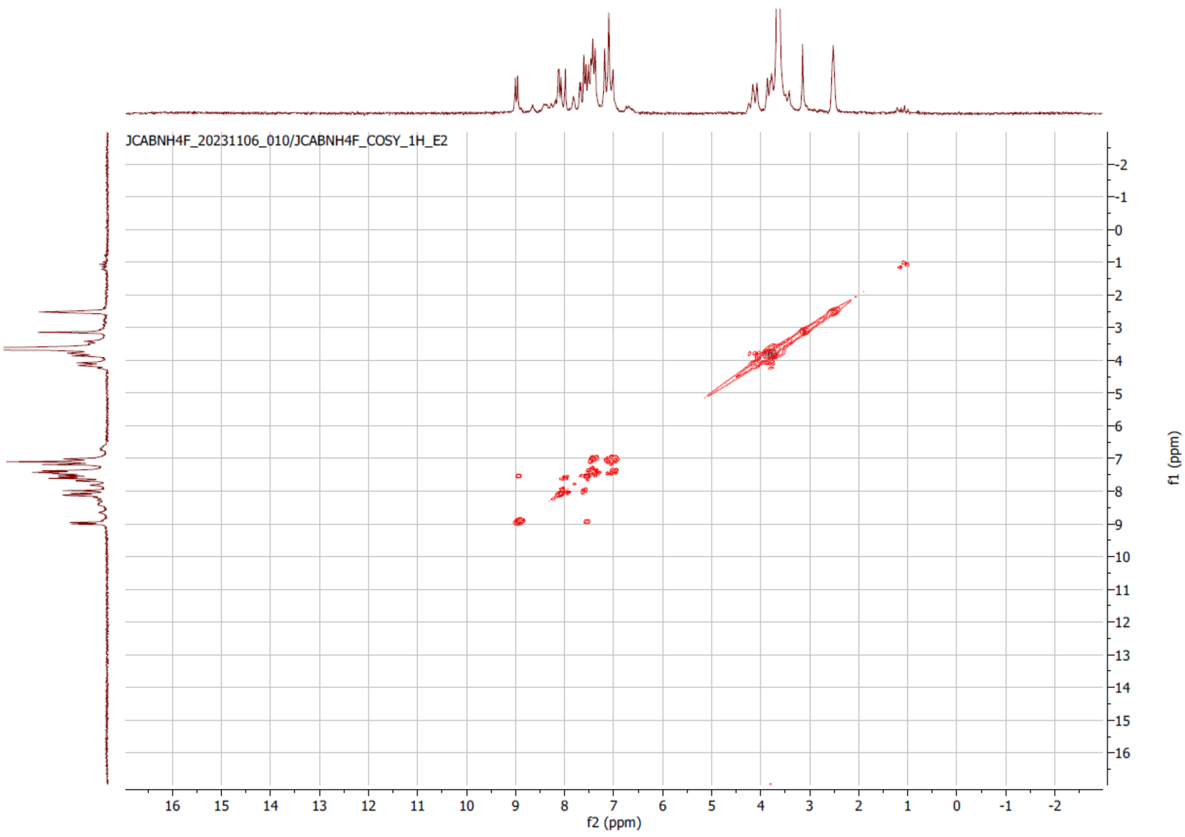

JCABNH4F_20231106_010/JCABNH4F_COSY_1H_E2
f2 (ppm)
f1 (ppm)

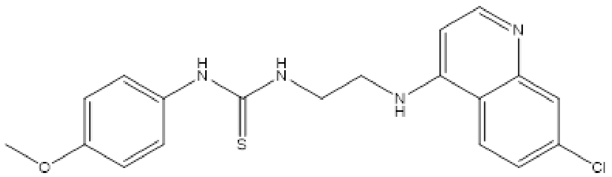

O
H
N
H
N
S
N
H
N
Cl

33

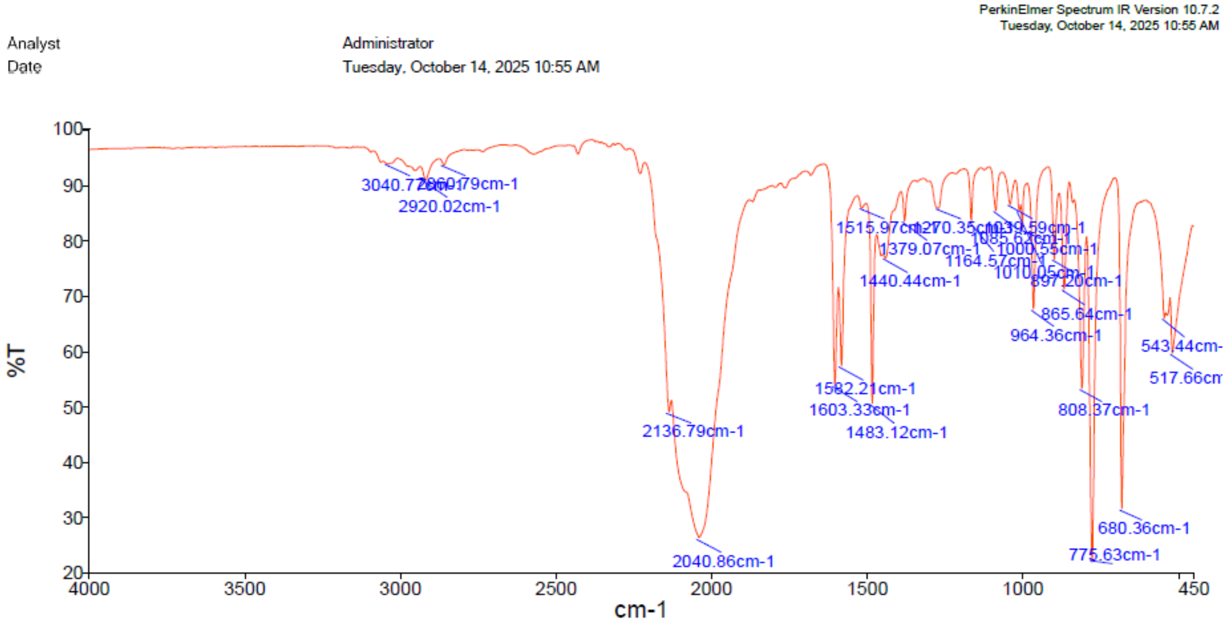

PerkinElmer Spectrum IR Version 10.7.2
Tuesday, October 14, 2025 10:55 AM
Analyst
Administrator
Date
Tuesday, October 14, 2025 10:55 AM
%T
cm-1
2920.02cm-1
2136.79cm-1
2040.86cm-1
1582.21cm-1
1603.33cm-1
1483.12cm-1
1515.97cm-1
1379.07cm-1
1440.44cm-1
1164.57cm-1
1000.55cm-1
897.20cm-1
865.64cm-1
964.36cm-1
808.37cm-1
680.36cm-1
775.63cm-1

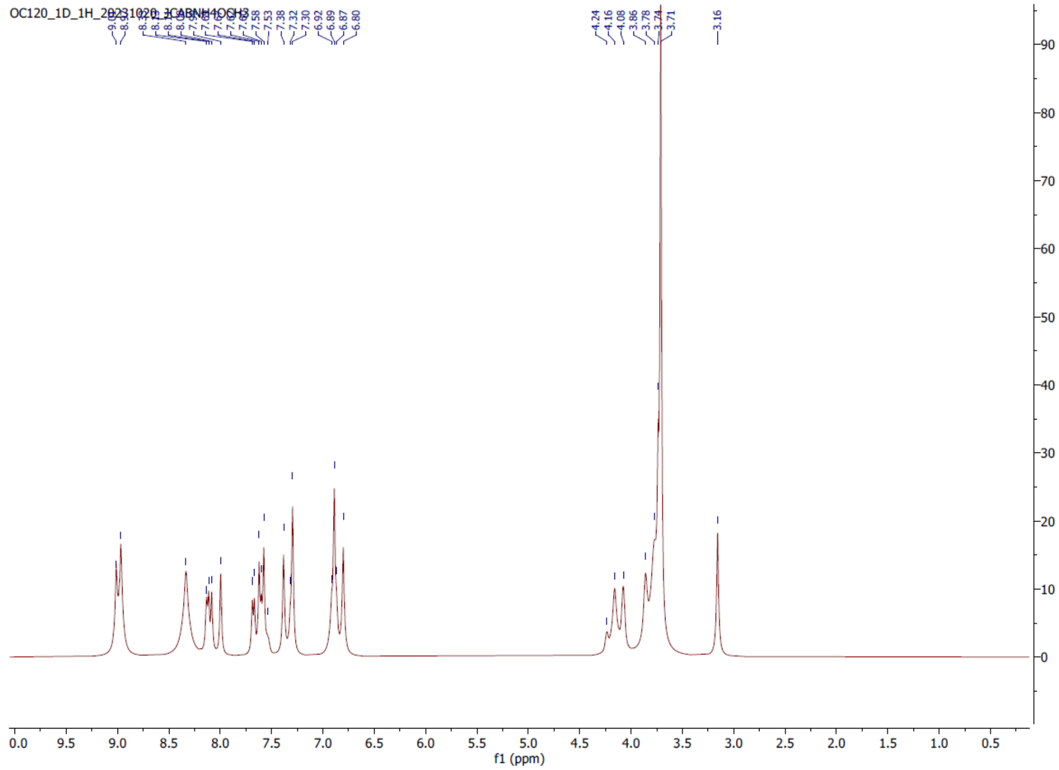
OC120_1D_1H_20231020_JCABNH4OCH3
7.53
7.38
7.32
7.30
6.92
6.89
6.87
6.80
4.24
4.16
4.08
3.86
3.78
3.74
3.71
3.16
f1 (ppm)

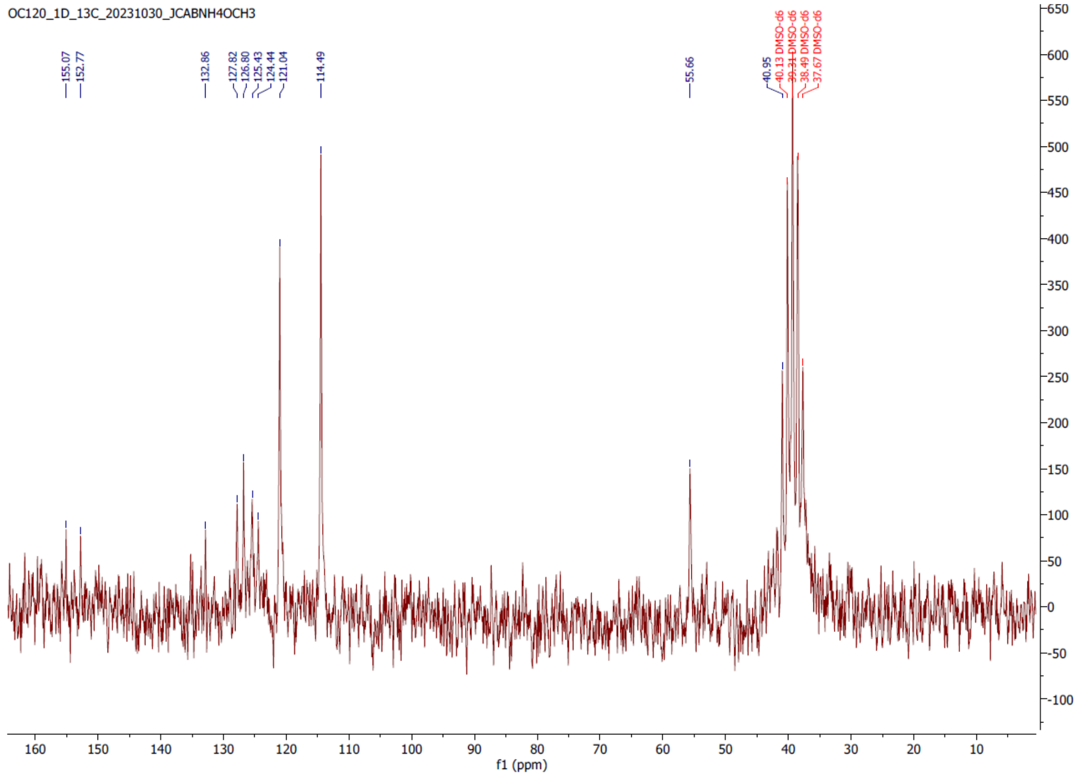
OC120_1D_13C_20231030_JCABNH4OCH3
155.07
152.77
132.86
127.82
126.80
125.43
124.44
121.04
114.49
55.66
40.95
40.13 DMSO-d6
39.31 DMSO-d6
38.49 DMSO-d6
37.67 DMSO-d6
f1 (ppm)

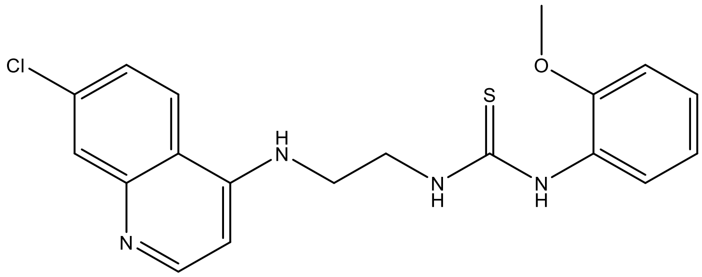
Cl
H
N
N
H
S
N
H
O
N

34

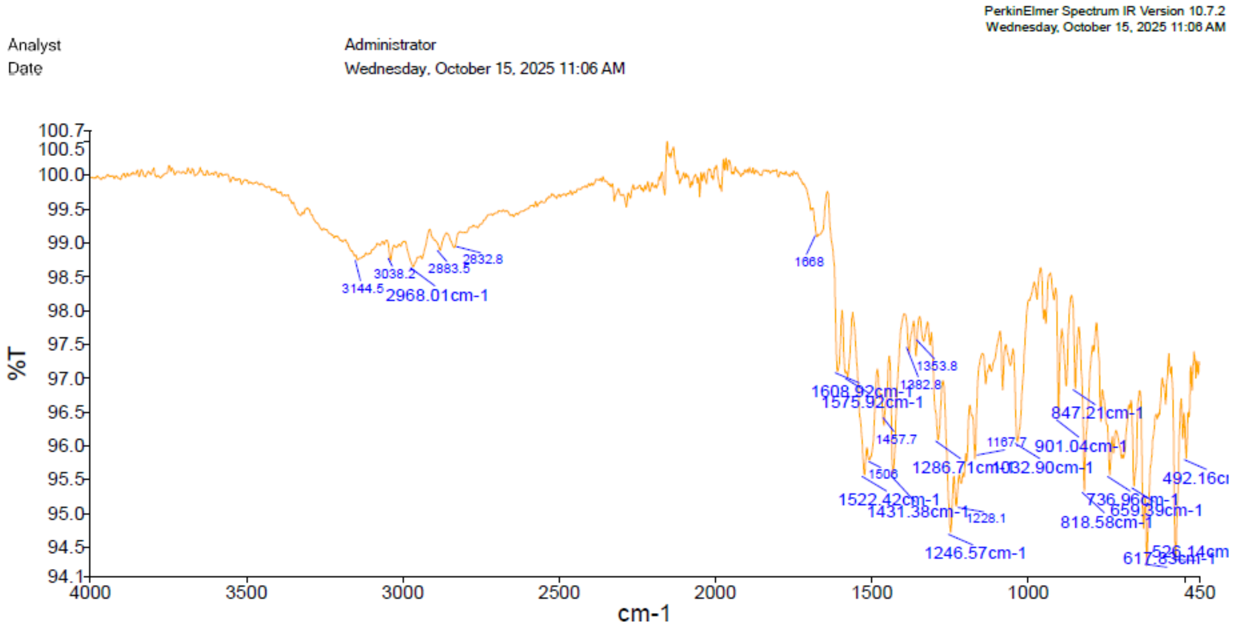
PerkinElmer Spectrum IR Version 10.7.2
Wednesday, October 15, 2025 11:06 AM
Analyst
Administrator
Date
Wednesday, October 15, 2025 11:06 AM
%T
cm-1
3144.5
3038.2
2968.01cm-1
2883.5
2832.8
1668
1608.92cm-1
1575.92cm-1
1522.42cm-1
1506
1457.7
1431.38cm-1
1382.8
1353.8
1286.71cm-1
1246.57cm-1
1228.1
1167.7
1032.90cm-1
901.04cm-1
847.21cm-1
818.58cm-1
736.96cm-1
659.39cm-1
617.23cm-1
526.14cm
492.16cı

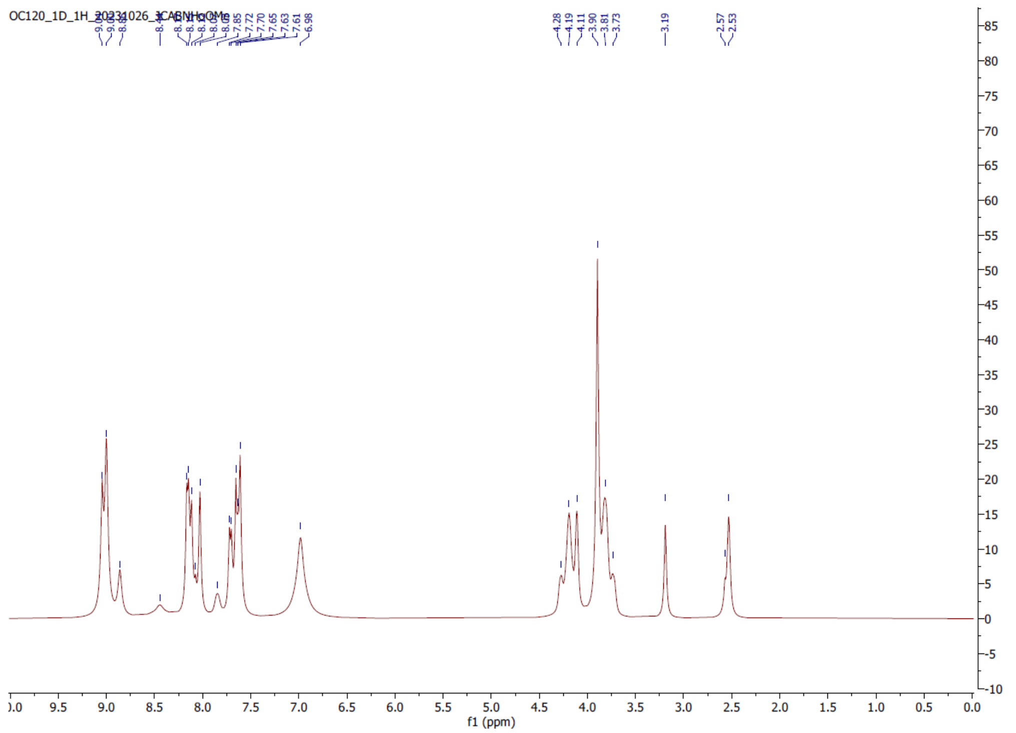
OC120_1D_1H_20231026_JCABNH2OMe
7.85
7.72
7.70
7.65
7.63
7.61
6.98
4.28
4.19
4.11
3.90
3.81
3.73
3.19
2.57
2.53
f1 (ppm)

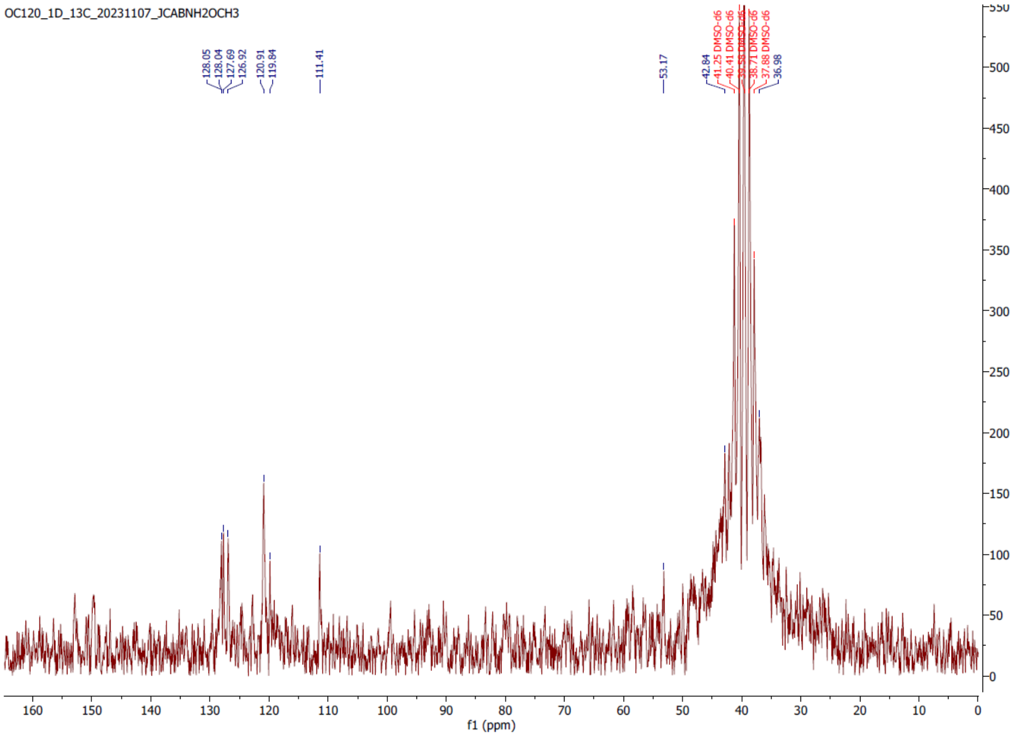
OC120_1D_13C_20231107_JCABNH2OCH3
128.05
128.04
127.69
126.92
120.91
119.84
111.41
53.17
42.84
41.25 DMSO-d6
40.41 DMSO-d6
38.71 DMSO-d6
37.88 DMSO-d6
36.98
f1 (ppm)
